# Phylogeographic Patterns Are Strongly Associated With Biogeographic Patterns in the Irano‐Anatolian Global Biodiversity Hotspot

**DOI:** 10.1111/mec.70355

**Published:** 2026-04-29

**Authors:** Jalil Noroozi, Dennis Larsson, Amir Talebi, Sorour Rahmanian, Moslem Doostmohammadi, Tahereh Setayesh, Dominik Metschina, Ovidiu Paun, Gerald M. Schneeweiss

**Affiliations:** ^1^ Department of Botany and Biodiversity Research University of Vienna Vienna Austria; ^2^ School of Biology and Center of Excellence in Phylogeny of Living Organisms, College of Science University of Tehran Tehran Iran; ^3^ German Centre for Integrative Biodiversity Research (iDiv), Halle‐Jena‐Leipzig Leipzig Germany; ^4^ Department of Biology, Faculty of Sciences Shahid Bahonar University of Kerman Kerman Iran; ^5^ Biology Department College of Charleston Charleston South Carolina USA

## Abstract

Biogeographic breaks, that is, shifts in overall species composition, are expected to be associated with phylogeographic breaks because of shared ecological or evolutionary factors operating at both the interspecific and intraspecific level. Here, we test the hypothesis that biogeographic and phylogeographic boundaries are congruent using mountain species of the Iranian Plateau as our model system. To this end, we analysed the genetic structure from RAD‐sequencing data of four montane (i.e., mid‐elevation) and five alpine plant species endemic to yet widely distributed in the Iranian mountains. Phylogeographic boundaries (breaks) were inferred via the Monmonier maximum difference algorithm and compared to biogeographic breaks identified previously based on floristic data. Major phylogeographic break zones, supported by several montane and alpine species, were identified between Alborz and Zagros as well as between the Azerbaijan Plateau and Zagros (each of those areas corresponding to an area of endemism), thus supporting the biogeography and phylogeography concordance hypothesis. Deviations from this pattern of congruence between biogeographic and phylogeographic breaks mostly concern the presence of additional phylogeographic breaks within areas of endemism. Moreover, the genetic structure is stronger in alpine than in montane species, which can at least partly be attributed to the stronger isolation of high‐elevation habitats acting as sky islands.

## 1 Introduction

Wallace (1876) was among the first biogeographers to identify and map biogeographic breaks (boundaries) in areas of transition between biogeographical regions. These breaks are characterized by shifts in overall species composition (Takhtajan 1986) and result from historical and ecological processes that restrict the distribution of a large fraction of species (Antonelli 2017; Cox and Moore 2000; Ma et al. 2021; Morrone 2009). Typically associated with environmental discontinuities (Djamali et al. 2012; Ficetola et al. 2017; Wieters et al. 2012), these breaks are often caused by continental drift, climatic differences, or the presence of mountain chains (Antonelli 2017). On a global scale, Ficetola et al. (2017) found that tectonic movements, sharp changes in climate conditions, and orographic barriers are the main determinants of current zoogeographic boundaries. Similarly, in their study on Andean birds, Hazzi et al. (2018) highlighted that bioregional boundaries of high mountain biota are often related to specific topographic features such as warm and/or dry valleys.

Despite the importance of biogeographic breaks for many species, some are distributed across both sides of these boundaries. Still, phylogeographic breaks within such species are expected to align with biogeographic breaks (the biogeography and phylogeography concordance hypothesis: Brante et al. 2012) because of shared ecological or evolutionary factors operating at both the intraspecific and interspecific level (Avise et al. 1987). Numerous studies in both marine and terrestrial realms have demonstrated that intraspecific boundaries (phylogeographic breaks) often correspond with interspecific boundaries (biogeographic breaks), supporting the concordance between biogeographic and phylogeographic break zones (Avise 1992; Avise et al. 1987; Bowen et al. 2016; Brante et al. 2012; Burton 1998; Dawson 2001; Ewers‐Saucedo et al. 2016; Ma et al. 2021; Novaes et al. 2013). A corollary of this hypothesis is phylogeographic concordance among species, that is, that patterns of genetic variation are concordant among species (Kelly and Palumbi 2010; Burton 1998), as observed for species similarly affected by Pleistocene climate fluctuations (Kirschner et al. 2022). Since biogeographic breaks are identified using entire biota, they do not account for taxon‐specific differences, leading to some discordance between phylogeographic and biogeographic breaks. Such incongruences may result, for instance, from the insufficient power of genetic drift in species with large population sizes, such as in many marine species (Crandall et al. 2019), or from differences in ecological traits, in particular concerning dispersal capability (Irwin 2002; Papadopoulou and Knowles 2016).

In mountain ranges, the *proportion* of endemism increases with elevation (Noroozi et al. 2018; Steinbauer et al. 2016), with endemic richness, that is, the number of endemic species, often peaking at mid elevations (e.g., Trigas et al. 2013). Consequently, biogeographic structure in high‐mountain ranges is expected to be largely determined by endemic, often range‐restricted species. Indeed, this has been observed in southwestern Asian mountain ranges, where biogeographic units derived solely from endemic high‐mountain taxa (areas of endemism) closely align with those derived from the entire high‐mountain flora (bioregions; Noroozi et al. 2021). Thus, if biogeographic units and their boundaries are largely determined by high‐elevation endemics, it can be expected that phylogeographic boundaries of more widespread high‐elevation (alpine) species may be more strongly correlated with biogeographic boundaries than is the case in mid‐elevation (montane) species, for which lower‐elevation areas, such as major valleys, are less severe barriers than they are for high‐elevation species. To our knowledge, this hypothesis has not been tested yet.

Here, we investigate whether biogeographic and phylogeographic boundaries are congruent in mountain species, with a focus on endemics of the Iranian Plateau. Using RADseq data, we inferred the phylogeographic structure for nine such mountain plant species (four from mid‐elevations, five from high‐elevations), each being distributed across at least three major mountain ranges identified as areas of endemism (Noroozi et al. 2021, 2019, 2018). Specifically, we want to address two key questions: (1) Are phylogeographic breaks congruent with biogeographic breaks, as proposed by the biogeography and phylogeography concordance hypothesis? (2) Do high‐elevation species have a higher degree of concordance between phylogeographic and biogeographic breaks, reflecting the influence of high‐elevation endemics on areas of endemism and bioregions (Noroozi et al. 2021)? Although not the focus of this study, still with the available data it is possible (3) to assess and discuss species‐specific phylogeographic patterns of the investigated species.

## 2 Materials and Methods

### 2.1 Study Area and Biogeographic Units

**FIGURE 1:**
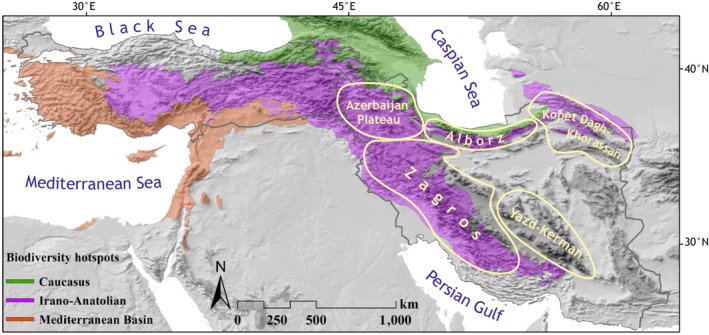
Topographic map of SW Asia and its global biodiversity hotspots, areas of endemism on the Iranian Plateau indicated by yellowish outlines.

The Iranian Plateau displays considerable topographic heterogeneity, shaped by a complex tectonic history (Stöcklin 1968, 1974). The elevation of the region ranges from 26 m below sea level up to 5671 m a.s.l. The region is at the confluence of three phytogeographical regions: the Euro‐Siberian in the north, the Saharo‐Sindian in the south and the Irano‐Turanian region covering the high mountain ranges. Most of the plant diversity of Iran is found in two global biodiversity hotspots, that is, the Irano‐Anatolian and the Caucasus hotspots (Figure 1), with over 75% of the c. 2600 endemic species confined to mountains, that is, elevations above 1400 m a.s.l. which cover ca. 50% of the Iranian surface area (Noroozi et al. 2019). Of those, several major mountain ranges (i.e., Alborz, Zagros, the mountains of northwestern Iran collectively referred to as Azerbaijan Plateau (Noroozi et al. 2019), Kopet‐Dagh Khorassan, Yazd‐Kerman mountains) have been identified as areas of endemism (Noroozi et al. 2019; Noroozi et al. 2018; Figure 1) and as bioregions considering the entire (sub)alpine flora (Noroozi et al. 2021). The areas of endemism are strongly associated with the topographic structure of the region and are confined to the high mountain ranges (Noroozi et al. 2019), but the exact environmental drivers have not been tested so far. As biogeographic histories of areas of endemism (i.e., areas of non‐random distributional congruence among taxa: Morrone 1994) are expected to have been impacted by common geological, ecological and/or evolutionary processes (Harold and Mooi 1994; Morrone 1994), boundaries between areas of endemism, especially when non‐nesting as in the study area, can be treated as biogeographic breaks. Accordingly, an association between biogeographic breaks (identified via areas of endemism from the entire flora or a large subset thereof) and phylogeographic breaks (identified in species occurring in multiple areas of endemism) can be tested using available data on areas of endemism in the study region.

### 2.2 Selected Species and Sampling

We selected nine mountain species to achieve a representative sampling both across elevational belts (four of them found in the mid‐elevation montane zone and five growing in the alpine zone: Table 1) and previously identified areas of endemism (each species is distributed in at least three major mountain ranges and thus areas of endemism of the study region) as well as to cover phylogenetically divergent lineages (jointly five plant families: Table 1). According to the Chromosome Counts Database (Rice et al. 2015), the investigated species are diploid (no data available for *Helichrysum oligocephalum* and *Crepis heterotricha*). Dedicated studies on pollination and dispersal biology of the studied species are lacking, but general morphology of reproductive structures suggests that none of the species is wind‐pollinated, but rather animal‐pollinated and/or self‐pollinating, and that none of the species is animal‐dispersed, but rather anemochoric (cypselas with a hairy pappus in *Crepis* and *Helichrysum*, inflated closed fruits in *Didymophysa* and *Physoptychis*) or barochoric with unspecific diaspores (seeds in *Allium* and *Dielsiocharis*; mericarps in *Onosma* and *Phlomis*; cypselas without hairy pappus in *Tanacetum*). All these species are endemics of the Iranian Plateau, which allowed these to be sampled over their entire ranges. For each species, 8–15 localities were sampled with a minimum distance of 10 kms (Table S1). Fresh leaves of two to seven individuals (on average four) from each locality were collected and stored in silica gel until DNA extraction. Individuals were sampled at least five meters apart to avoid collecting clones.

**TABLE 1:** Elevational, geographical, and ecological distributions of the investigated species.

Species	Distribution	No. of sampled populations
Montane zone		
*Allium scabriscapum* (Amaryllidaceae)	Azerbaijan, Alborz, Zagros, Yazd‐Kerman	11
*Helichrysum oligocephalum* (Asteraceae)	Azerbaijan, Alborz, Zagros	13
*Onosma microcarpa* (Boraginaceae)	Azerbaijan, Alborz, Zagros	14
*Phlomis olivieri* (Lamiaceae)	Azerbaijan, Alborz, Zagros	14
Alpine zone		
*Crepis heterotricha* (Asteraceae)	Alborz, Zagros, Yazd‐Kerman	12
*Didymophysa aucheri* (Brassicaceae)	Azerbaijan, Alborz, Zagros	8
*Dielsiocharis kotschyi* (Brassicaceae)	Alborz, Kopet Dagh, Zagros, Yazd‐Kerman	15
*Physoptychis gnaphalodes* (Brassicaceae)	Azerbaijan, Alborz, Kopet Dagh, Zagros	15
*Tanacetum kotschyi* (Asteraceae)	Azerbaijan, Alborz, Zagros	11

### 2.3 Molecular Data Generation

DNA extraction was done using the Invisorb Spin Plant Mini Kit (Invitec Diagnostics, Berlin, Germany) following the manufacturer's protocol with one exception: following the plant matter lysis, 200 μL of chloroform:isoamyl alcohol (24:1) were added to the lysis solution, centrifuged for 5 min at 11,000 rpm, and subsequently the upper phase of the solution was pipetted to the pre‐filter. The extracted genomic material was cleaned with NucleoSpin gDNA Clean‐up (Macherey‐Nagel, Düren, Germany) and eluted in 100 μL ddH_2_O. A Qubit Fluorometer (Thermo Fisher Scientific, Vienna, Austria) was used to measure the DNA concentrations of all samples.

The RAD libraries were prepared following Paun et al. (2015) using 150 ng of DNA as starting material, digested using PstI restriction enzyme (New England BioLabs, Frankfurt/Main, Germany). Each multiplexed library included between 35 and 64 indexed samples. All sequencing was done at the Vienna Biocenter Core Facilities (https://ngs.vbcf.ac.at). The libraries were sequenced on Illumina HiSeq2000 v4 (for *Dielsiocharis kotschyi*, *Onosma microcarpa*, *Phlomis olivieri*) or Illumina NovaSeq (*Allium scabriscapum*, *Crepis heterotricha*, *Didymophysa aucheri*, *Helichrysum oligocephalum*, *Physoptychis gnaphalodes*, *Tanacetum kotschyi*) with single‐end 100 bp reads. To increase coverage in species with likely large genomes, *A. scabriscapum* was sequenced a second time (for genome sizes in *Allium* see, for example, Ohri et al. 1998) on Illumina NextSeq platform with single‐end 150 bp reads; two samples of *A. scabriscapum* were added twice to each library and their results were merged.

*BamIndexDecoder* as part of *illumina2bam 1.03* (https://github.com/gq1/illumina2bam) was used for demultiplexing of the reads based on index reads using default settings, followed by *process_radtags* from *Stacks2 2.41* (Rochette et al. 2019) for demultiplexing based on inline barcodes and quality filtering. Default settings were used for *process_radtags*, except for setting the sliding window length to 0.06 instead of the default 0.15 and setting the score limit to 20 instead of 10 for stricter quality filtering. For the *Allium* library that has been sequenced as 150 bp reads, the data were trimmed to the same size as the rest (94 bp after removal of the 6 bp long inline barcode) using *Trimmomatic 0.39* (Bolger et al. 2014). Because some samples had extremely high numbers of reads (the highest read count was 41.4 million reads in one sample of *C. heterotricha* ), causing extremely long assembly times, all samples in all species were subsampled to contain at most 10 million reads, affecting 56 samples across the species *A. scabriscapum*, *C. heterotricha* , *D. aucheri, H. oligocephalum*, *P. gnaphalodes* and *T. kotschyi*.

We followed Paris et al. (2017) to find the optimal assembly parameters for the *denovo_map.pl* script from *Stacks2* and explored different values of the parameters *M*, the allowed differences between alleles within samples, and *n*, the allowed differences between loci across samples. The parameter optimization was done on a subsampled dataset with only one sample from each population, selecting the one with the highest read count. Due to computational constraints, we limited our exploration of the parameter space to the diagonal values (*n* = *M* = 1, *n* = *M* = 2, etc. up to a value of 7), as equal *n* and *M* values are expected to provide a good balance between intra‐individual and inter‐individual merging. We selected the assembly parameters that resulted in the most ‘*r80* loci’ (locus present in 80% of samples). In *A. scabriscapum*, *C. heterotricha* , *D. aucheri*, *D. kotschyi* and *P. olivieri* , the *r80* loci number did not peak. In those cases, the choice of optimal parameter values was instead based on when the distribution of SNPs per locus reached a plateau.

Each species was then de‐novo assembled using all samples and default settings other than the *M* and *n* parameters, which were set to their optimal values. In the case of *A. scabriscapum*, the number of assembled loci became too large to handle computationally when all samples were included, and it was necessary to first construct the catalogue of loci during the *cstacks* step of the *denovo_map.pl* pipeline using a subset of samples (the same subset of samples used for the optimization), before mapping all samples to the catalogued loci during the *sstacks* step.

For generating the final SNP data set, the following filtering steps were applied using *populations* as part of *Stacks2* in three steps: First, the loci with more than 50% missing data across all samples were removed. Second, potentially paralogous loci were removed by excluding loci that were heterozygous for more than 50% of the samples and only retaining loci with a number of SNPs below a certain threshold set by cumulatively increasing the number of allowed SNPs per locus until at least 90% of all loci were included (the maximum number of SNPs per locus thus reached was 11: see Section 2.4); this ensures that the top 10% SNP rich loci, which most likely contain paralogous loci, are excluded. A custom script was used to extract the loci below the set threshold and to include them in the whitelist used during the SNP filtering. Third, singletons were removed and only one random SNP per locus was retained. Final SNP data sets are available in NEXUS file format and in STRUCTURE file format as Appendices S1 and S2, respectively.

### 2.4 Data Analyses

Although our data set likely includes loci that may be under divergent selection, their inclusion is not expected to affect the inference of genetic structure (e.g., Gaither et al. 2015), hence the subsequent analyses were done with the complete SNP data set. The genetic structure was inferred using geographically constrained non‐negative matrix factorization as implemented in *TESS3* (Caye et al. 2016) using the *R* package *tess3r 1.1.0* after file format conversion to GENO using *LEA 2.6.0* (Frichot and François 2015). *TESS3* was run for 10 replications each for *K* = 1–10 using default settings. Additionally, to reconstruct the reticulate relationships between the populations of each taxon, Neighbour‐Net analyses were run using *Splitstree4 4.16* (Huson and Bryant 2005) and distances calculated with the Hasegawa‐Kishino‐Yano substitution model (Hasegawa et al. 1985) and empirical base frequencies. Finally, for each species an ordination was conducted by calculating principal components (PC) using the PCA (Principal component analysis) function in the *R* (3.6.3) package *adegenet 2.1.3* (Jombart 2008), preceded by conversion to STRUCTURE file format using *PGDspider 2.1.1.5* (Lischer and Excoffier 2011). Eventually, the number of genetic groups (phylogroups) was determined taking the following considerations into account: (i) the *K*‐value, where the cross‐validation score, that is, the root mean‐squared errors computed on a 95% subset of loci, calculated in *TESS3* starts to plateau or rise again (Frichot and François 2015); (ii) phylogroups are clearly discernible in the Neighbour‐Net and the PCA and (iii) phylogroups are biologically reasonable (e.g., phylogroups only present in strongly admixed populations are biologically non‐interpretable).

In order to identify the main phylogeographic breaks (i.e., major intraspecific genetic boundaries), the Monmonier's maximum difference algorithm (Monmonier 1973) was employed (Manni et al. 2004) using the monmonier function of the R package *adegenet* (Jombart 2008; Jombart et al. 2014). Briefly, the algorithm starts from the highest distance (in our case genetic distance) between neighbouring points (in our case populations) in a network and iteratively moves to the next‐highest distance until a predefined threshold is reached. For our analysis, the threshold distance was set to the default value used by the monmonier function, which corresponds to the third quartile of all distances between neighbouring points in the constructed network. To apply Monmonier's algorithm to a network of populations rather than individuals, genotypes of individuals within a population were summarized into allele counts per population (i.e., allele frequencies) using the genind2genpop function from adegenet. The sensitivity of boundary identification to different settings was assessed by combining two methods for calculating population genetic distances with two methods for constructing connection networks (i.e., the population network), resulting in four analyses per species. Specifically, for population genetic distances, we used Nei's genetic distance (Nei 1972, 1978), transformed into an Euclidian distance using the cailliez function in the R package *ade4* (Dray and Dufour 2007), and Roger's genetic distance (Rogers 1972) both calculated using the dist.genpop function in *adegenet*. For constructing population connection networks, we used Delaunay triangulation, which connects points by forming triangles such that no point lies inside the circumcircle of any triangle, and the Gabriel graph, which connects two points only if no other point lies within the diameter of the circle defined by the two points. This methodological approach allowed systematically evaluating the impact of different genetic distance metrics and network types on the detection of phylogeographic boundaries.

## 3 Results

### 3.1 Molecular Data Generation

After demultiplexing and quality filtering, the average read count for samples of each species ranged from 1,269,533 to 7,863,239 reads, with the minimum number of reads for samples of each species ranging from 340,735 to 1,924,467 and the maximum ranging from 2,861,512 to 10,000,000 (Table 2). The optimal *n* and *M* parameters for the de novo assembly using stacks2 ranged from 1 to 5 across the species (Table 2). The maximum number of SNPs per locus for each species ranged from 5 to 11, and the number of SNPs retained in each species dataset ranged from 27,649 to 61,626 after all filtering was applied (Table 2).

**TABLE 2:** Characteristics of the RAD‐seq data.

Species	Read numbers		*n* = *M* ^a^	Max. SNPs per locus	SNPs retained	*K* ^b^
	Average	Range				
*Allium scabriscapum*	4,787,837	923,034–10^7^	5	9	61,626	3
*Helichrysum oligocephalum*	7,652,978	4,036,353–10^7^	1	7	51,946	1
*Onosma microcarpa*	1,618,169	401,908–5,259,744	5	11	37,991	3
*Phlomis olivieri*	1,492,997	291,444–6,057,631	4	10	27,649	5
*Crepis heterotricha*	6,624,716	2,504,805–10^7^	4	6	36,989	2
*Didymophysa aucheri*	7,863,239	1,924,467–10^7^	4	8	45,952	6
*Dielsiocharis kotschyi*	1,269,533	340,735–2,861,512	4	11	27,839	5
*Physoptychis gnaphalodes*	6,327,883	1,455,645–10^7^	3	9	48,642	4
*Tanacetum kotschyi*	7,590,953	1,742,790–10^7^	2	5	50,973	7

^a^ Assembly parameters (see text for details).

^b^ Number of gene pools (phylogroups) as inferred with *TESS3*.

### 3.2 Genetic Structure of the Species

**FIGURE 2:**
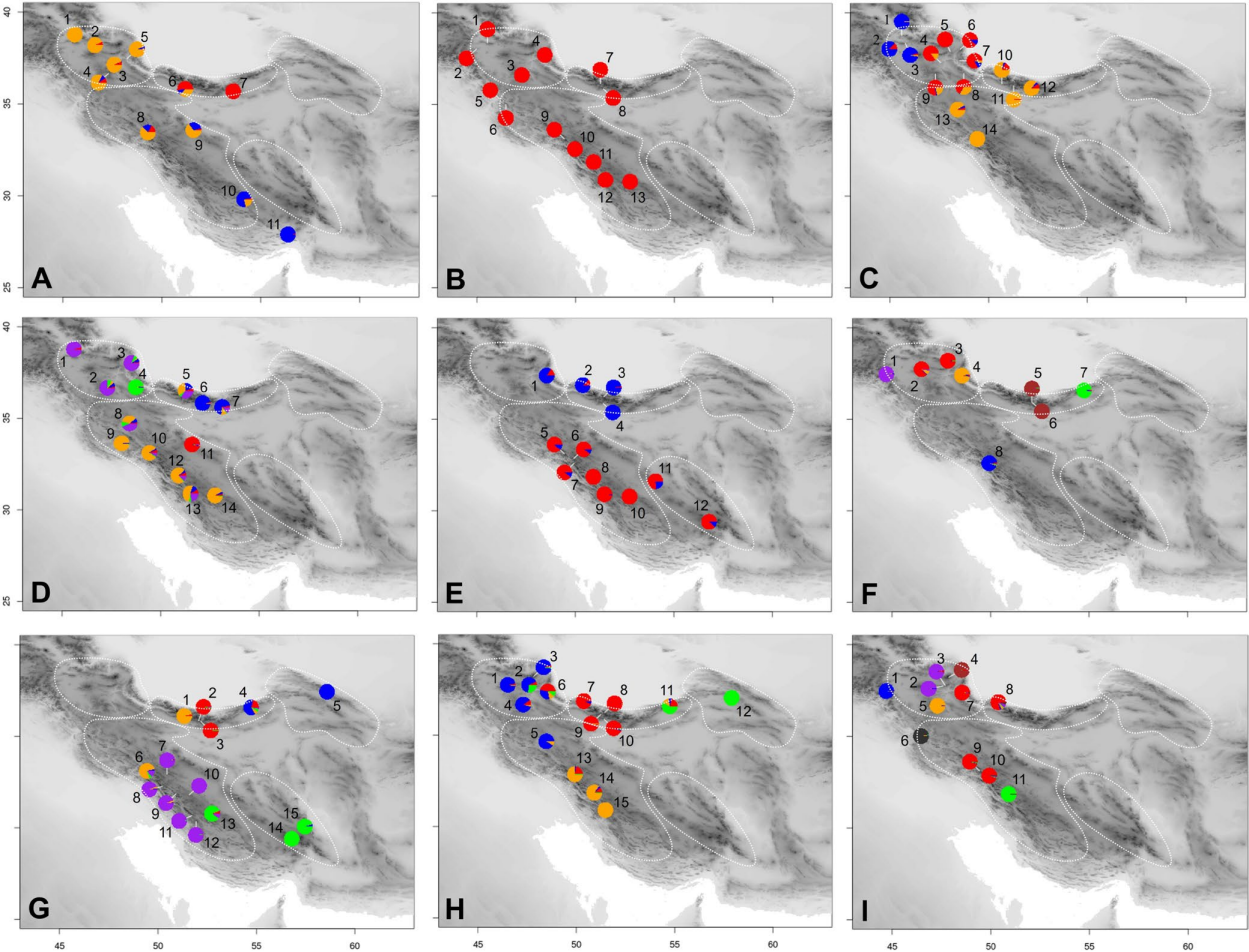
Phylogeographic structure of the investigated species (A–D: Montane species; E–I: Alpine species) inferred using *TESS3*: (A) *Allium scabriscapum*, (B) *Helichrysum oligocephalum*, (C) *Onosma microcarpa*, (D) *Phlomis olivieri*, (E) *Crepis heterotricha*, (F) *Didymophysa aucheri*, (G) *Dielsiocharis kotschyi*, (H) *Physoptychis gnaphalodes* and (I) *Tanacetum kotschyi*. The different areas of endemism in the region are indicated as dotted outlines.

The genetic structure inferred by *TESS3* (Figure 2, Figure S1), networks (Neighbour‐Net; Figure S2), and ordination (Figure S3) is congruent. Only in *P. oliveri* and *D. kotschyi* the number of phylogroups suggested by *TESS3* (two each: Figure S1) appears to be too small when compared with Neighbour‐Nets (Figure S2) and ordinations (Figure S3), which most likely is due to strong hierarchical population structure; here, the number of phylogroups most consistent among networks and ordinations was chosen. The number of phylogroups ranged from two to seven in the analysed species (Figure 2) except in *H. oligocephalum* where only a single phylogroup was found (Figure 2B). The number of phylogroups in the four montane species (*A. scabriscapum* and *O. microcarpa* with three phylogroups each; *P. oliveri* with five phylogroups; *H. oligocephalum* with a single phylogroup) was on average three (3.7 if excluding *H. oligocephalum*), whereas that in the five alpine species (*T. kotschyi* with seven phylogroups; *D. aucheri* with six phylogroups; *D. kotschyi* with five phylogroups; *P. gnaphalodes* with four phylogroups; *C. heterotricha* with two phylogroups) was on average 4.8.

In the following, only species having more than one phylogroup (i.e., all but *H. oligocephalum*) are considered. In most species, phylogroups, whose precise delimitation was occasionally hampered by broader zones of admixed populations separating phylogroups (e.g., in *A. scabriscapum*), were longitudinally arranged along the main mountain axes (Azerbaijan Plateau—Alborz—Kopet Dagh‐Khorassan; Azerbaijan Plateau—Zagros—Yazd‐Kerman). Deviations from this arrangement along major mountain axes were found in *O. microcarpa* , *D. kotschyi* and *T. kotschyi* (Figure 2C,G,I), where populations from (north)western Zagros and western (to central) Alborz belonged to the same phylogroup. Correspondence of phylogroups with areas of endemism was only moderate, because areas of endemism (indicated in Figure 2) often contained more than one phylogroup (e.g., *O. microcarpa* in the Azerbaijan Plateau: Figure 2C; *D. aucheri* in the Alborz range: Figure 2F; *P. oliveri* in Zagros: Figure 2D) or shared phylogroups (e.g., Zagros and Yazd‐Kerman in *D. kotschyi*: Figure 2G; Alborz and Kopet Dagh‐Khorassan in *P. gnaphalodes*: Figure 2H; Alborz and Zagros in *D. kotschyi* and *T. kotschyi*: Figure 2F,I).

In detail, phylogeographic structure in the studied species differed considerably. In *A. scabriscapum* (Figure 2A), one phylogroup dominated, the other two being restricted to the eastern periphery of the distribution range (one in eastern Alborz, one in southeastern Zagros), connected to the main phylogroup by geographically intermediate genetically admixed populations. In contrast, in *O. microcarpa* (Figure 2C), the genetic differentiation, although also longitudinal, was more even, with a sharply delimited phylogroup (hardly any admixture) in the western Azerbaijan Plateau, another one in the eastern Azerbaijan Plateau and, connected to it via admixed populations, a phylogroup in western Zagros and Alborz. An even stronger differentiation was found in *P. olivieri* , where in addition to phylogroups from the main mountain ranges (Azerbaijan Plateau, Alborz, Zagros), connected via admixed populations, two additional essentially non‐admixed phylogroups (easternmost Azerbaijan Plateau; northeastern Zagros) were found (Figure 2D). Among the alpine species, *C. heterotricha* had the lowest number of phylogroups, but these were clearly separated latitudinally into a northern (Azerbaijan Plateau and Alborz) and a southern group (Zagros and Yazd‐Kerman; Figure 2E). The remaining four species had strong phylogeographic structure with essentially no admixed populations (*D. aucheri* and *T. kotschyi*: Figure 2F,I) or with a few admixed populations, especially in eastern Alborz and the Azerbaijan Plateau (*D. kotschyi* and *P. gnaphalodes*: Figure 2G,H).

### 3.3 Break Zones

Using different genetic distances (Nei's distance and Roger's distance, respectively) had minimal impact on the identified boundaries, with some differences concerning either the number of boundaries (the smaller set always being a subset of the larger set; e.g., *C. heterotricha* ) and/or their order (e.g., in *O. microcarpa* ; Figure S4). Employing different population networks (Delaunay triangulation and Gabriel graphs, respectively) led to expected differences in network paths, as a Gabriel graph includes only a subset of the connections present in the Delaunay triangulation (Figure S4). Nevertheless, there was still considerable overlap in the set of edges identified by both methods (e.g., in *D. kotschyi* all boundaries identified on the Gabriel graph were also found with Delaunay triangulation: Figure S4). In case of differences, the identified edges were geographically proximate (e.g., in *O. microcarpa* edges separating different populations in the Azerbaijan Plateau and Zagros; Figure S4), allowing general break zones to be identified.

**FIGURE 3:**
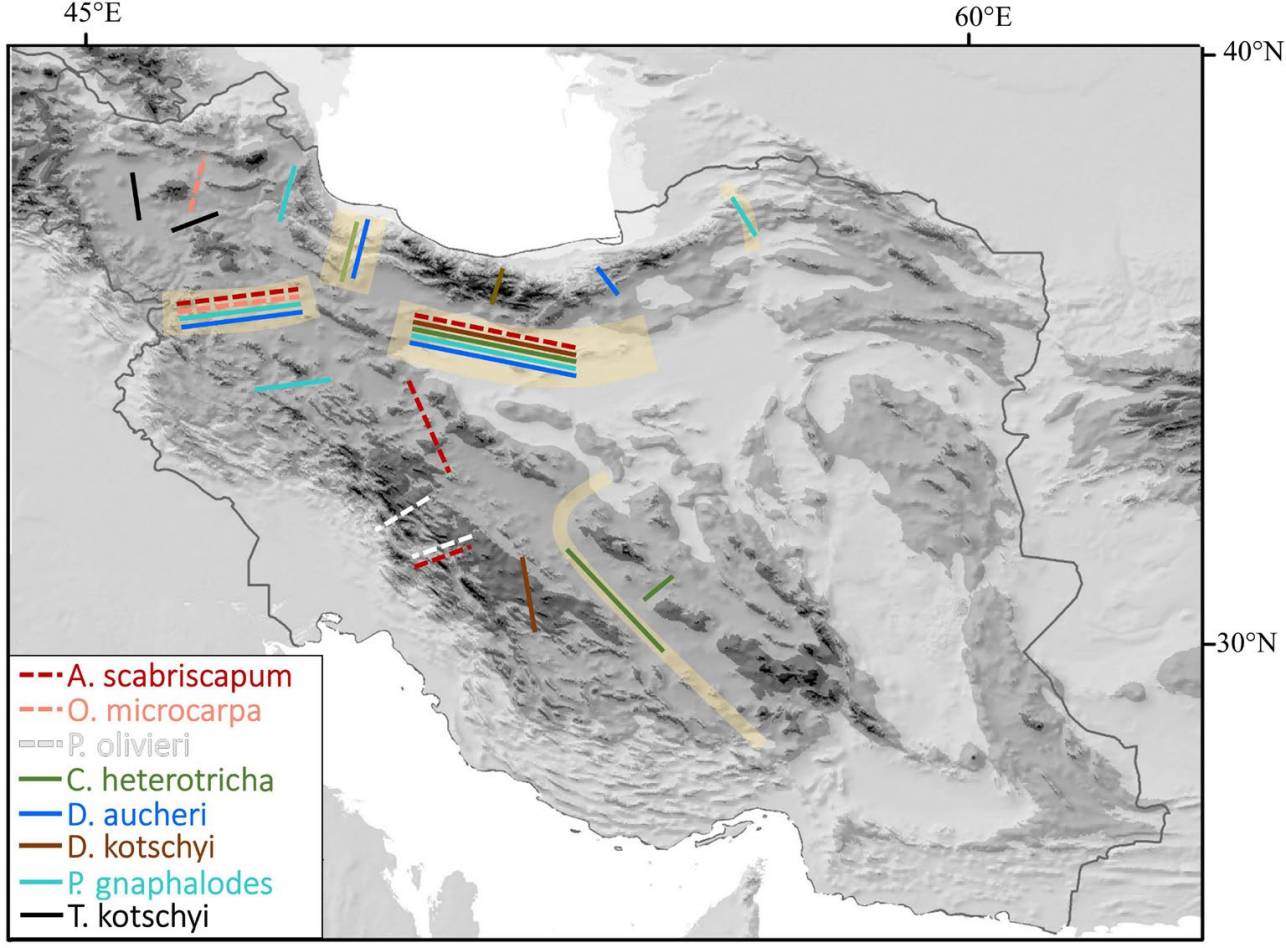
Break zones in the mountain flora of the Iranian Plateau. Biogeographic boundaries are indicated by pale yellow lines, their thickness being proportional to the number of species crossing this boundary; phylogeographic breaks of the eight species showing phylogeographic structure (indicated by different colours) are shown by thin lines (dashed lines for montane species and solid lines for alpine species).

In Figure S4, the path through the strongest genetic distances between neighbours (i.e., the putative genetic boundaries) is shown by arrows connecting the *middle points* of the edges between populations, the actual genetic boundary may, however, lie anywhere along these edges. Therefore, genetic boundaries (break zones) were placed taking also plausible barriers, such as topographic ones (e.g., low‐elevation areas), into account. Additionally, if the path shown in Figure S4 went through spatially close edges crossing the same putative barrier, these were considered to belong to the same genetic boundary (break zone). Furthermore, in Figure 3 only those genetic boundaries (break zones) identified in a species at least once in each type of population network (Delaunay triangulation and Gabriel graph), irrespective of whether this is based on Nei's or Roger's distance, are shown. An example is illustrated for *A. scabriscapum*, where one edge (in the Delaunay graph) and three edges (in the Gabriel graph; these edges are indicated in orange in Figure S4) connecting populations in Alborz (in the north) and Zagros (in the south) are interpreted to represent the same single break zone (shown in Figure 3) corresponding to the lowland area between the two mountain ranges. Major break zones (genetic boundaries) were identified between Alborz and Zagros (along the Central Desert of Iran; supported by five out of eight species: one montane and four alpine species) and between the Azerbaijan Plateau and Zagros (along the lowlands of Kurdistan; supported by four out of six species: two montane and two alpine species), both corresponding with biogeographic boundaries. Among the remaining break zones, only two were supported by two species each. However, only the break between the Azerbaijan Plateau and Alborz (two alpine species) corresponded to a biogeographic boundary (crossed by seven species), whereas the other, located within Central Zagros (two montane species), did not. The biogeographic boundaries between Alborz and Kopet Dagh‐Khorassan and between Zagros and Yazd‐Kerman were supported by break zones in single species (both alpine species), but only two species crossing these boundaries in each case. The remaining minor break zones, supported by single species only (either montane or alpine), were located in the Azerbaijan Plateau, Alborz, Zagros and Yazd‐Kerman, often corresponding to lower elevation areas separating high elevation ranges (e.g., four breaks in Azerbaijan Plateau, one between Central and Eastern Alborz, one in Northern Zagros, and one in Yazd‐Kerman).

## 4 Discussion

### 4.1 Biogeographic and Phylogeographic Breaks Are Concordant

Our study provides support for the congruency between biogeographic and phylogeographic patterns, that is, the biogeography and phylogeography concordance hypothesis (Bowen et al. 2016; Brante et al. 2012; Novaes et al. 2013). Specifically, of four phylogeographic break zones supported by at least two species, three are situated at the boundaries of areas of endemism or bioregions, which largely correspond to major mountain regions (Azerbaijan Plateau, Zagros, Alborz; Noroozi et al. 2019), or in transitional zones between areas of endemism (Talish Mts. between the Azerbaijan Plateau and Alborz, Mt. Shahvar [for location see population 11 of *P. gnaphalodes*: Figure 2H] between central Alborz and Kopet Dagh‐Khorassan) identified based on floristic data (Noroozi et al. 2019). Orographic barriers are among the most important determinants of biogeographic boundaries both for low and high elevation species (Ficetola et al. 2017), as is also the case in the study region, where the borders of areas of endemism and bioregions are the elevational discontinuities separating the mountain ranges from each other, for example, the Central Desert between Alborz and Zagros or the lowlands of Kurdistan between Azerbaijan Plateau and Zagros (Noroozi et al. 2021, 2019, 2018). In fact, bioregionalization of the region is mainly determined by high elevation species (Noroozi et al. 2021), and biogeographic boundaries are mainly supported by alpine species (Figure 3), even if ignoring those biogeographic boundaries that are not crossed by any of the sampled montane species (i.e., the one separating Alborz from Kopet Dagh‐Khorassan and the one separating Zagros from Yazd‐Kerman). In the European Alps, topographical barriers had a profound effect on the dispersal of alleles during post‐glacial migration and shaped the genetic structures of alpine species (Thiel‐Egenter et al. 2011), and this is likely also the case in our study area (Ahmadzadeh et al. 2013; Rajaei et al. 2013). Jointly, these results suggest that for mountain biota topographic barriers, such as deep valleys, likely often together with historical range shifts, are the main drivers of genetic divergences from the populational to the species level (Thiel‐Egenter et al. 2011).

In the study area, phylogeographic break zones deviate from biogeographic patterns in two ways. First, the biogeographic boundaries between Alborz and Kopet Dagh‐Khorassan and between Zagros and Yazd‐Kerman are not well supported by phylogeographic break zones (one species each). This is likely simply the result of the low number of sampled species that span these boundaries (two each). Investigating more species (including those distributed in only two mountain ranges such as Zagros and Yazd‐Kerman) can be expected to increase the support for these biogeographic boundaries.

Second, there is a considerable number of phylogeographic breaks that are not congruent with any biogeographic boundary, a pattern found also in other studies (e.g., Crandall et al. 2019; Kelly and Palumbi 2010). At least for the north‐western part of the study area (Azerbaijan Plateau, Alborz), a certain staggering of phylogeographic break zones may be expected from the biogeographically transitional character of this region (Noroozi et al. 2019). For instance, the Talish Mountains at the southwestern shores of the Caspian Sea are geographically and geologically (orogenetically) close to the Alborz Mountains, sharing many alpine species with the Alborz range (Noroozi et al. 2008). At the same time, however, they are geographically and, especially at their southern and western slopes, climatically close to the Azerbaijan Plateau, sharing many montane species with the Azerbaijan Plateau (Noroozi et al. 2018).

Phylogeographic breaks that are incongruent with biogeographic breaks usually concern only single species, making it unclear whether they reflect more general, yet subtle biogeographic structures or merely species‐specific idiosyncratic patterns. Some of the single breaks are clearly congruent with the topography and orogeny of the mountains (Figure 3): (1) the three breaks (two in *T. kotschyi* and one in *O. microcarpa* ) surrounding the volcanic and geographically isolated Sahand Mt. (3710 m a.s.l.) in the center of the Azerbaijan Plateau (for its location see population 2 of *T. kotschyi*: Figure 2I); (2) the break (in *P. gnaphalodes*) in northern Zagros separating the volcanic Alvand Mt. from the rest of Zagros (for its location see population 5 of *P. gnaphalodes*: Figure 2H), which agrees with the orographic and edaphic similarity as well as floristic connections, particularly in alpine habitats, between Alvand Mt. and mountains in the adjacent Azerbaijan Plateau and even Alborz (Bagheri et al. 2022); (3) the break (in *C. heterotricha* ) in Yazd‐Kerman between the Shirkuh Mts. in the northern part and the Kerman Massif in the southern part of the Yazd‐Kerman region. The high number of phylogeographic breaks in the Zagros range (in *D. kotschyi*, *A. scabriscapum*, *P. olivieri* : Figure 3) is likely due to both the high topographic heterogeneity and the climatic heterogeneity (both longitudinally and latitudinally). This mountain range stretches from north‐west to south‐central Iran, with scattered and isolated alpine zones throughout. The climate in the western parts is more humid than in the east, with corresponding differences in vegetation, that is, oak woodlands covering the western area and steppe shrublands dominating the eastern regions (Noroozi et al. 2020; Zohary 1973). Moreover, in Zagros there have been several refugia during the glacial periods (Ahmadzadeh et al. 2013; Rajaei et al. 2013) and, as suggested for other regions (Alvarez et al. 2009; Hewitt 2004; Schönswetter et al. 2005), historical range shifts and accompanying population contractions and expansions have shaped the current genetic structure of mountain species. Therefore, it seems likely that at least some of those minor phylogeographic breaks identified here reflect a broader pattern, but additional studies will be needed to test this.

### 4.2 Phylogeographic Patterns in Iranian Mountain Plants

Although the inferred number of phylogroups should be interpreted with caution, it appears that phylogeographic structure in montane species is on average lower (i.e., fewer phylogroups) than that of alpine species, even if ignoring the extreme case of *Helichrysum oligocephalum*, which shows no phylogeographic structure (Figure 2B). This may suggest that in mid‐elevation species the level of isolation is lower due to the higher degree of contiguity of the montane zone compared to the alpine zone. Yet, three mid‐elevation species show heterogeneous and strong phylogeographic patterns, which are likely the result of range shifts and phases of geographic isolation caused by Pleistocene climate oscillations (Ahmadzadeh et al. 2013; Rajaei et al. 2013). Specifically, for montane species, extended periods of isolation may have occurred during Pleistocene cold periods, when elevational zones descended for 1500–1600 m (Kuhle 2008), causing montane vegetation types to be more fragmented (similar to forest vegetation in Europe: Bennett et al. 1991; Leroy and Arpe 2007). Such a hypothesis of glacial range contraction remains, however, to be tested.

In contrast to montane species, alpine species are expected to experience stronger isolation during warmer periods (Pleistocene interglacials and the Holocene), when they are forced into higher elevations (Rahbek et al. 2019), but also this hypothesis of inter‐ and postglacial contraction remains to be tested. The degree of isolation and thus of phylogeographic structure may also depend on the species' habitat requirements. Whereas *C. heterotricha* , a species of often contiguous thorn‐cushion vegetation (Noroozi et al. 2010), contains only two latitudinally separated phylogroups (Figure 2E), two species of alpine rocky habitats, *D. kotschyi* and *T. kotschyi*, comprise several phylogroups. Their stronger phylogeographic structure may be due to the usually scattered and geographically isolated distribution of rocky habitats, fostering allopatric divergence. Similarly, *D. aucheri*, a species restricted to subnival screes of the study, and *P. gnaphalodes*, a characteristic species of high alpine and subnival scree vegetation types area (Noroozi et al. 2011, 2014), show strong phylogeographic structure. This is likely the result of limited gene flow between the populations on their ecological ‘sky islands’ (He and Jiang 2014; Warshaltl 2007), which is commonly found in subnival species, for instance, in the Himalaya (Luo et al. 2017, 2018, 2016).

## 5 Conclusion

This study supports the hypothesis that biogeographic break zones are mostly congruent with phylogeographic break zones, suggesting that both are affected similarly by orographic boundaries. This underlines, at least in the study region, the validity of areas of endemism as a basis for nature conservation planning, as areas of endemism to some extent also reflect intraspecific diversity. As expected, genetic patterns of alpine species are, on average, more structured than those of montane species. One likely relevant factor is the stronger isolation of high‐elevation habitats acting as sky islands, but more studies will be necessary to assess the role of Pleistocene climate oscillations in combination with differences in biological features (with respect to, for instance, pollination, dispersal, or habitat characteristics beyond elevational position) in shaping patterns of taxa in the high mountains of South West Asia.

## Author Contributions

**Jalil Noroozi:** conceptualization (lead), data curation (lead), funding acquisition (lead), project administration (lead), writing – original draft (lead). **Dennis Larsson:** analyses (lead), software (lead), visualization (lead), review and editing (equal). **Amir Talebi:** data curation (partly), review and editing (equal). **Sorour Rahmanian:** analyses (partly), software (partly), visualization (partly), review and editing (equal). **Moslem Doostmohammadi:** data curation (partly), review and editing (equal). **Tahereh Setayesh:** lab‐work (partly), review and editing (equal). **Dominik Metschina:** lab‐work (partly), review and editing (equal); **Ovidiu Paun:** methodology (equal), review and editing (equal). **Gerald M. Schneeweiss:** methodology (lead), supervision (lead), writing – review and editing (active).

## Funding

This study was supported by Austrian Science Fund (FWF P31898 and FWF PAT7717923).

## Conflicts of Interest

The authors declare no conflicts of interest.

## Supporting information


**Appendix S1:** mec70355‐sup‐0001‐AppendixS1.zip.

**Appendix S2:** mec70355‐sup‐0002‐AppendixS2.zip.

**Figure S1:** Results of *TESS3* analyses. For each species, the following information is shown: plot of the cross‐validation score across different *K* values (1–10); plot of the difference in mean cross validation error across different *K* values (2–10); histogram of ancestry coefficients for different *K* values (2–10); diffusion map of the genetic groups for different *K* values (2–10).
**Figure S2:** Neighbour‐nets based on HKY distances (see main text for details). Colors of population numbers correspond to those used for genetic groups identified by TESS3 (see Figure 2; strongly admixed populations shown in grey). Species are arranged according to elevational zone (montane species, alpine species) and, within each zone, alphabetically.
**Figure S3:** Principal component analysis (PCA). Colors of population numbers and of polygons delimiting genetic groups correspond to those used for genetic groups identified by TESS3 (see Figure 2; strongly admixed populations shown in grey). Species are arranged according to elevational zone (montane species, alpine species) and, within each zone, alphabetically.
**Figure S4:** Phylogeographic barriers identified using Monmonier's maximum difference algorithm on population networks (left column: Delaunay triangulation; right column: Gabriel graph), where population distances are calculated from Nei distances (upper row) or Roger's distance (lower row; see main text for details). Arrows indicate the directionality of the path (from larger to smaller distances), different colors (blue, red) indicating distinct paths with the same starting point. Edges in orange (in *Allium scabriscapum* only) indicate a set of edges jointly corresponding to a single genetic boundary geographically placed at the lowlands between Alborz (north) and Zagros Mountains (south). Species are arranged according to elevational zone (montane species, alpine species) and, within each zone, alphabetically.
**Table S1:** List of populations of all species, their collected locations, dates, altitudes, coordinates and other relevant information.
**Appendix S1:** RADseq‐derived SNP data sets of the nine investigated plant species in NEXUS file format.
**Appendix S2:** RADseq‐derived SNP data sets of the nine investigated plant species in STRUCTURE file format.

## Data Availability

Raw sequence reads are available in the Short Read Archive under BioProject PRJNA1357660. SNP data sets in NEXUS and in STRUCTURE file format are available in Appendices S1 and S2, respectively. Other data are included in Figures S1–S4 and Table S1.
