# Supplementary material for: Phylogeographic Patterns Are Strongly Associated With Biogeographic Patterns in the Irano‐Anatolian Global Biodiversity Hotspot
Source: Mol Ecol. 2026 Apr 29;35:e70355. doi: 10.1111/mec.70355 (PMC13126619; doi:10.1111/mec.70355)

Fig. S1. Results of *TESS3* analyses. For each species, the follwoing information is shown: plot of the cross-validation score across different *K* values (1-10); plot of the difference in mean cross validation error across different K values (2-10); histogram of ancestry coefficients for different *K* values (2-10); diffusion map of the genetic groups for different *K* values (2-10).

p. 2-11 *Allium scabriscapum*
p. 12-21 *Helichrysum oligocephalum*
p. 22-31 *Onosma microcarpa*
p. 32-41 *Phlomis oliveri*
p. 42-51 *Crepis heteotricha*
p. 52-61 *Didymophysa aucheri*
p. 62-71 *Dielsiocharis kotschyi*
p. 72-81 *Physoptachys gnaphalodes*
p. 82-91 *Tanacetum kotschyi*

Allium scabriscapum

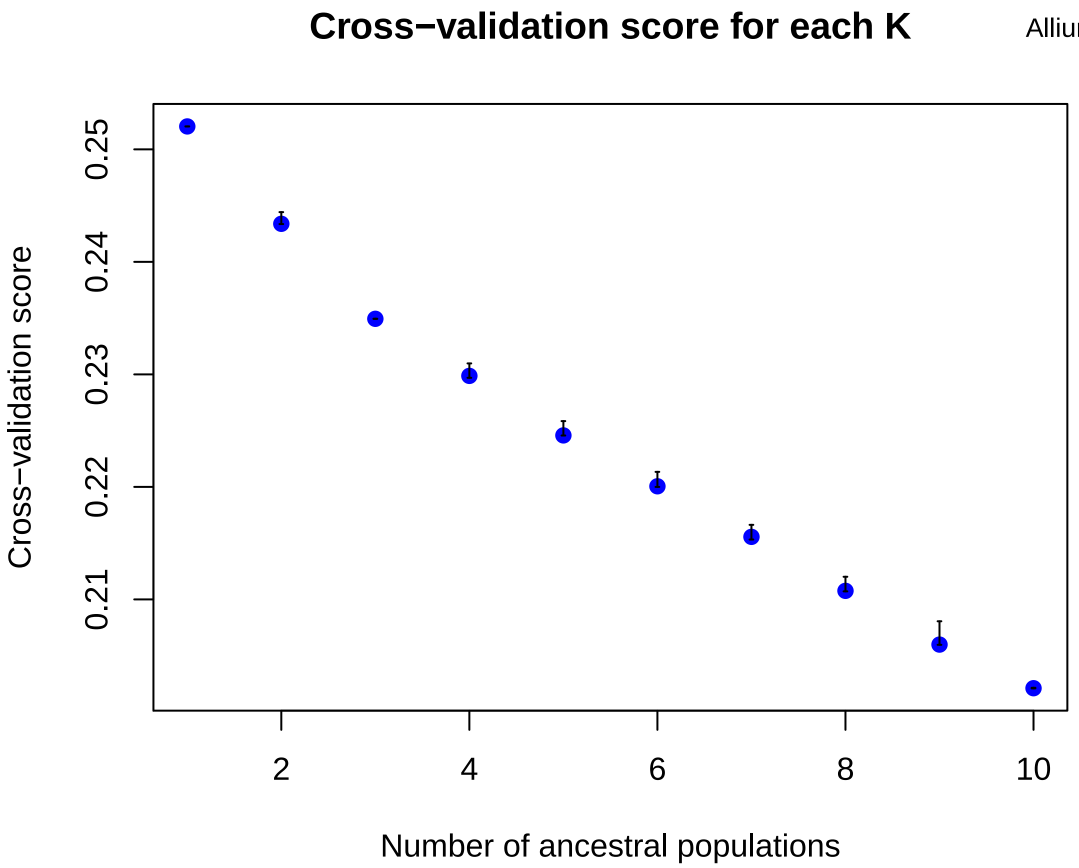

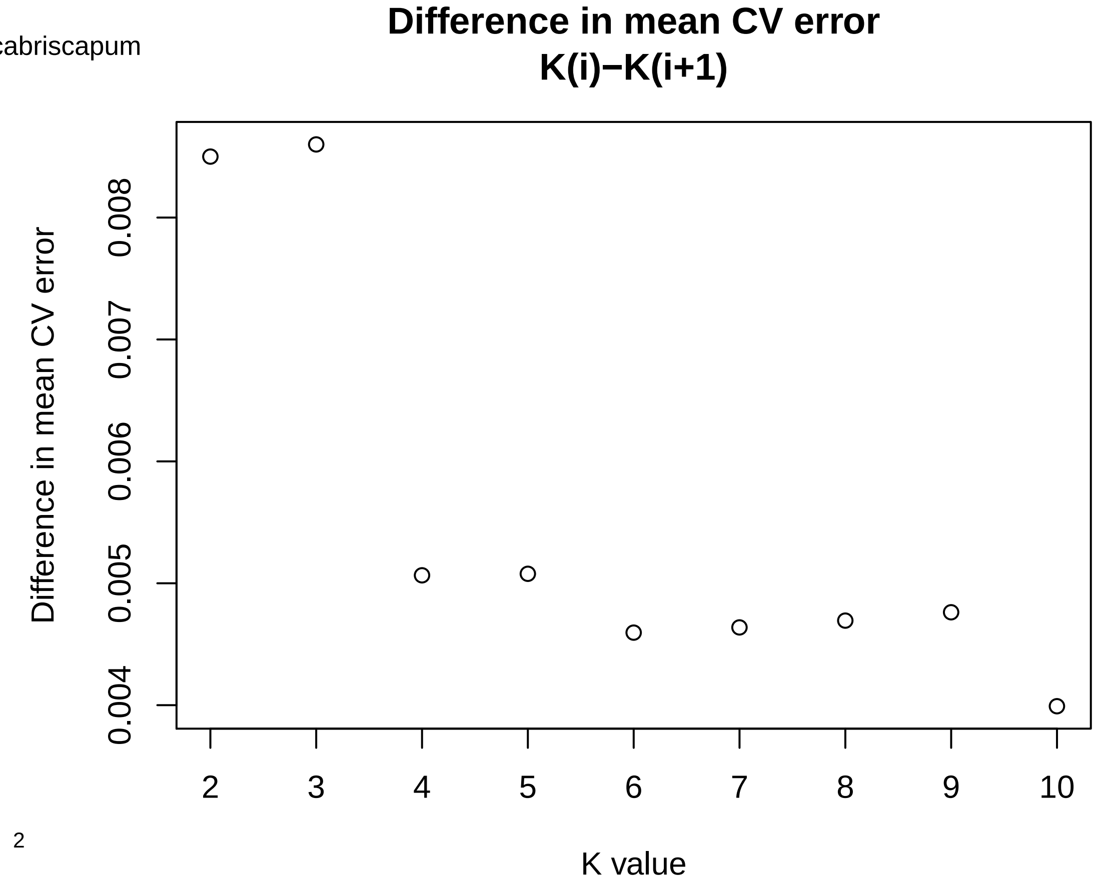

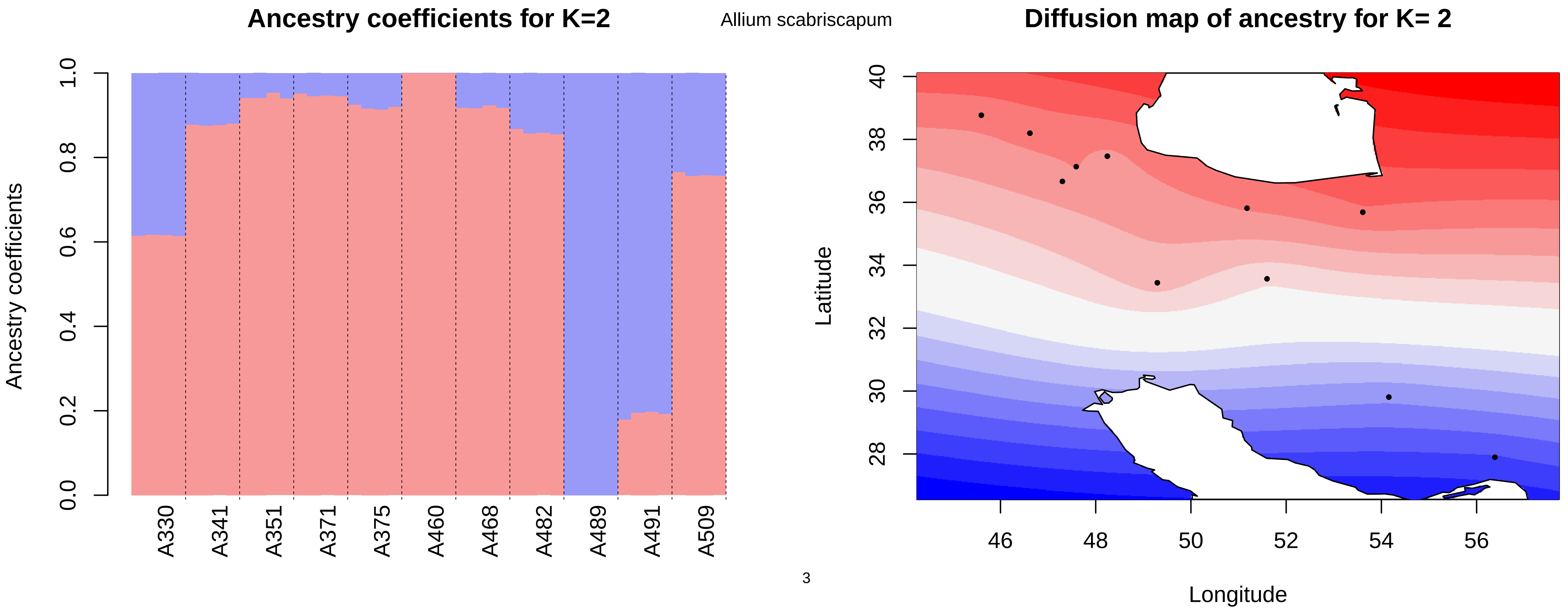

Ancestry coefficients for K=2
Allium scabriscapum
Diffusion map of ancestry for K= 2
Ancestry coefficients
0.0
0.2
0.4
0.6
0.8
1.0
A330
A341
A351
A371
A375
A460
A468
A482
A489
A491
A509
Latitude
40
38
36
34
32
30
28
46
48
50
52
54
56
Longitude

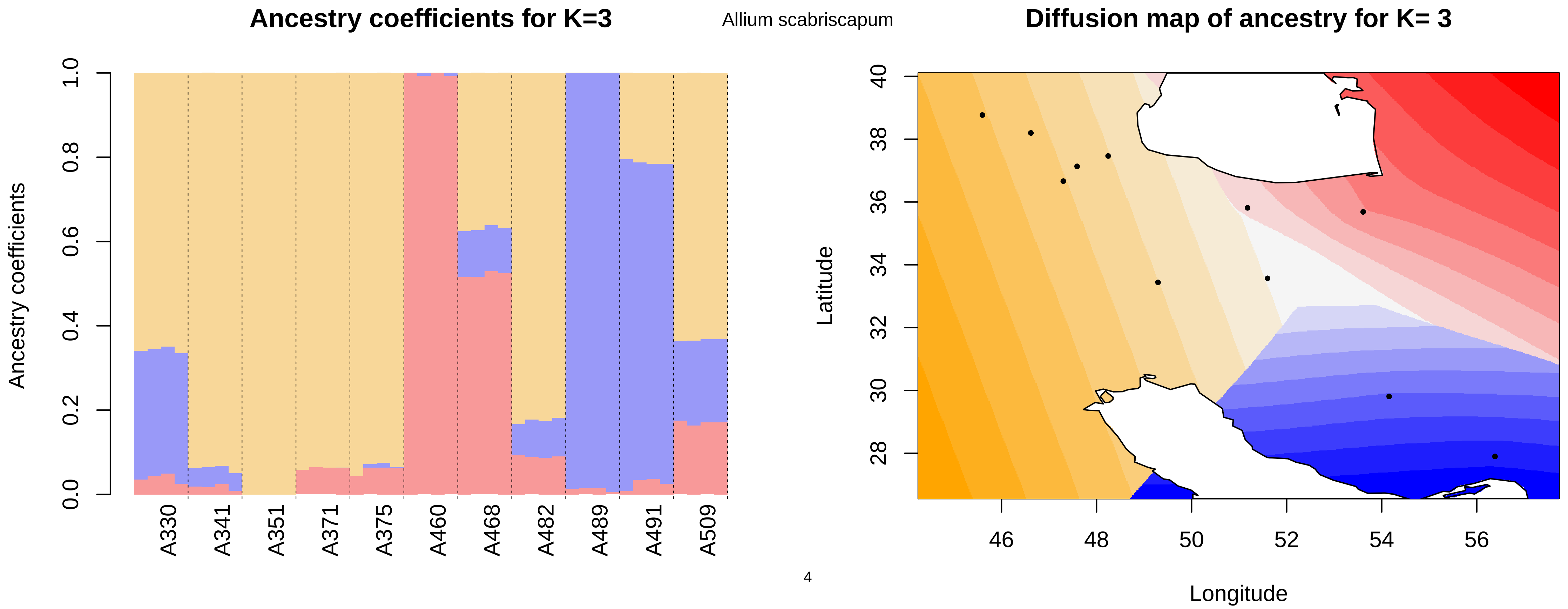
Ancestry coefficients for K=3
Allium scabriscapum
Diffusion map of ancestry for K= 3
Ancestry coefficients
0.0
0.2
0.4
0.6
0.8
1.0
A330
A341
A351
A371
A375
A460
A468
A482
A489
A491
A509
Latitude
28
30
32
34
36
38
40
46
48
50
52
54
56
Longitude

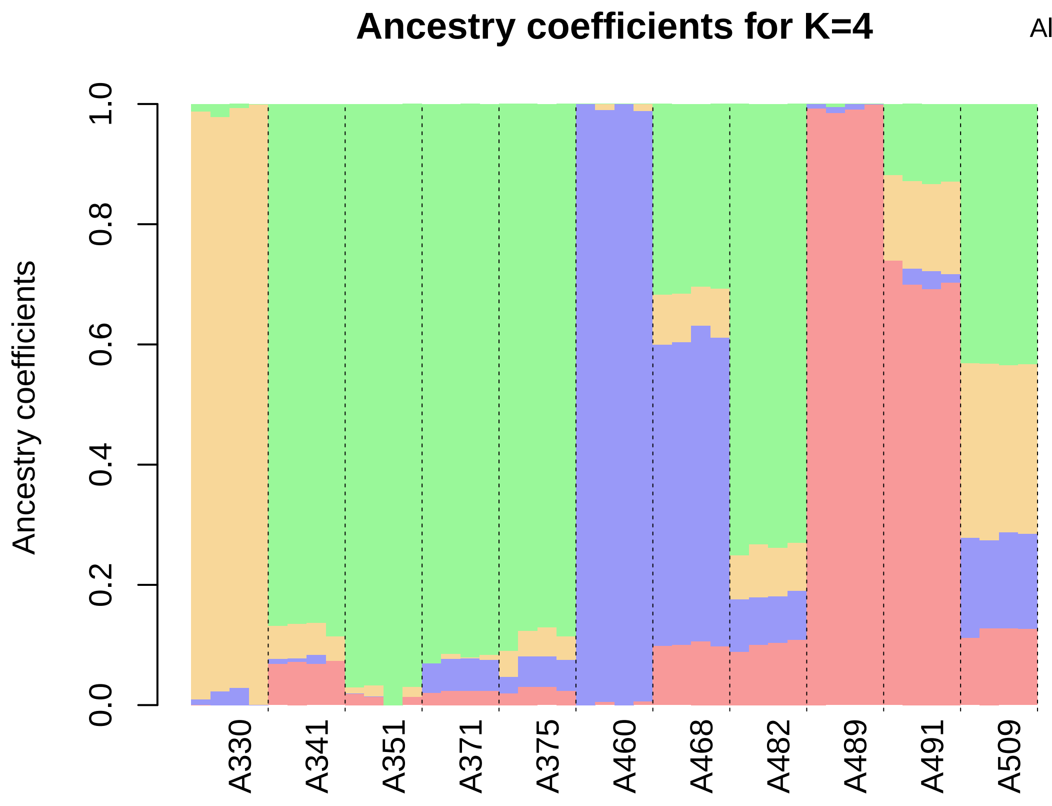

Allium scabriscapum

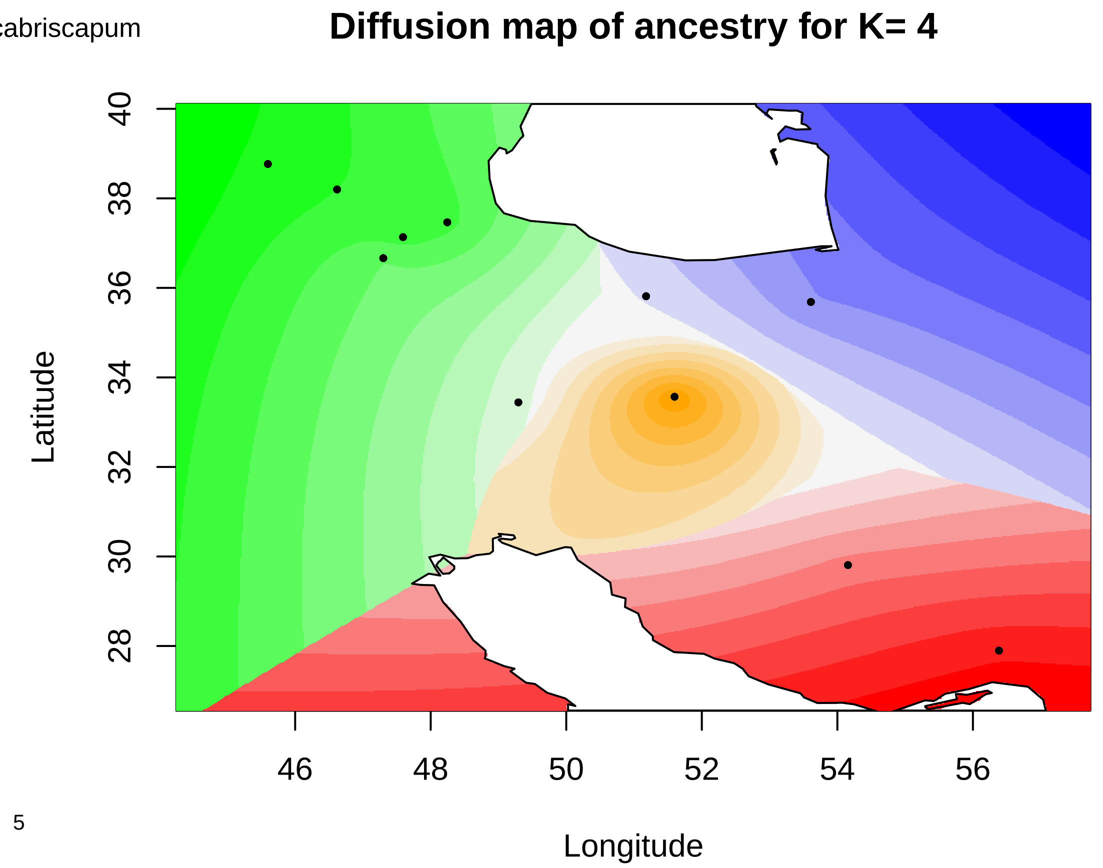

Allium scabriscapum

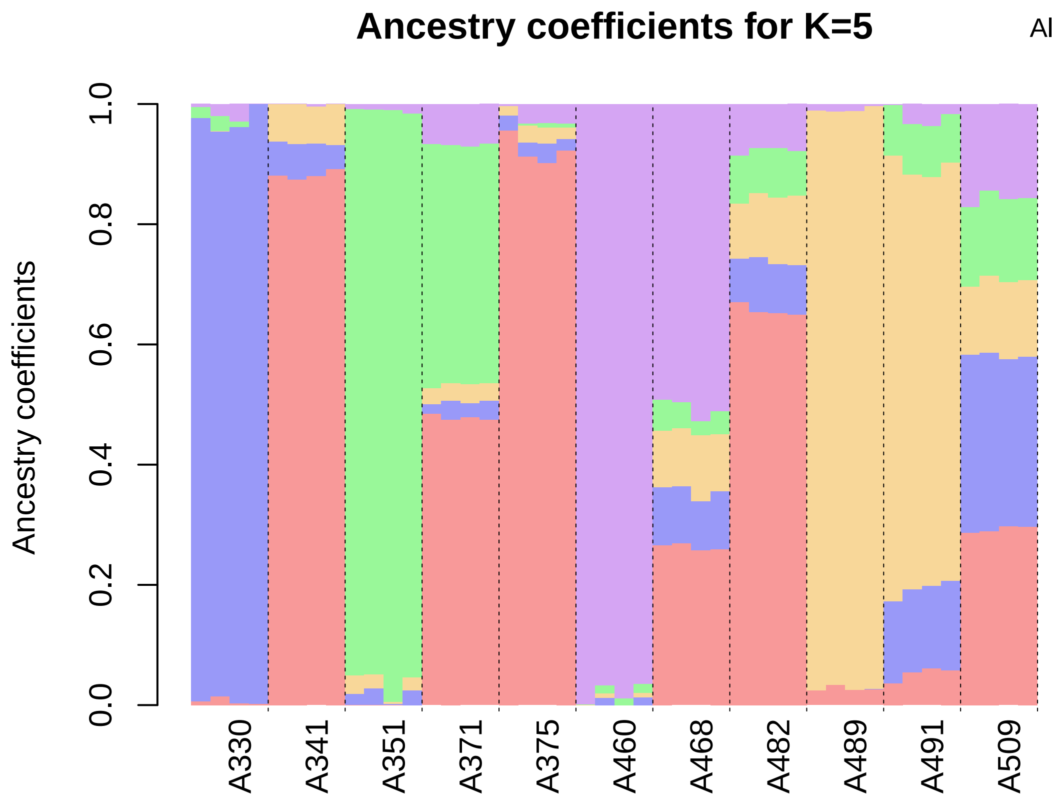

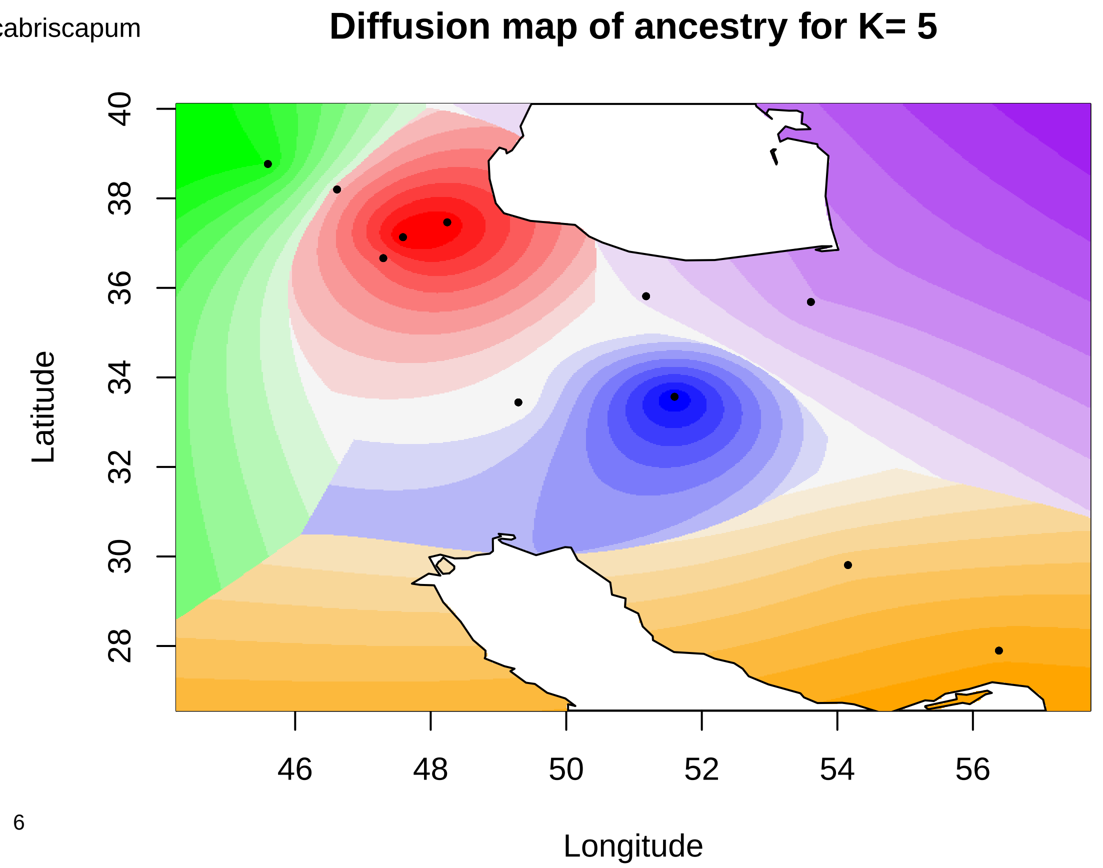

Allium scabriscapum

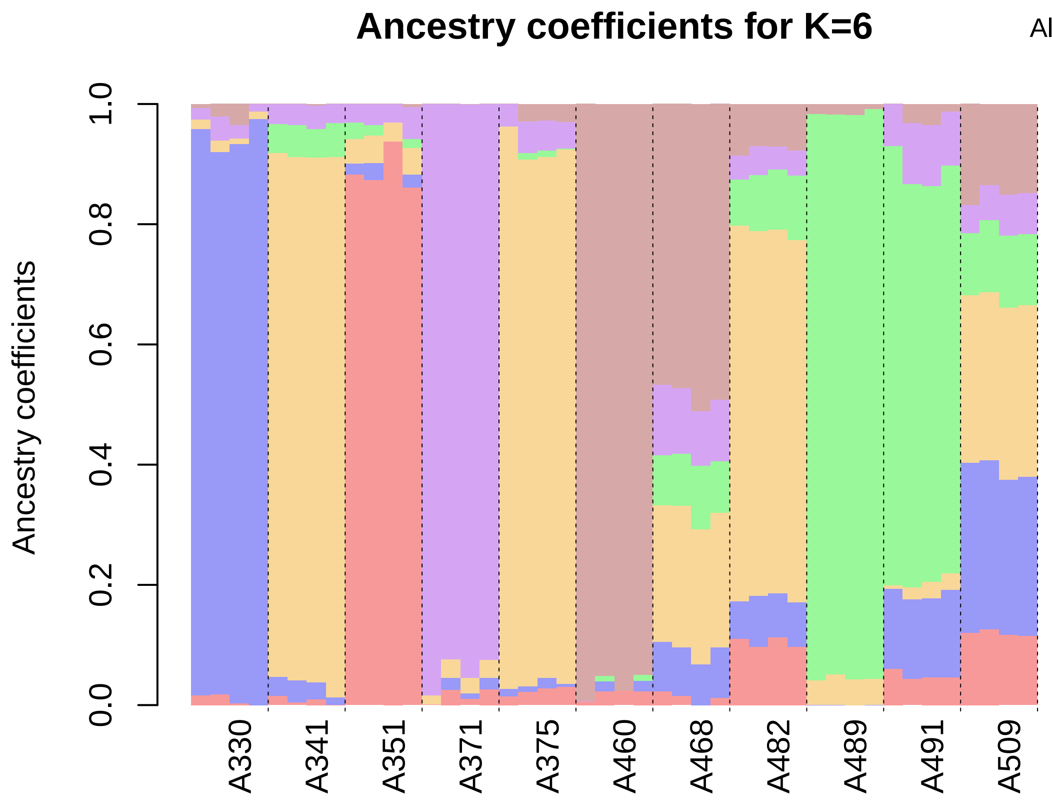

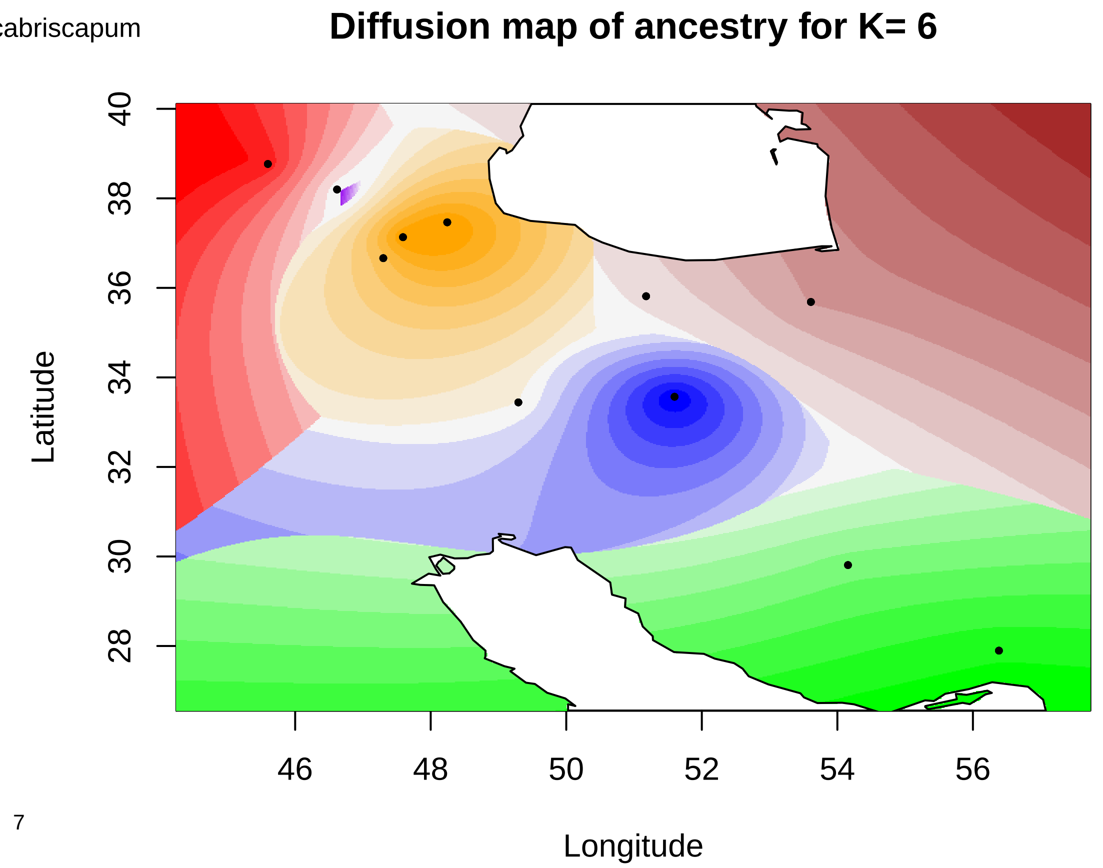

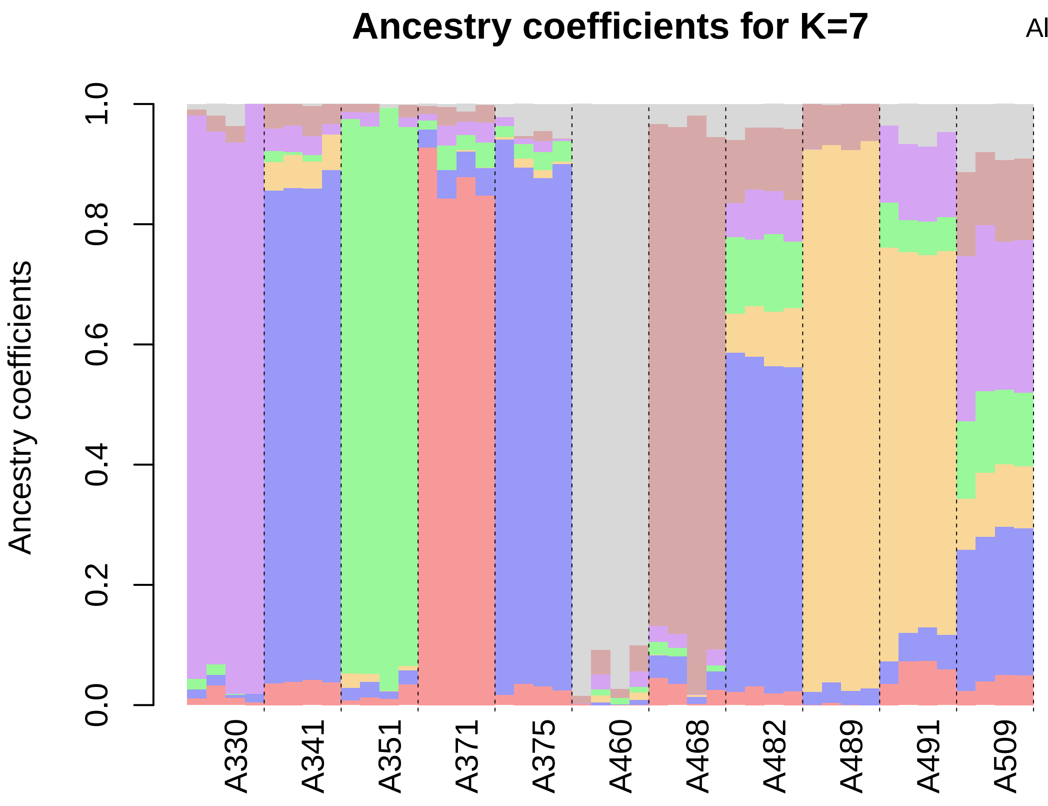

Allium scabriscapum

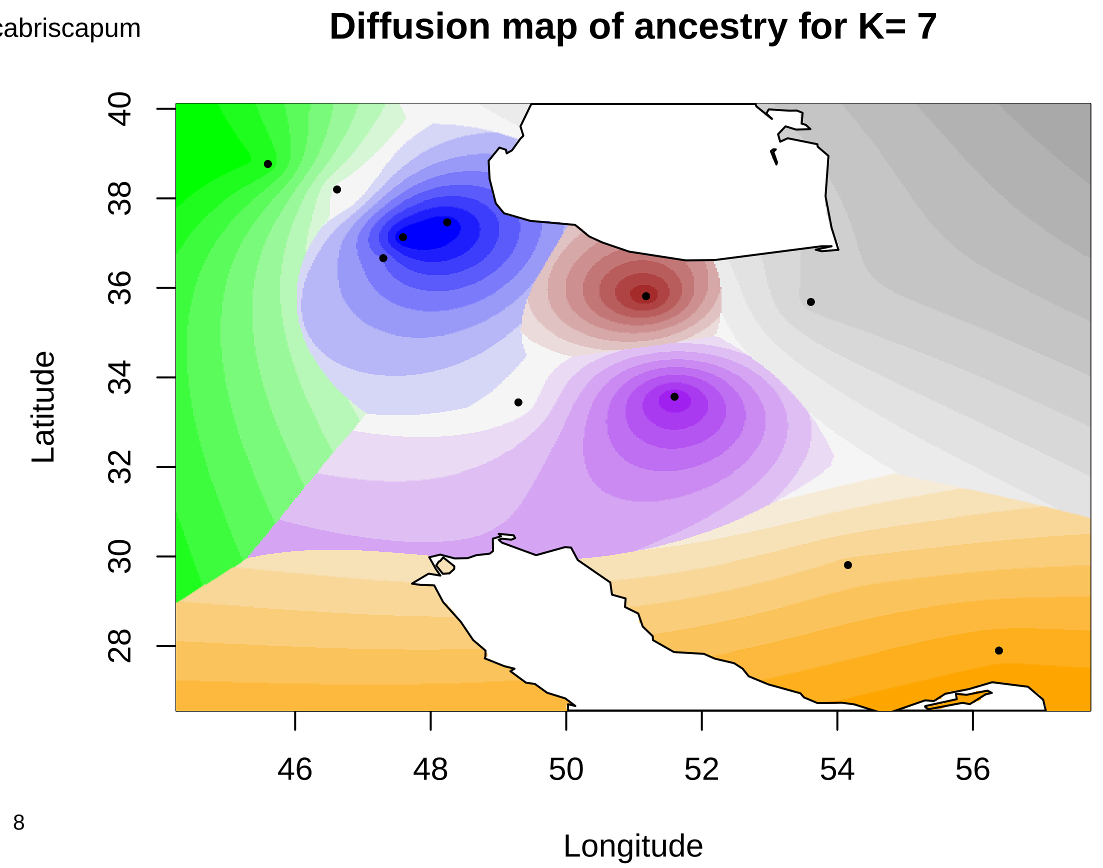

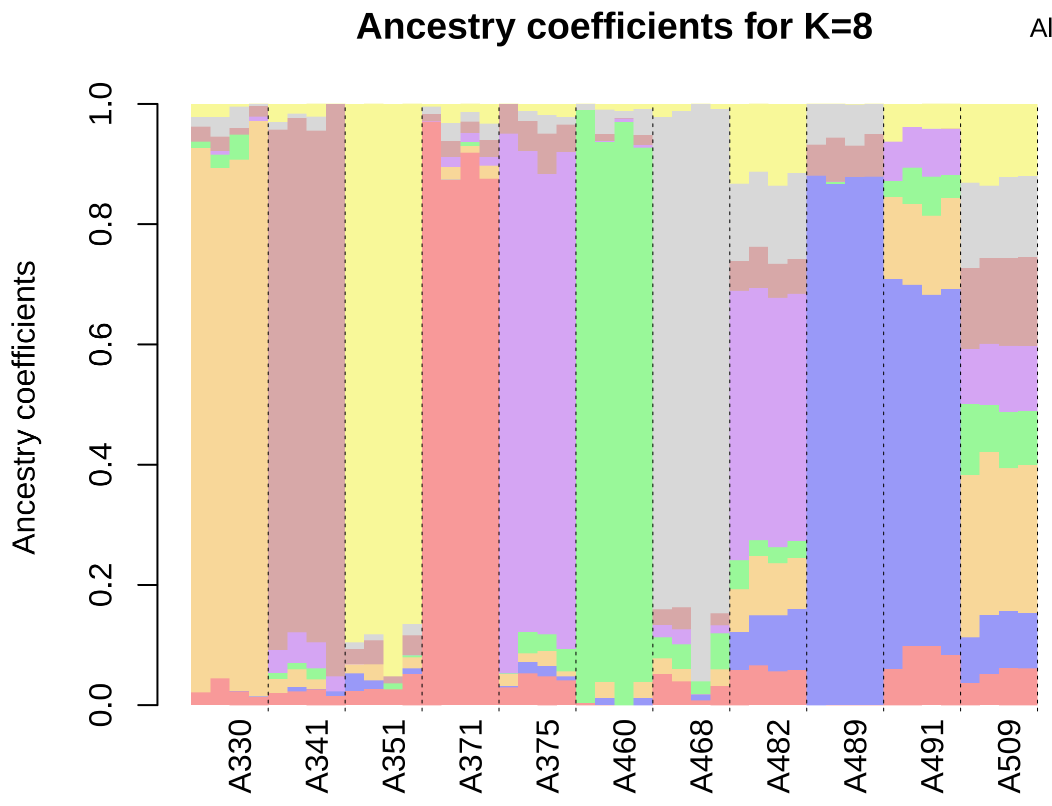

Allium scabriscapum

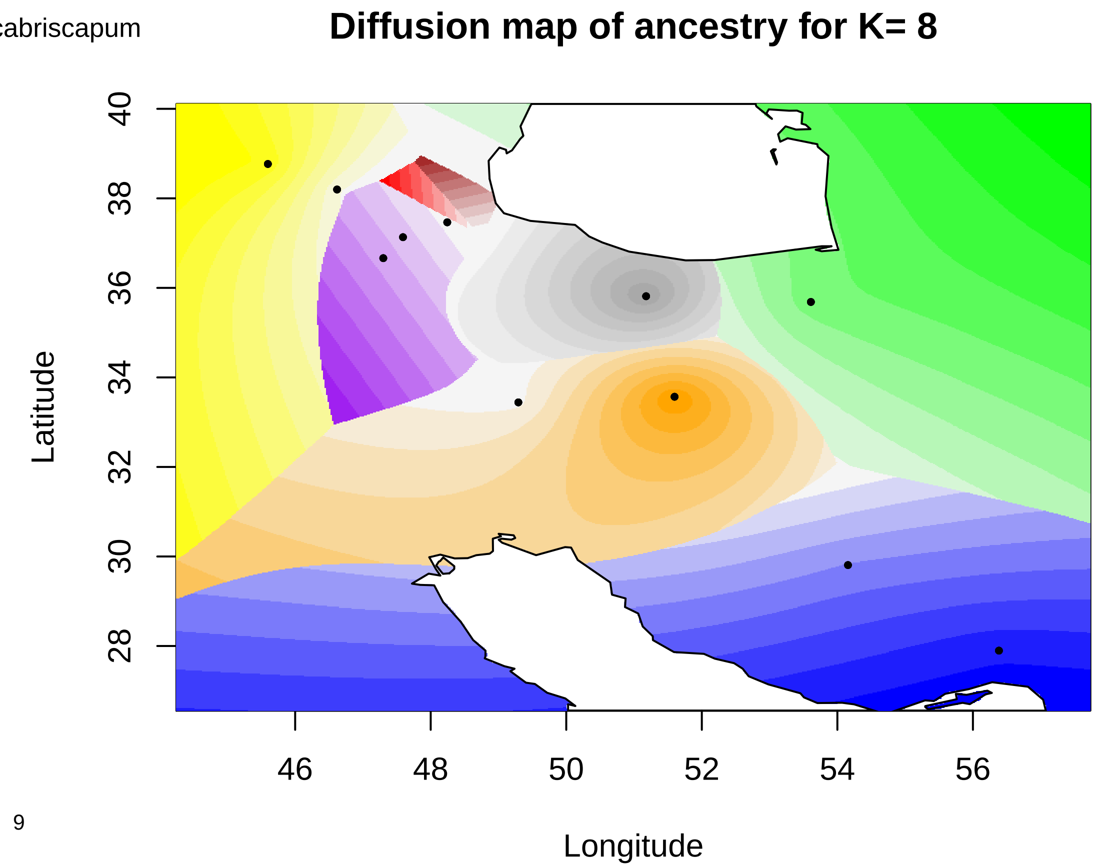

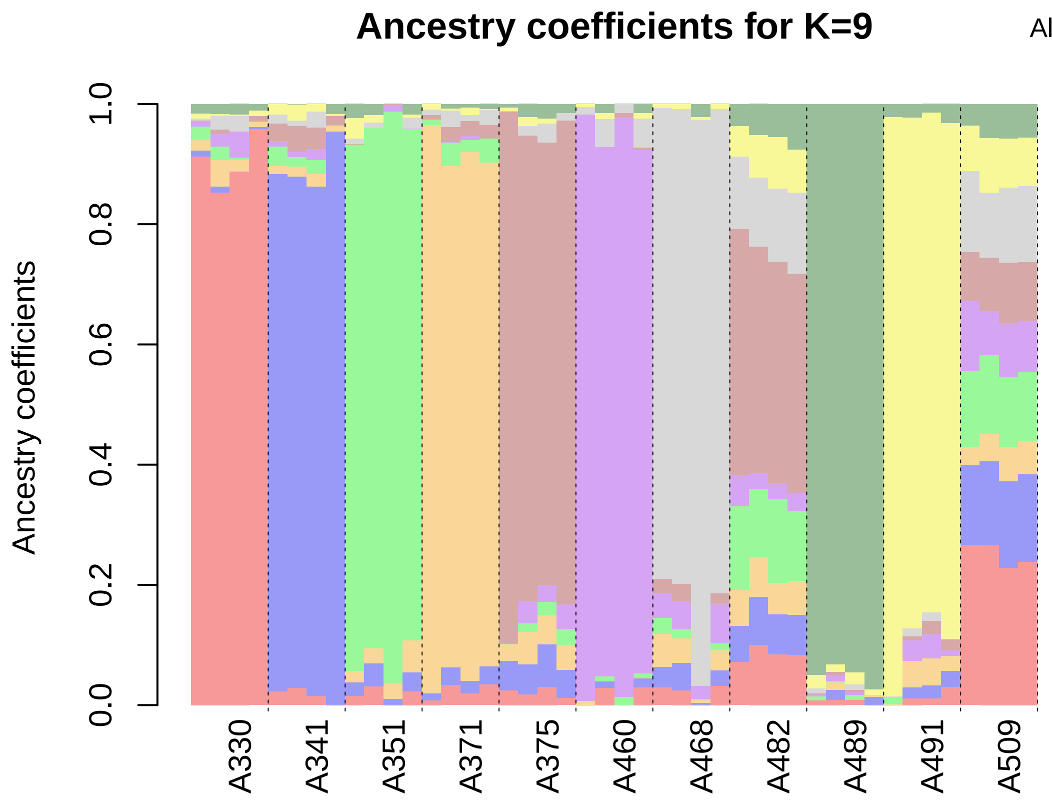

Allium scabriscapum

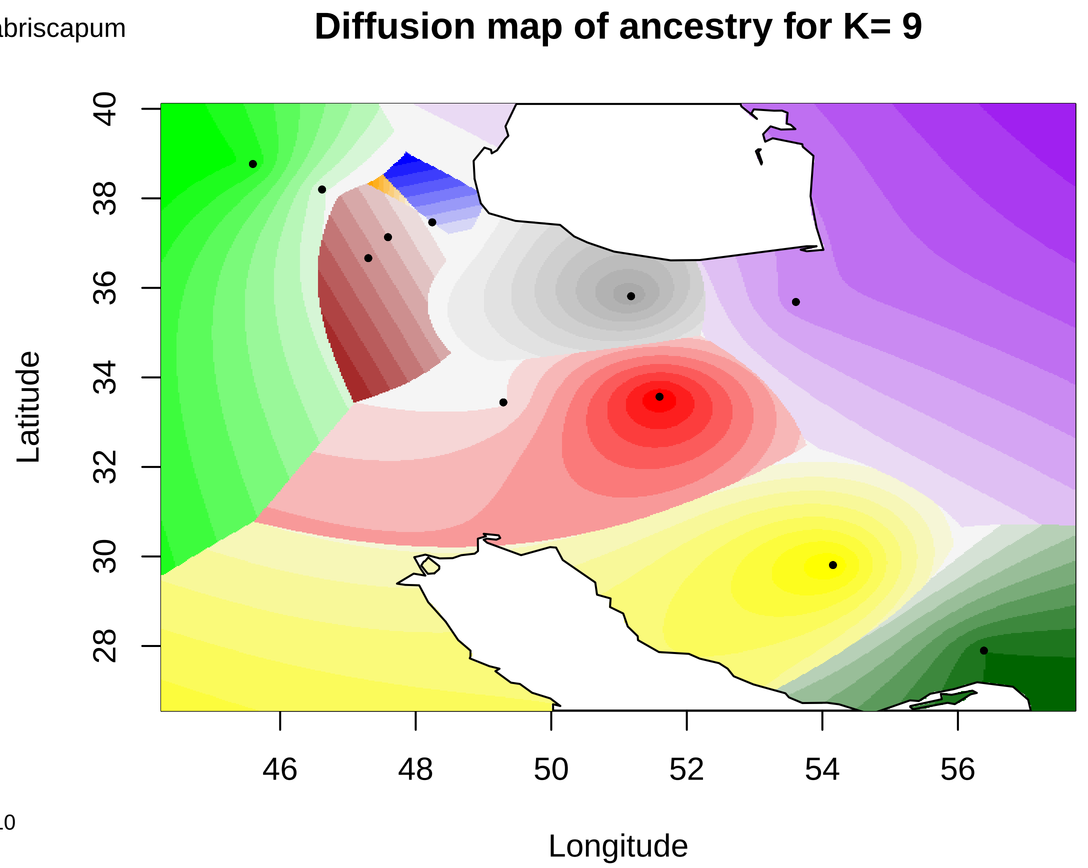

Allium scabriscapum

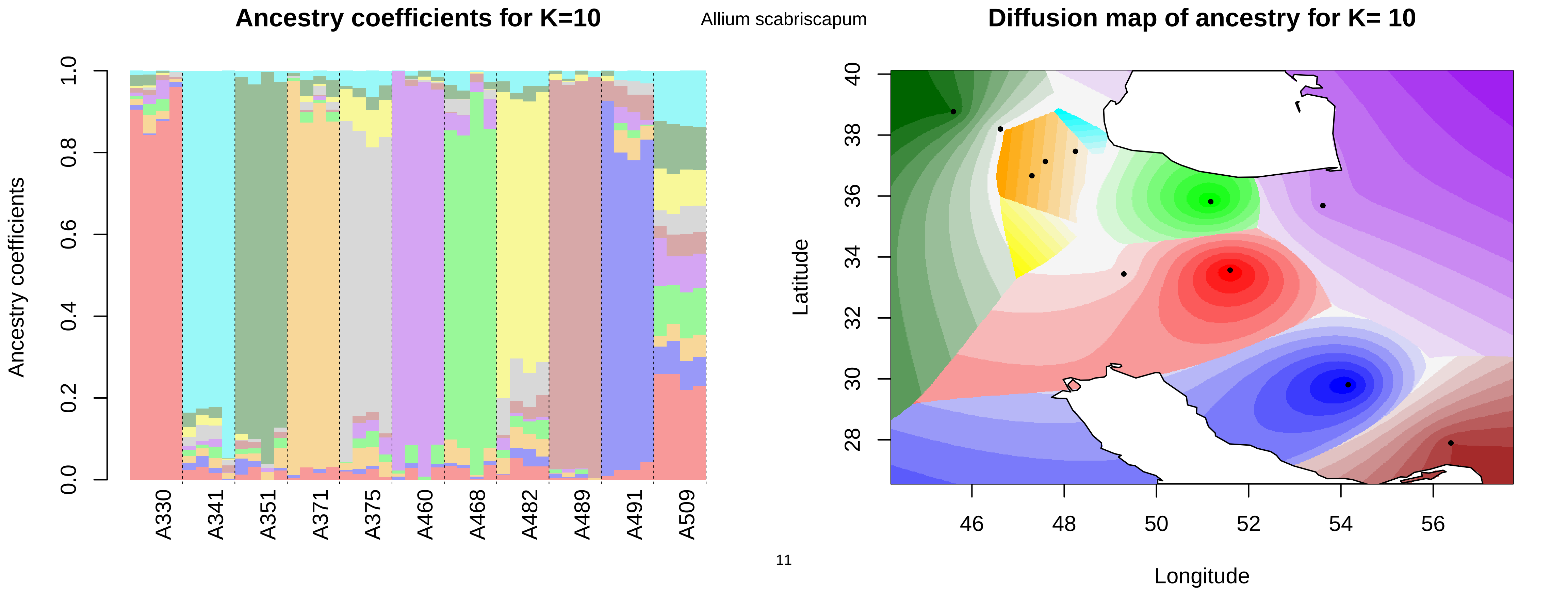

Helichrysum oligocephalum

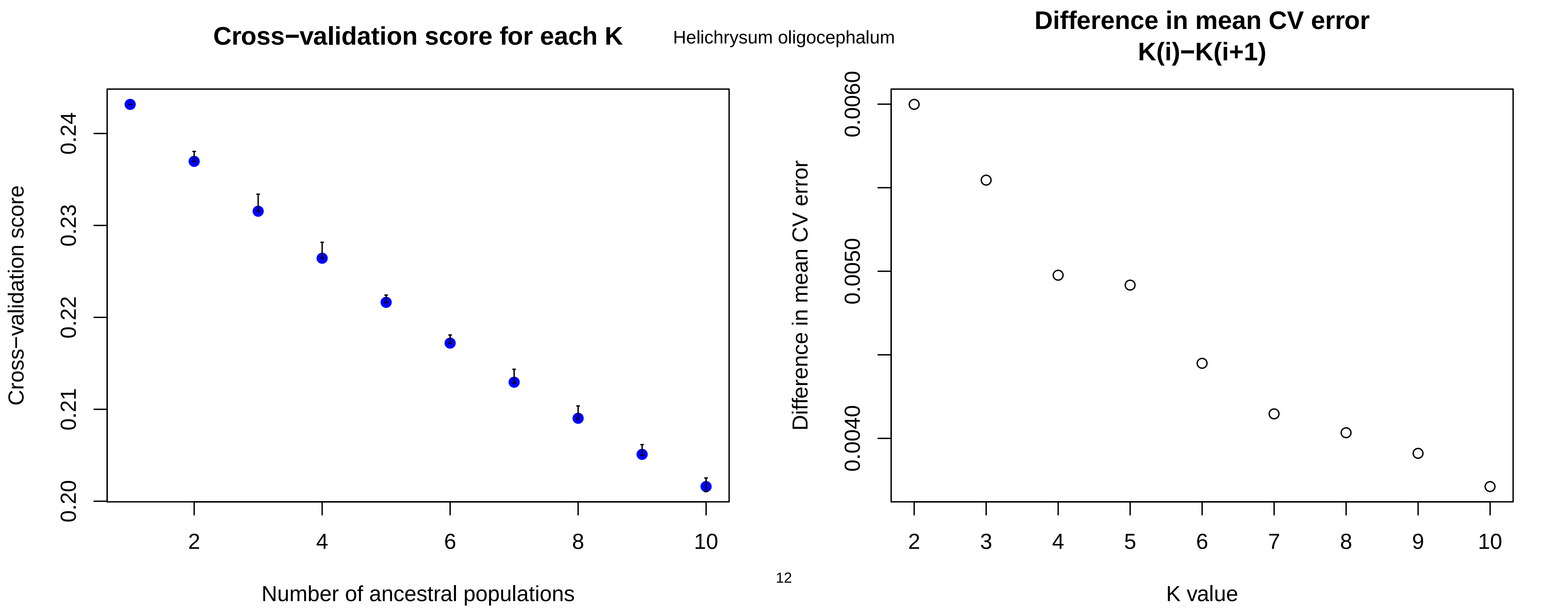

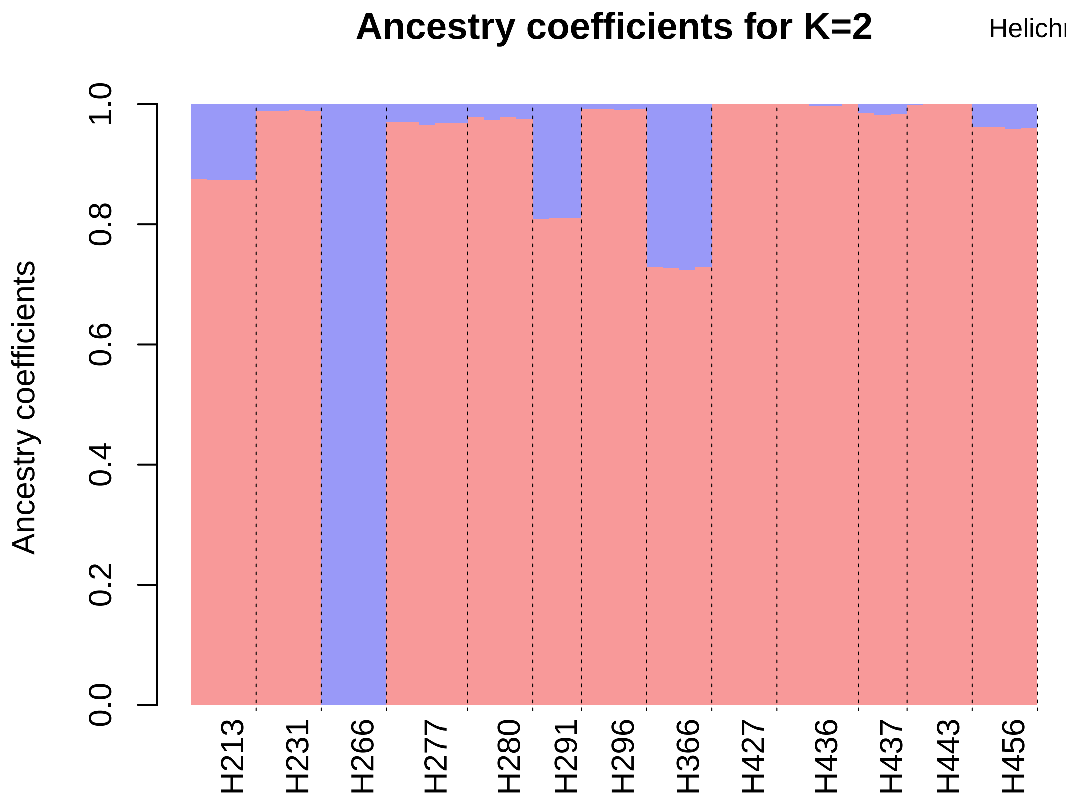

Helichrysum oligocephalum

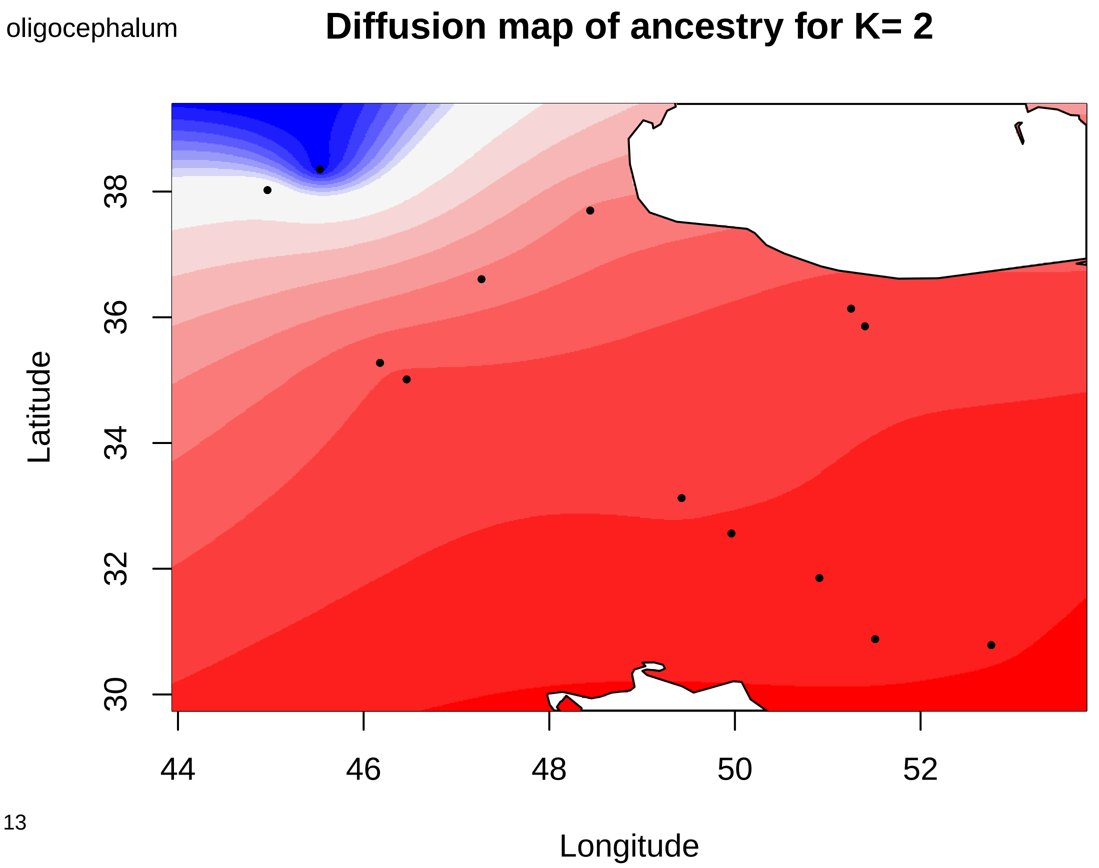

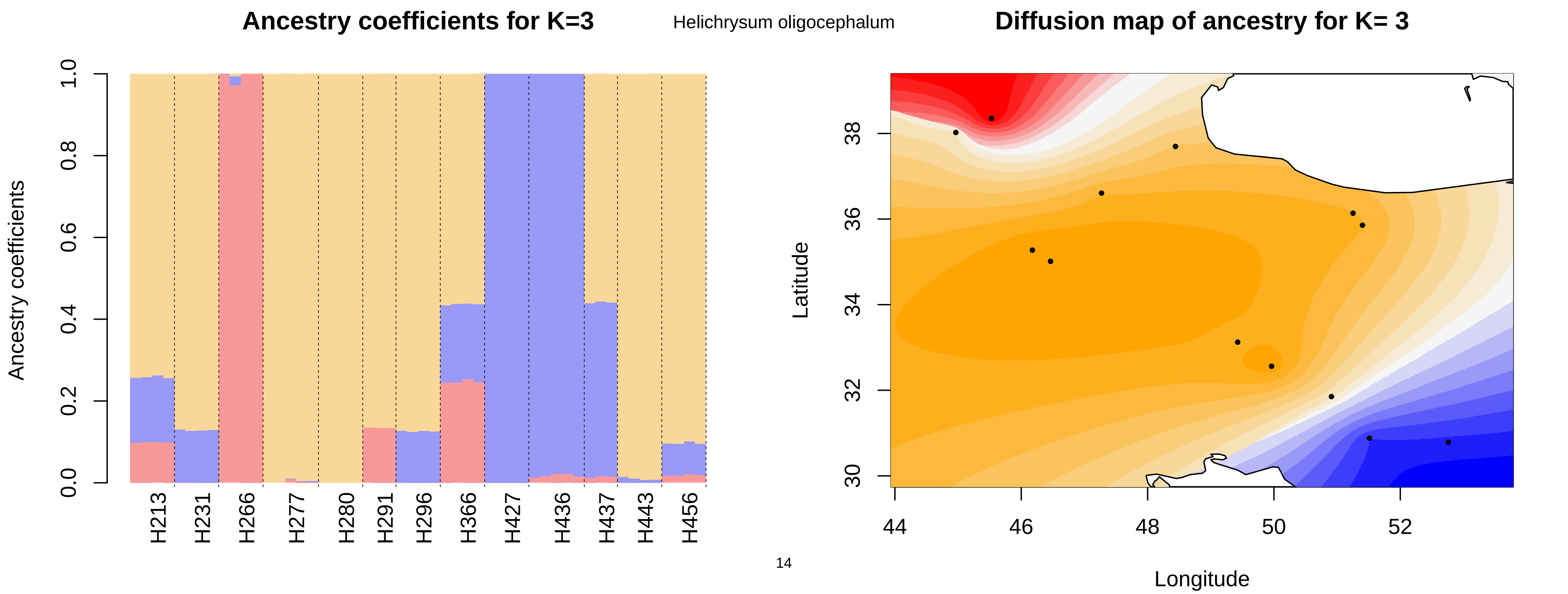
Ancestry coefficients for K=3
Helichrysum oligocephalum
Diffusion map of ancestry for K= 3
Ancestry coefficients
0.0
0.2
0.4
0.6
0.8
1.0
H213
H231
H266
H277
H280
H291
H296
H366
H427
H436
H437
H443
H456
Latitude
30
32
34
36
38
Longitude
44
46
48
50
52

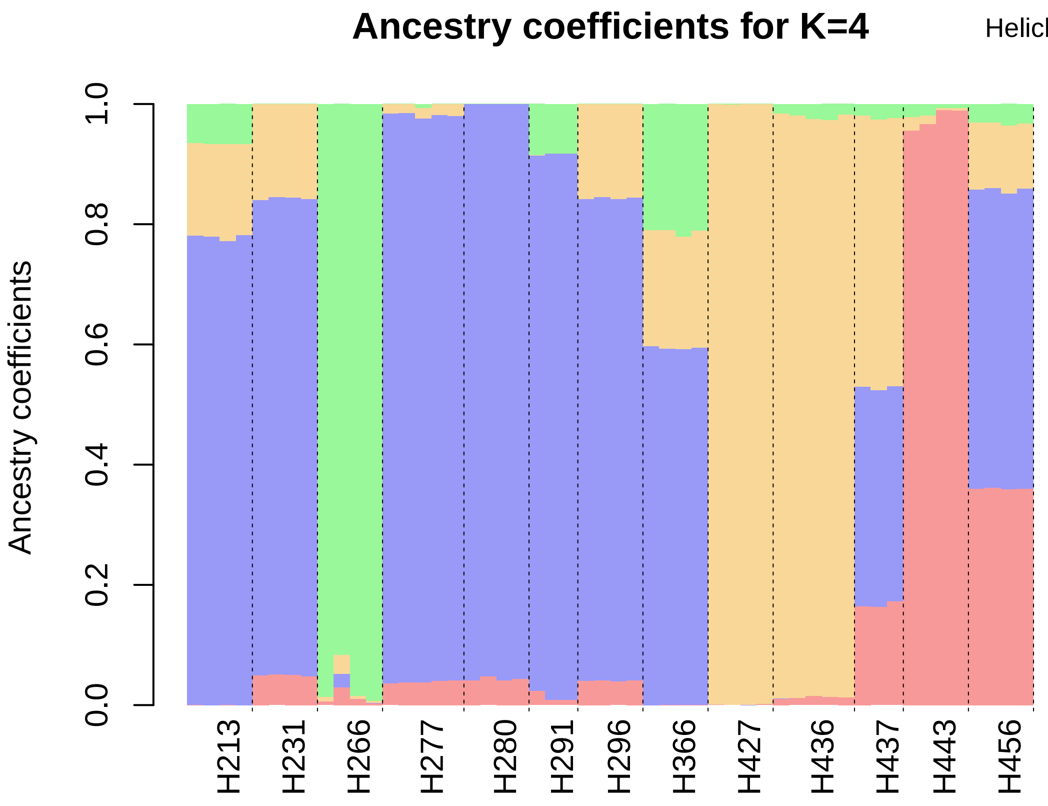

Helichrysum oligocephalum

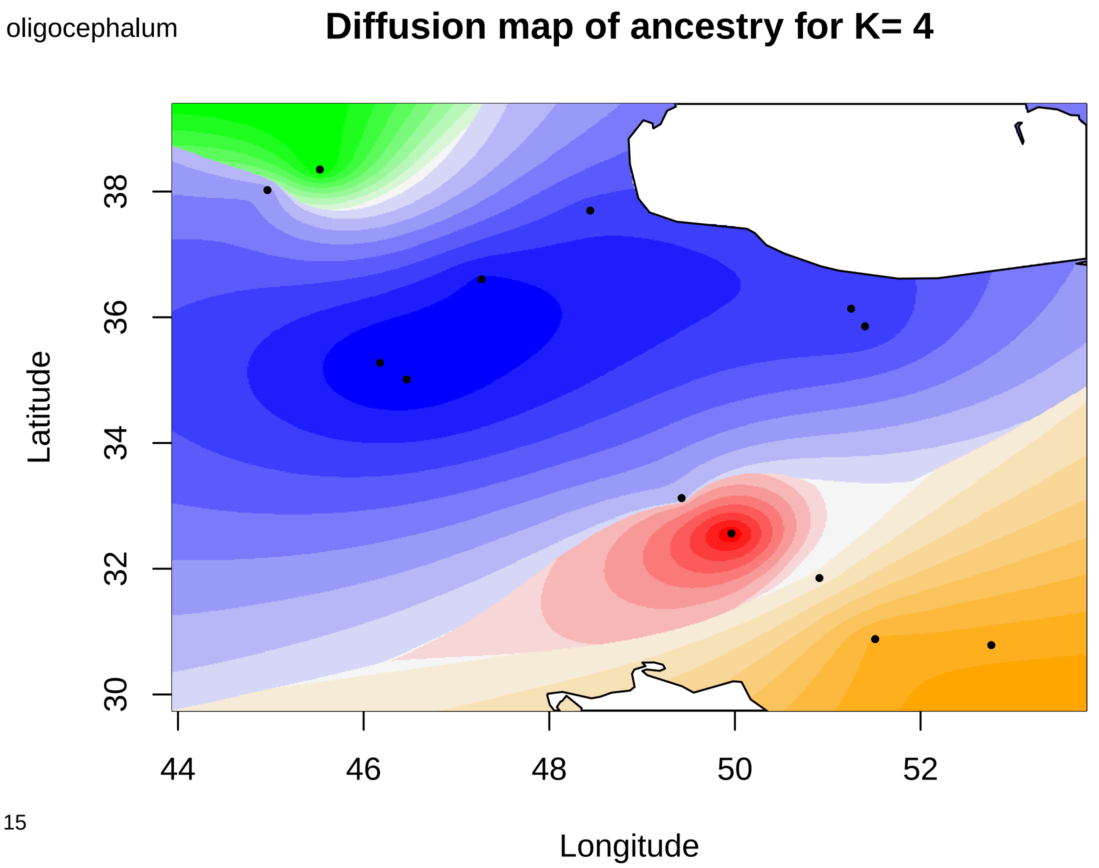

Helichrysum oligocephalum

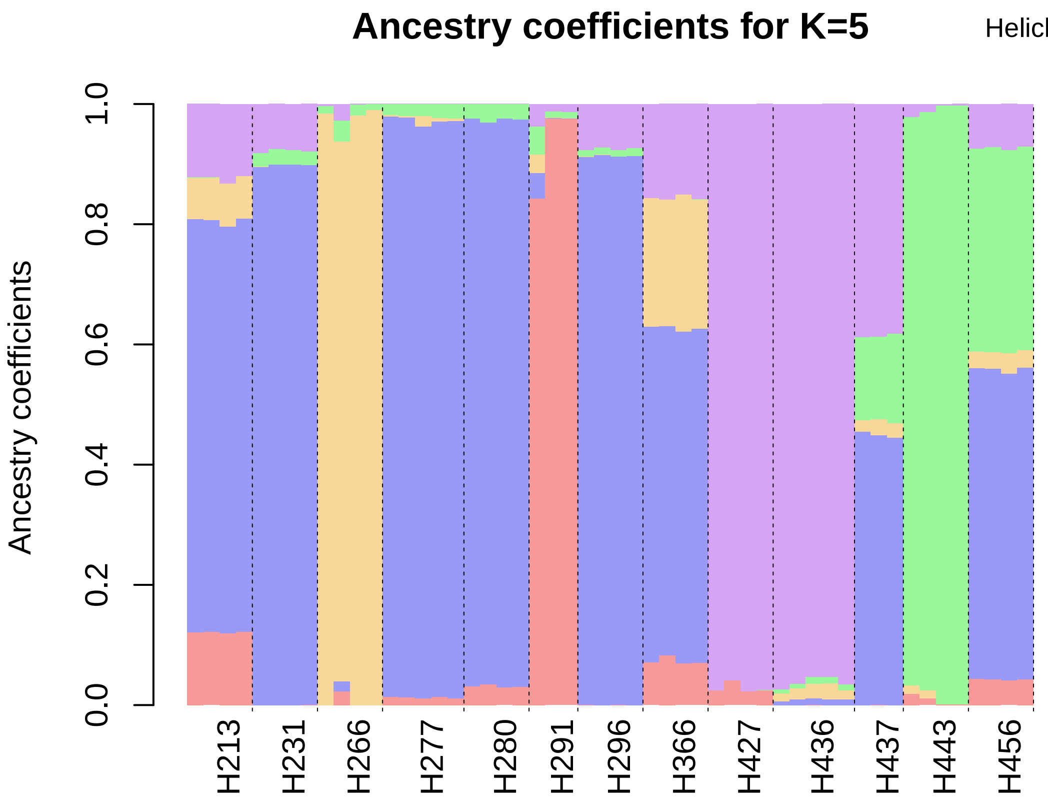

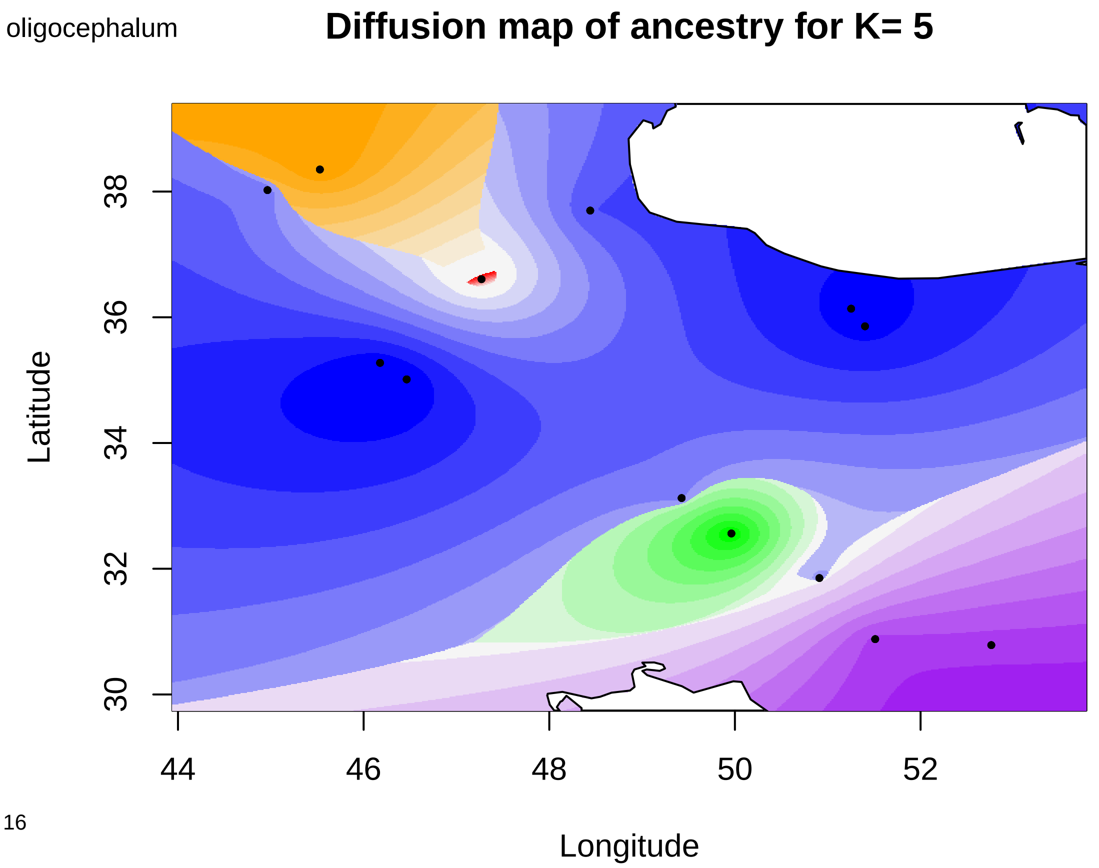

Helichrysum oligocephalum

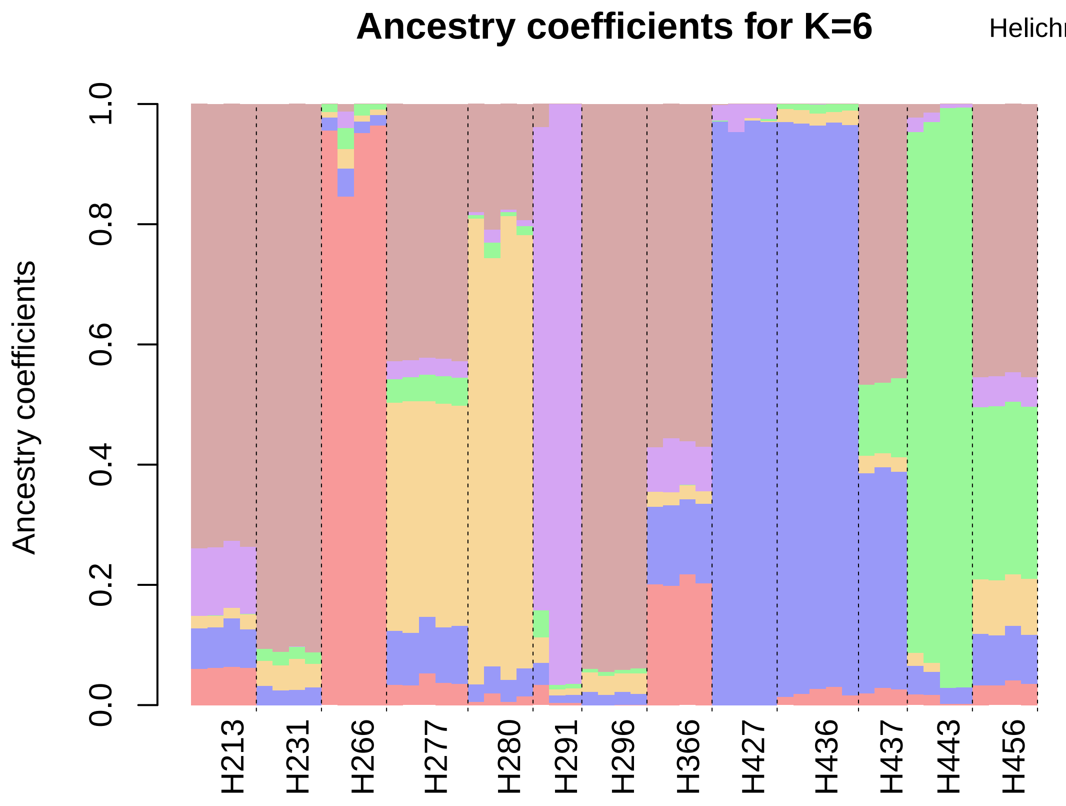

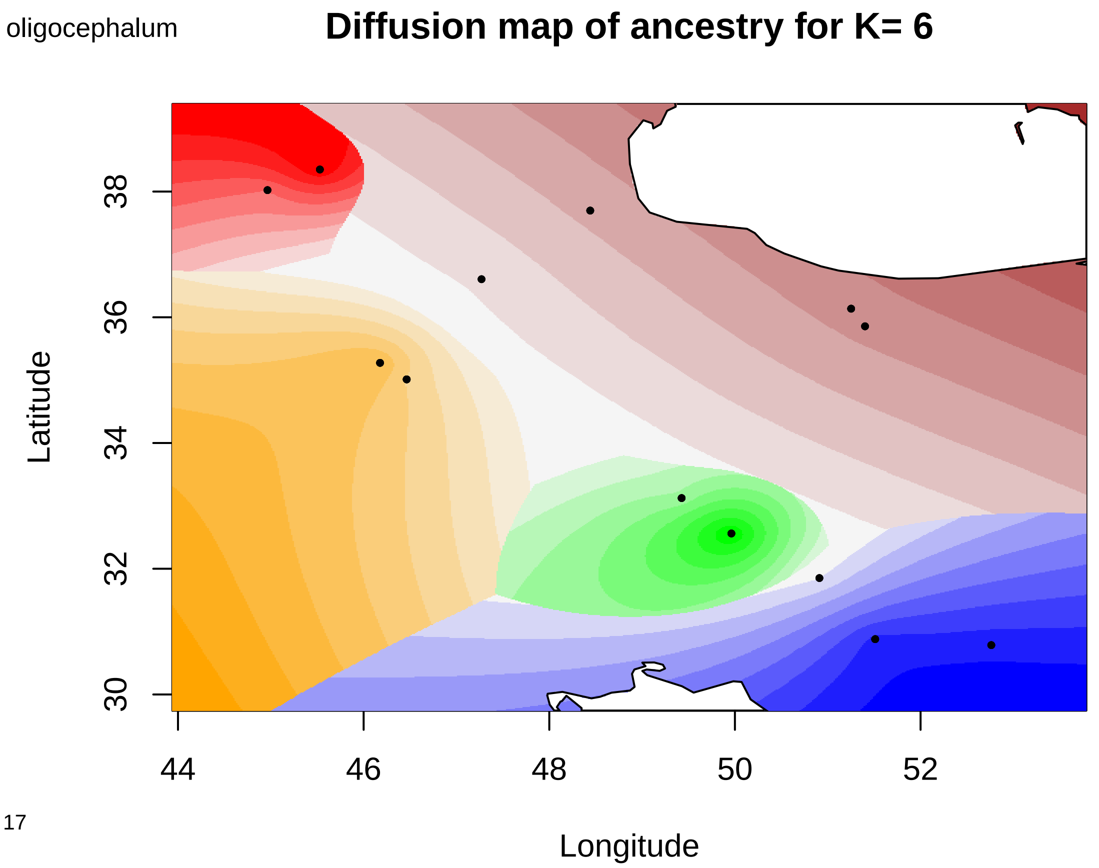

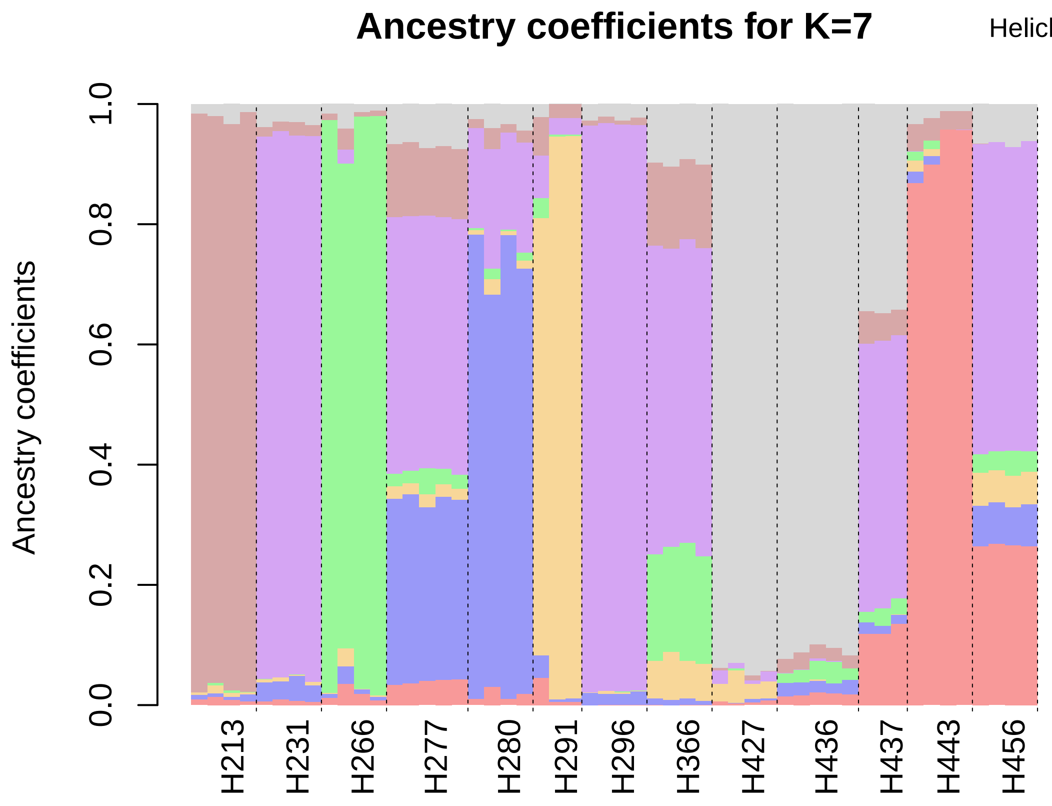

Helichrysum oligocephalum

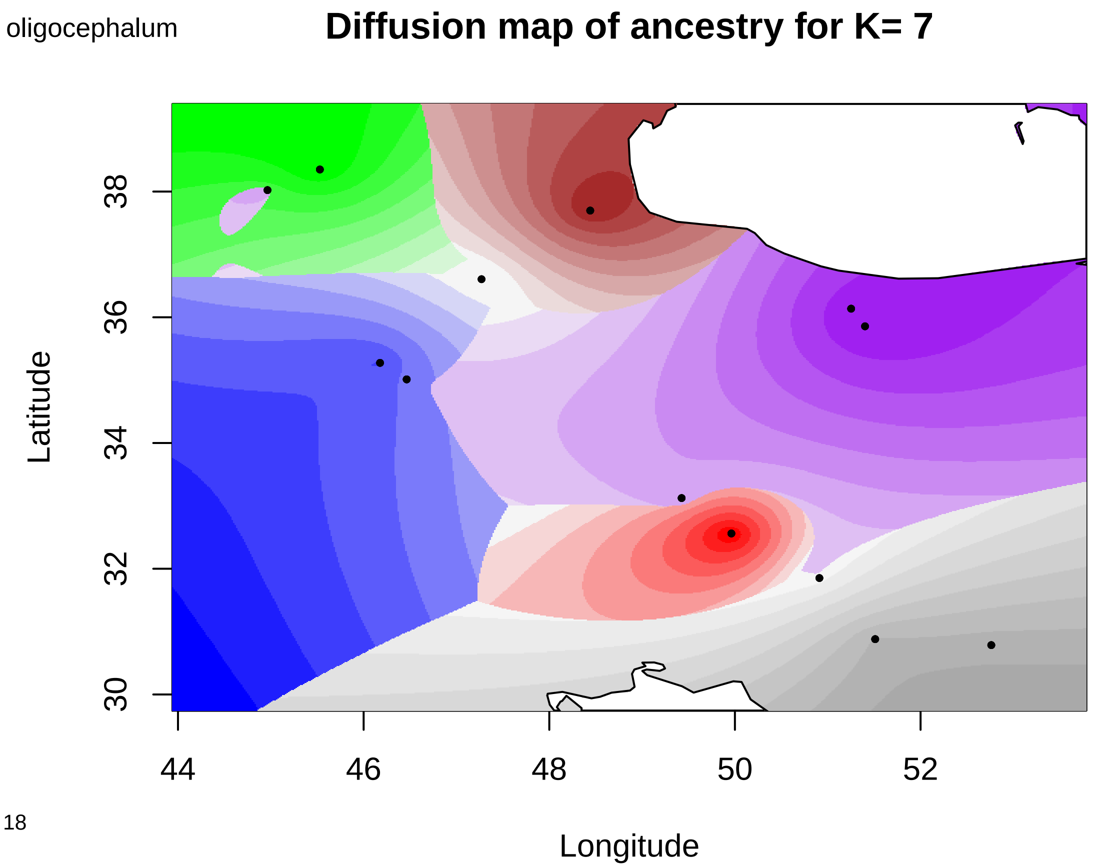

Helichrysum oligocephalum

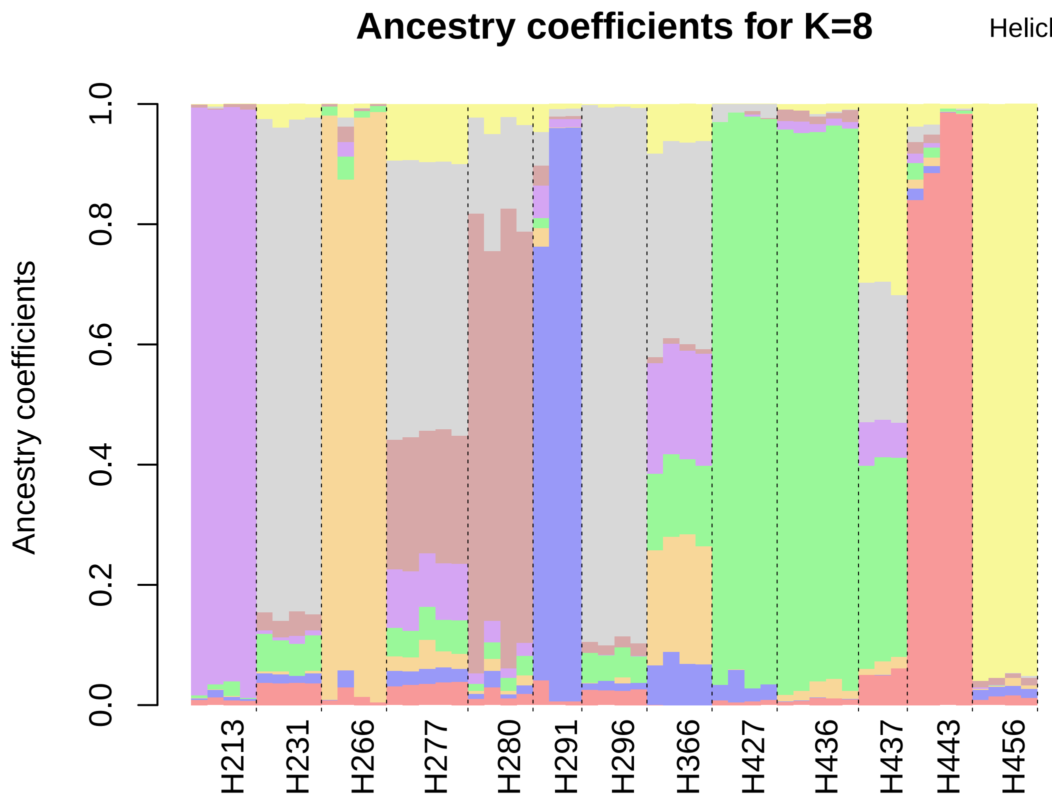

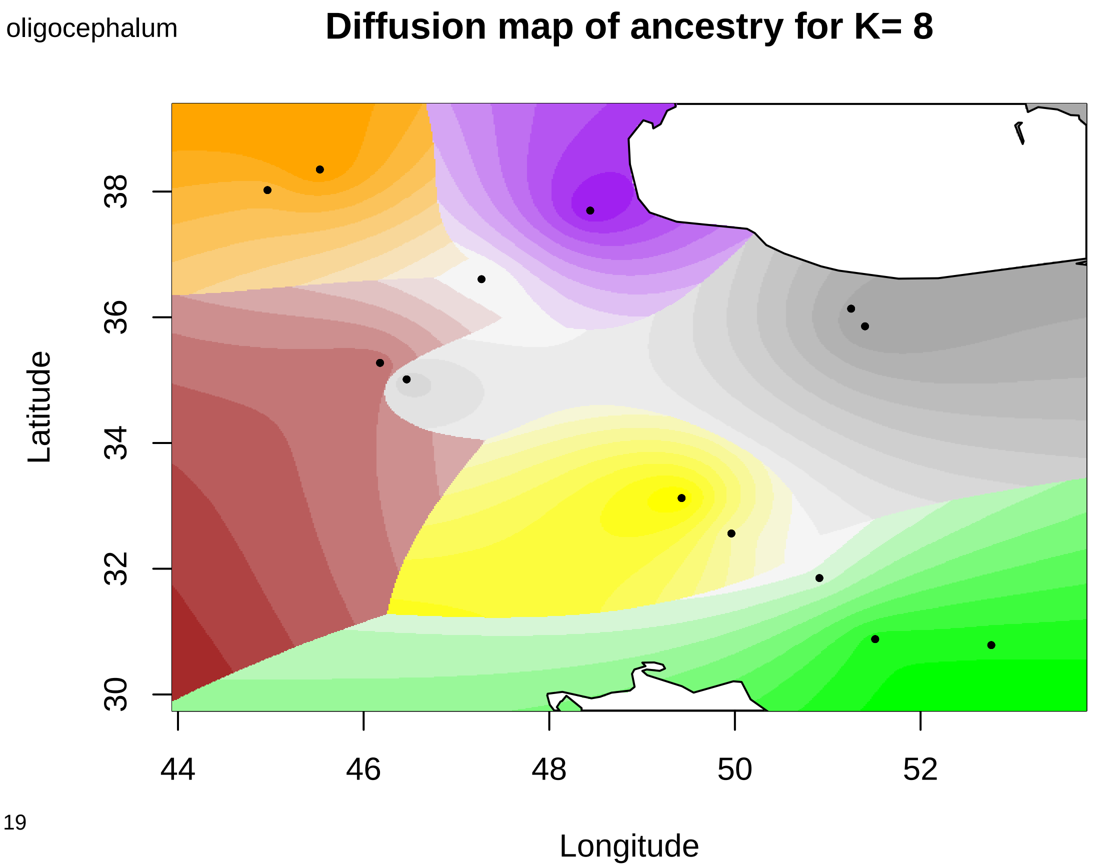

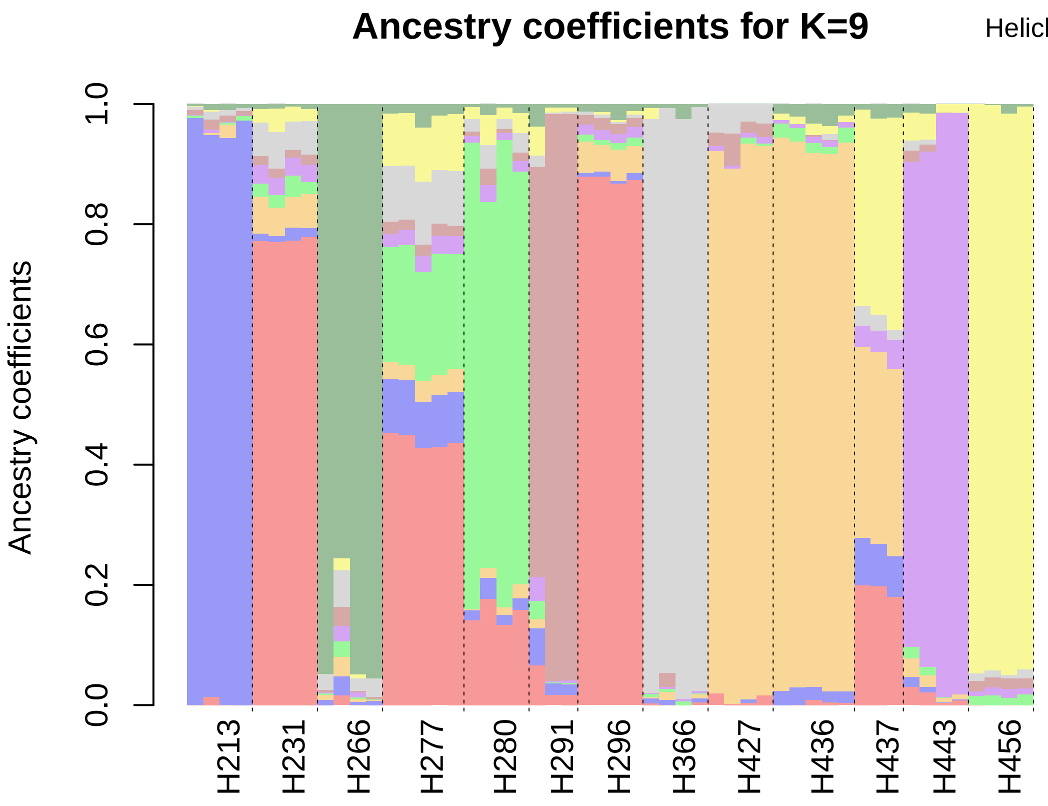

Helichrysum oligocephalum

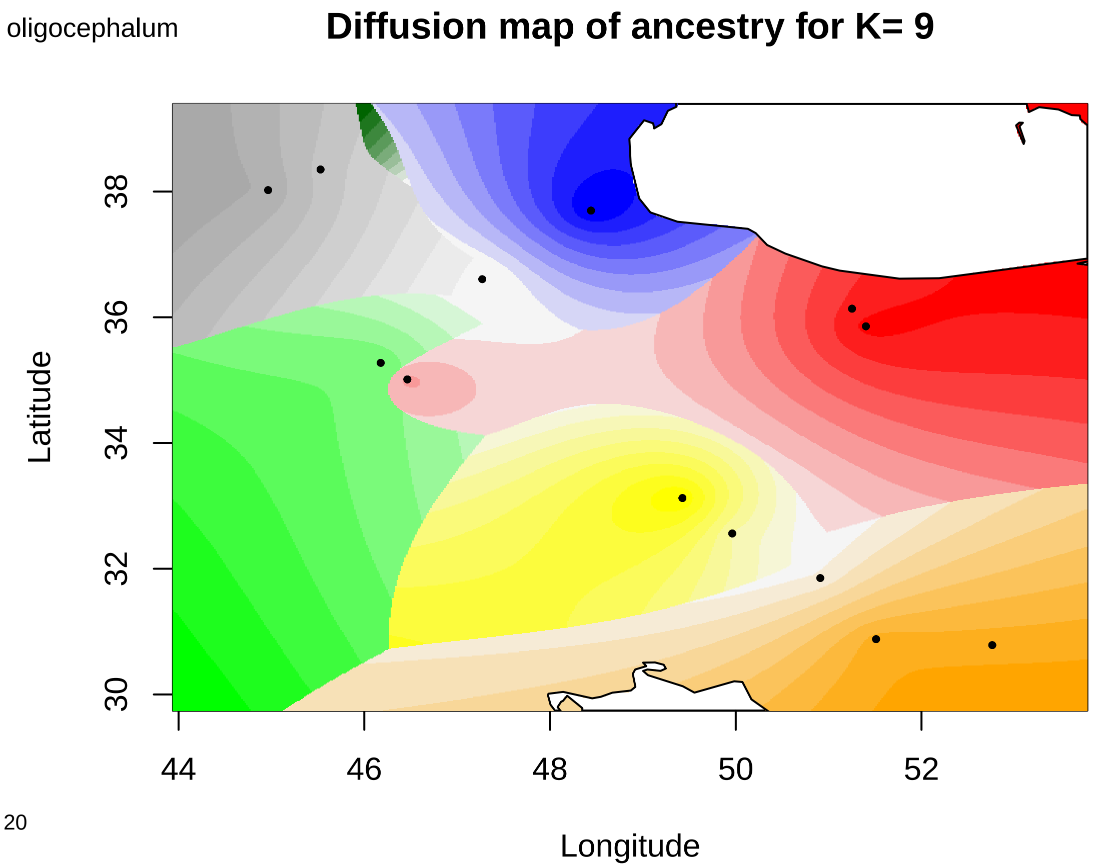

Helichrysum oligocephalum

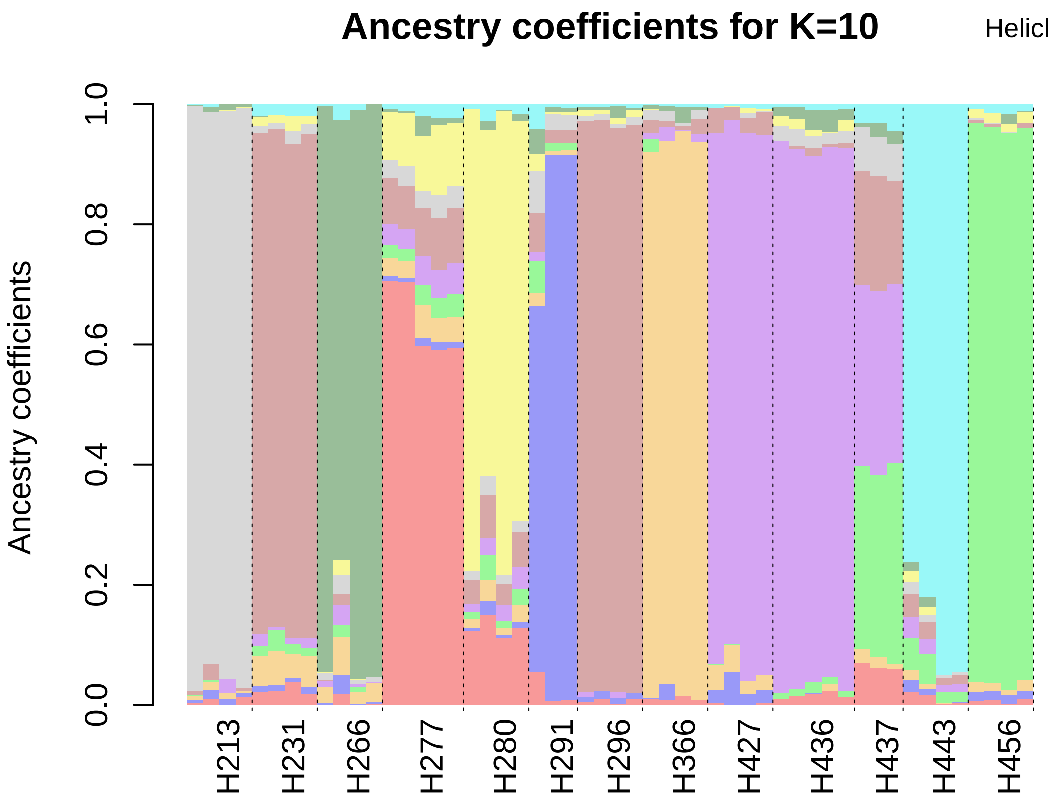

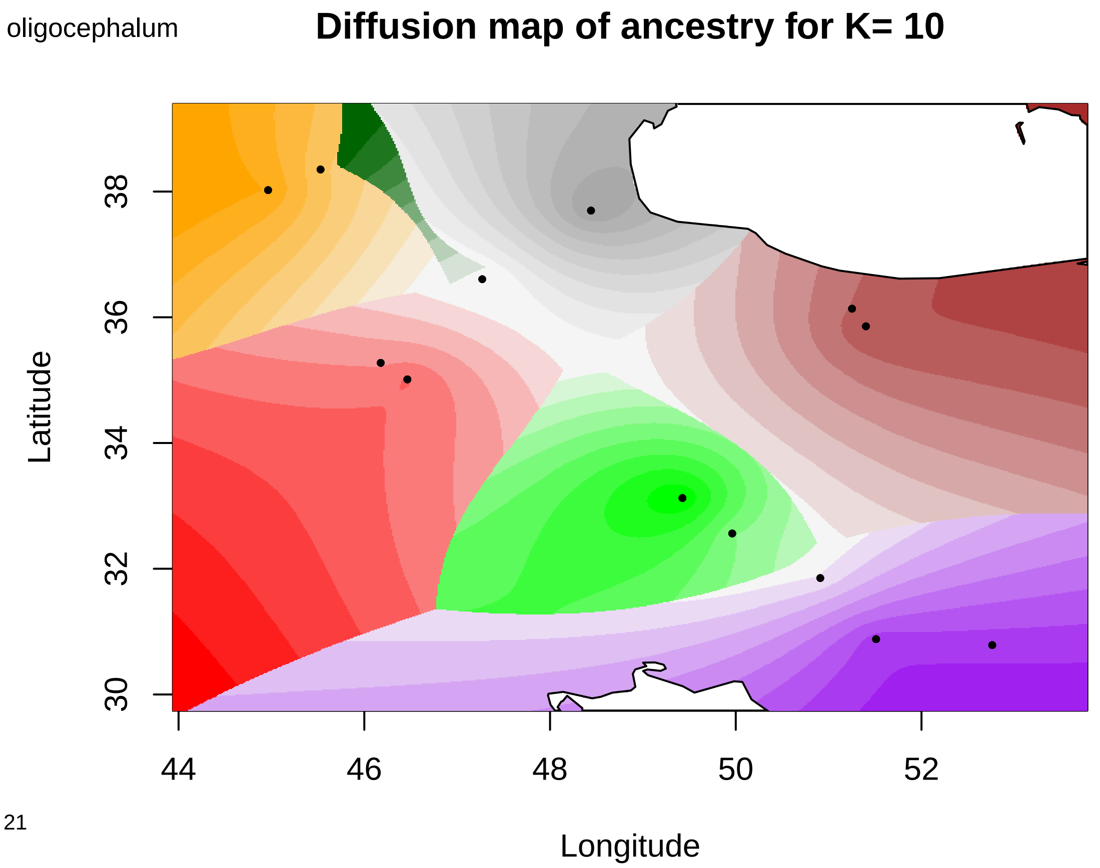

Onosma microcarpa

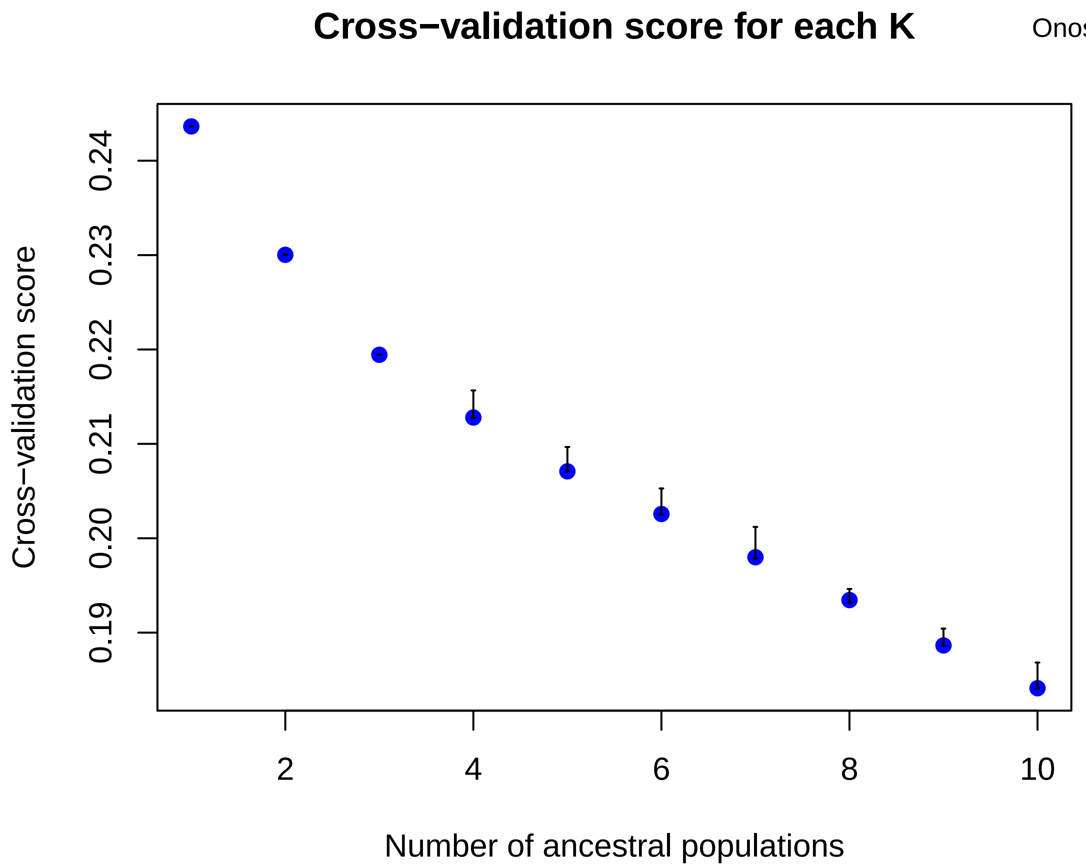

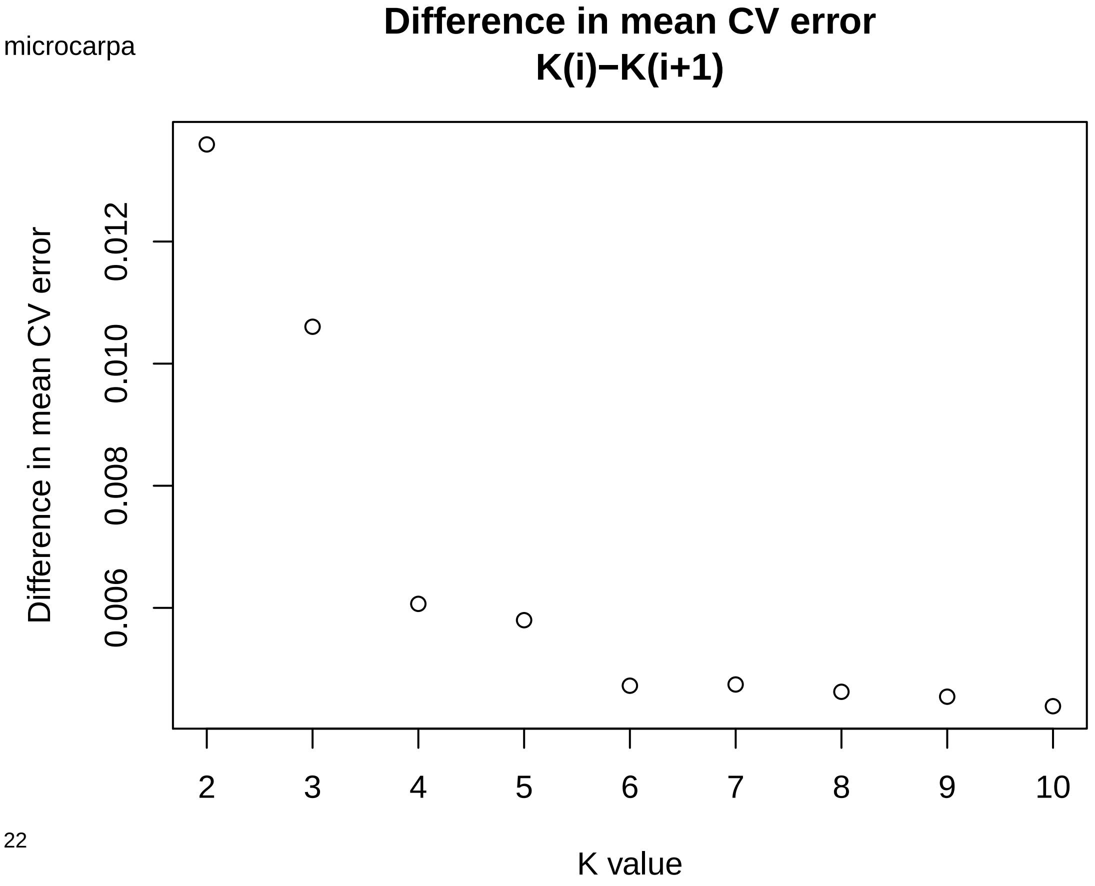

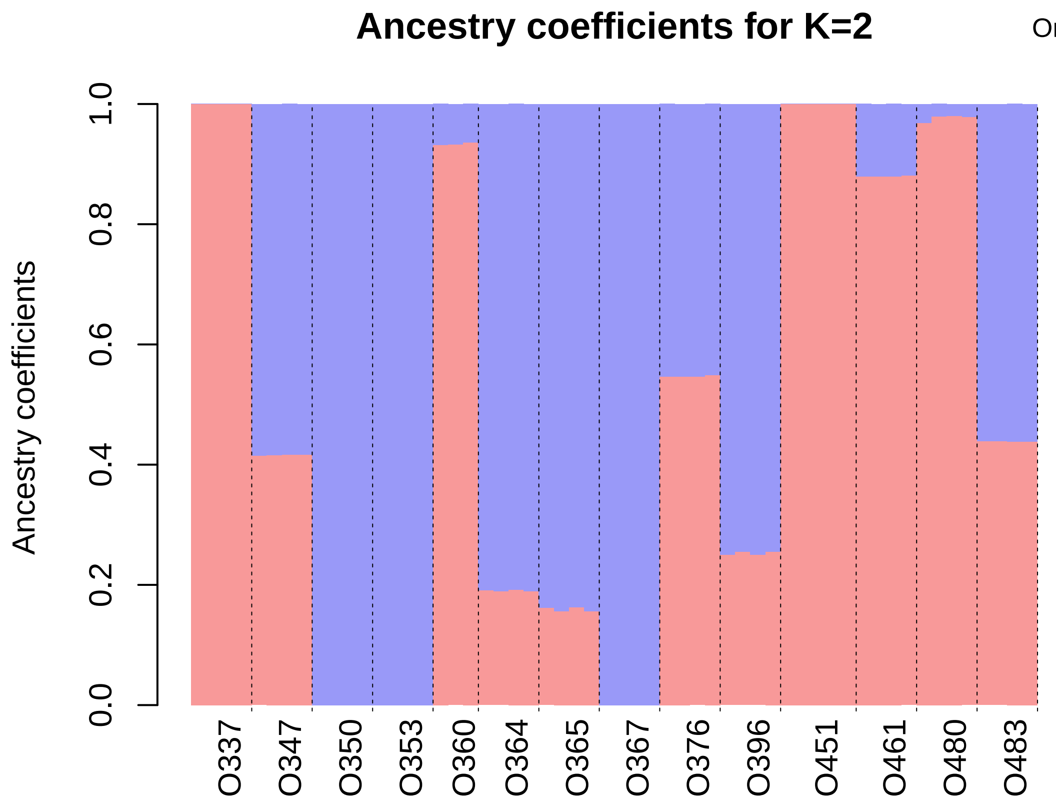

Onosma microcarpa

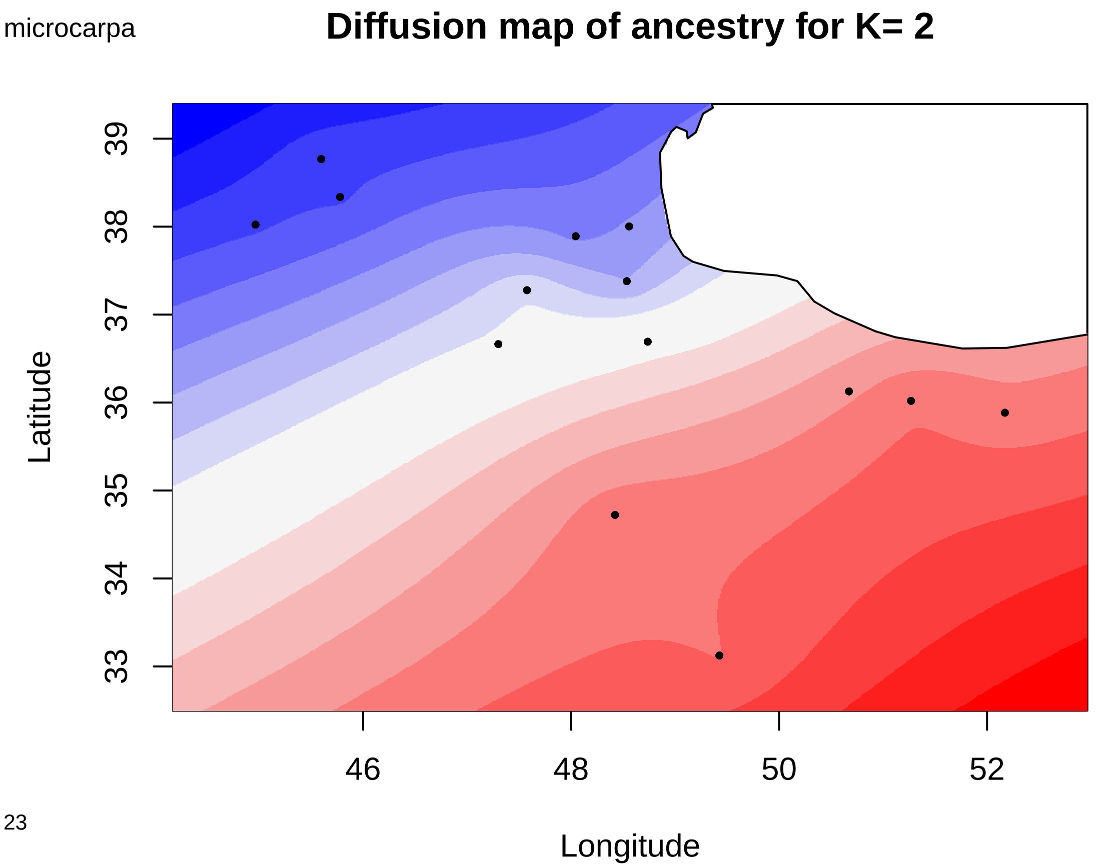

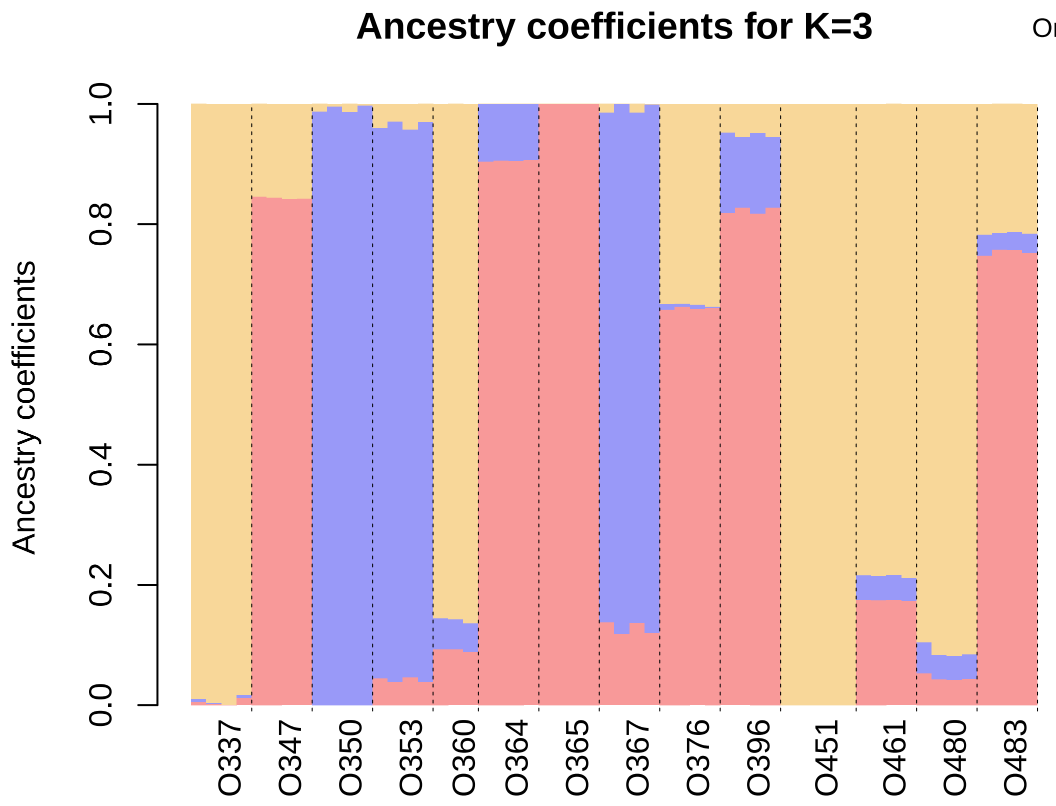

Onosma microcarpa

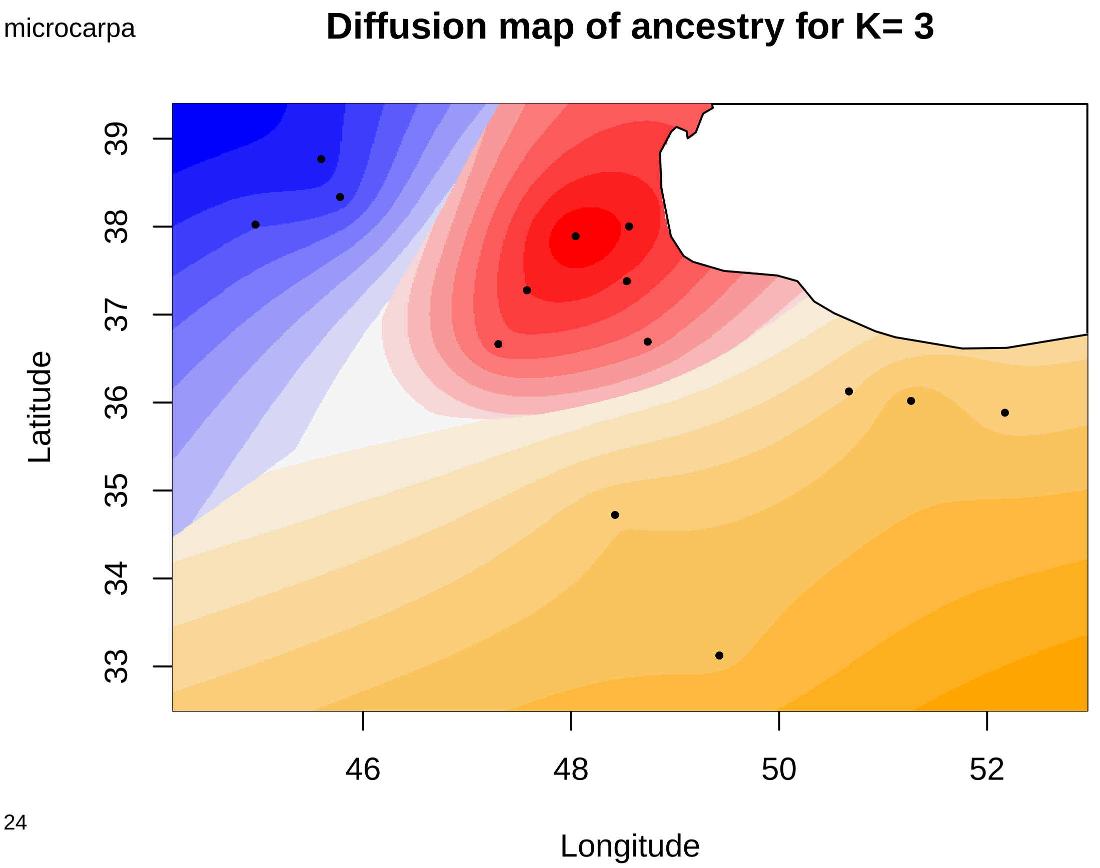

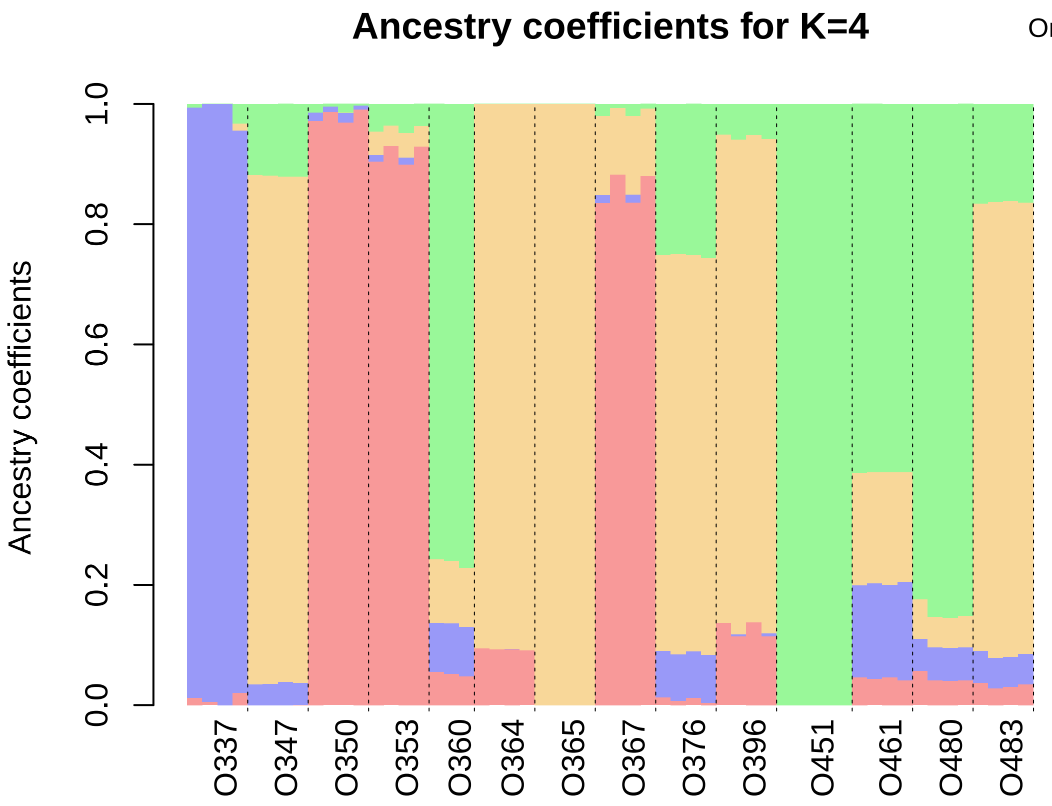

Onosma microcarpa

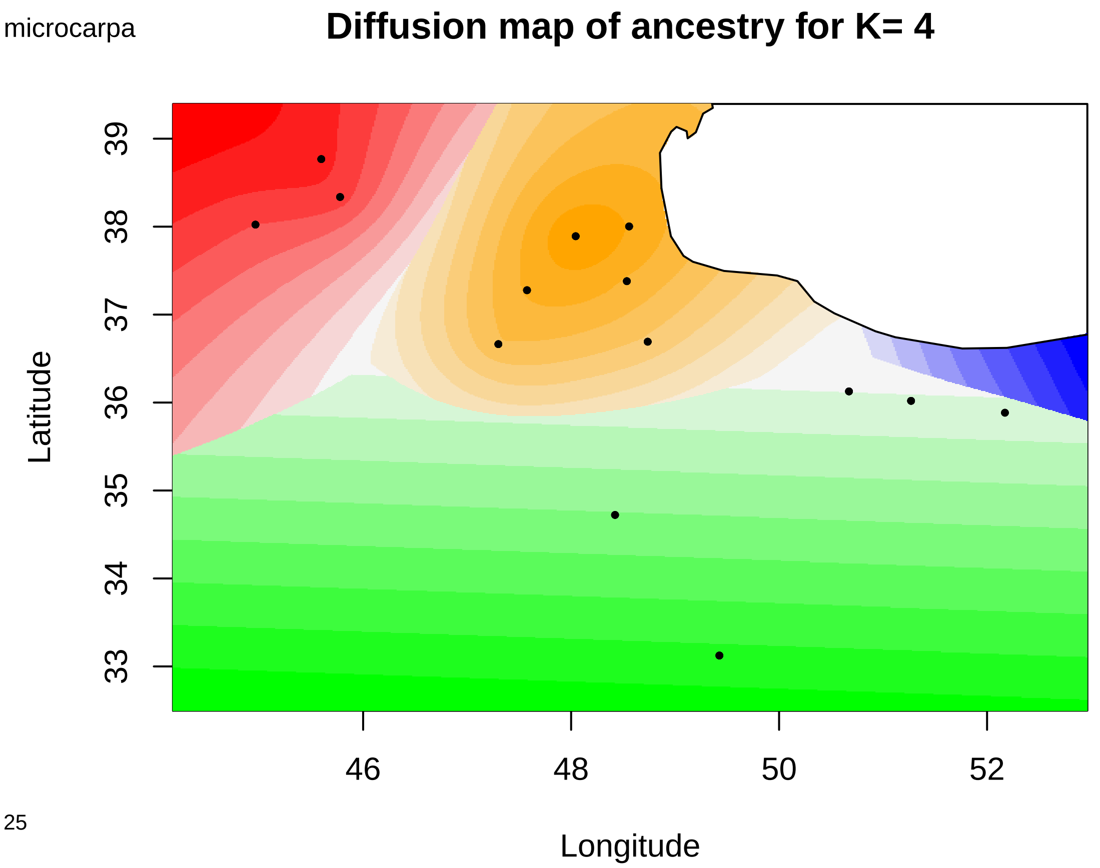

Onosma microcarpa

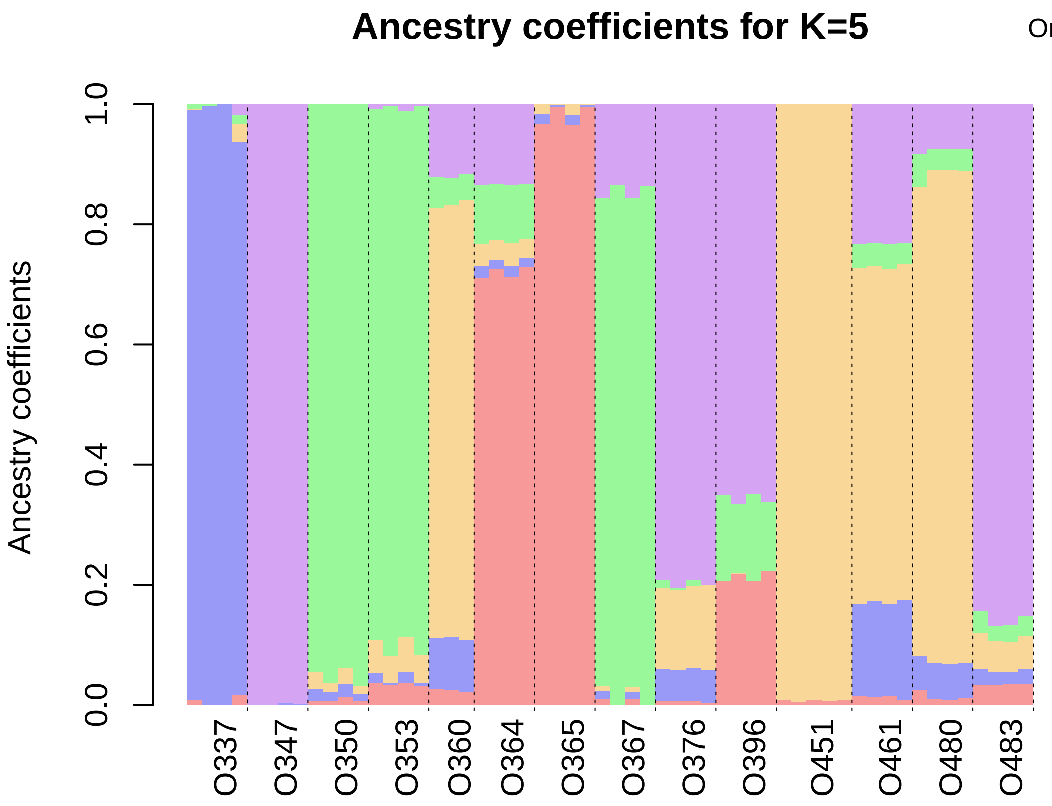

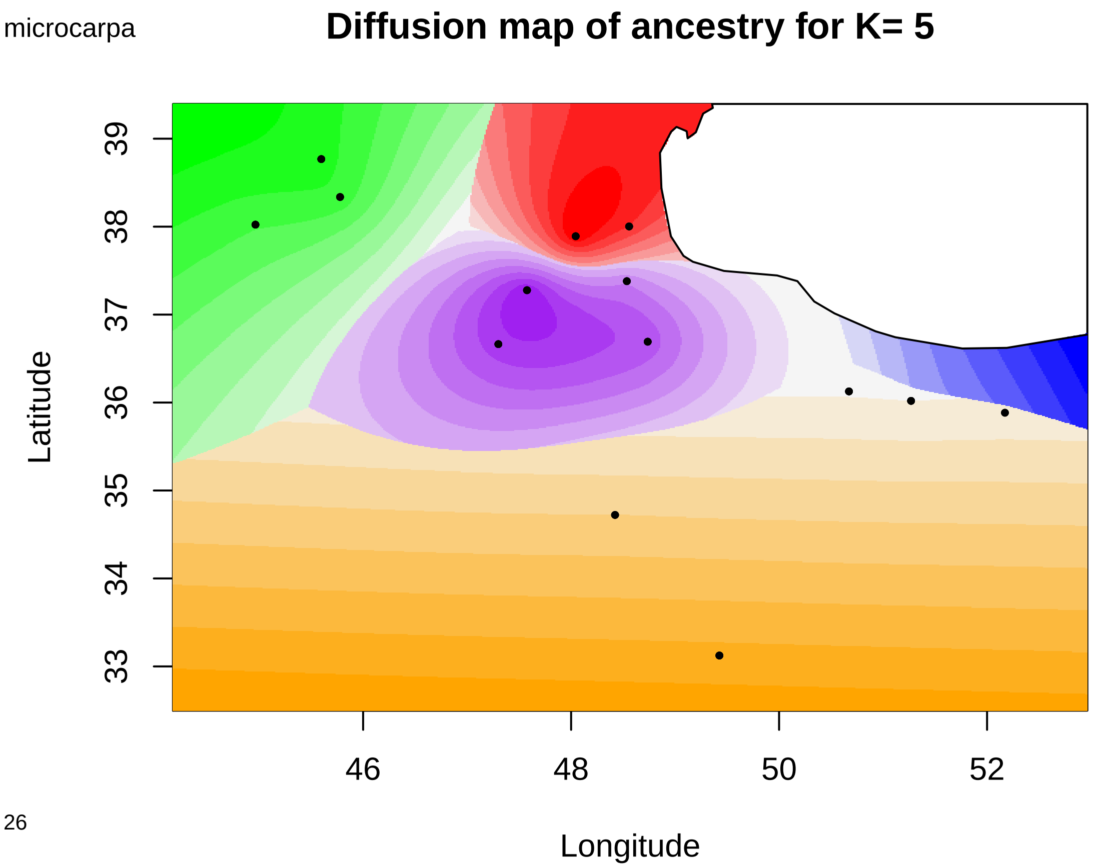

Onosma microcarpa

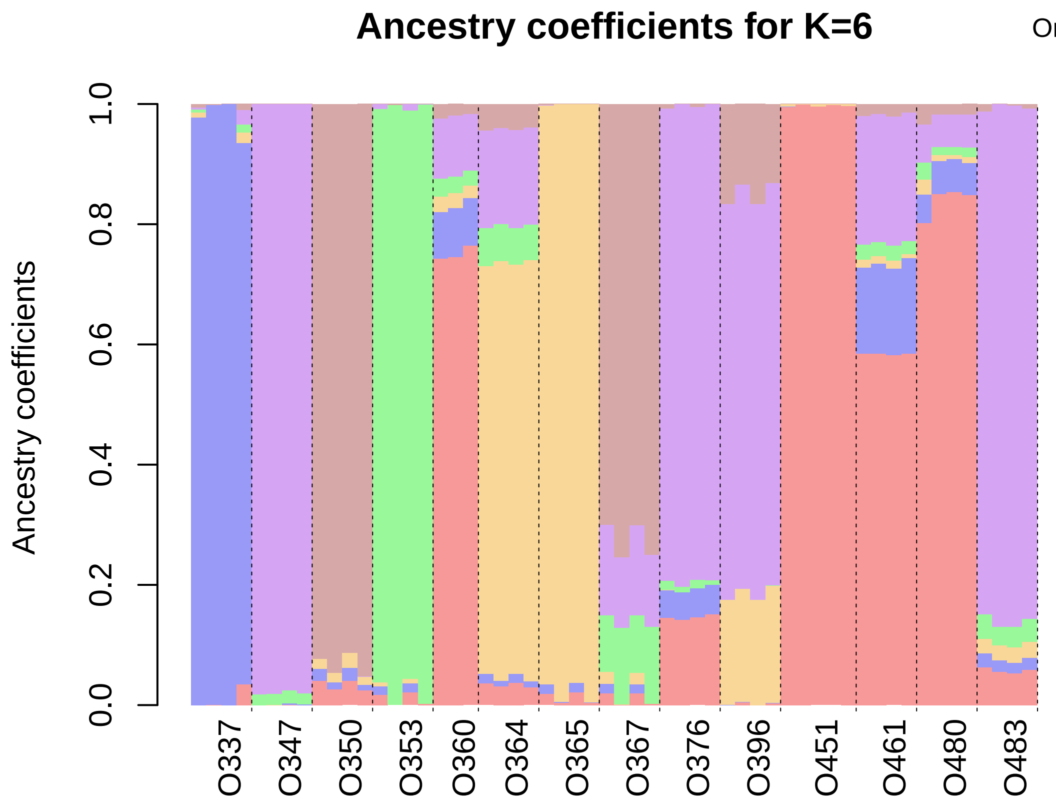

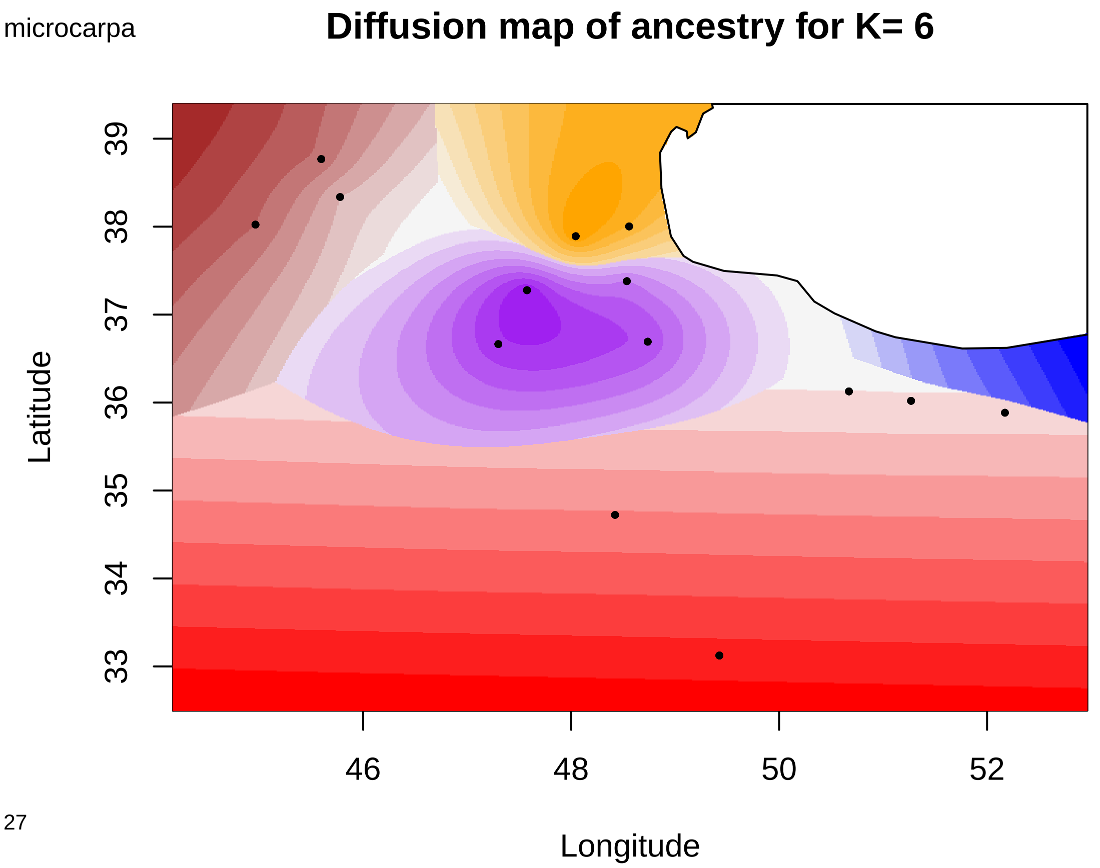

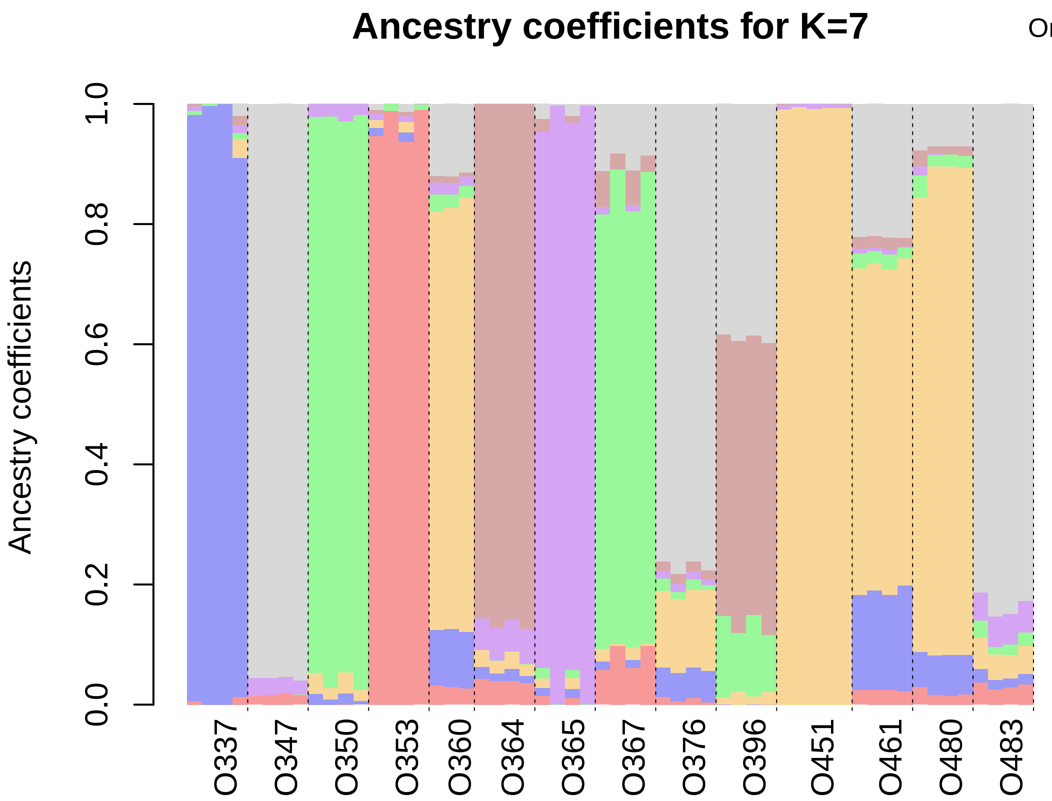

Onosma microcarpa

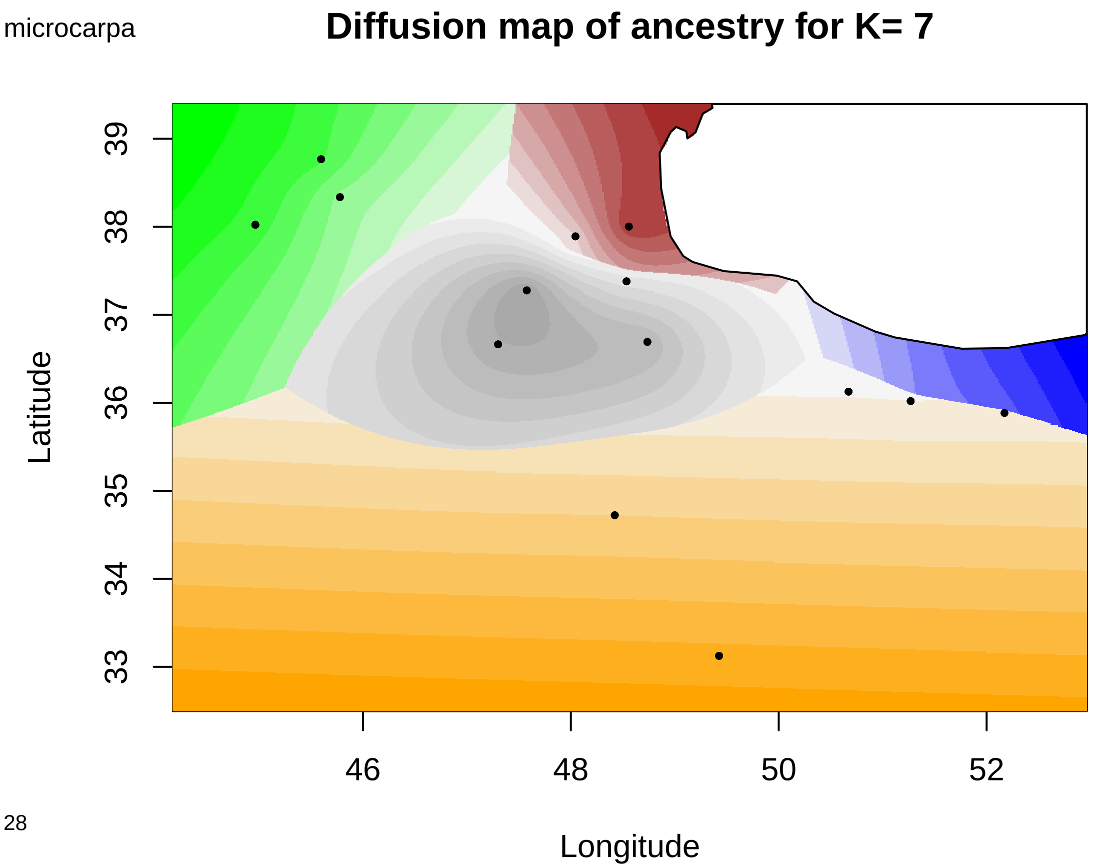

Onosma microcarpa

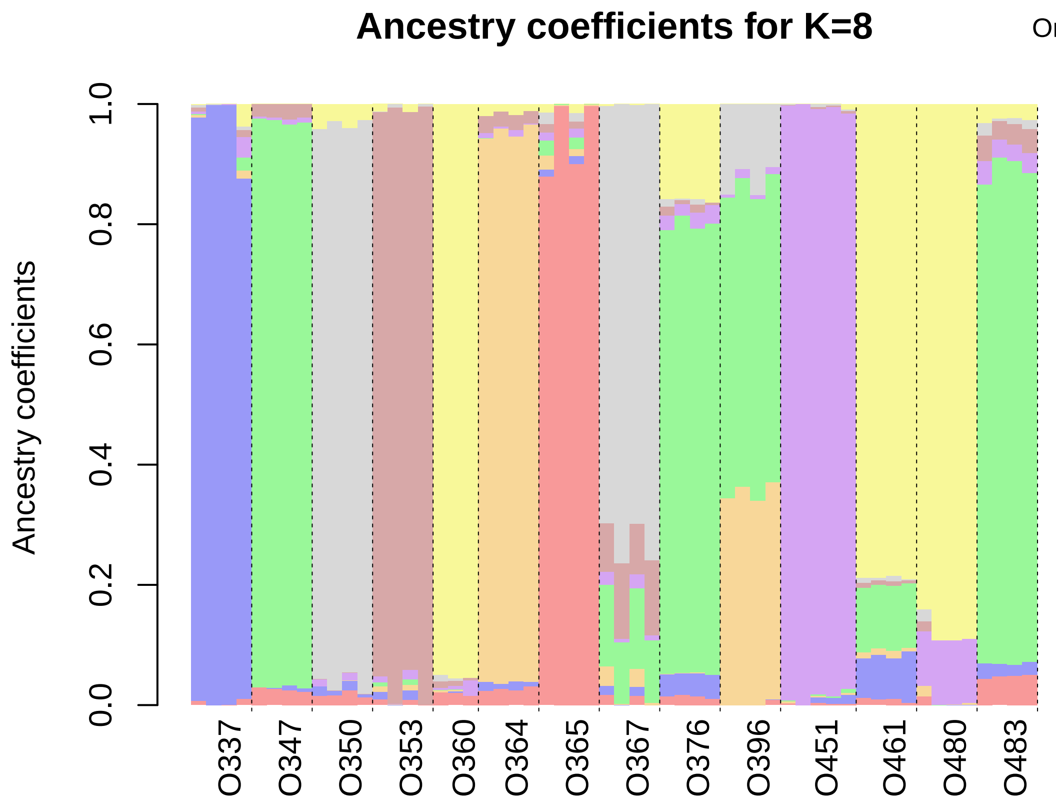

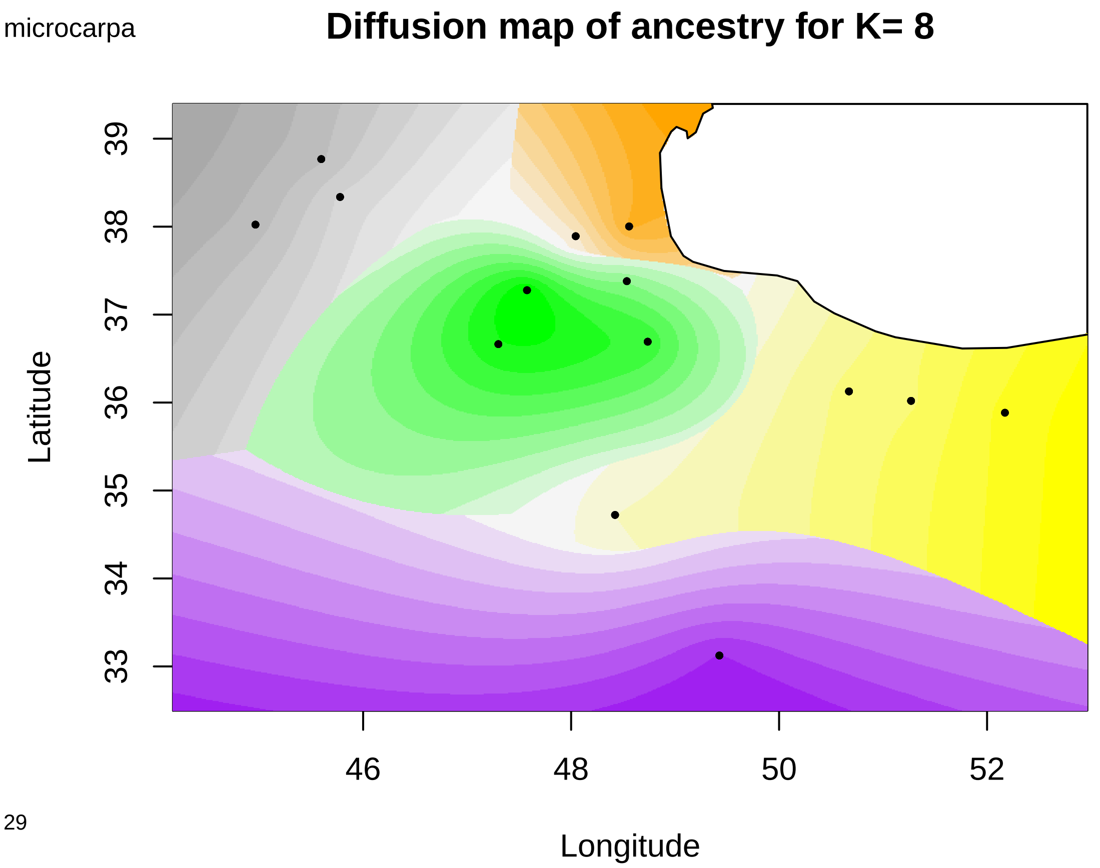

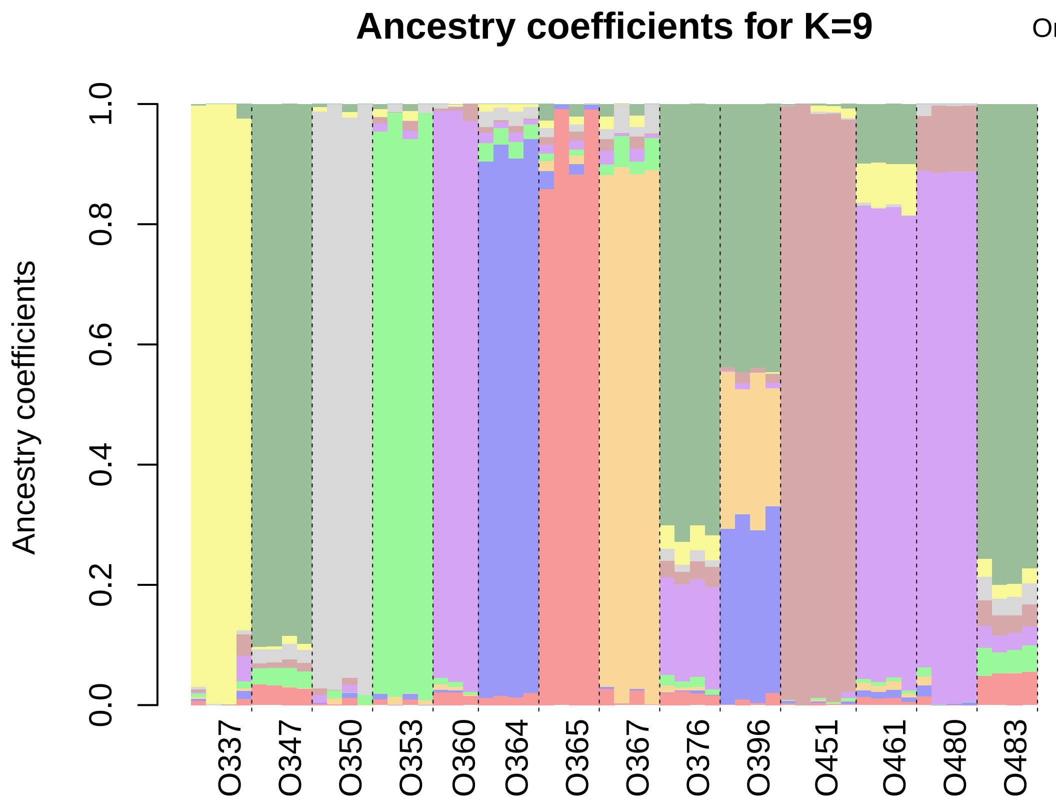

Onosma microcarpa

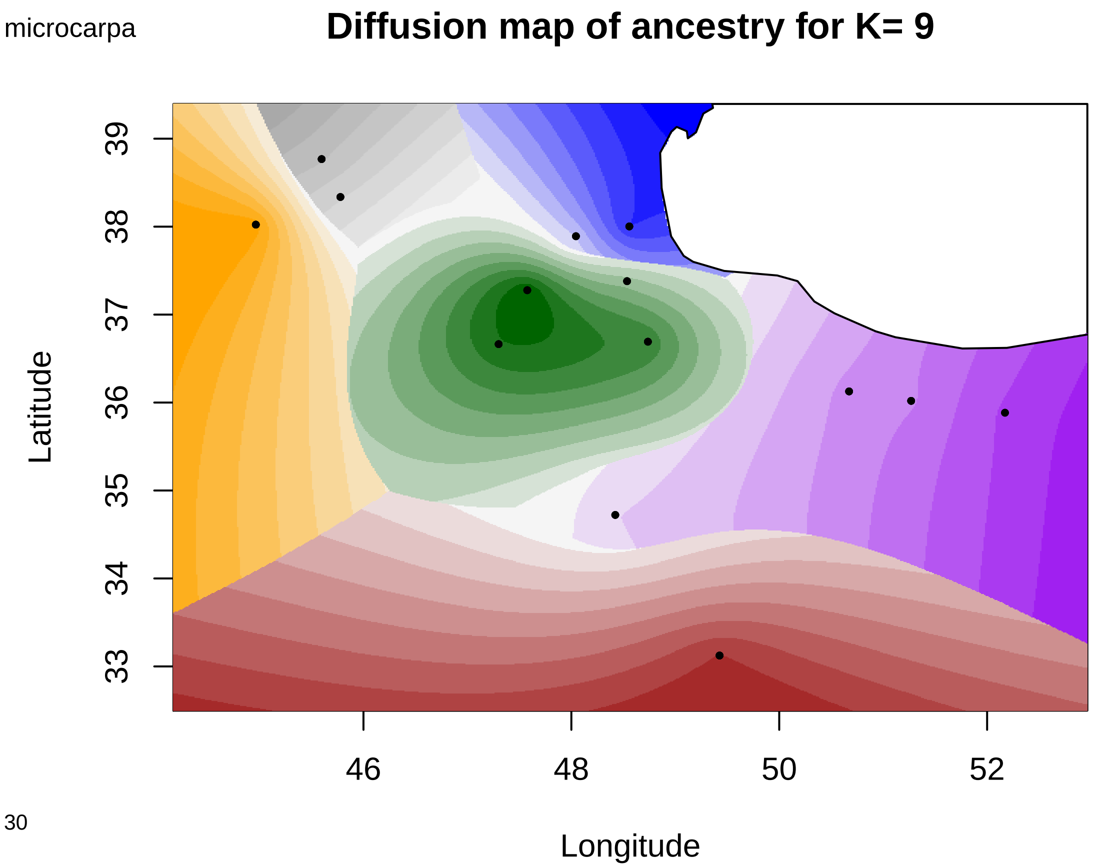

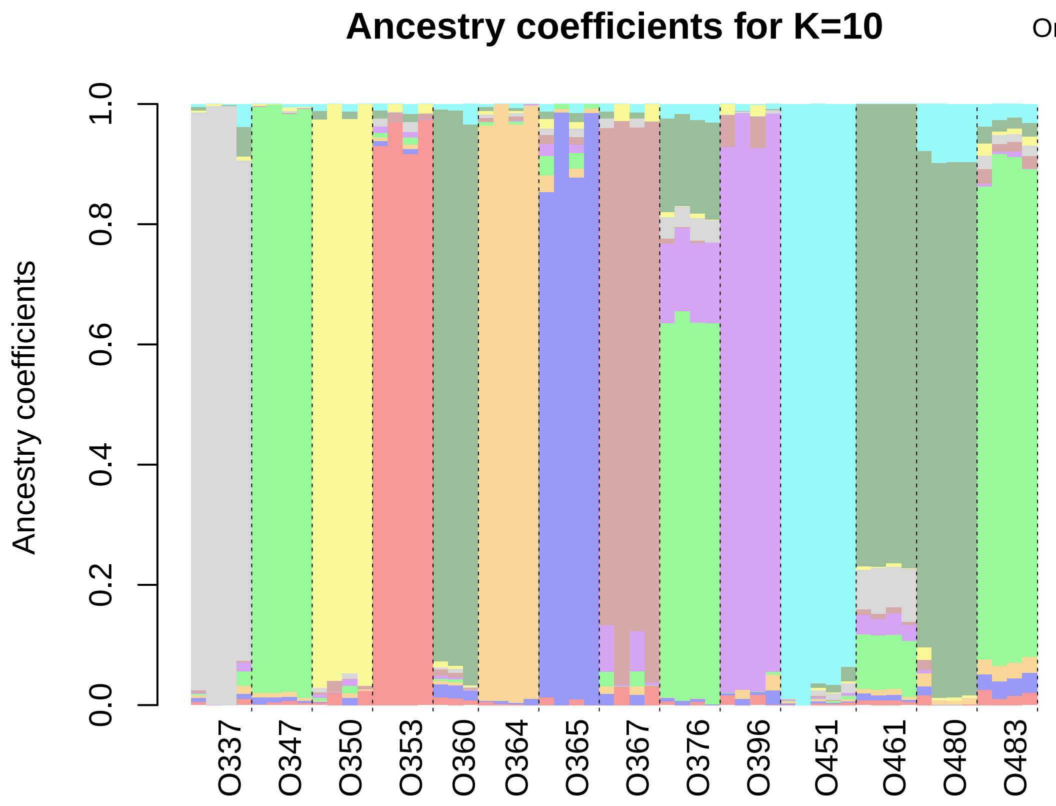

Onosma microcarpa

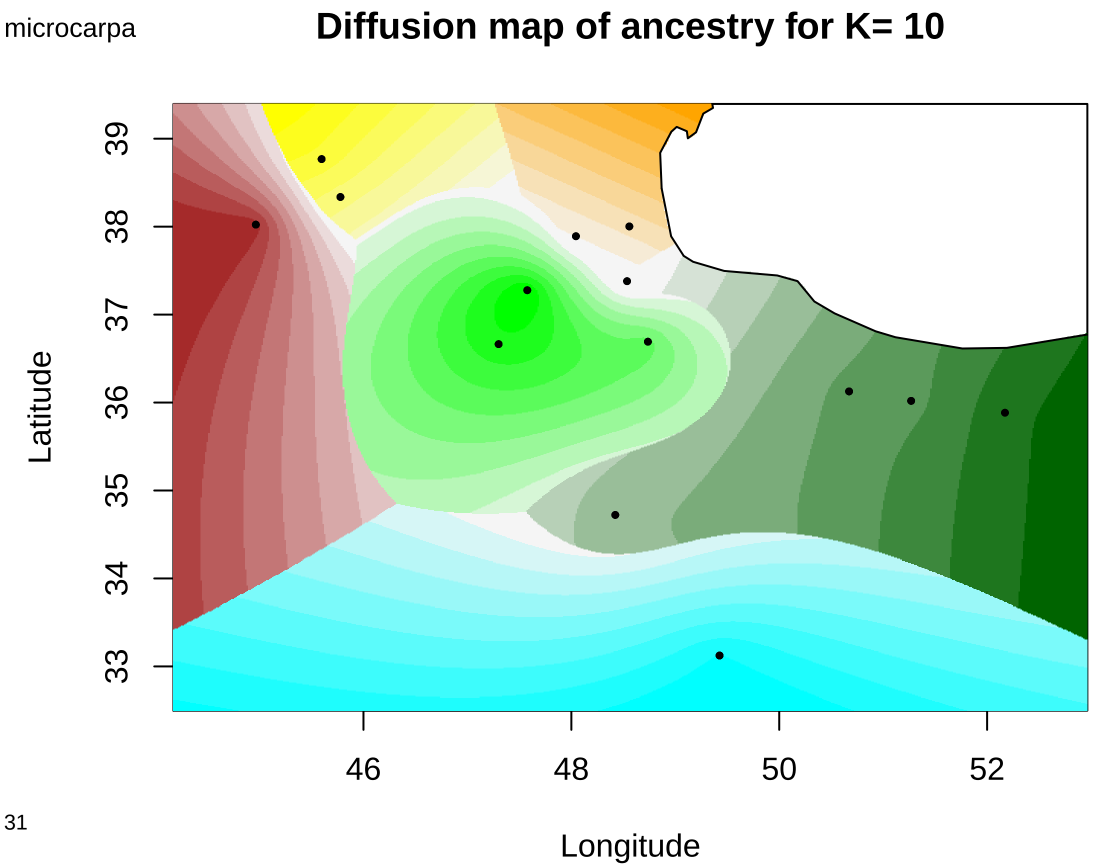

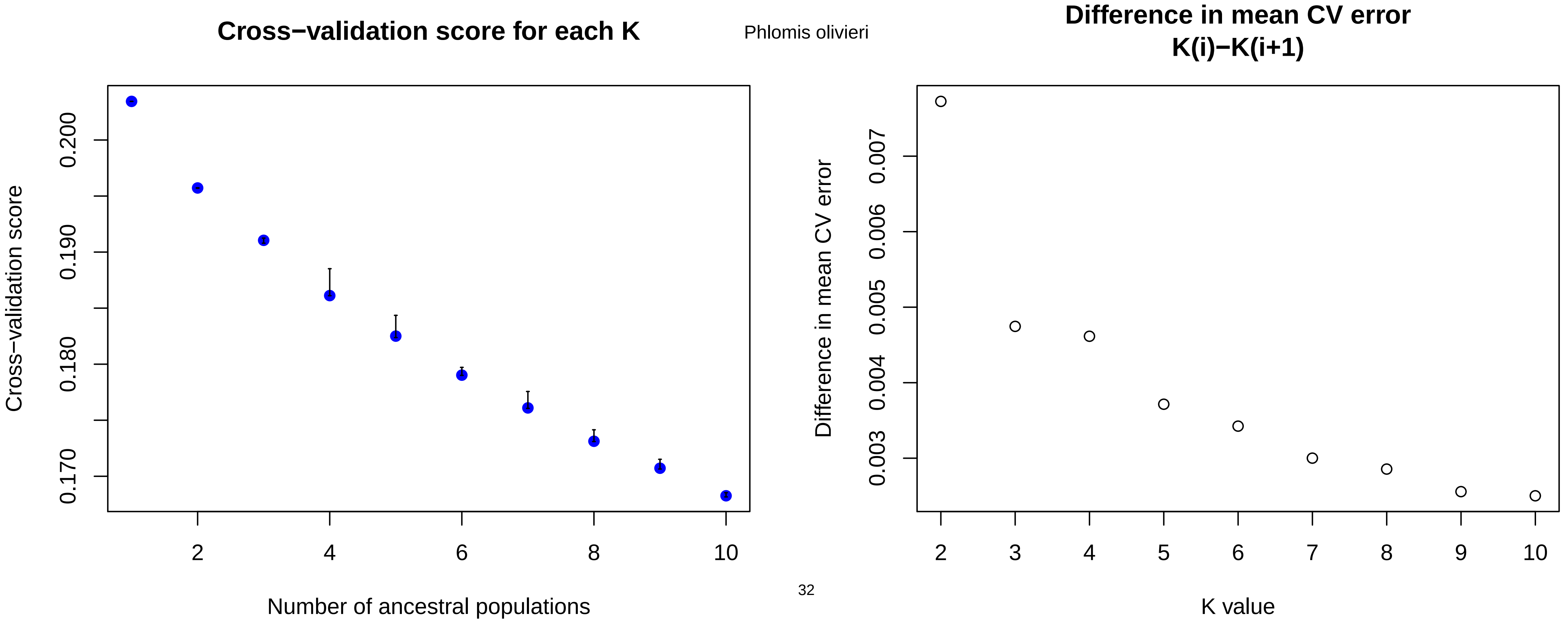
Cross−validation score for each K
Phlomis olivieri
Difference in mean CV error
K(i)−K(i+1)
Cross−validation score
0.170
0.180
0.190
0.200
2
4
6
8
10
Number of ancestral populations
Difference in mean CV error
0.003
0.004
0.005
0.006
0.007
2
3
4
5
6
7
8
9
10
K value

Phlomis olivieri

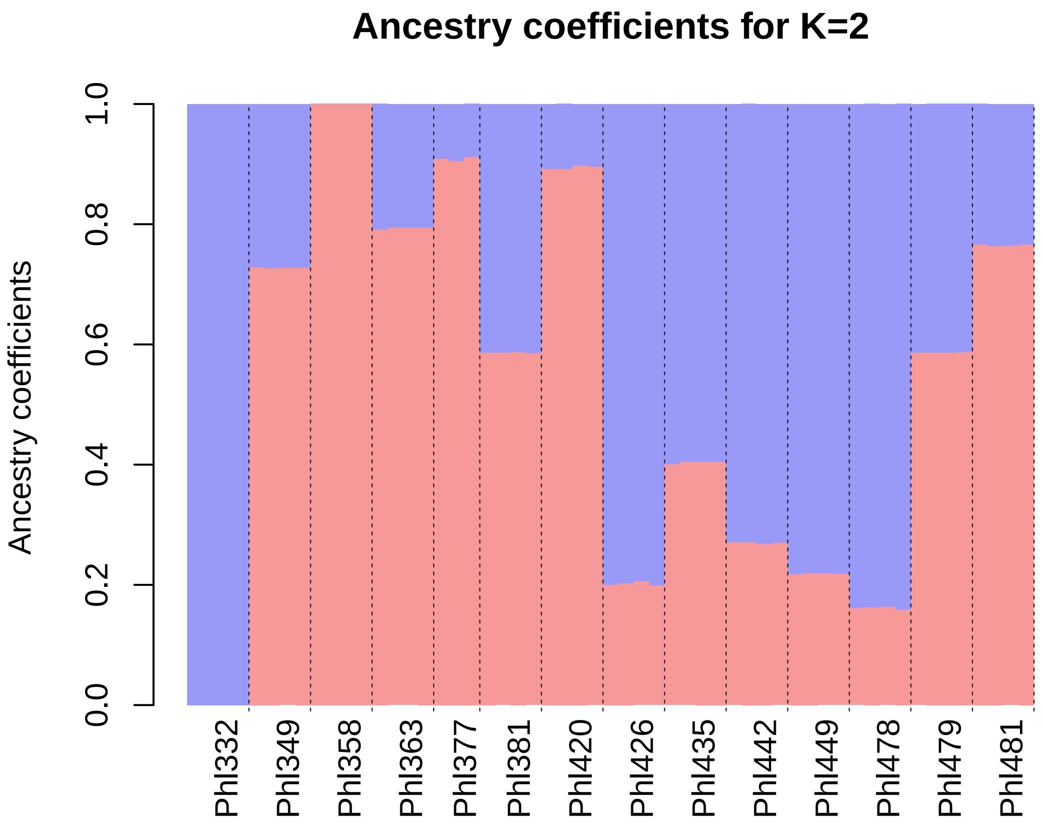

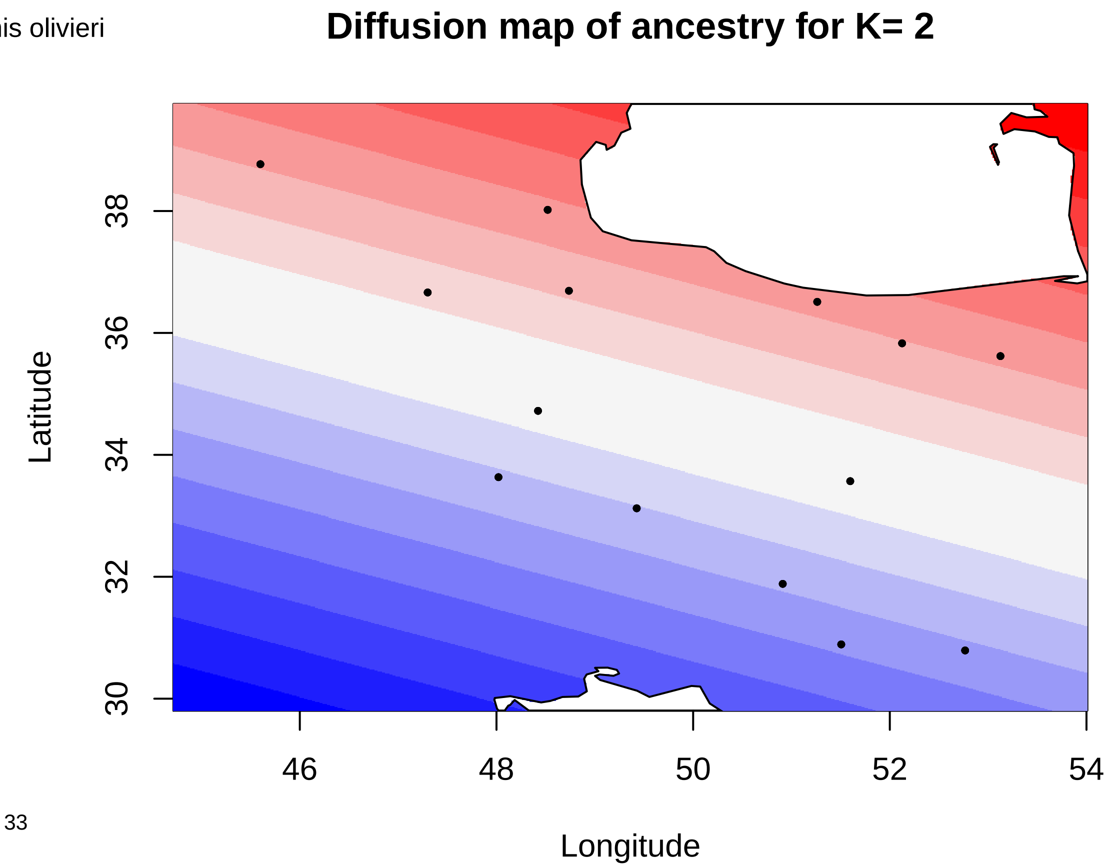

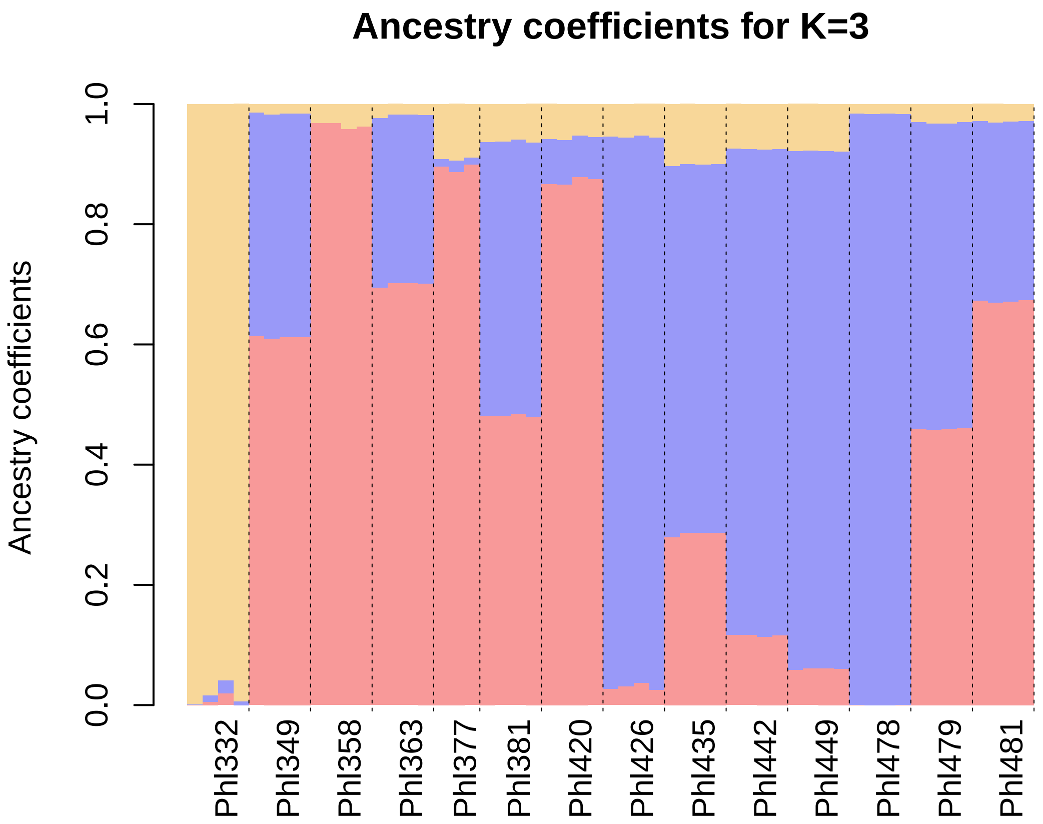

Phlomis olivieri

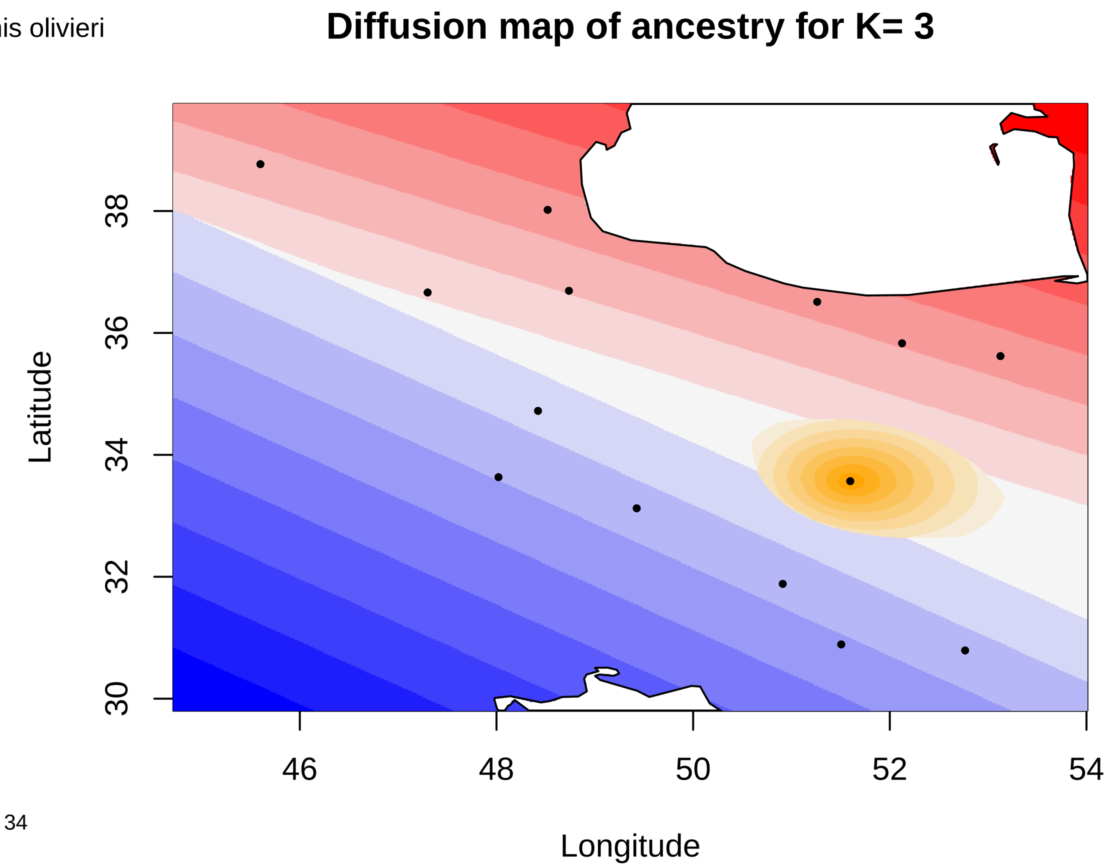

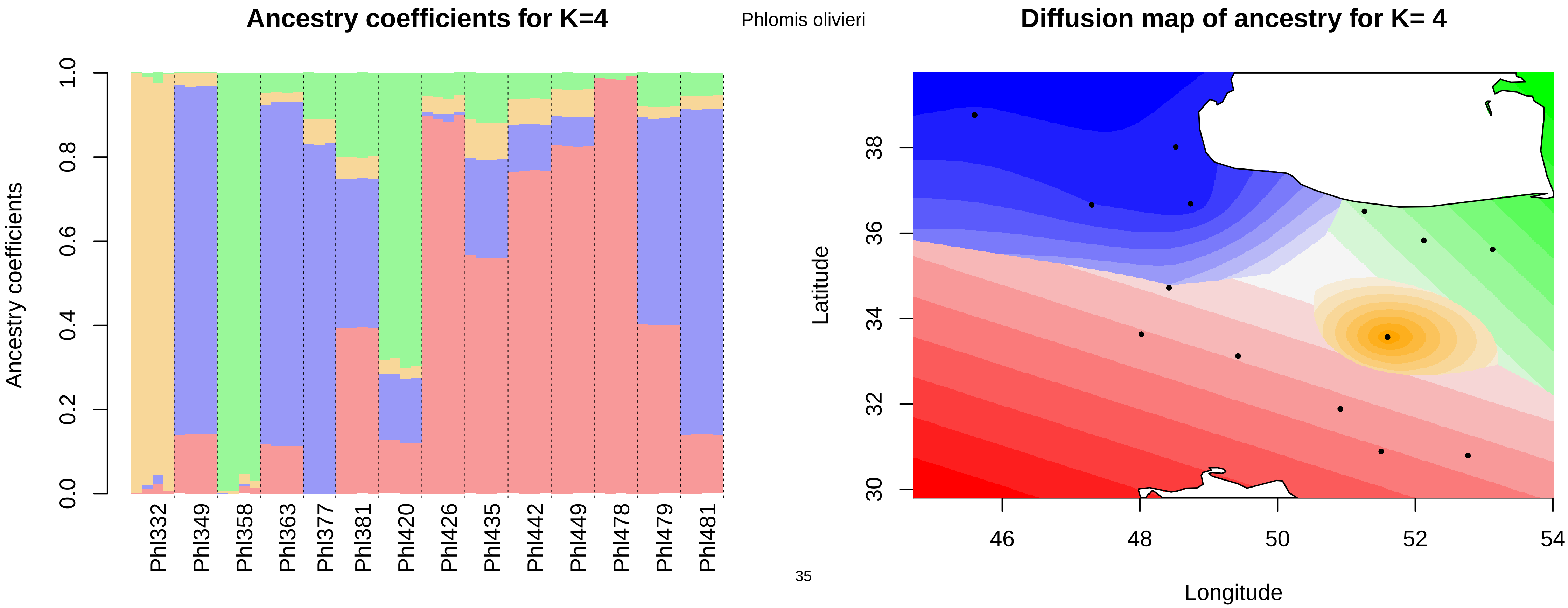
Ancestry coefficients for K=4
Phlomis olivieri
Diffusion map of ancestry for K= 4
Ancestry coefficients
0.0
0.2
0.4
0.6
0.8
1.0
Phl332
Phl349
Phl358
Phl363
Phl377
Phl381
Phl420
Phl426
Phl435
Phl442
Phl449
Phl478
Phl479
Phl481
Latitude
30
32
34
36
38
Longitude
46
48
50
52
54

Phlomis olivieri

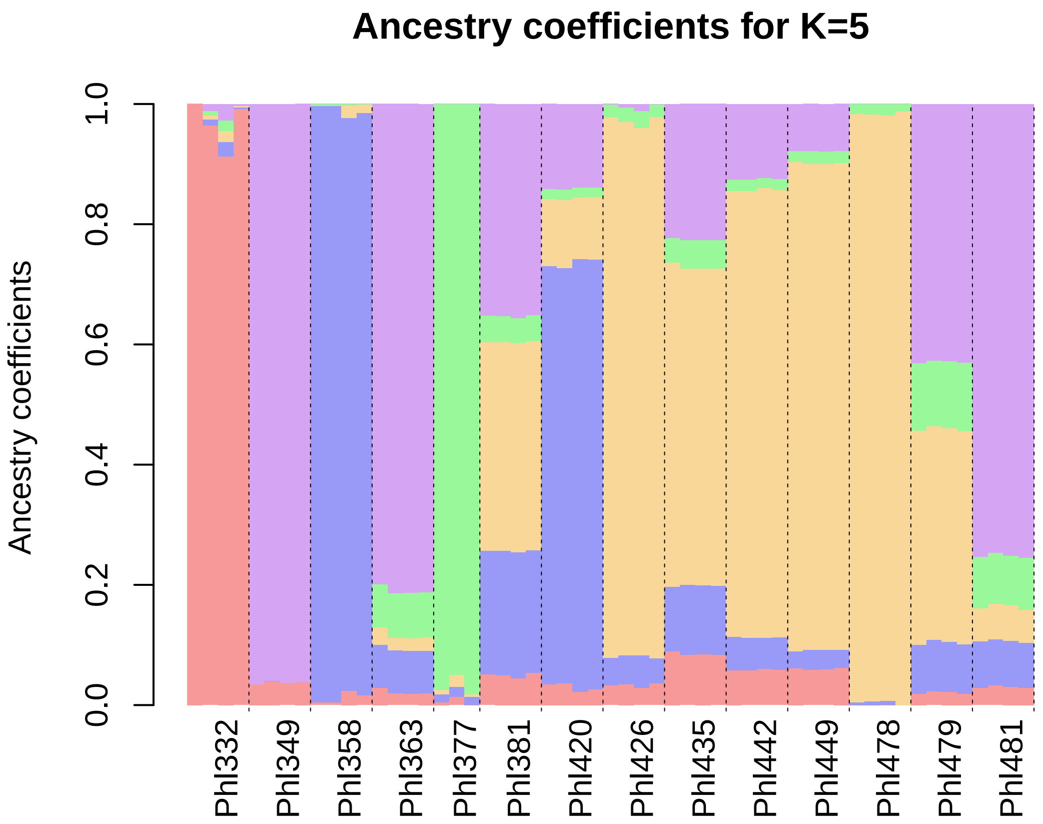

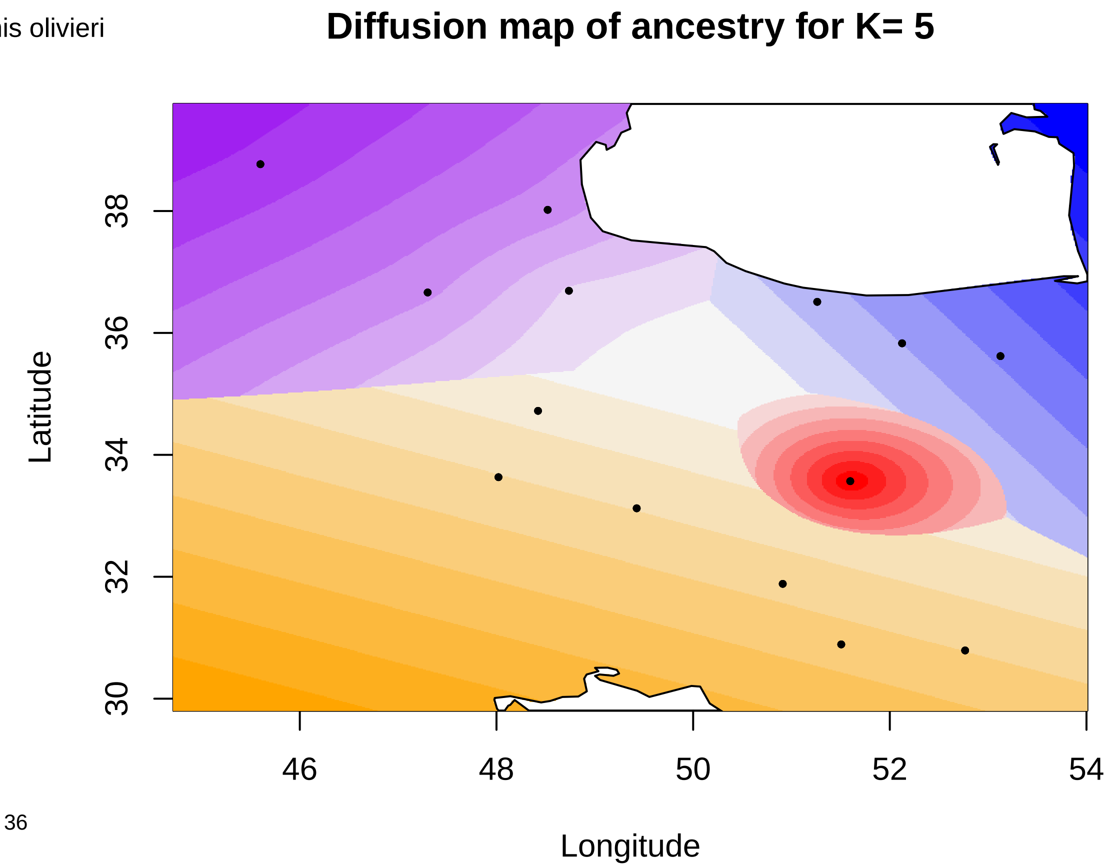

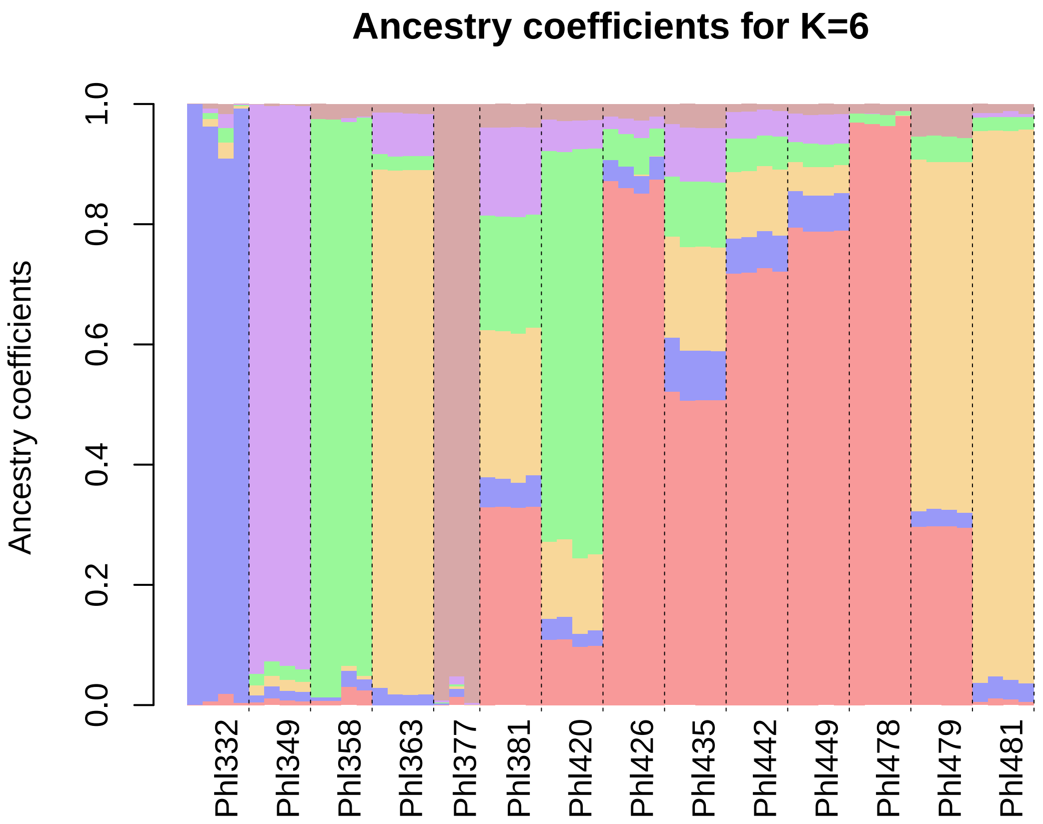

Phlomis olivieri

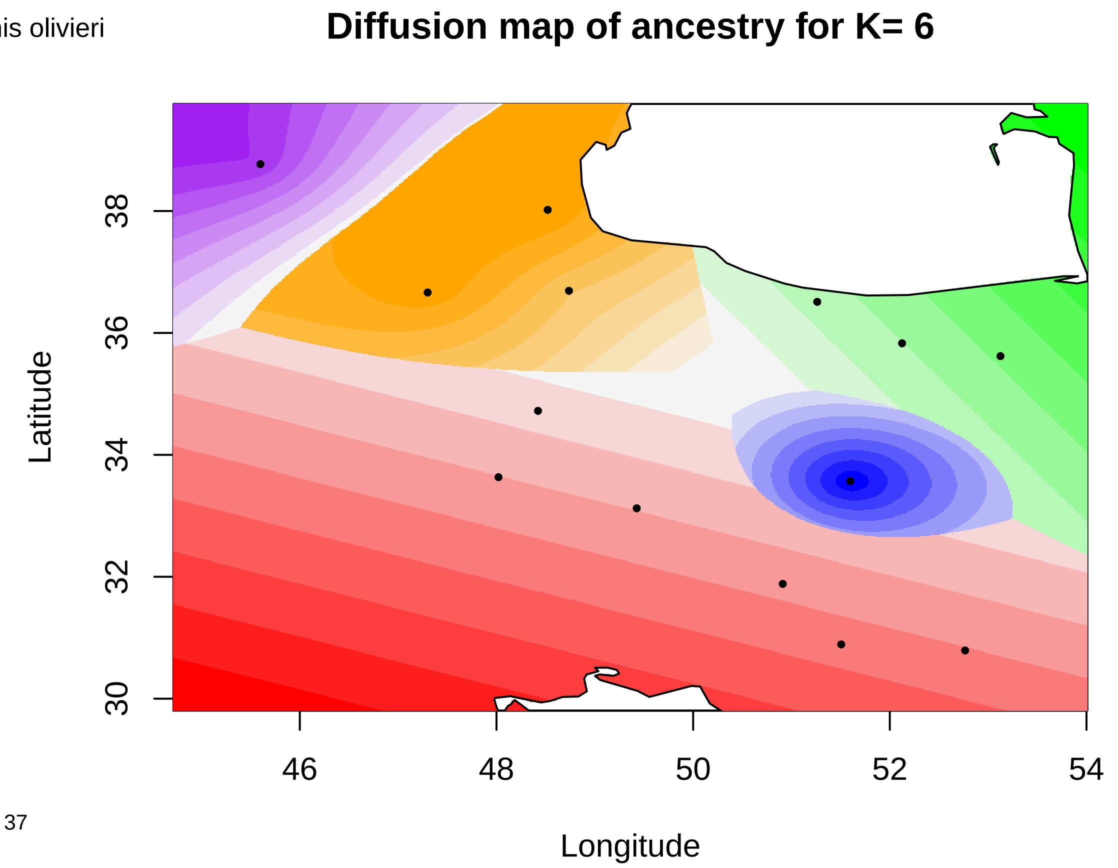

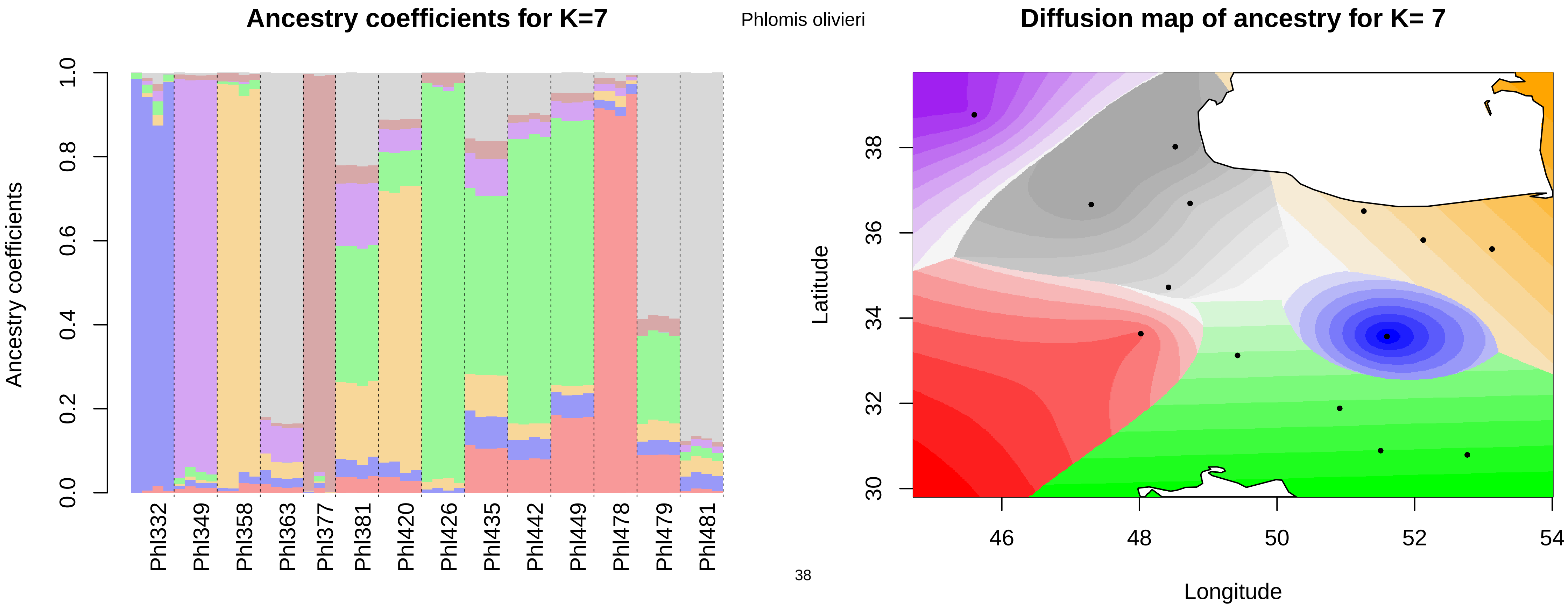
Ancestry coefficients for K=7
Phlomis olivieri
Diffusion map of ancestry for K= 7
Ancestry coefficients
0.0
0.2
0.4
0.6
0.8
1.0
Phl332
Phl349
Phl358
Phl363
Phl377
Phl381
Phl420
Phl426
Phl435
Phl442
Phl449
Phl478
Phl479
Phl481
Latitude
30
32
34
36
38
46
48
50
52
54
Longitude

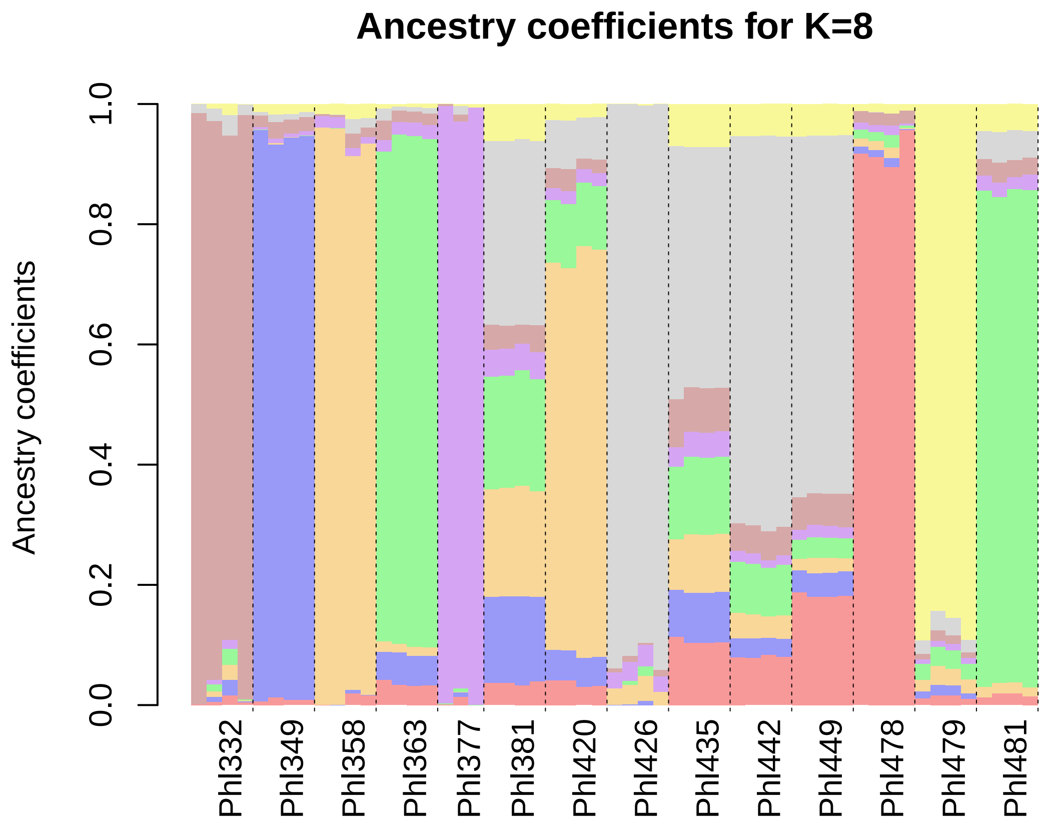

Phlomis olivieri

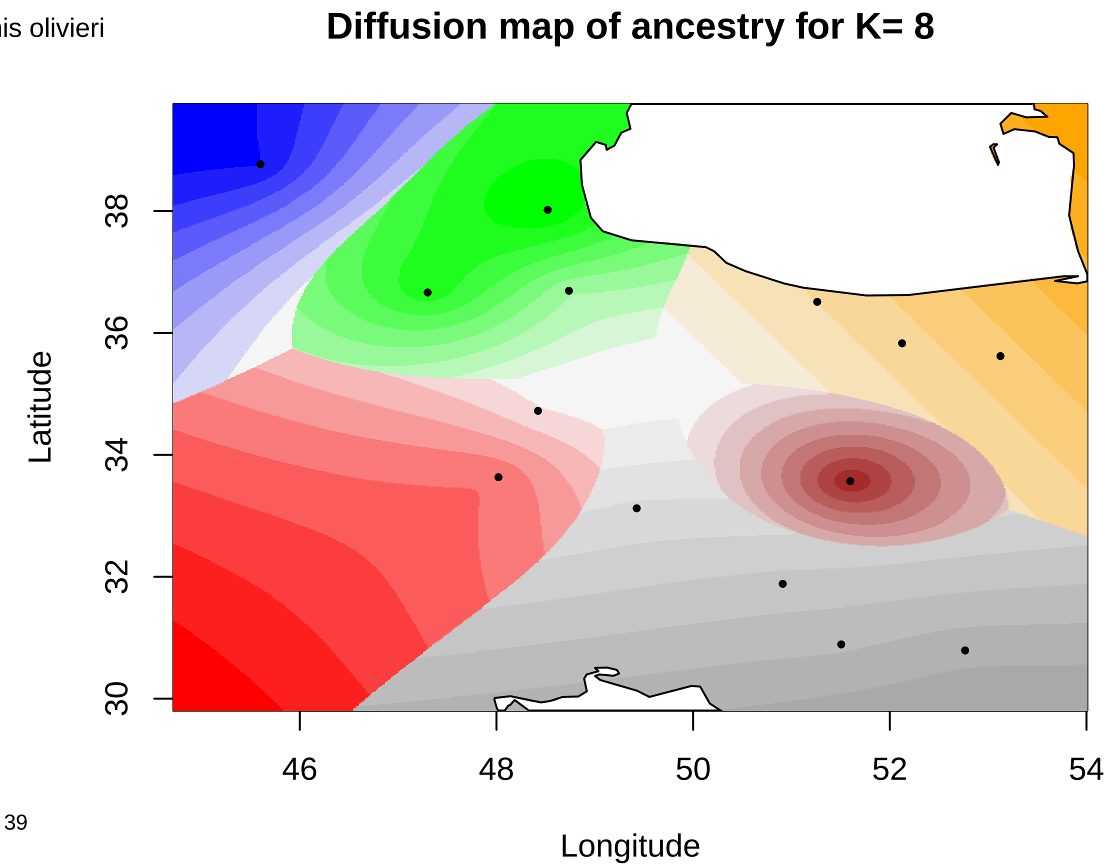

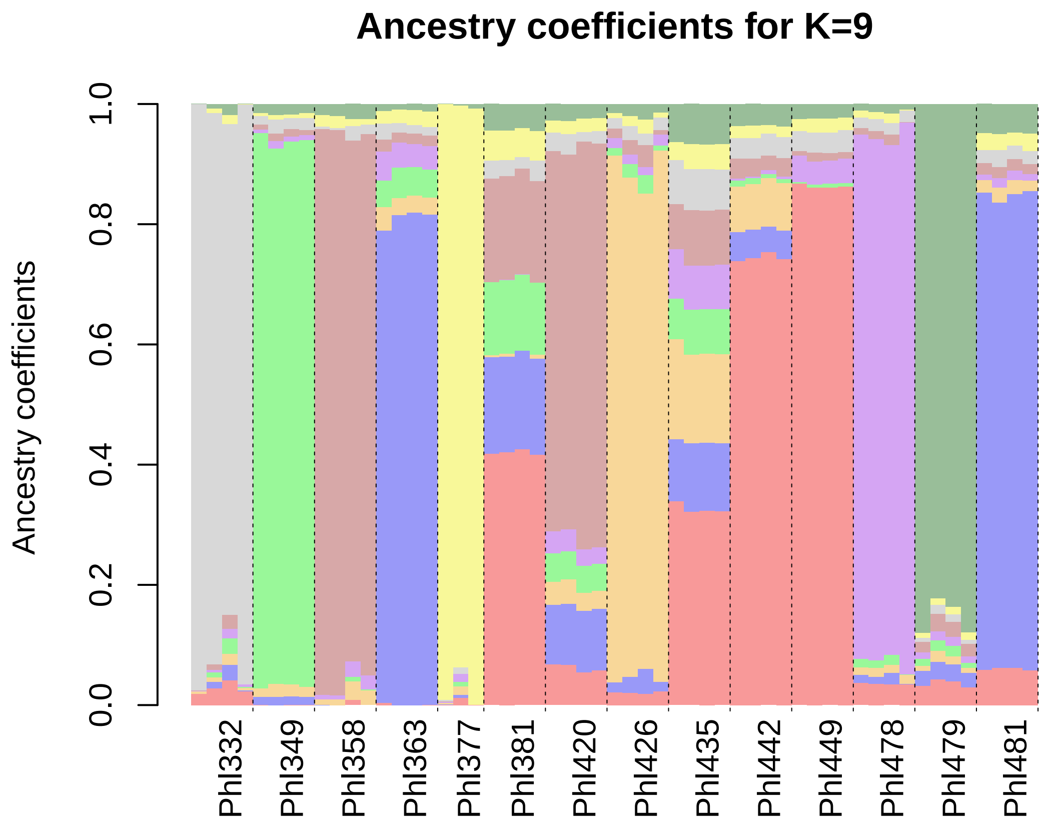

Phlomis olivieri

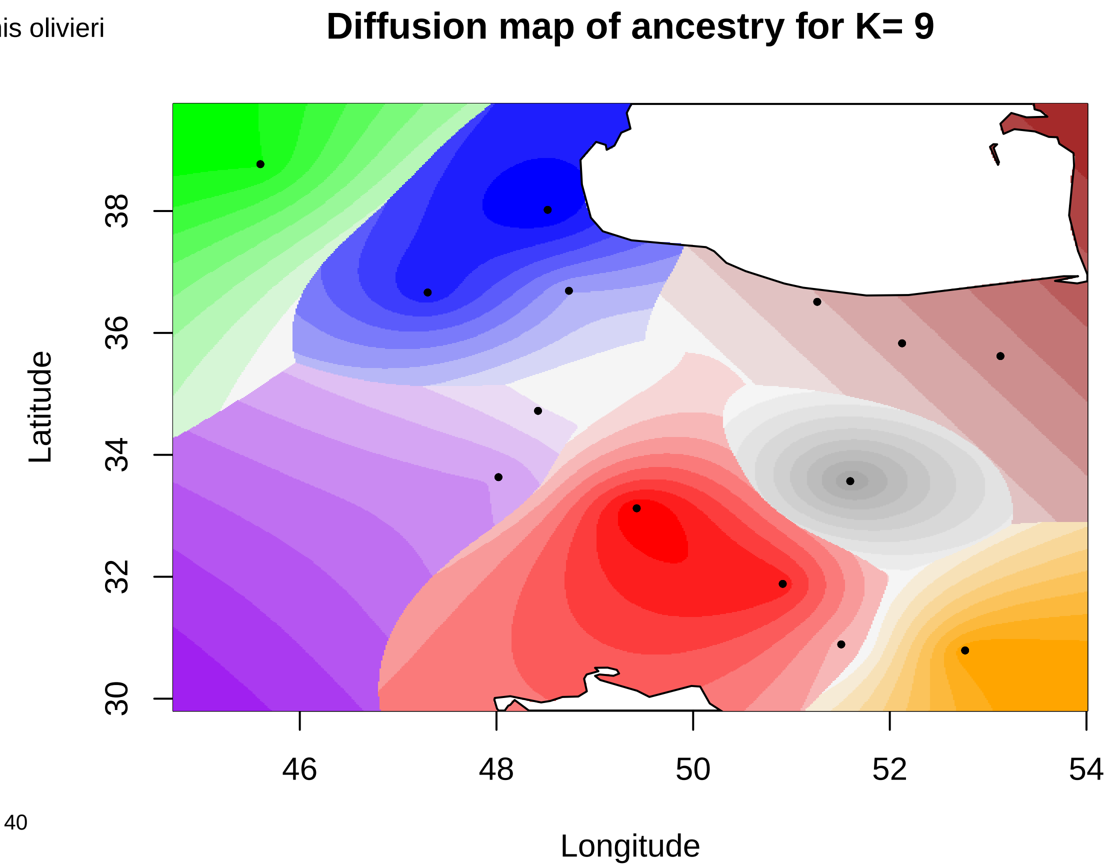

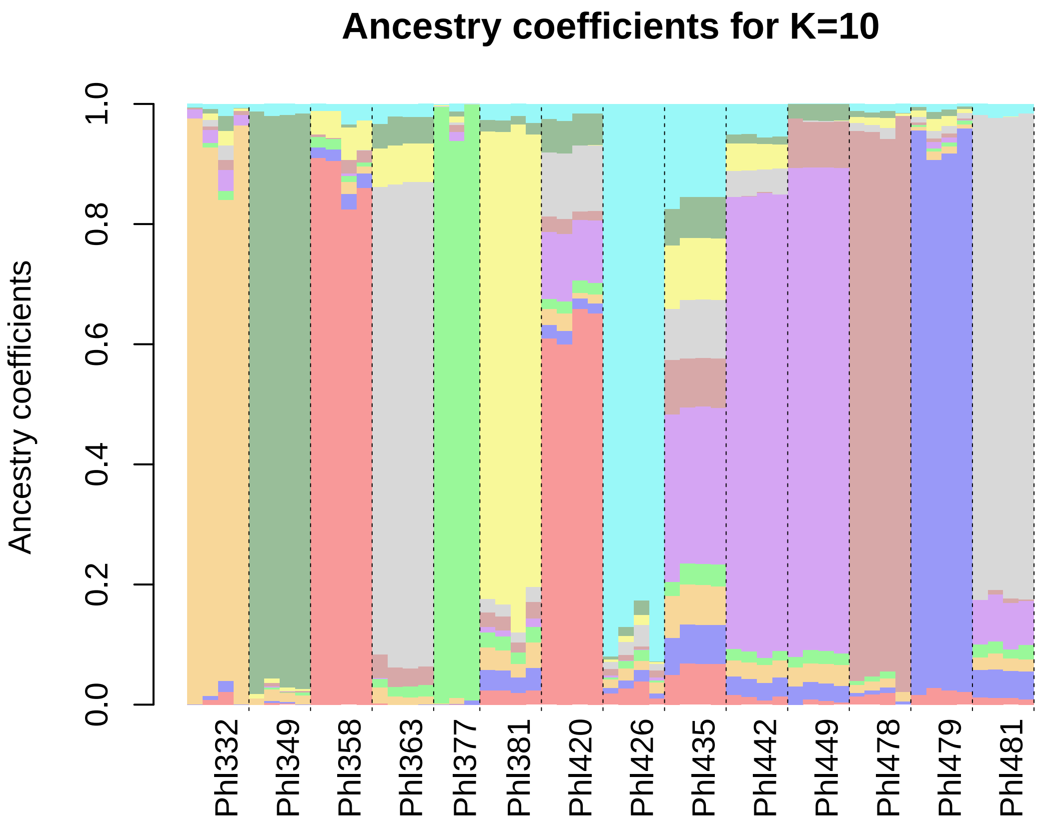

Phlomis olivieri

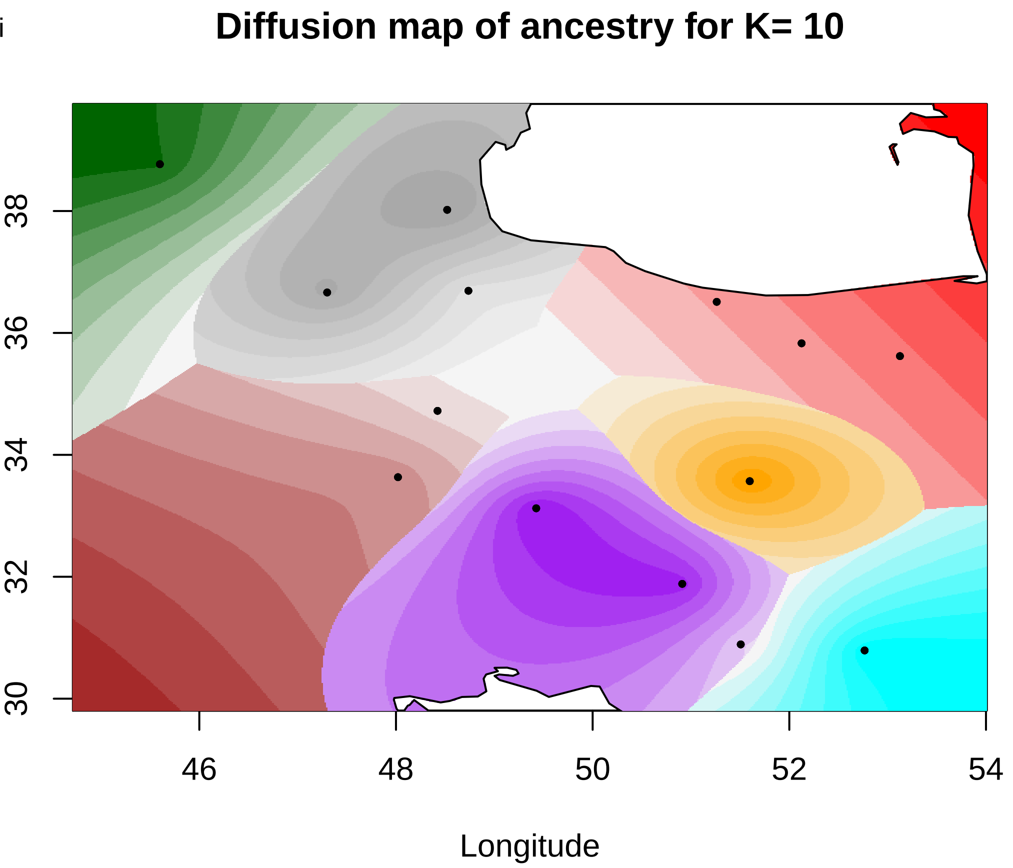

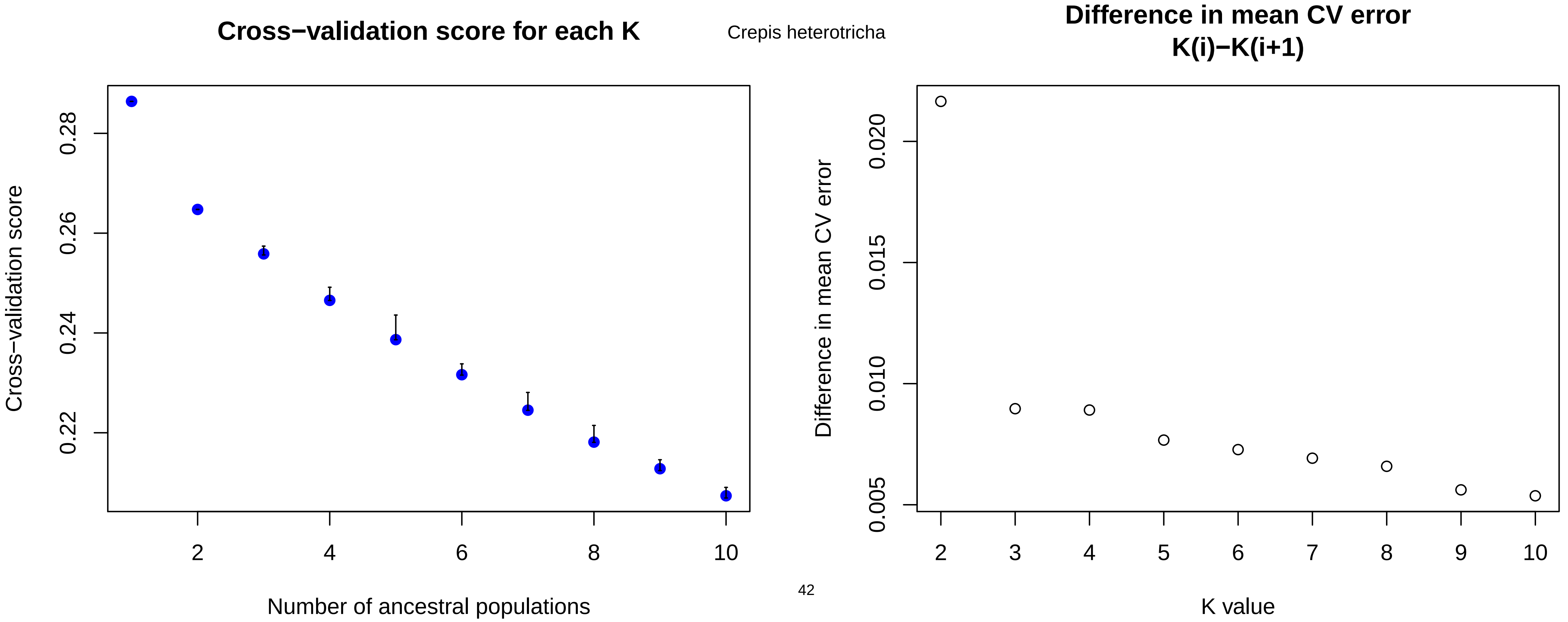
Cross−validation score for each K
Crepis heterotricha
Difference in mean CV error
K(i)−K(i+1)
Cross−validation score
0.22
0.24
0.26
0.28
2
4
6
8
10
Number of ancestral populations
Difference in mean CV error
0.005
0.010
0.015
0.020
2
3
4
5
6
7
8
9
10
K value

Crepis heterotricha

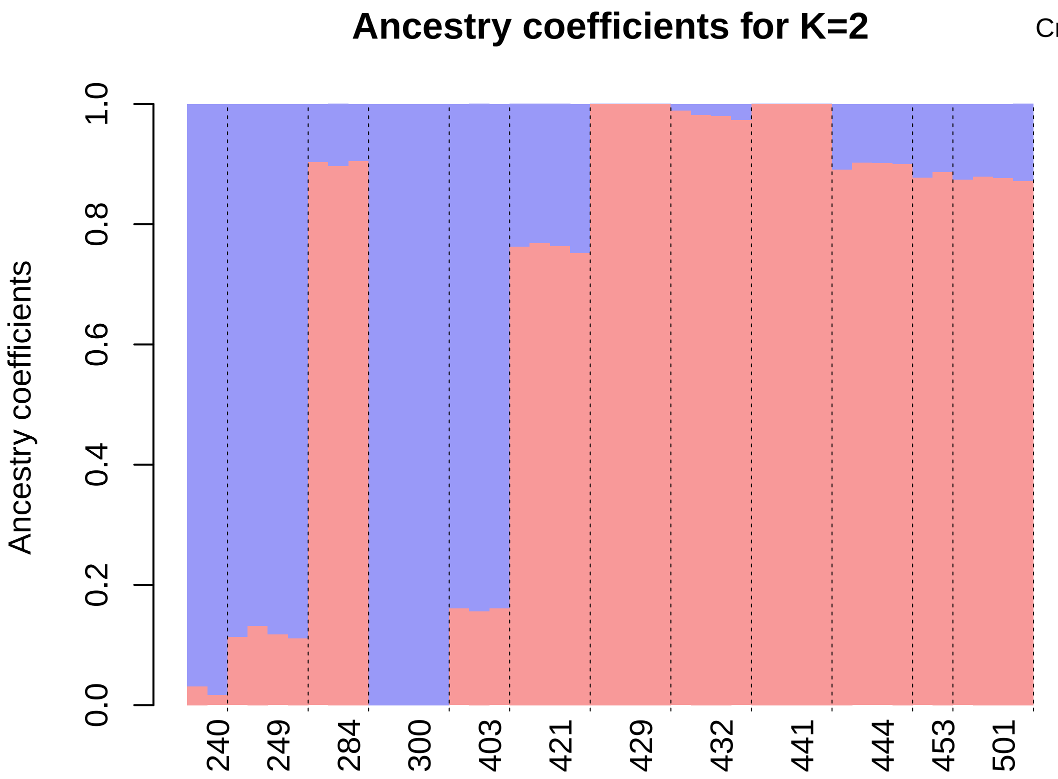

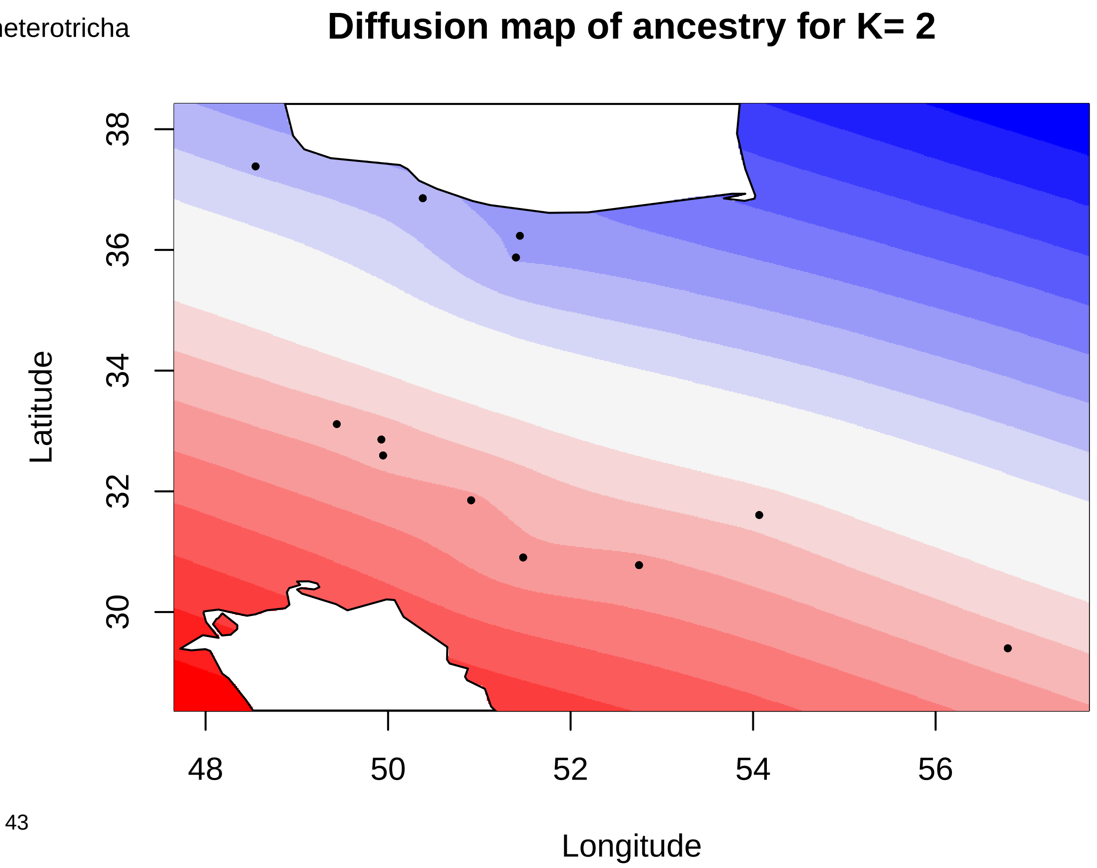

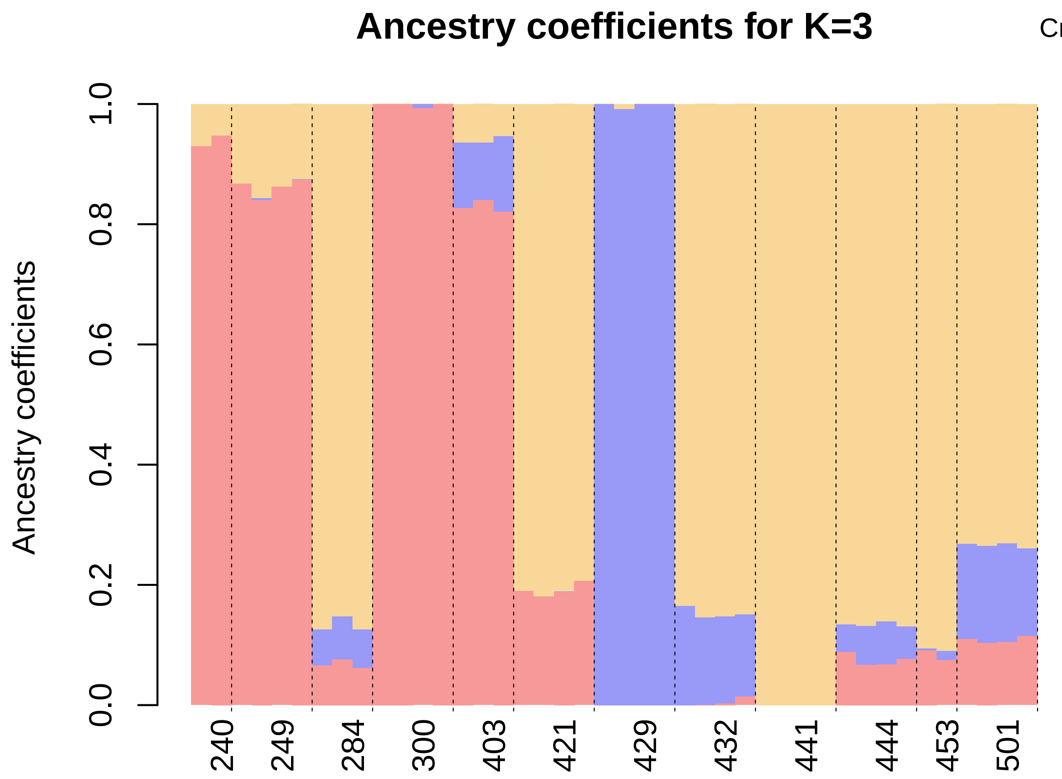

Crepis heterotricha

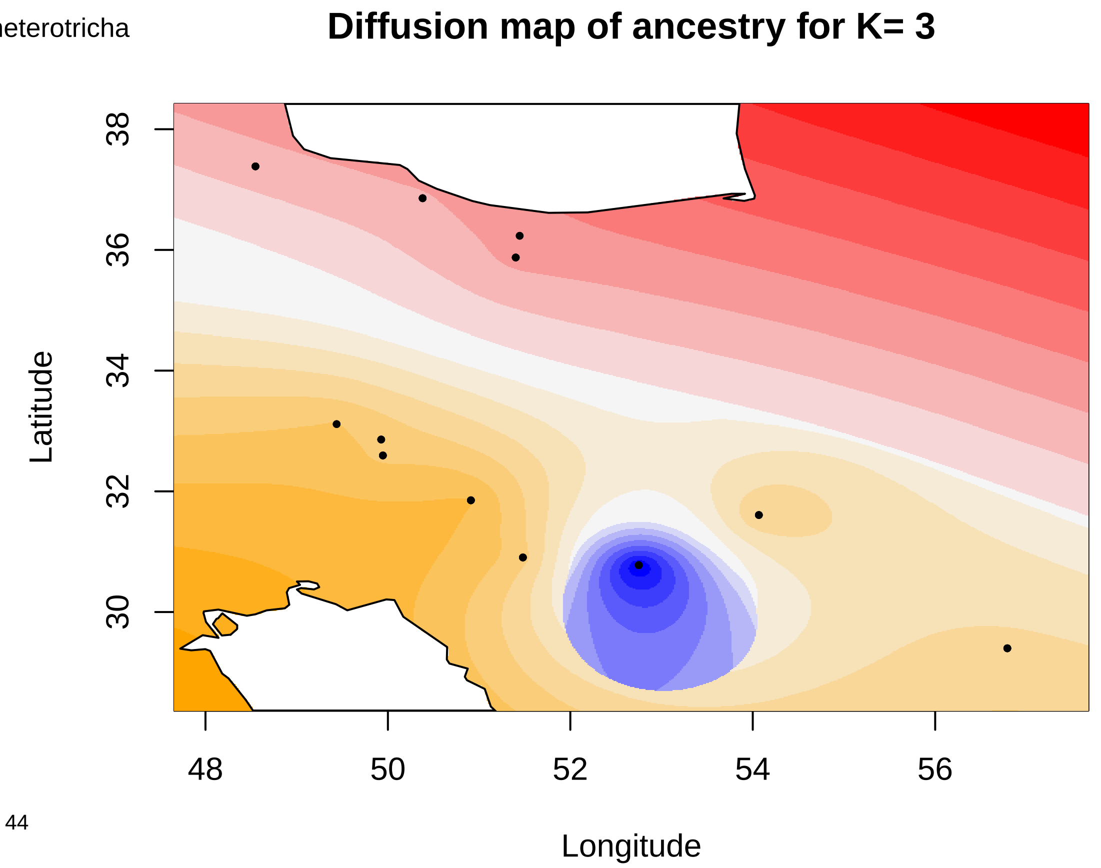

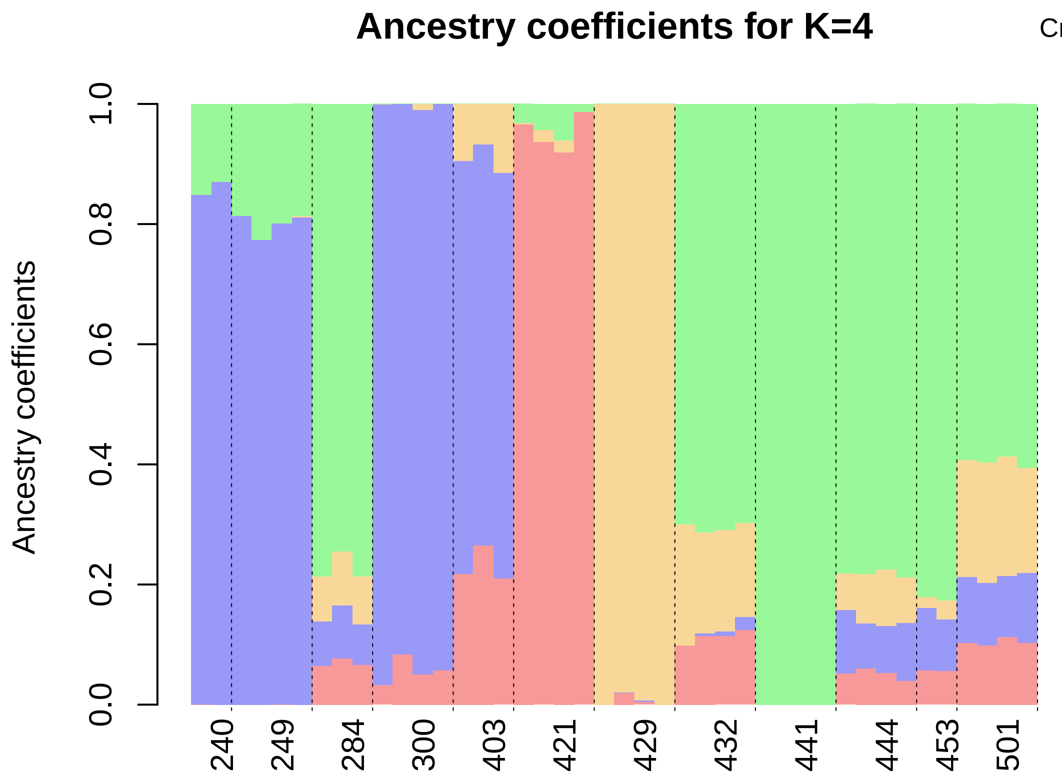

Crepis heterotricha

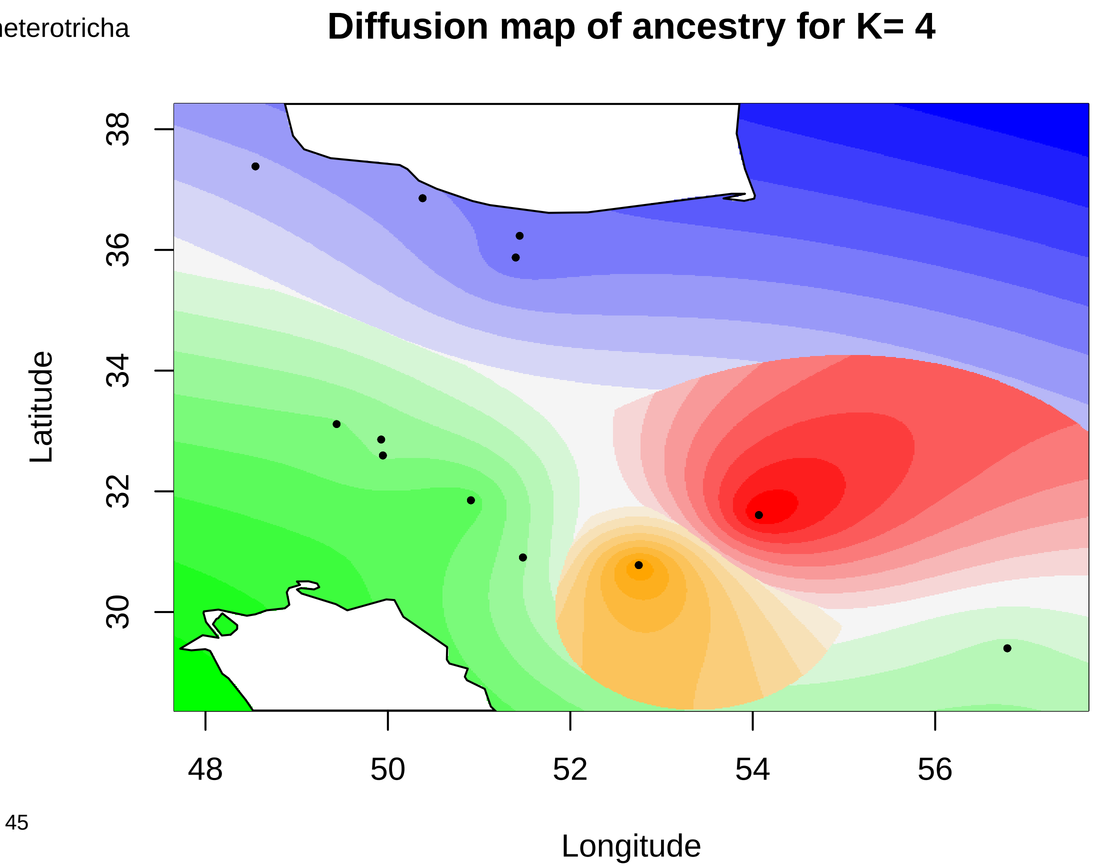

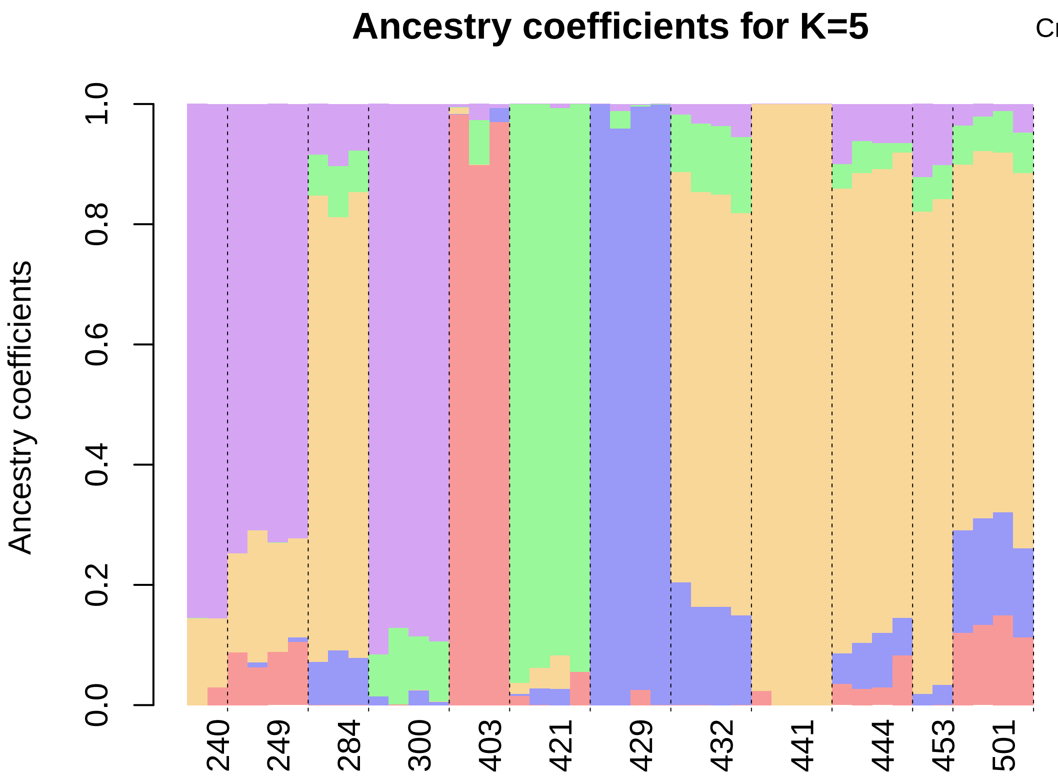

Crepis heterotricha

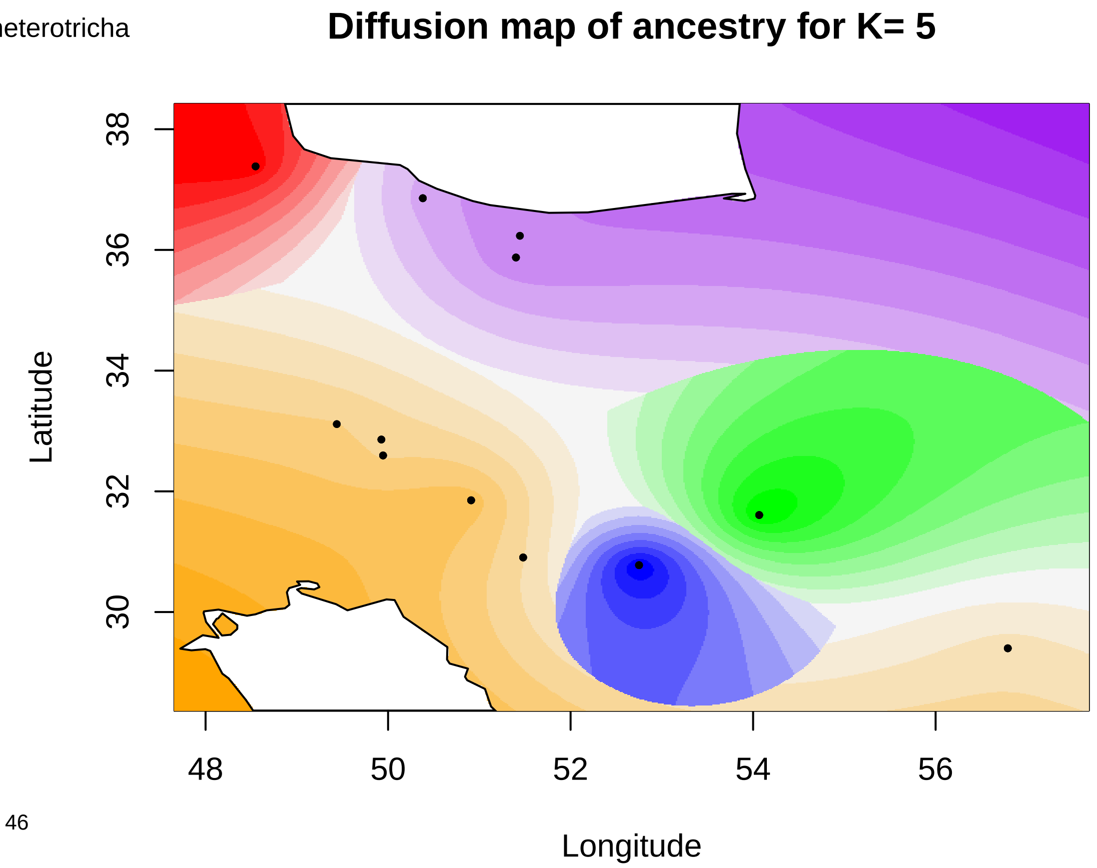

Crepis heterotricha

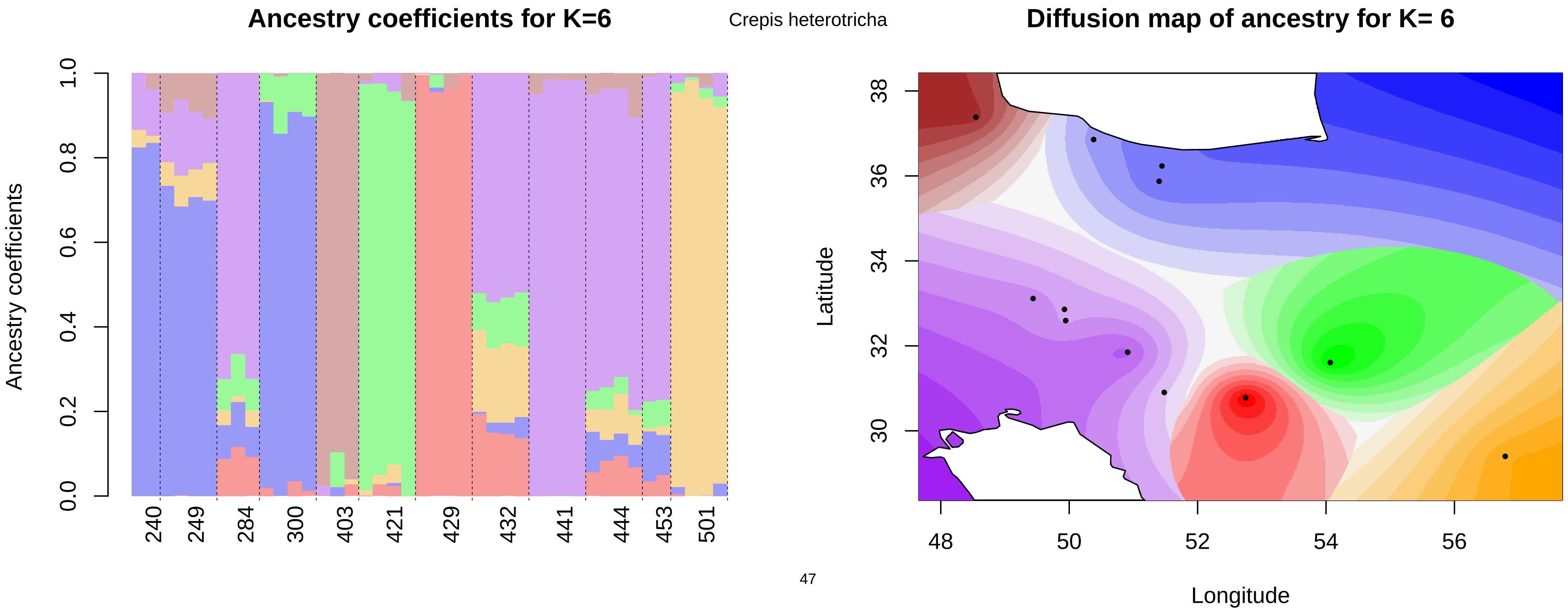

Crepis heterotricha

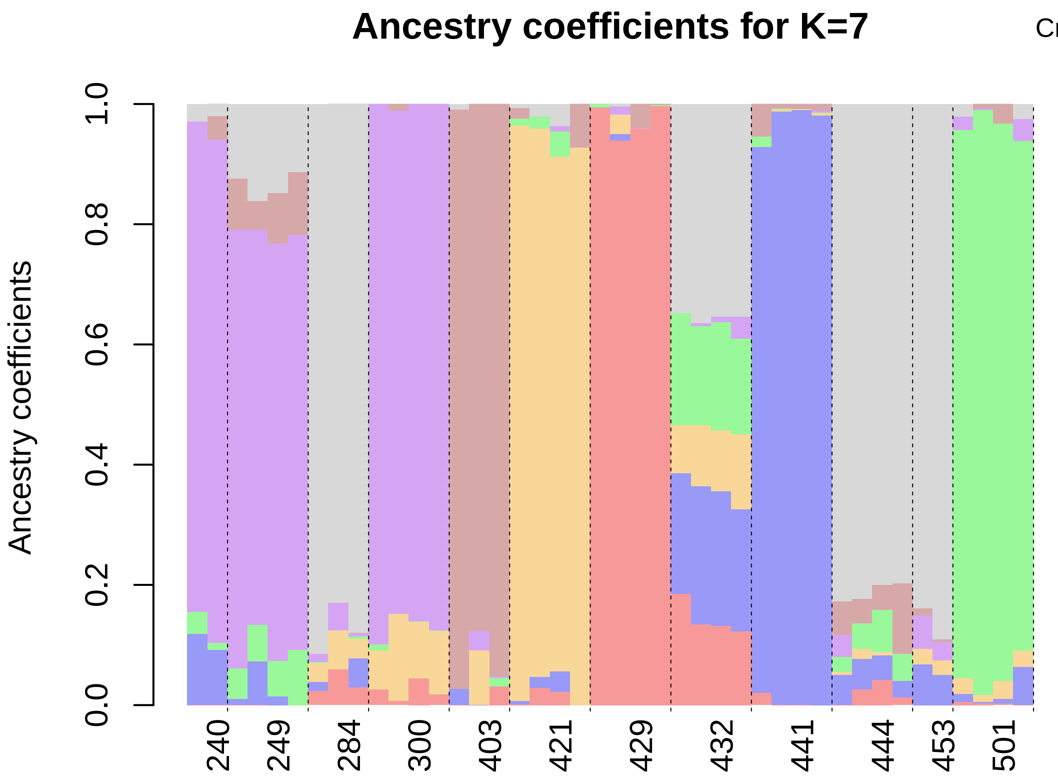

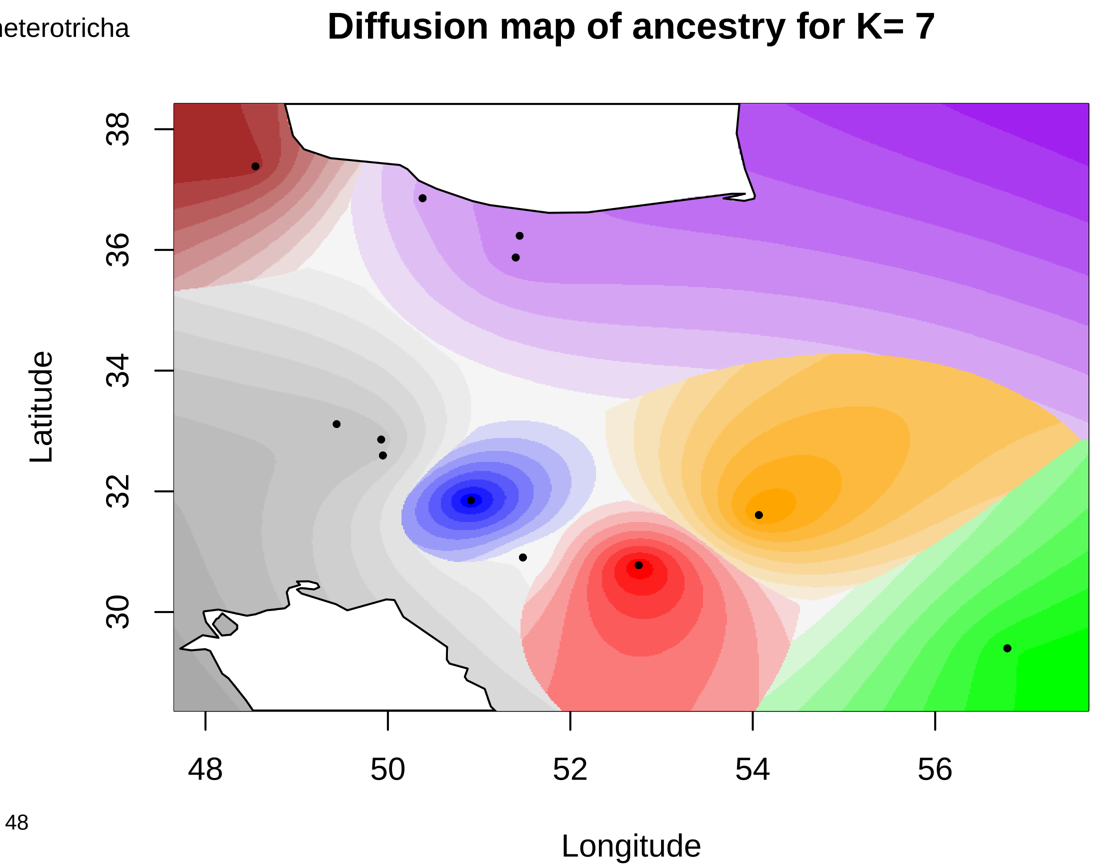

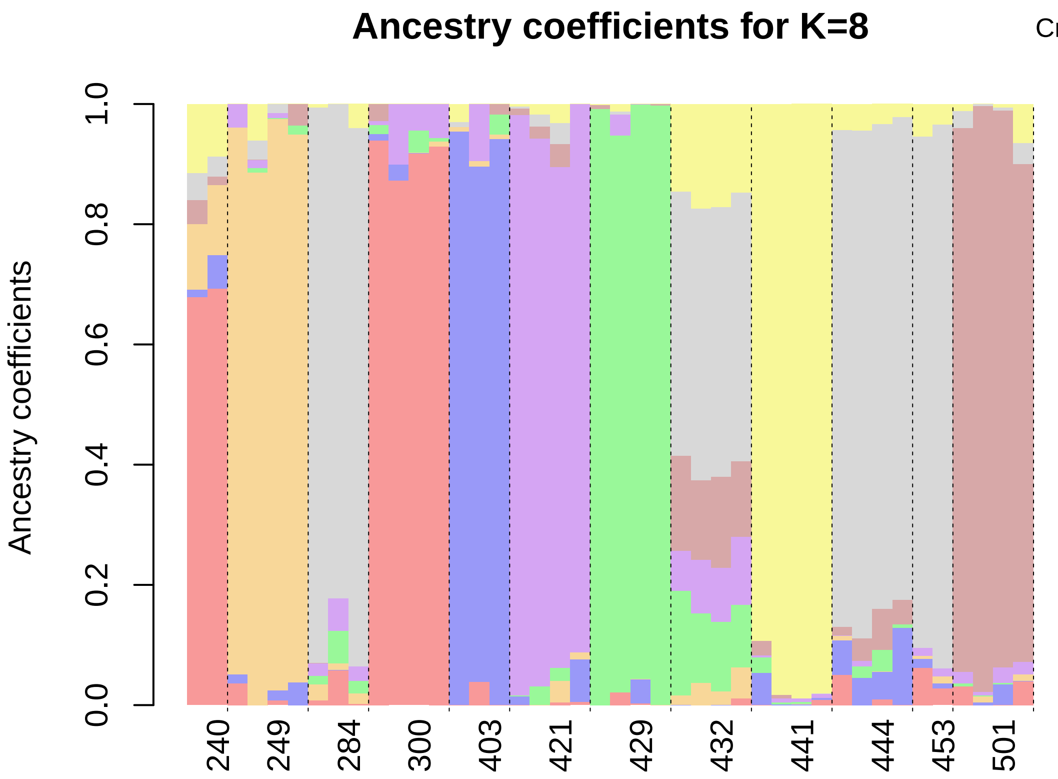

Crepis heterotricha

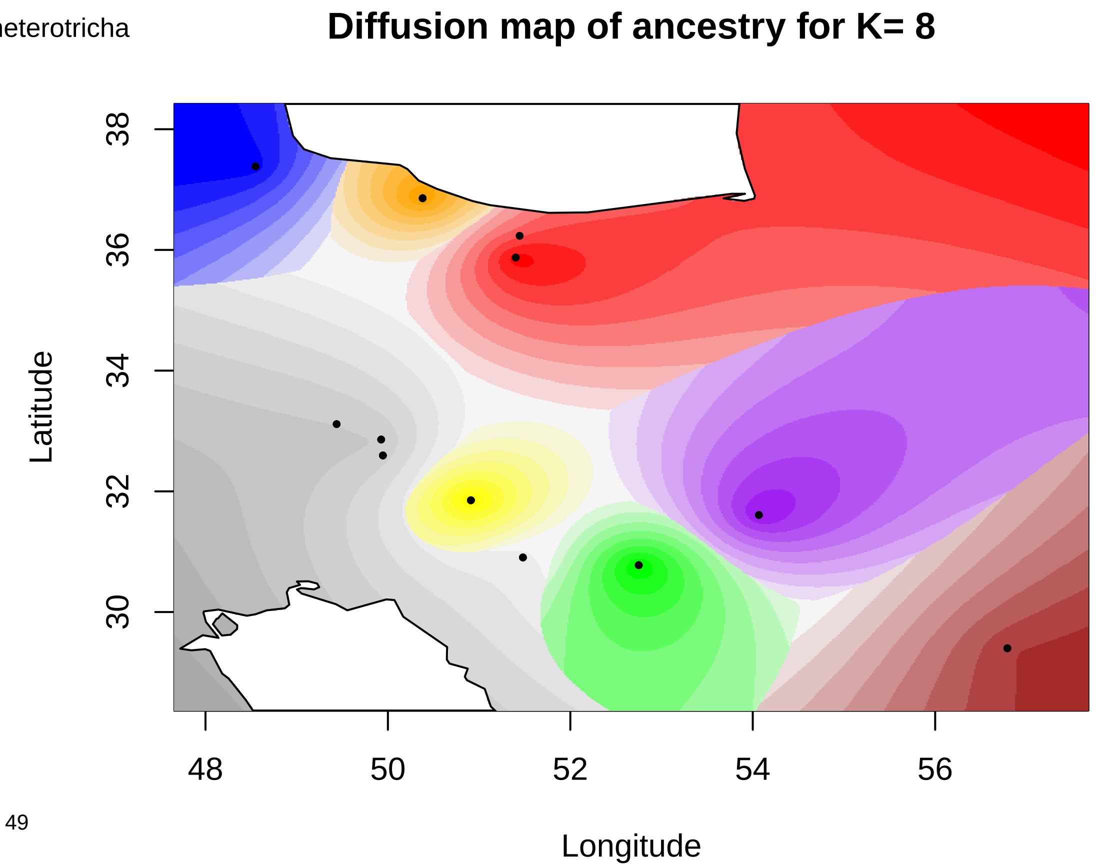

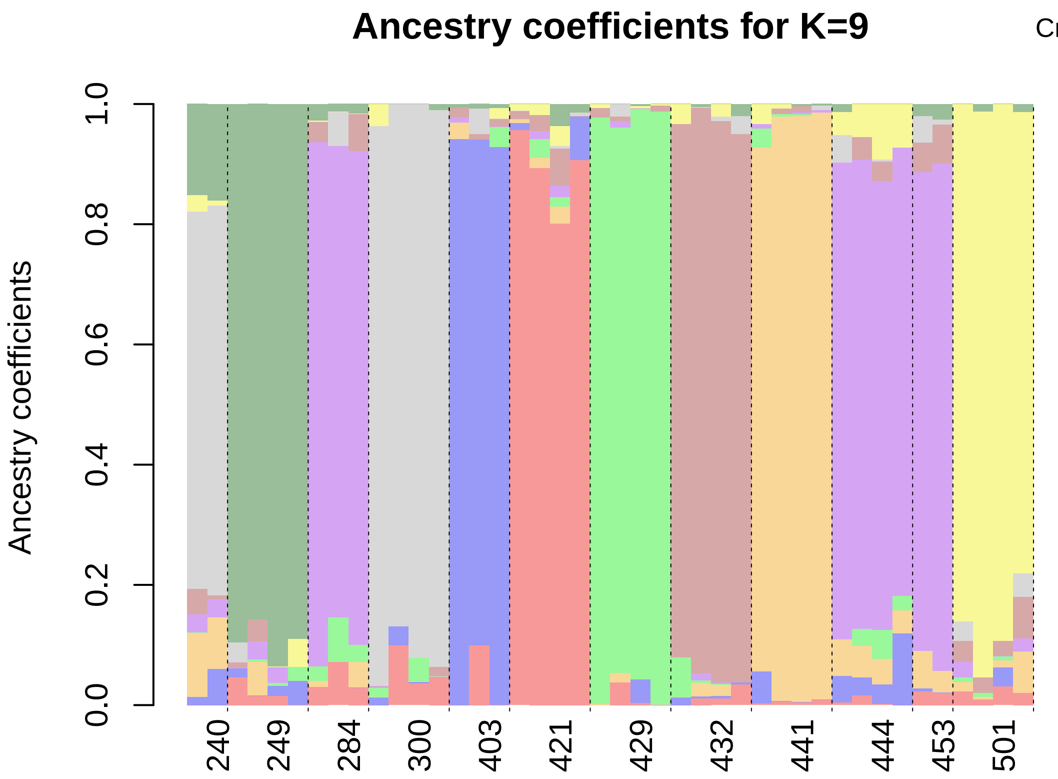

Crepis heterotricha

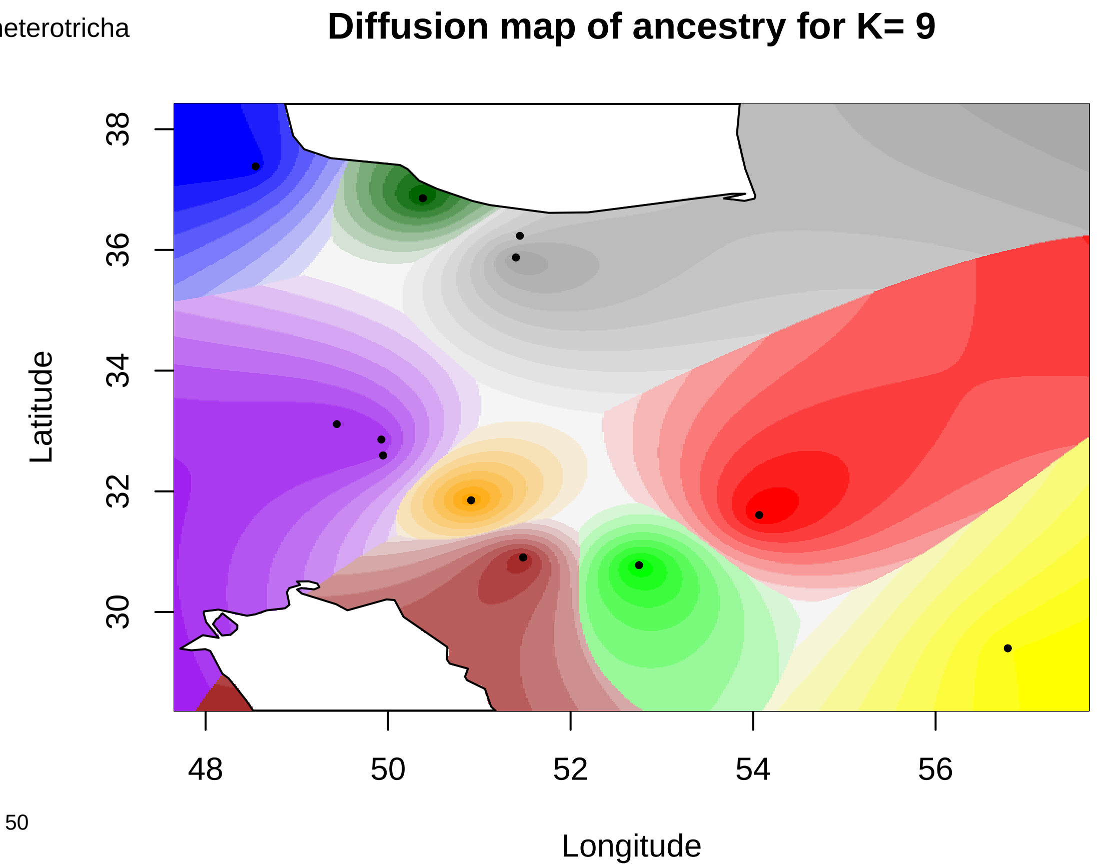

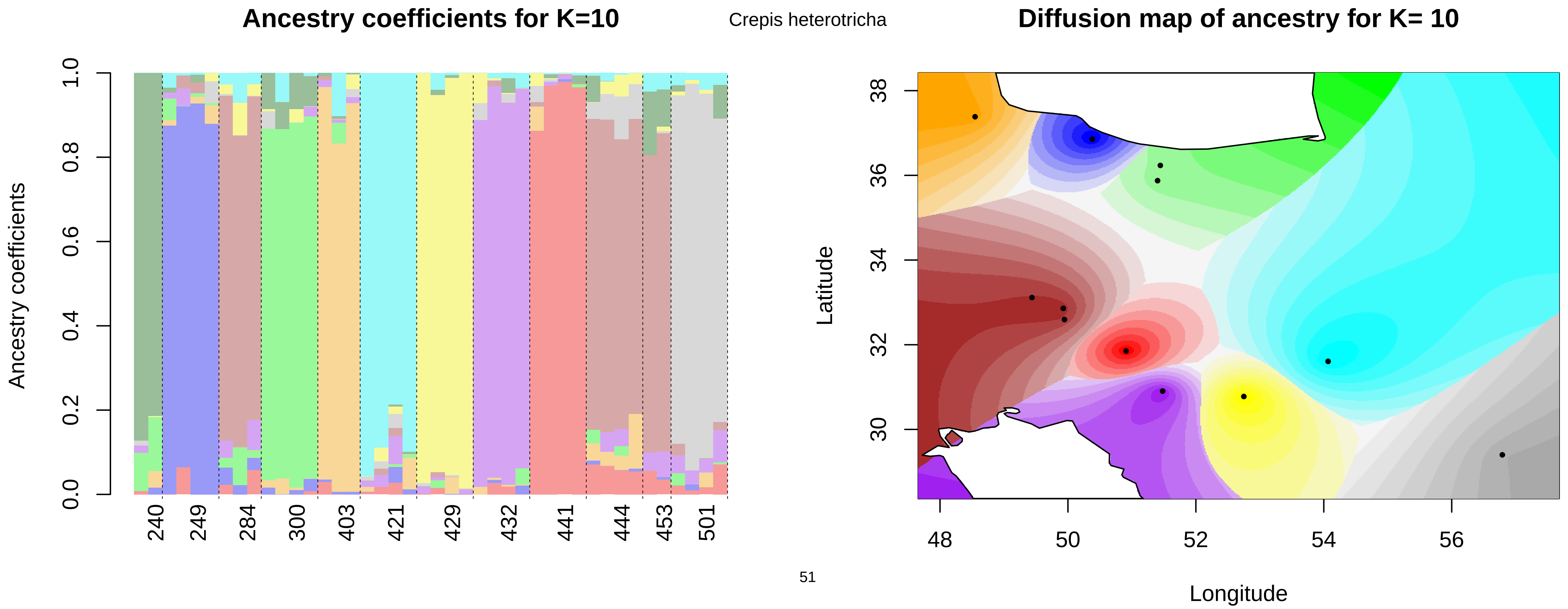
Ancestry coefficients for K=10
Crepis heterotricha
Diffusion map of ancestry for K= 10
Ancestry coefficients
0.0
0.2
0.4
0.6
0.8
1.0
240
249
284
300
403
421
429
432
441
444
453
501
Latitude
30
32
34
36
38
Longitude
48
50
52
54
56

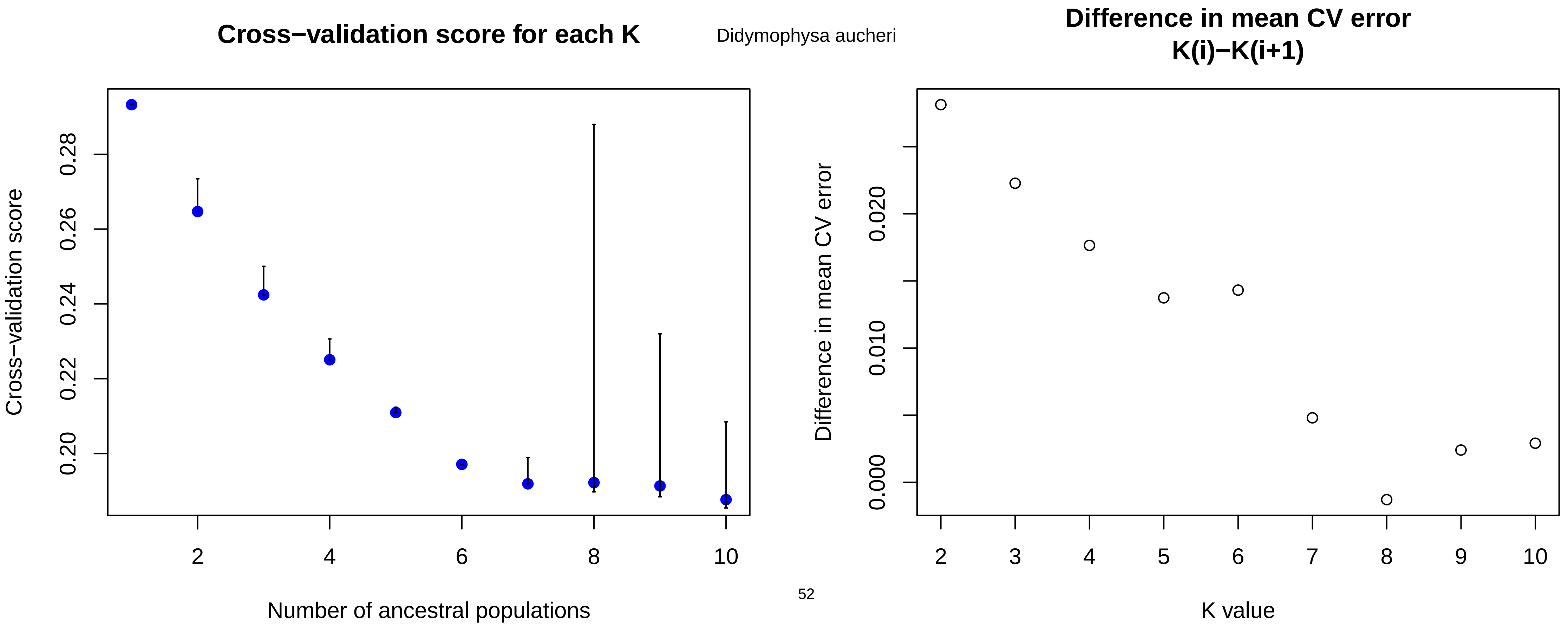
Cross−validation score for each K
Didymophysa aucheri
Difference in mean CV error
K(i)−K(i+1)
Cross−validation score
0.20
0.22
0.24
0.26
0.28
2
4
6
8
10
Number of ancestral populations
Difference in mean CV error
0.000
0.010
0.020
2
3
4
5
6
7
8
9
10
K value

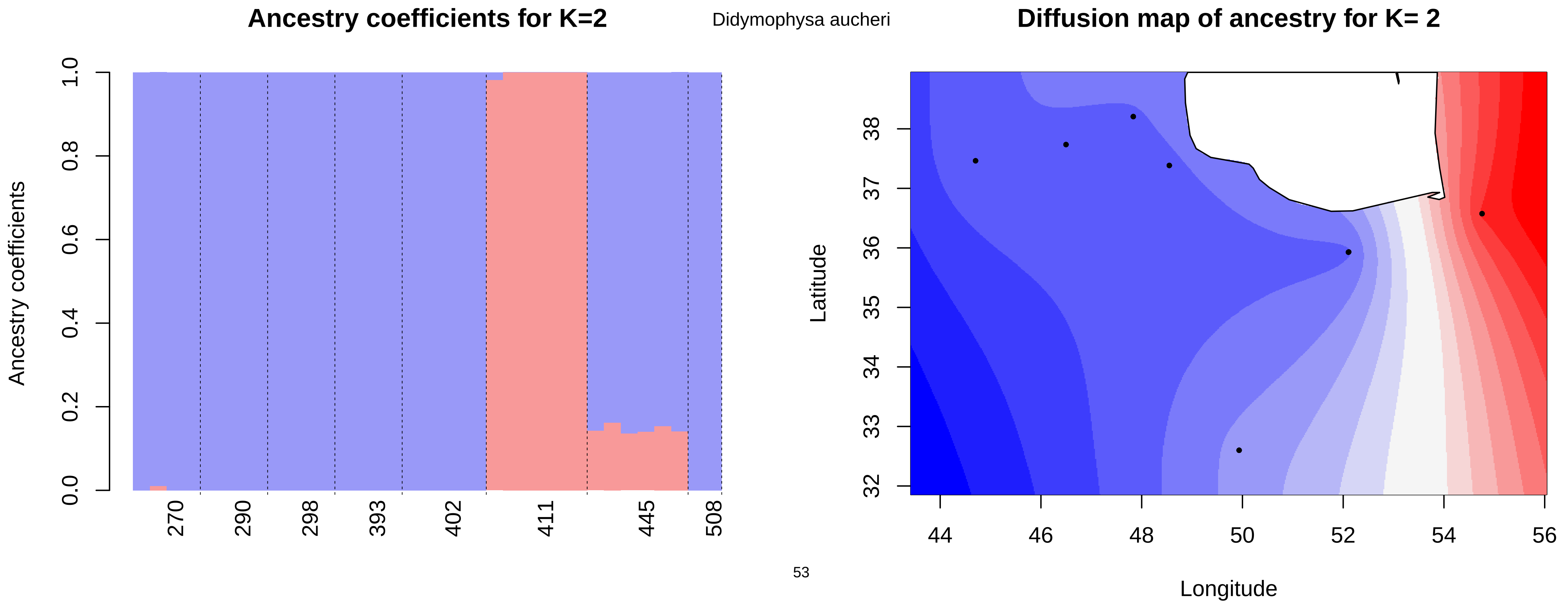

Ancestry coefficients for K=2
Didymophysa aucheri
Diffusion map of ancestry for K= 2
Ancestry coefficients
0.0
0.2
0.4
0.6
0.8
1.0
270
290
298
393
402
411
445
508
Latitude
32
33
34
35
36
37
38
Longitude
44
46
48
50
52
54
56

Didymophysa aucheri

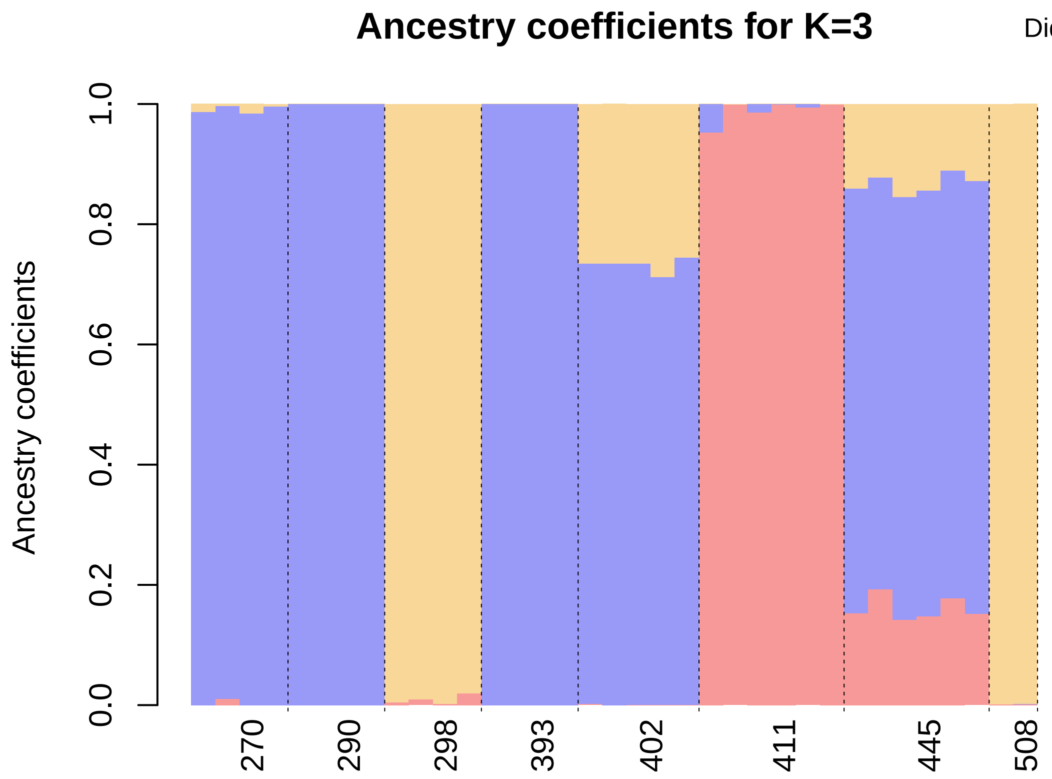

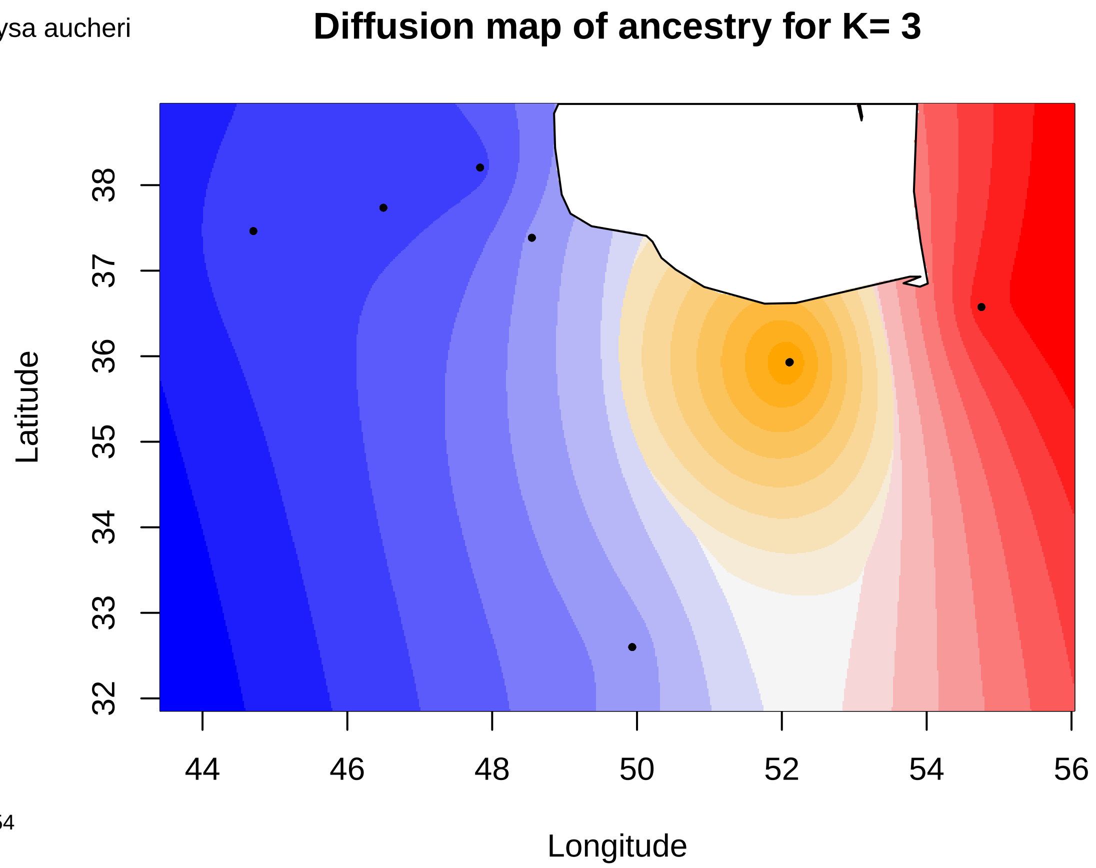

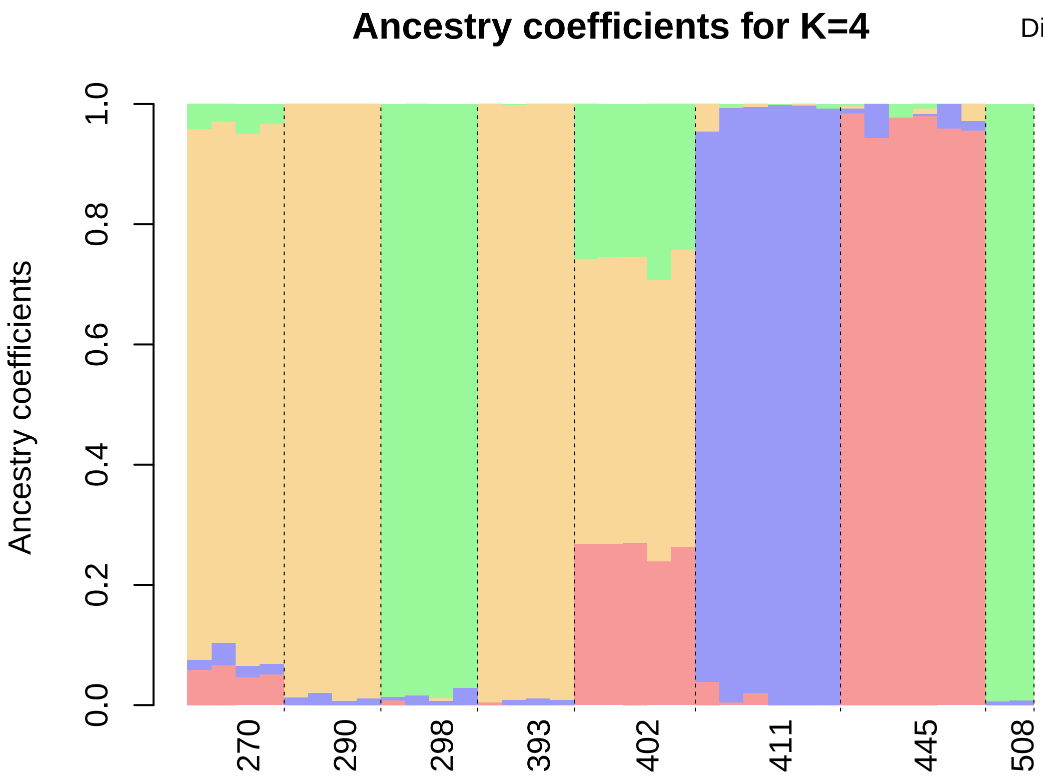

Didymophysa aucheri

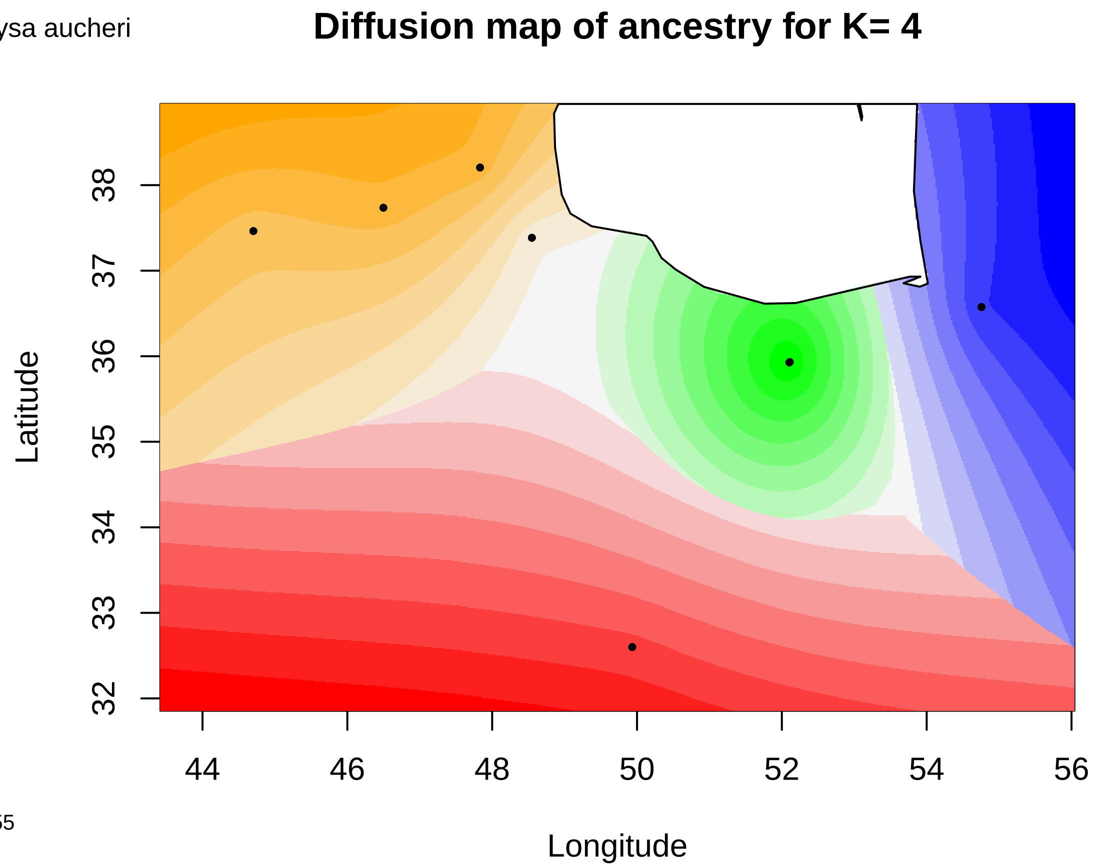

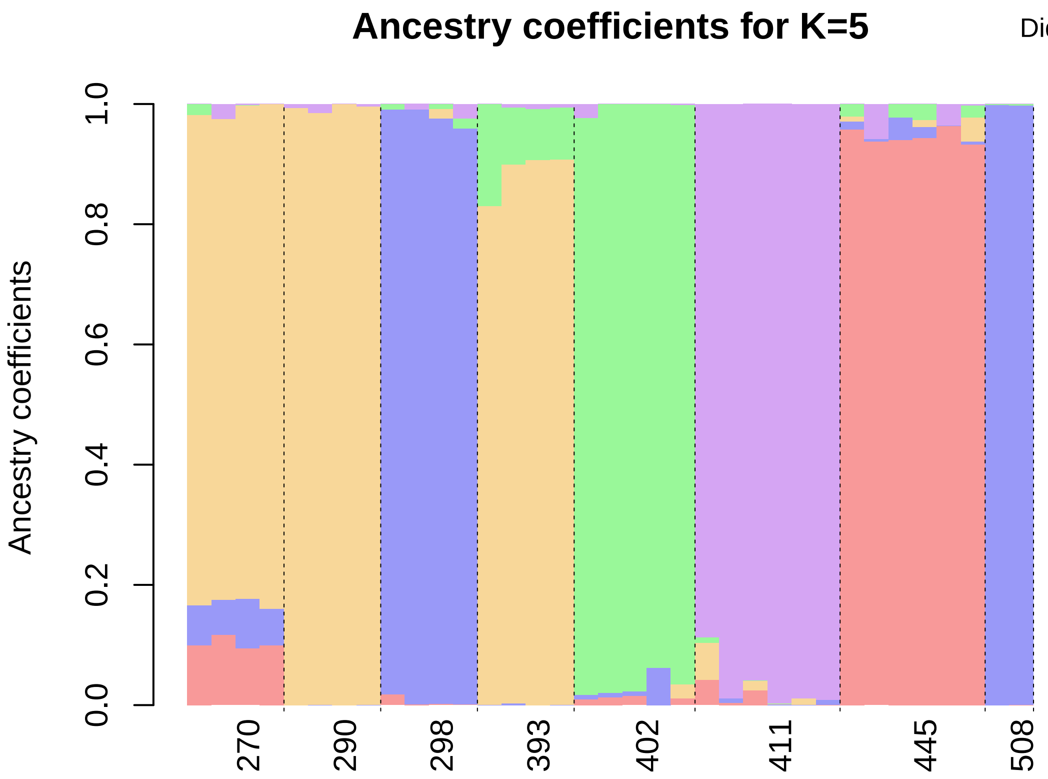

Didymophysa aucheri

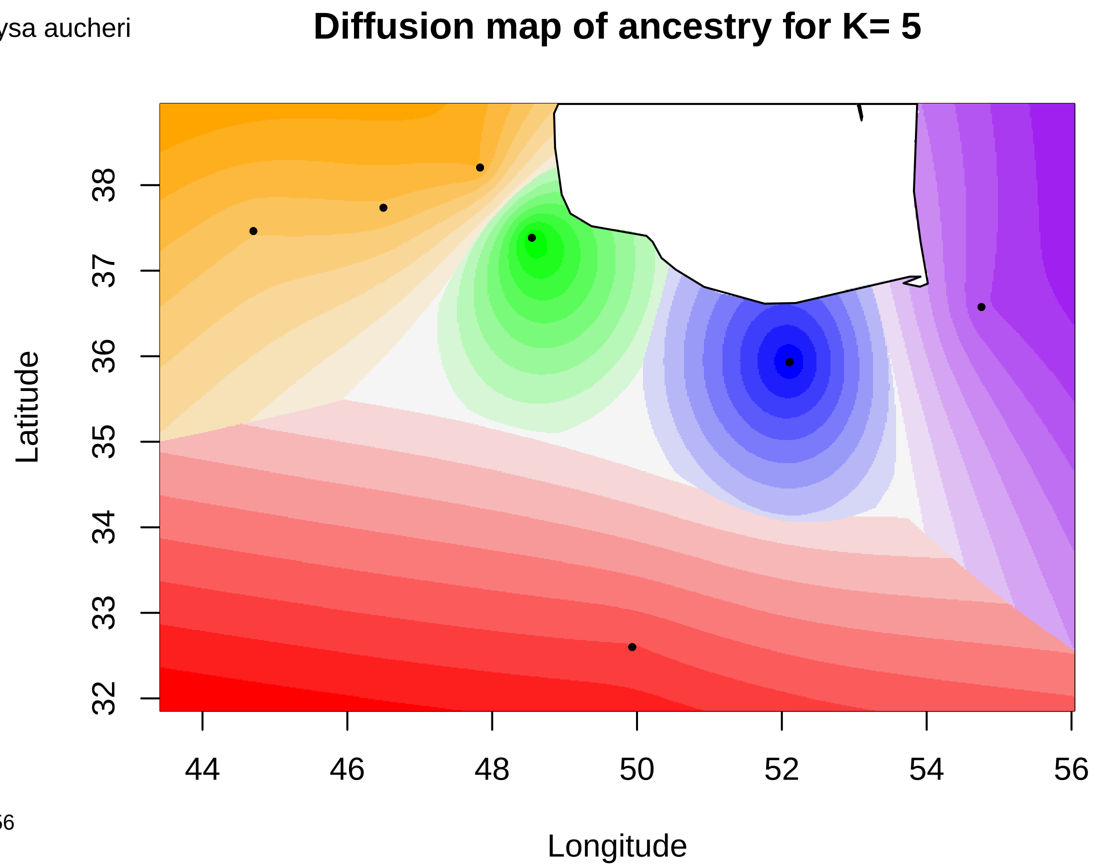

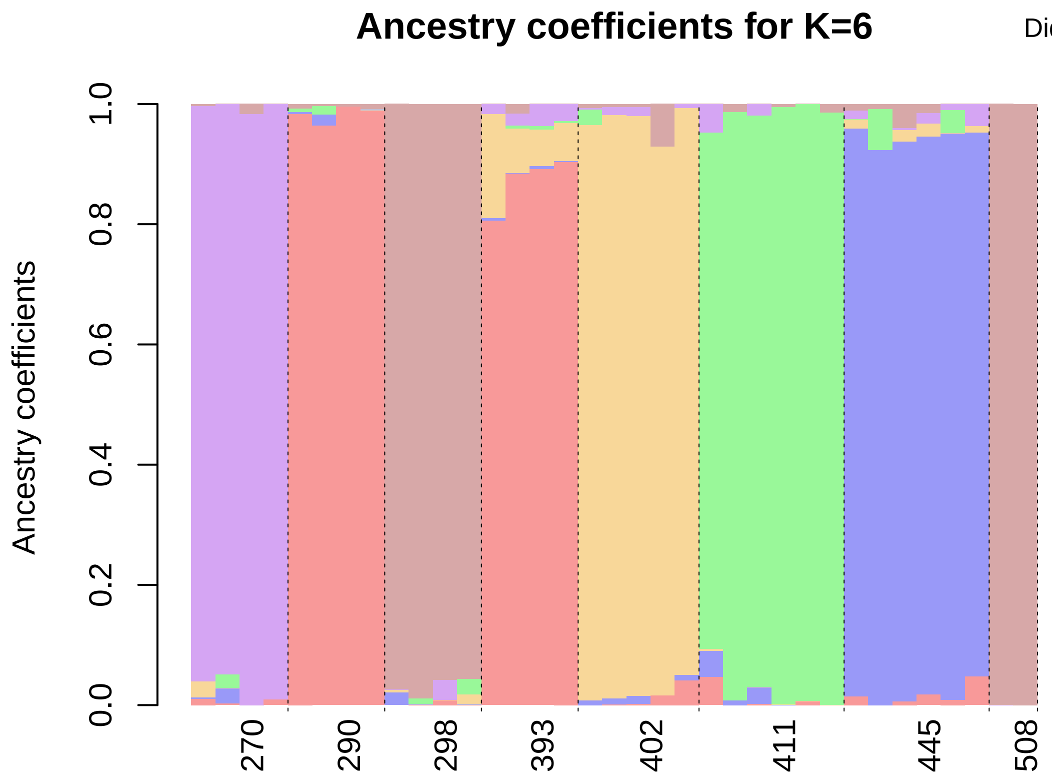

Didymophysa aucheri

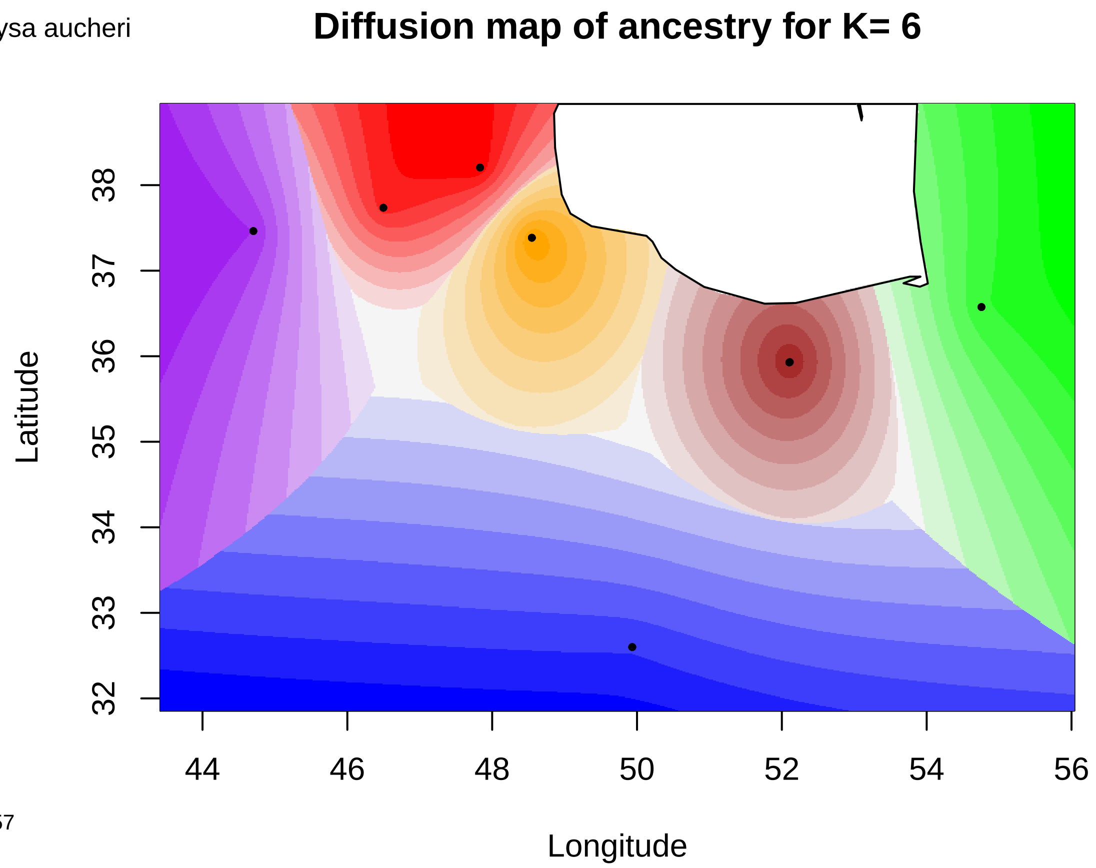

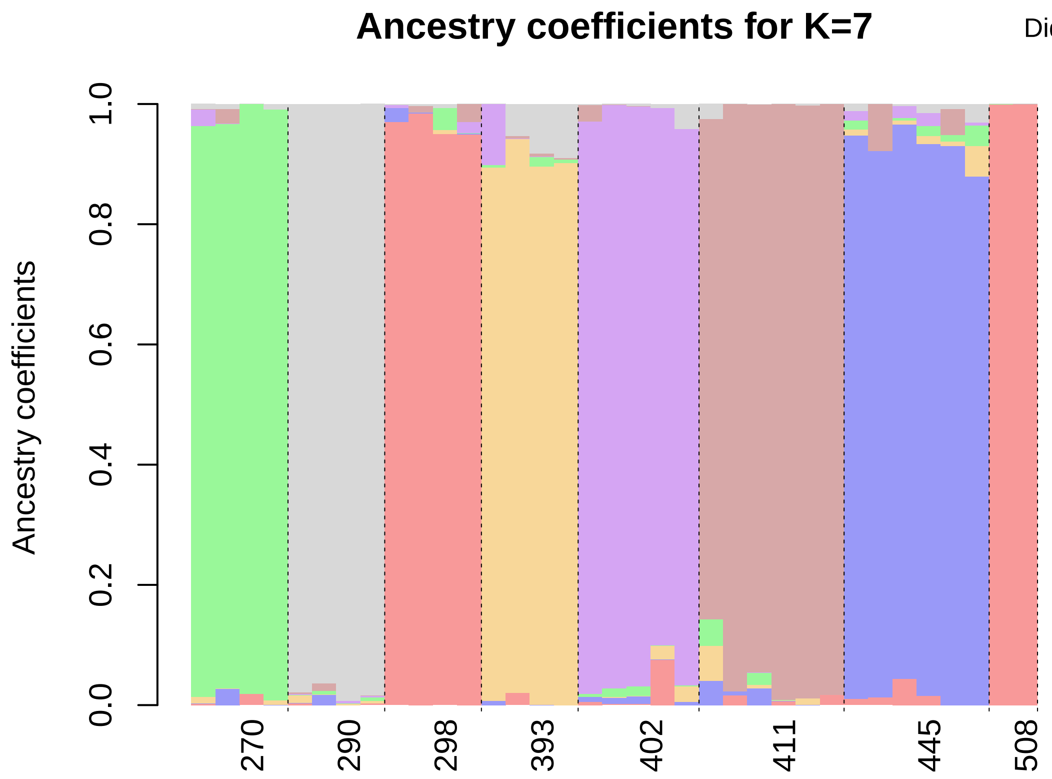

Didymophysa aucheri

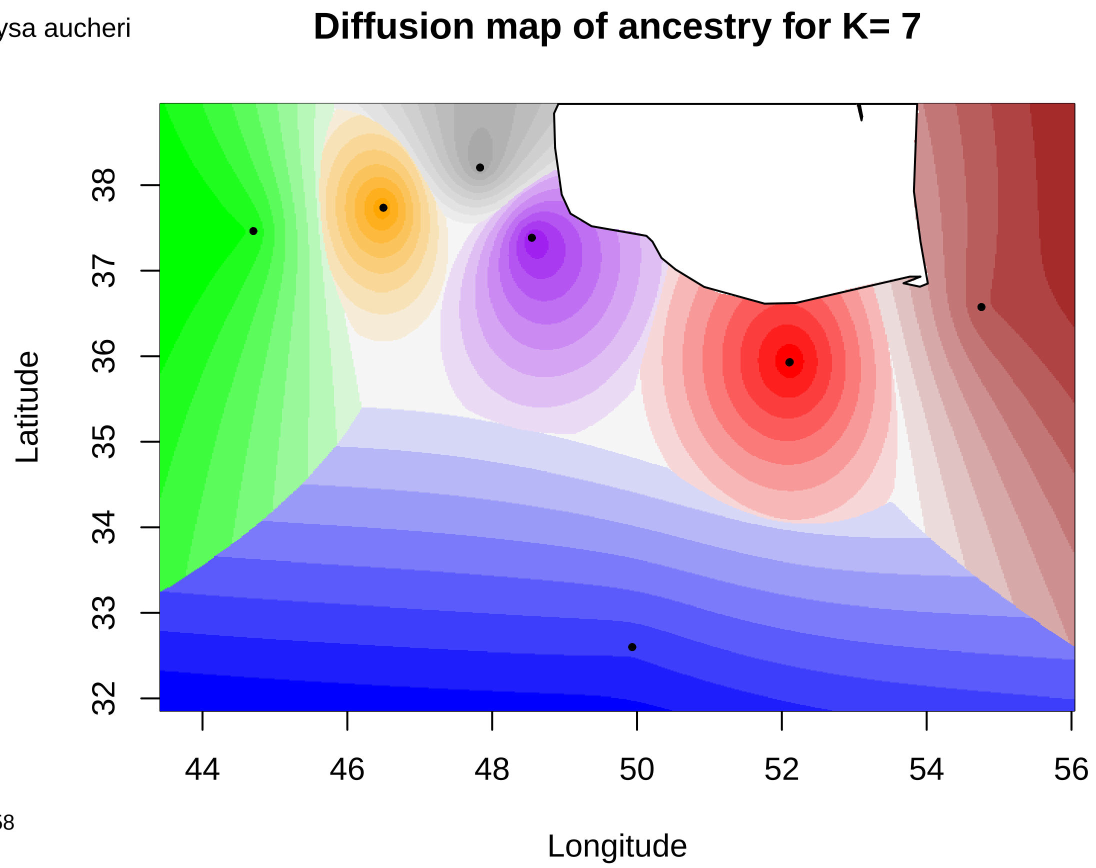

Didymophysa aucheri

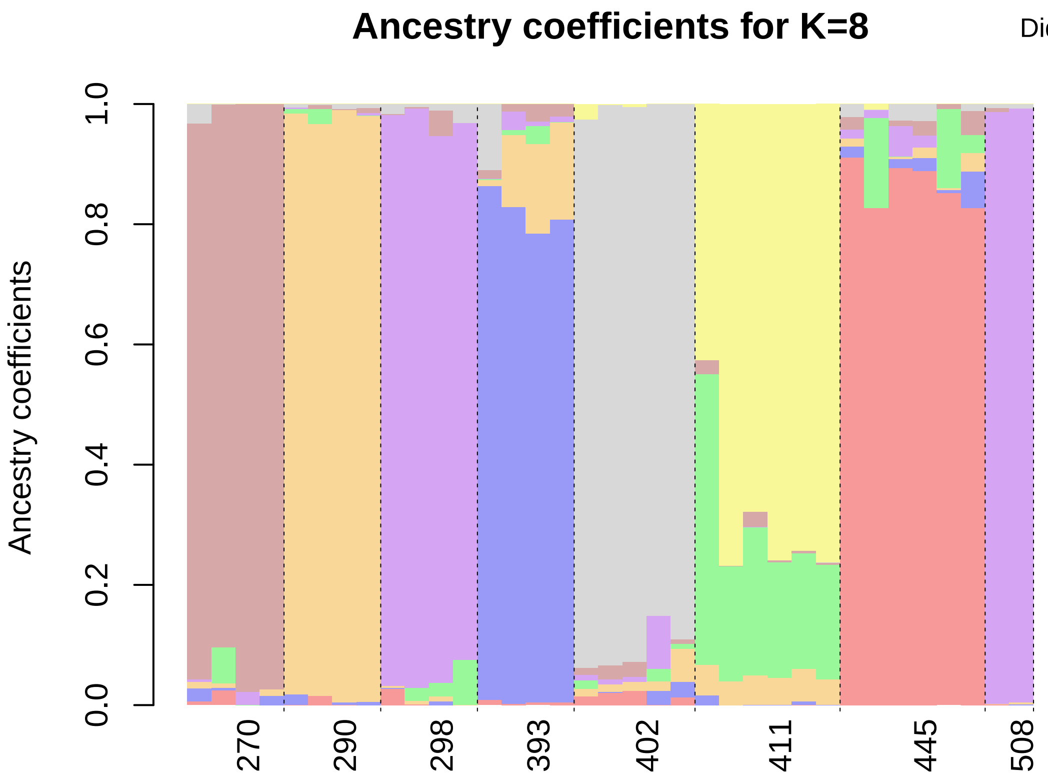

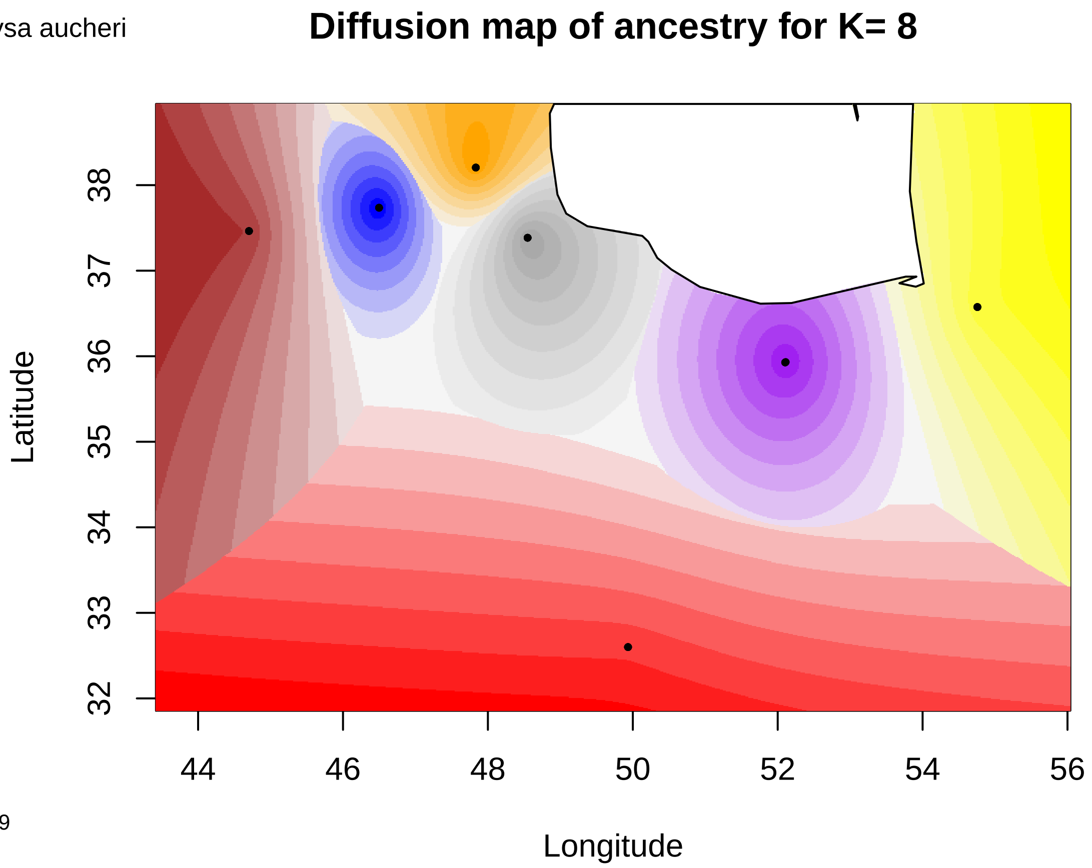

Didymophysa aucheri

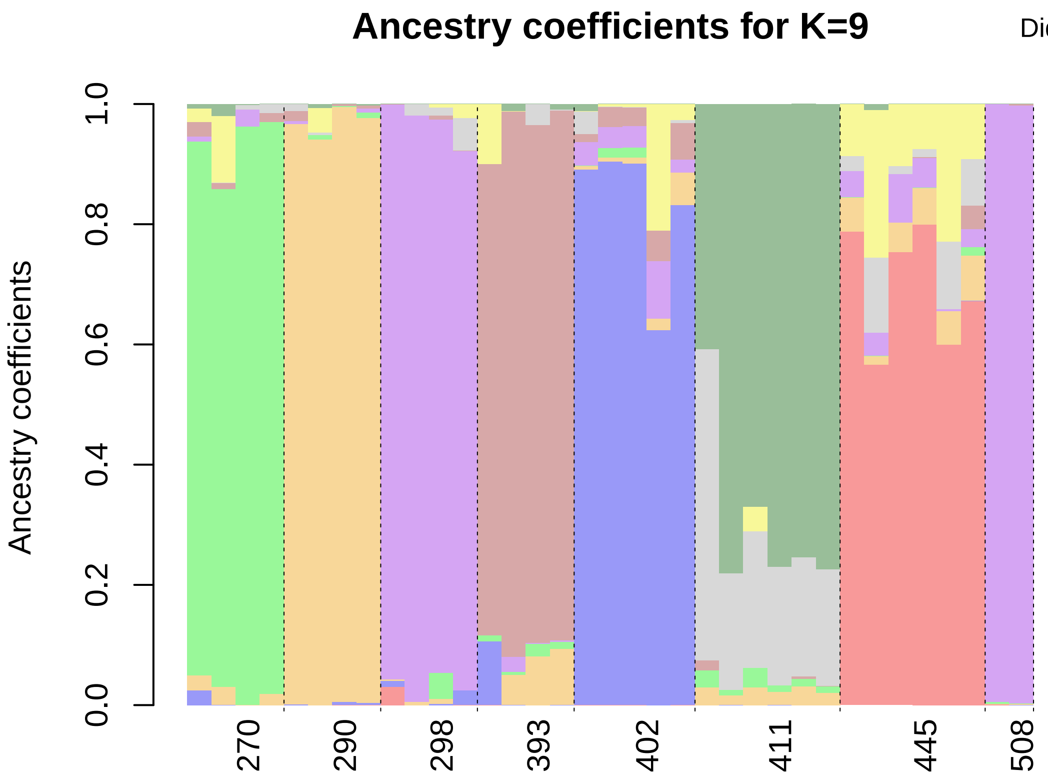

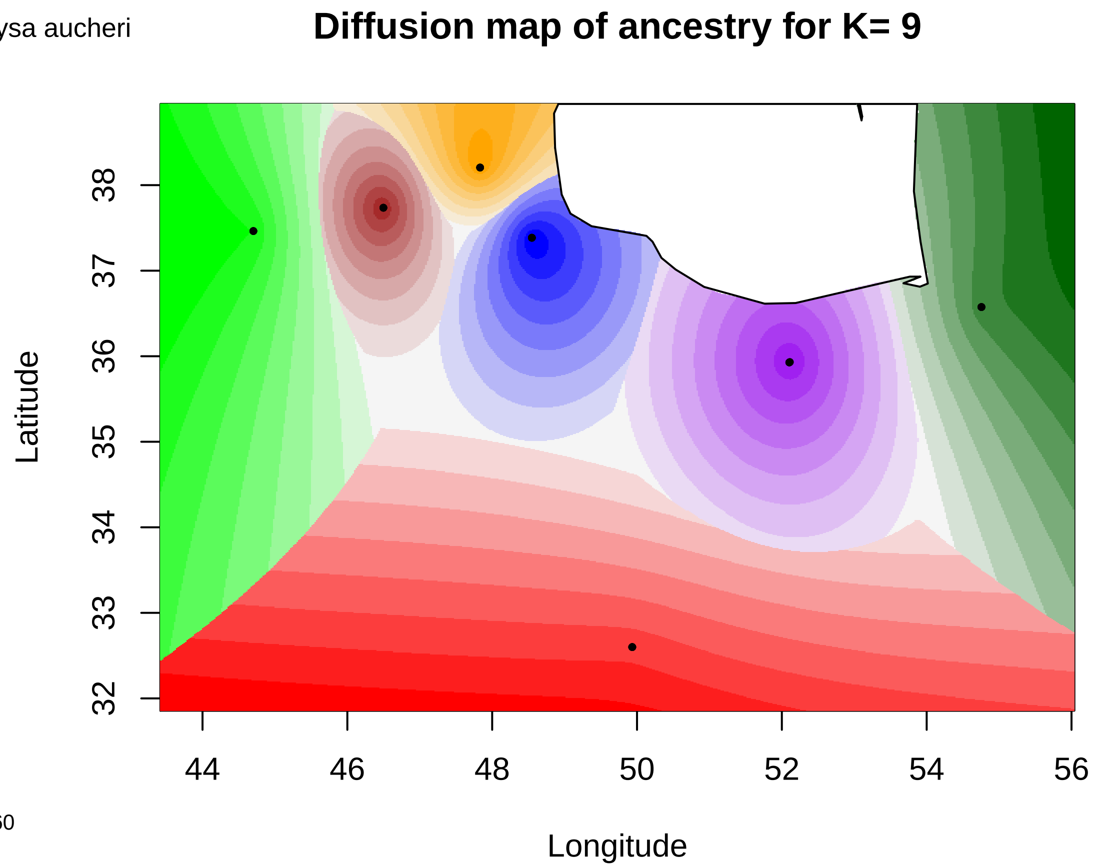

Didymophysa aucheri

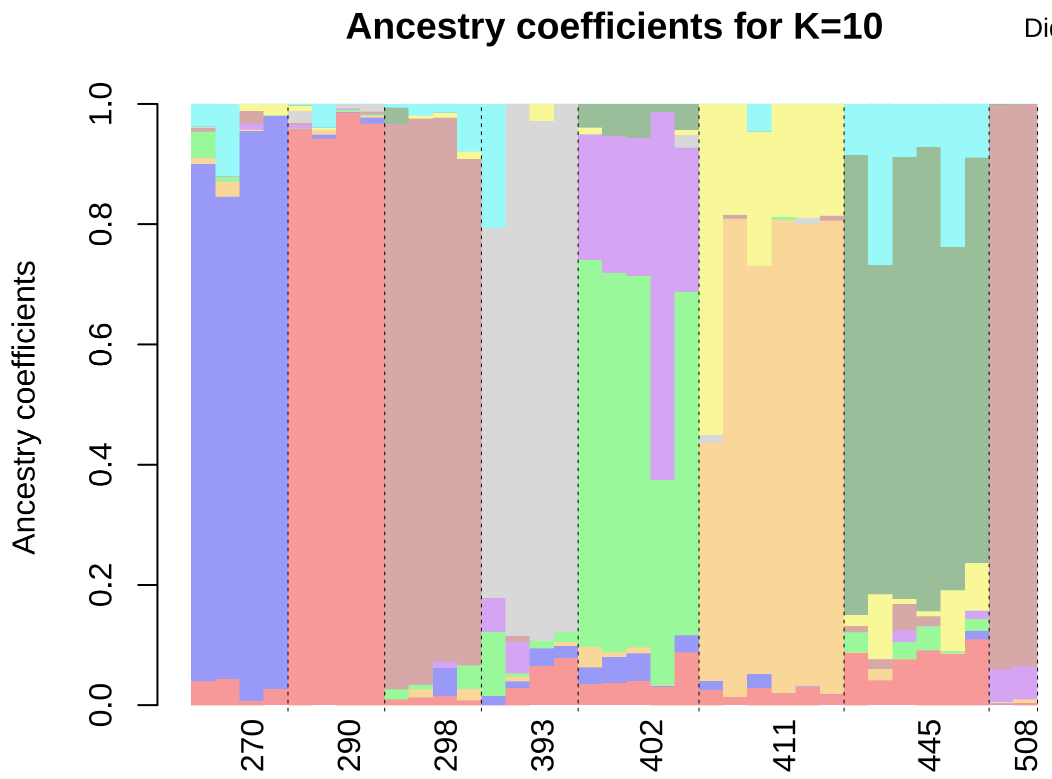

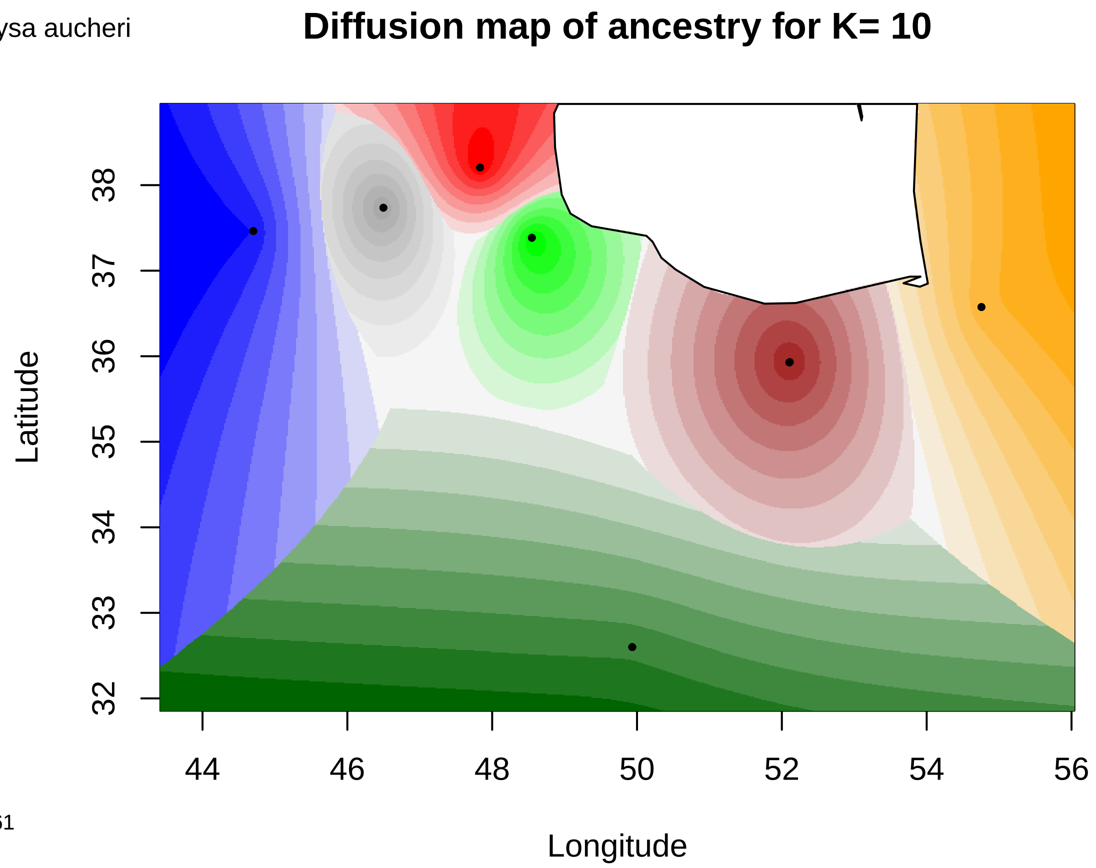

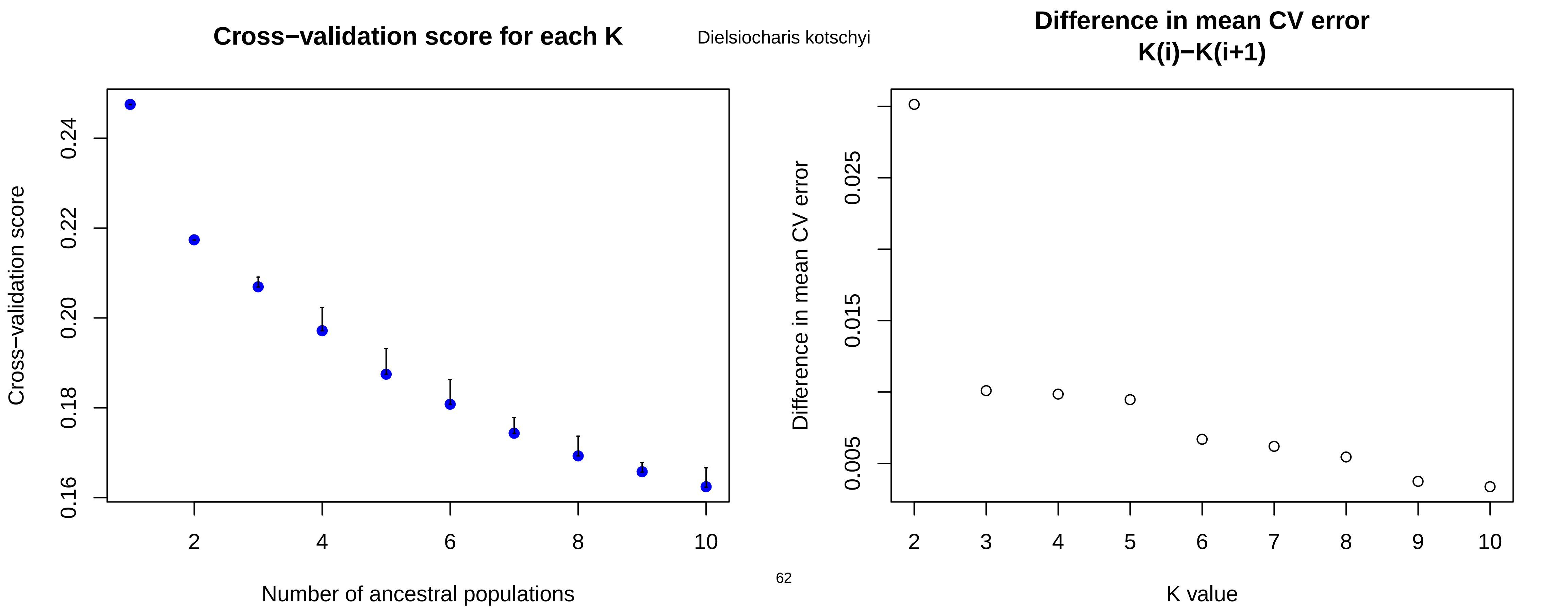

Cross−validation score for each K
Dielsiocharis kotschyi
Difference in mean CV error
K(i)−K(i+1)
Cross−validation score
0.16
0.18
0.20
0.22
0.24
2
4
6
8
10
Number of ancestral populations
Difference in mean CV error
0.005
0.015
0.025
2
3
4
5
6
7
8
9
10
K value

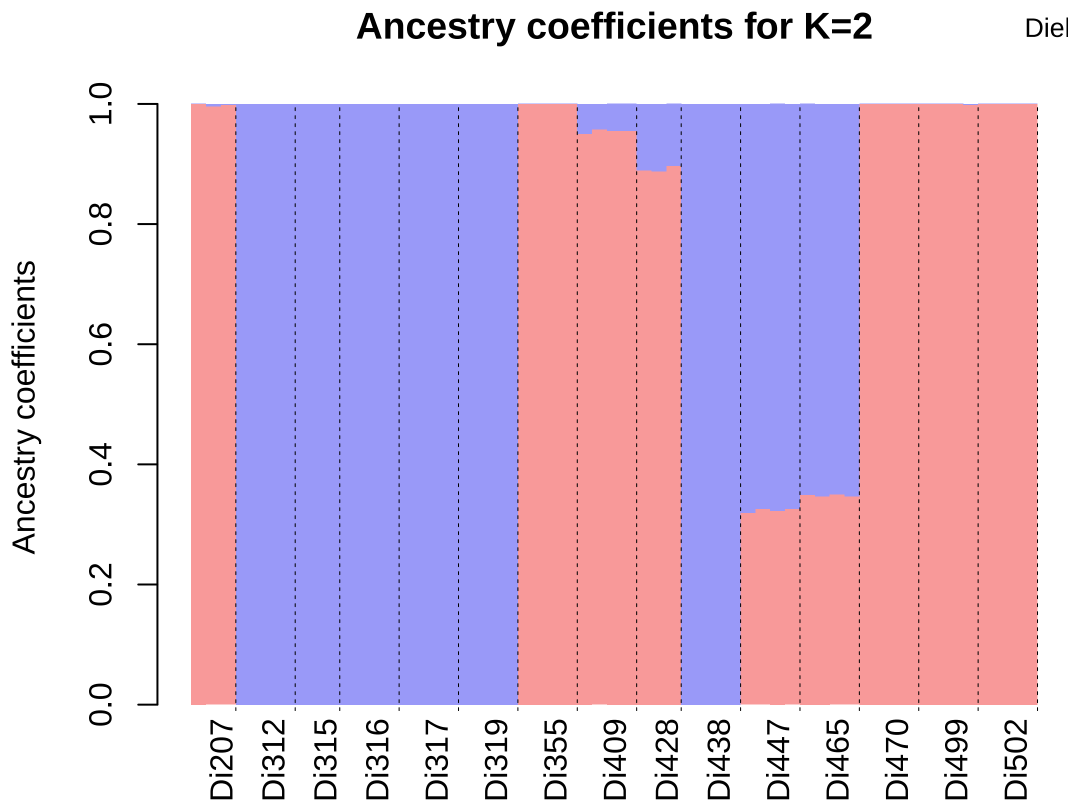

Dielsiocharis kotschyi

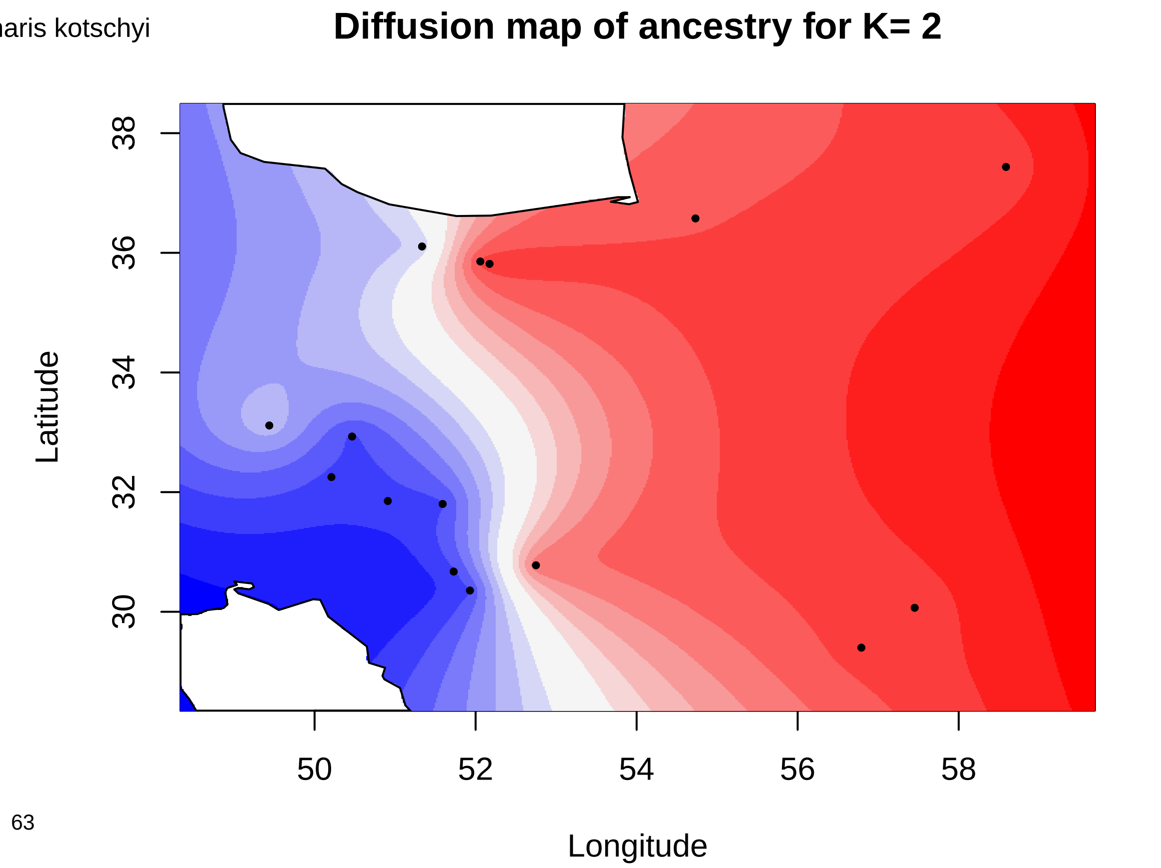

Dielsiocharis kotschyi

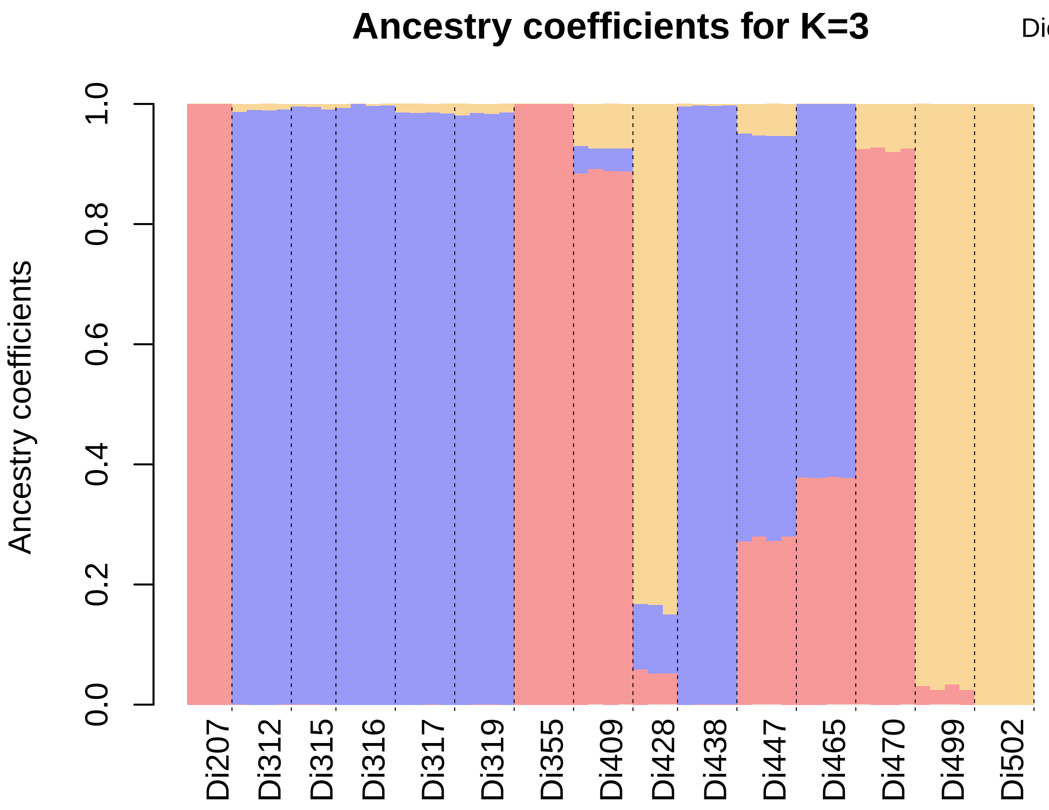

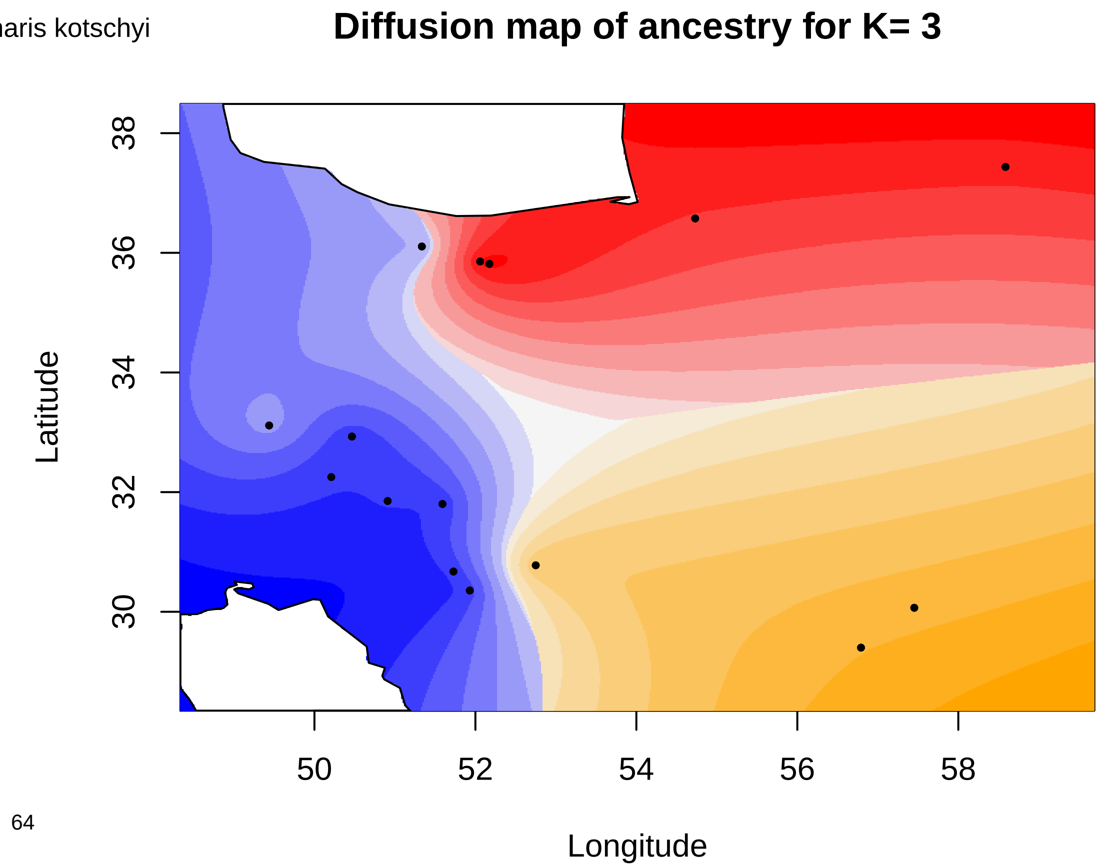

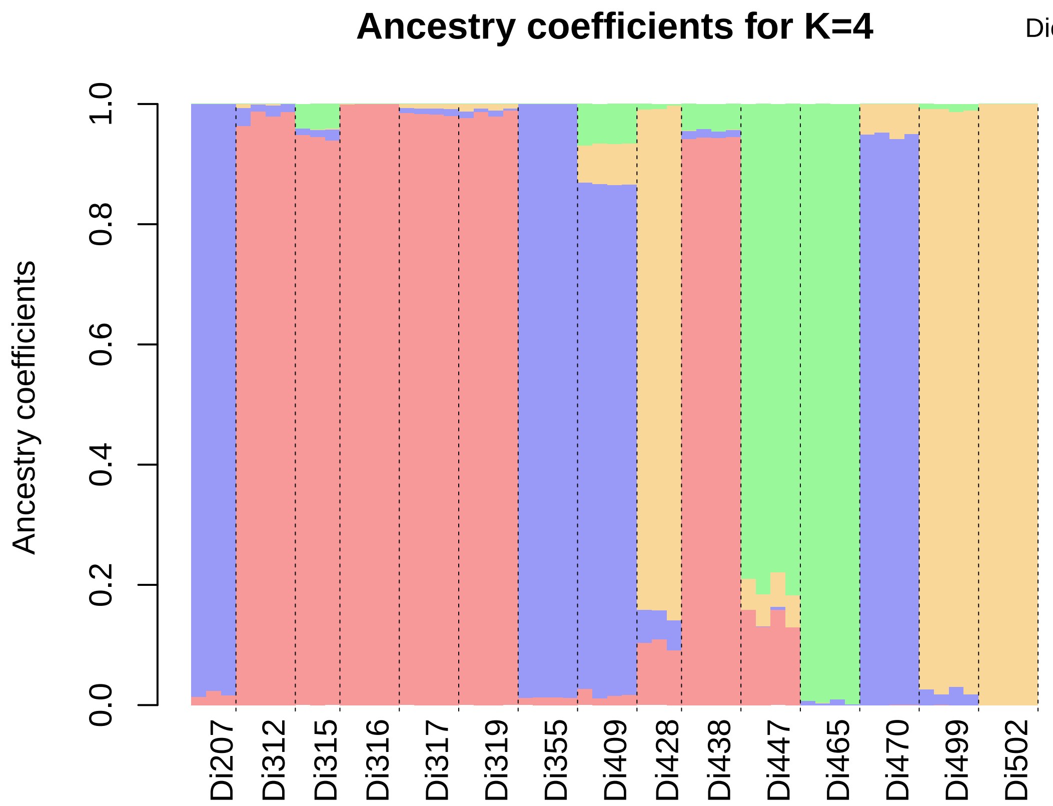

Dielsiocharis kotschyi

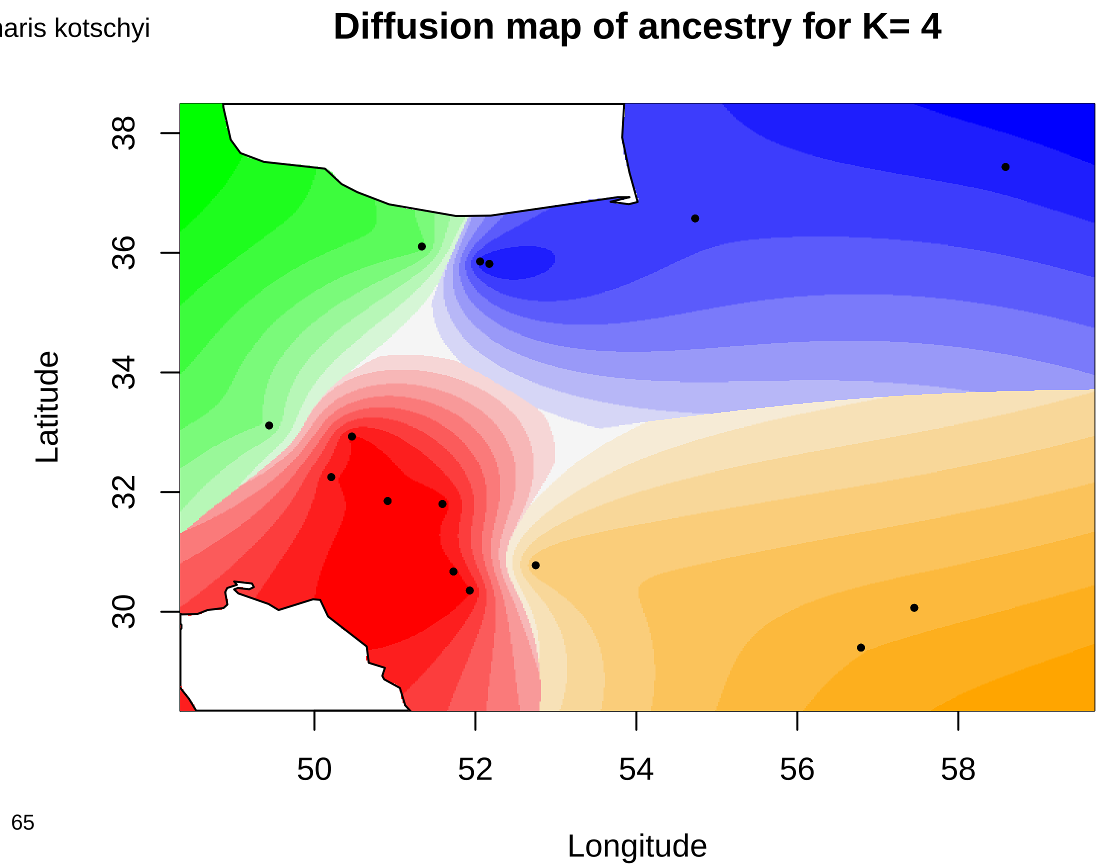

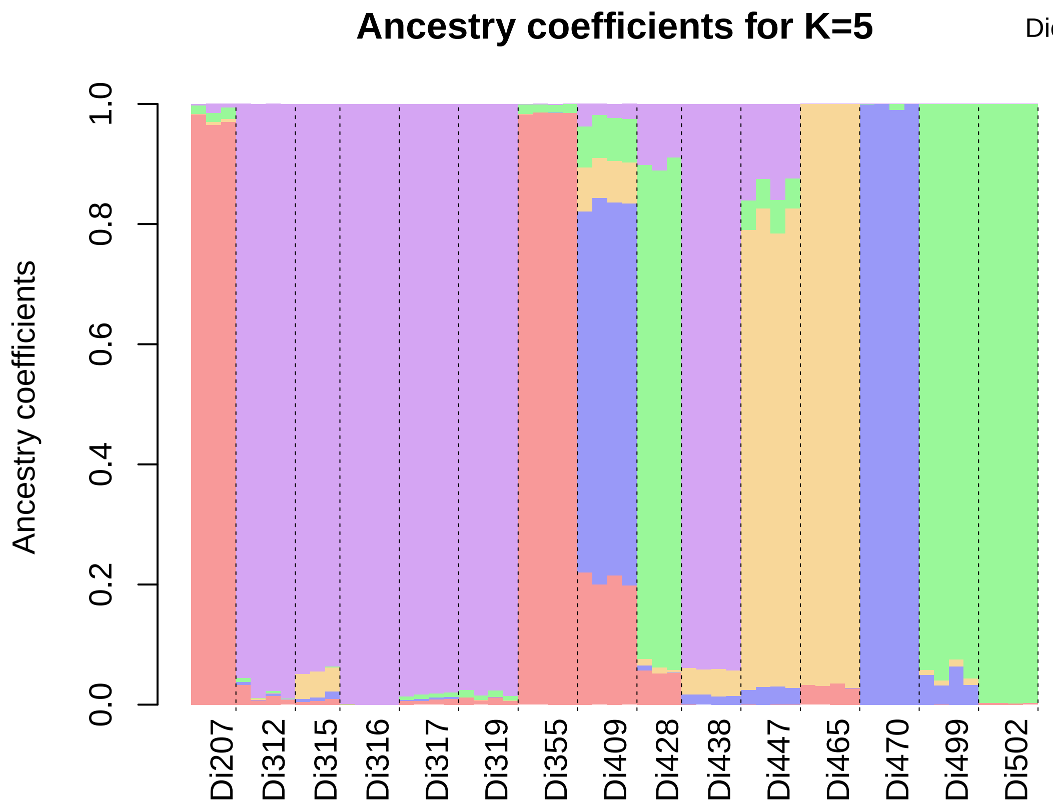

Dielsiocharis kotschyi

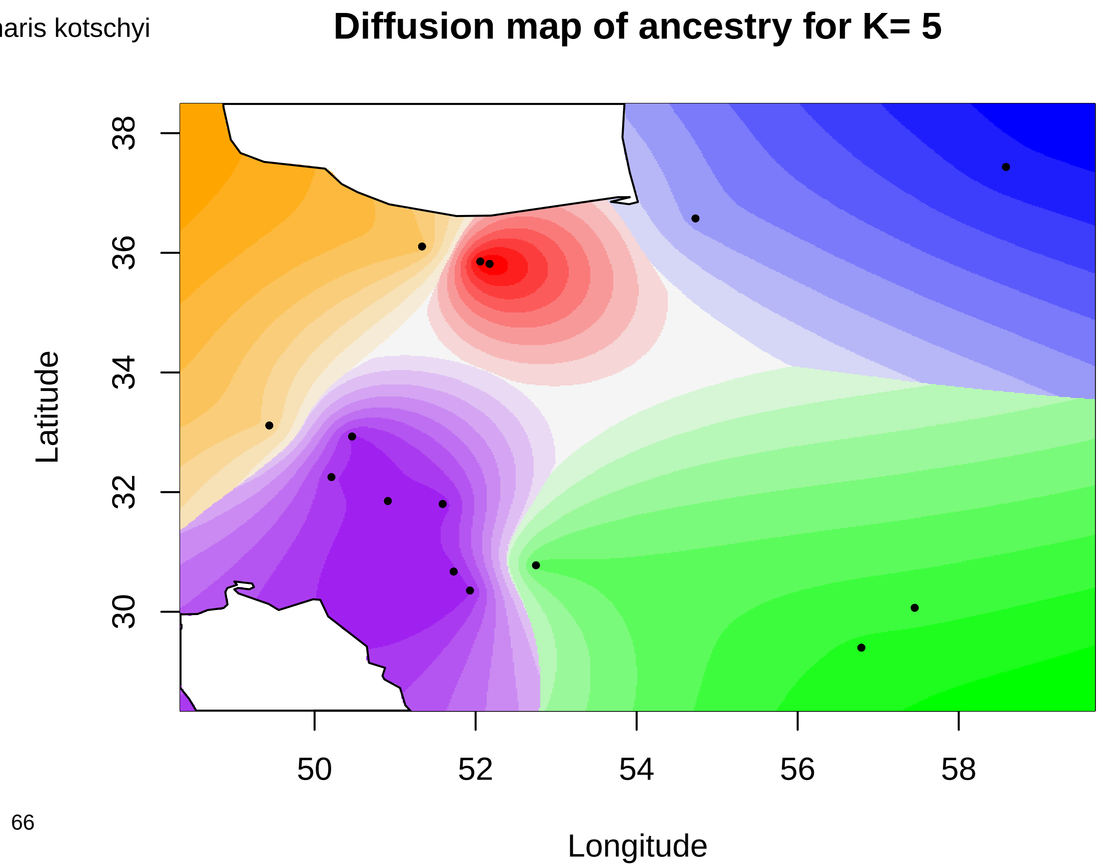

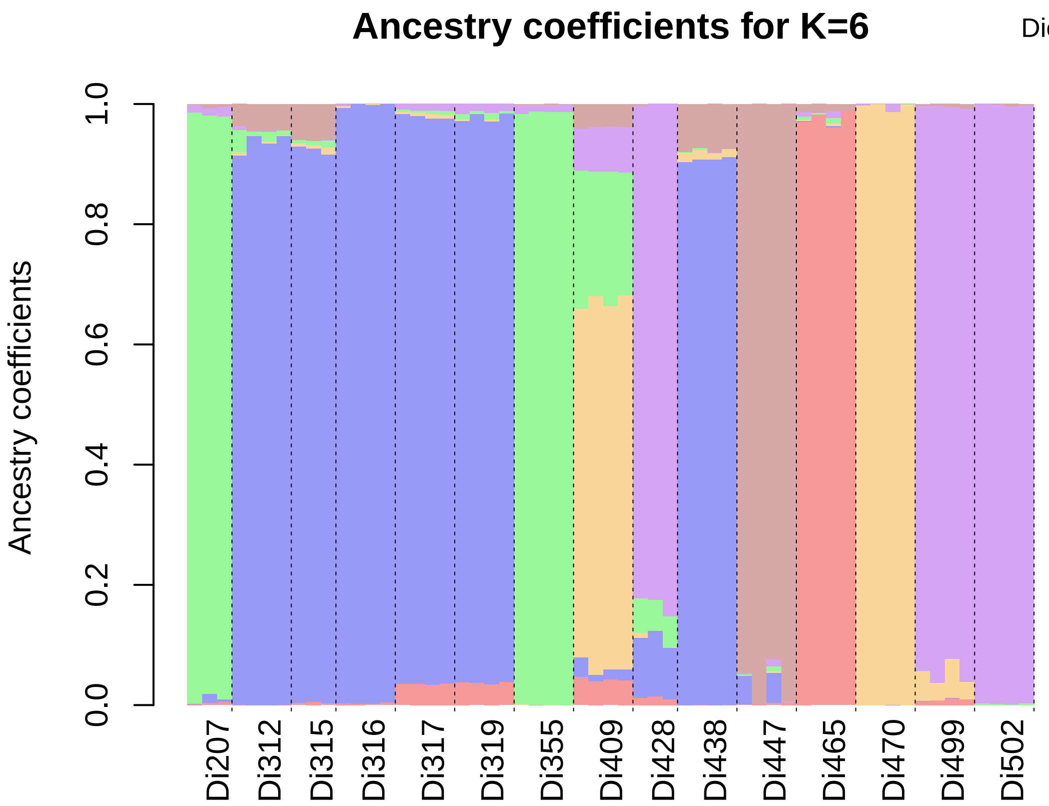

Dielsiocharis kotschyi

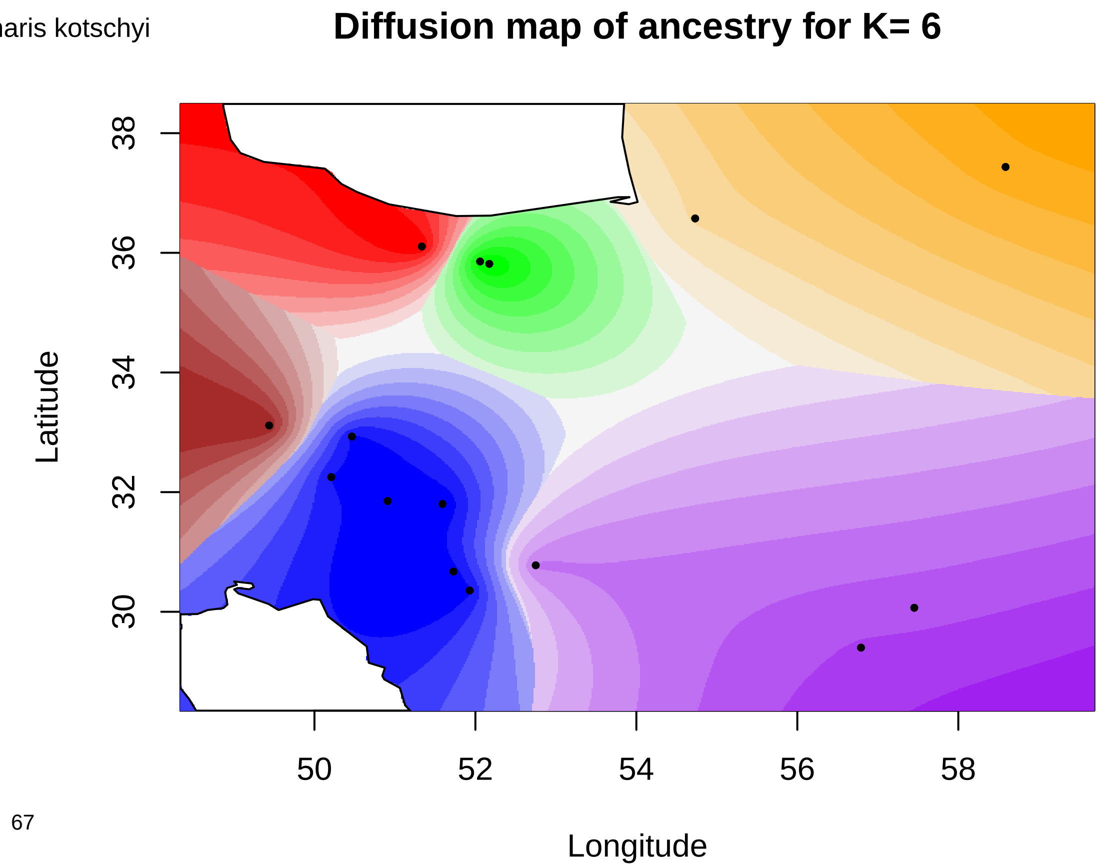

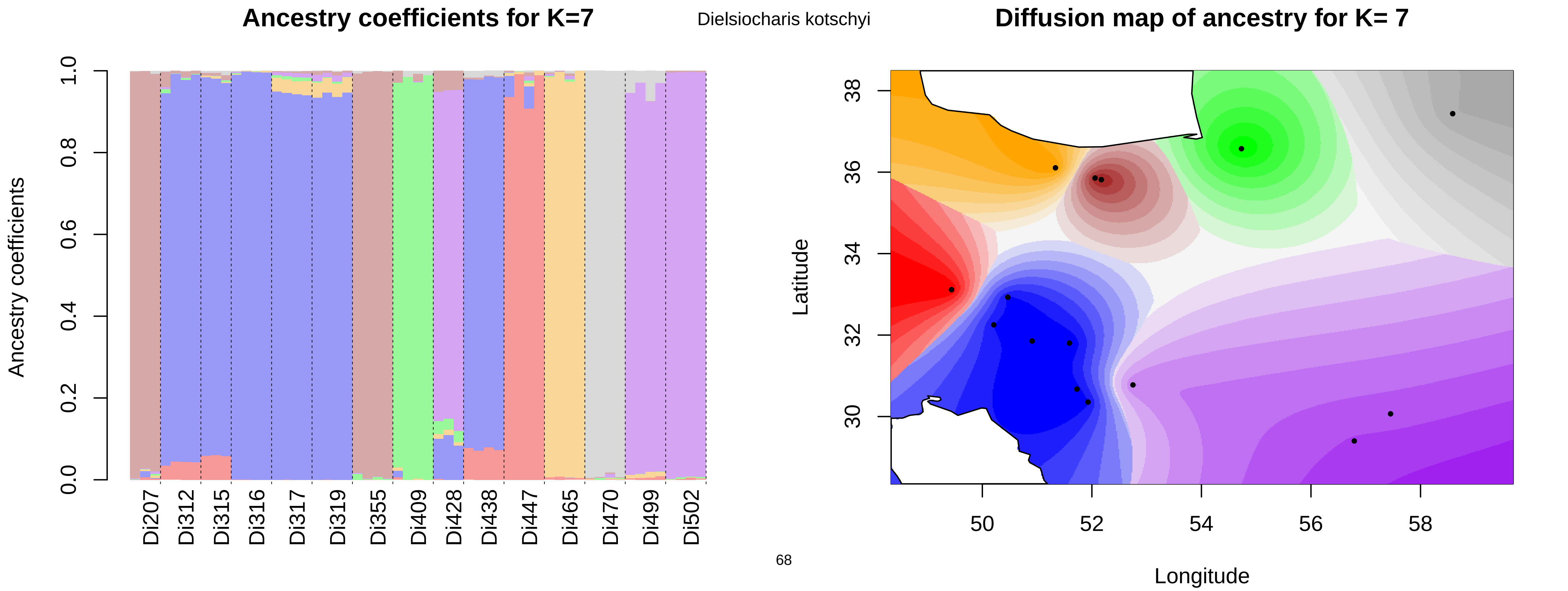

Ancestry coefficients for K=7
Dielsiocharis kotschyi
Diffusion map of ancestry for K= 7
Ancestry coefficients
0.0
0.2
0.4
0.6
0.8
1.0
Di207
Di312
Di315
Di316
Di317
Di319
Di355
Di409
Di428
Di438
Di447
Di465
Di470
Di499
Di502
Latitude
30
32
34
36
38
Longitude
50
52
54
56
58

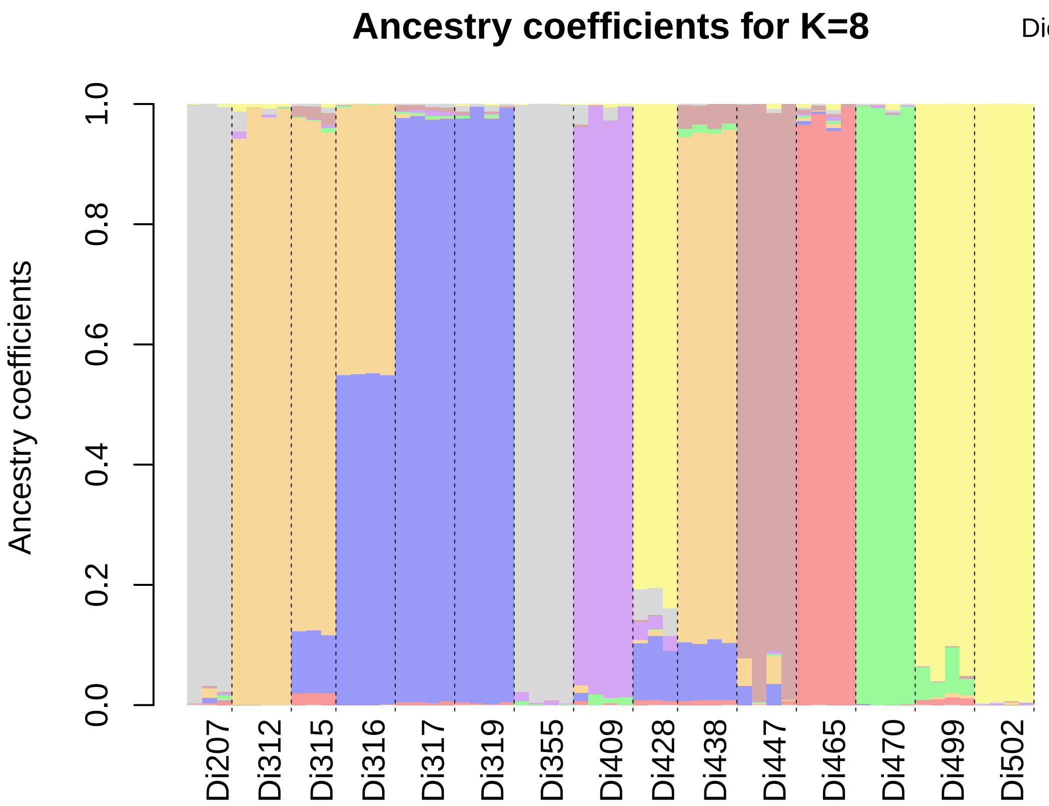

Dielsiocharis kotschyi

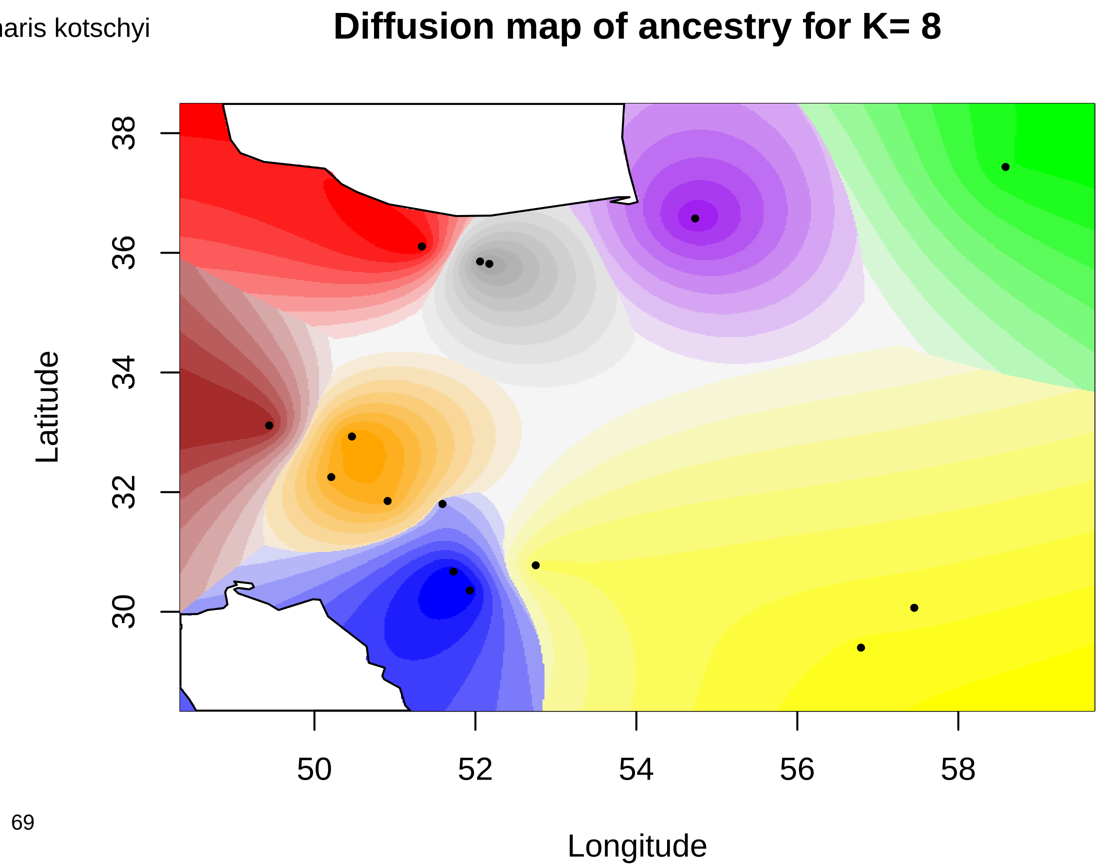

Dielsiocharis kotschyi

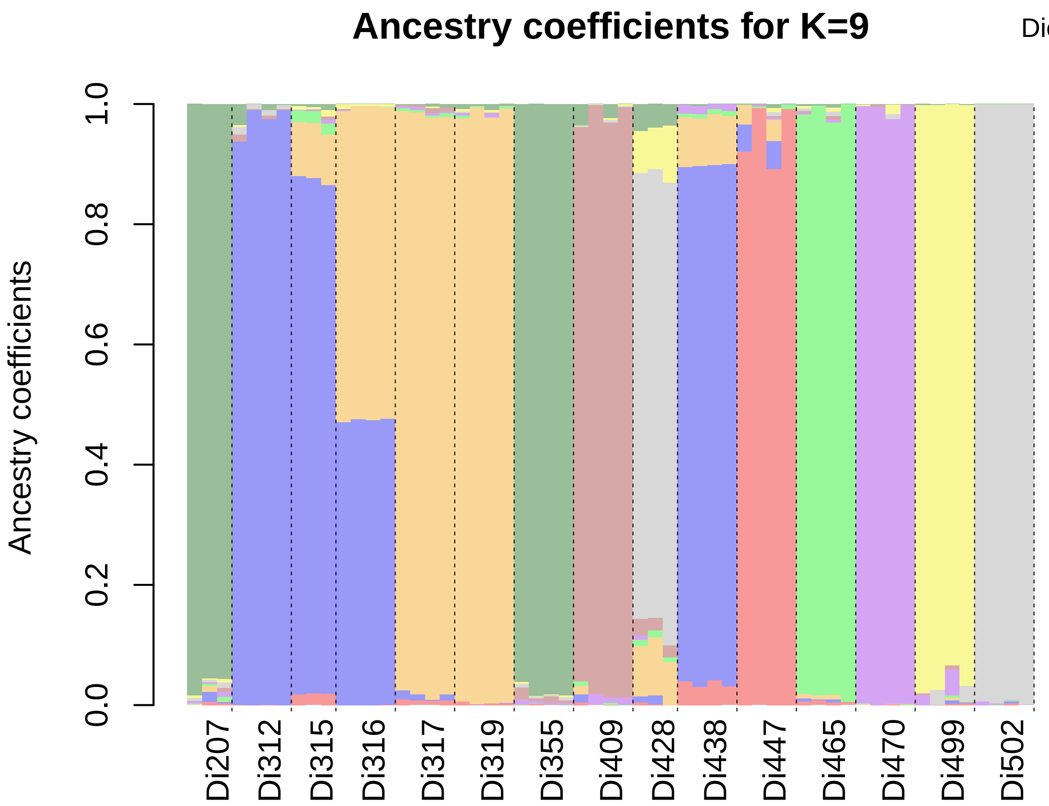

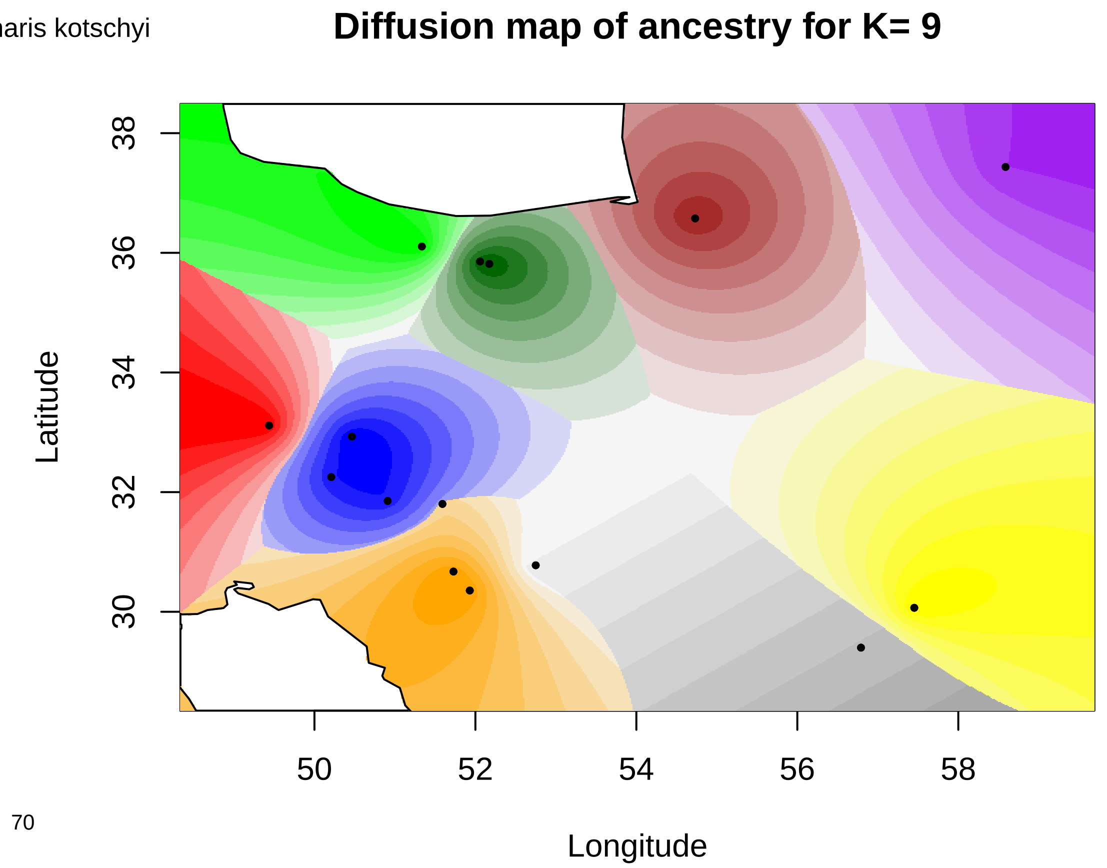

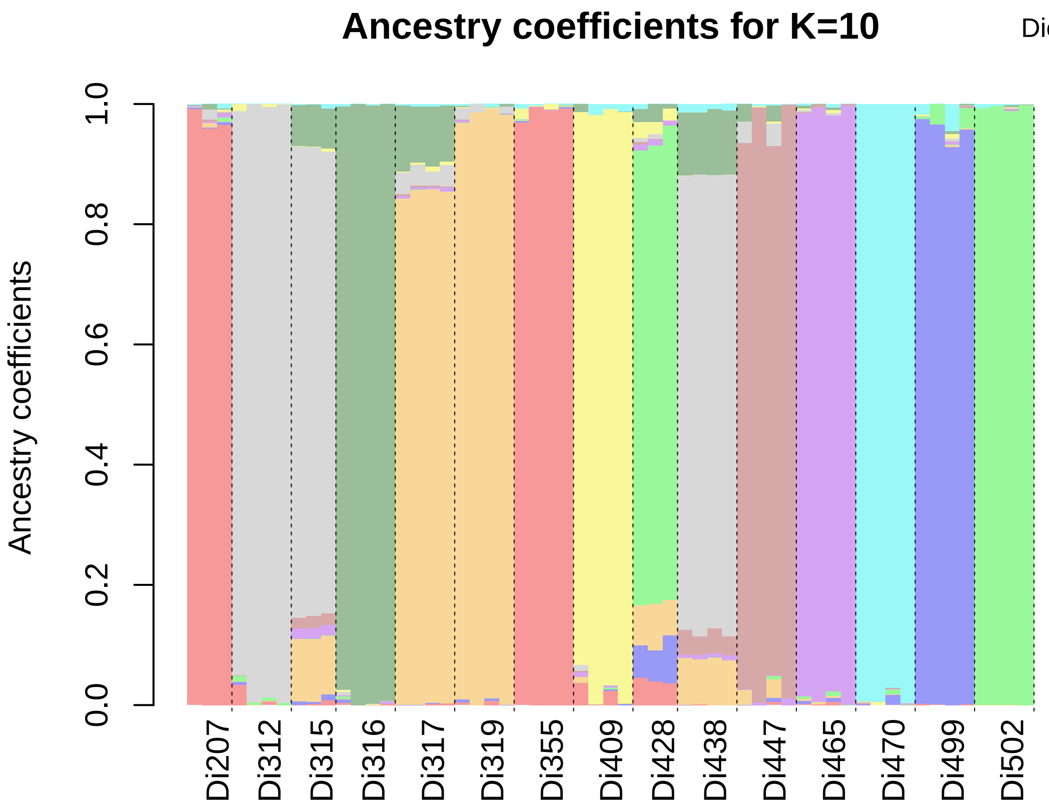

Dielsiocharis kotschyi

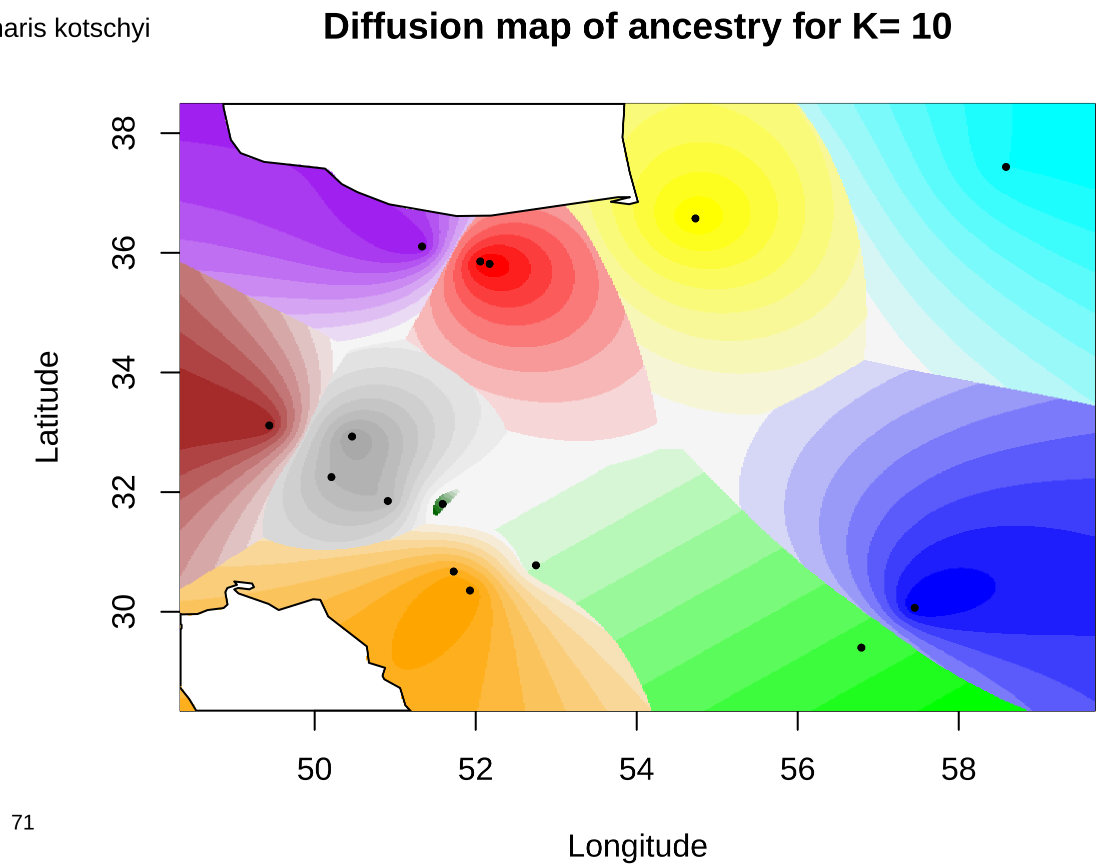

Physoptachys gnaphalodes

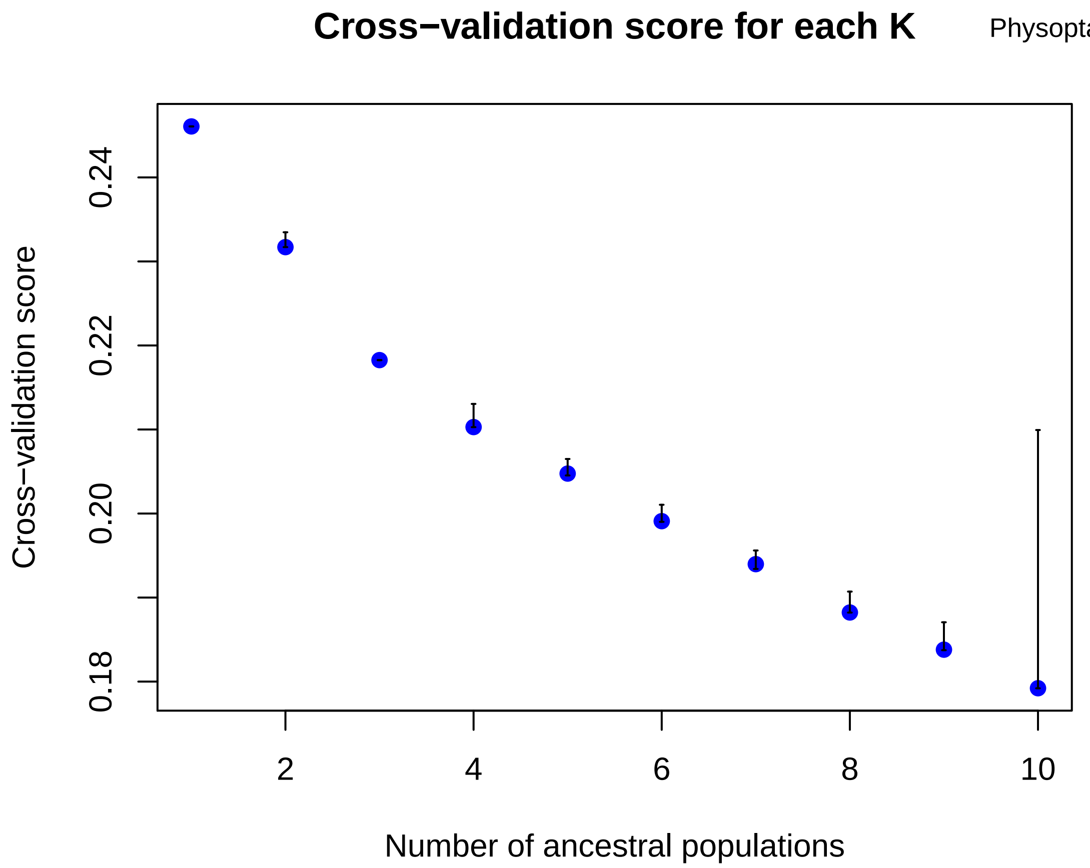

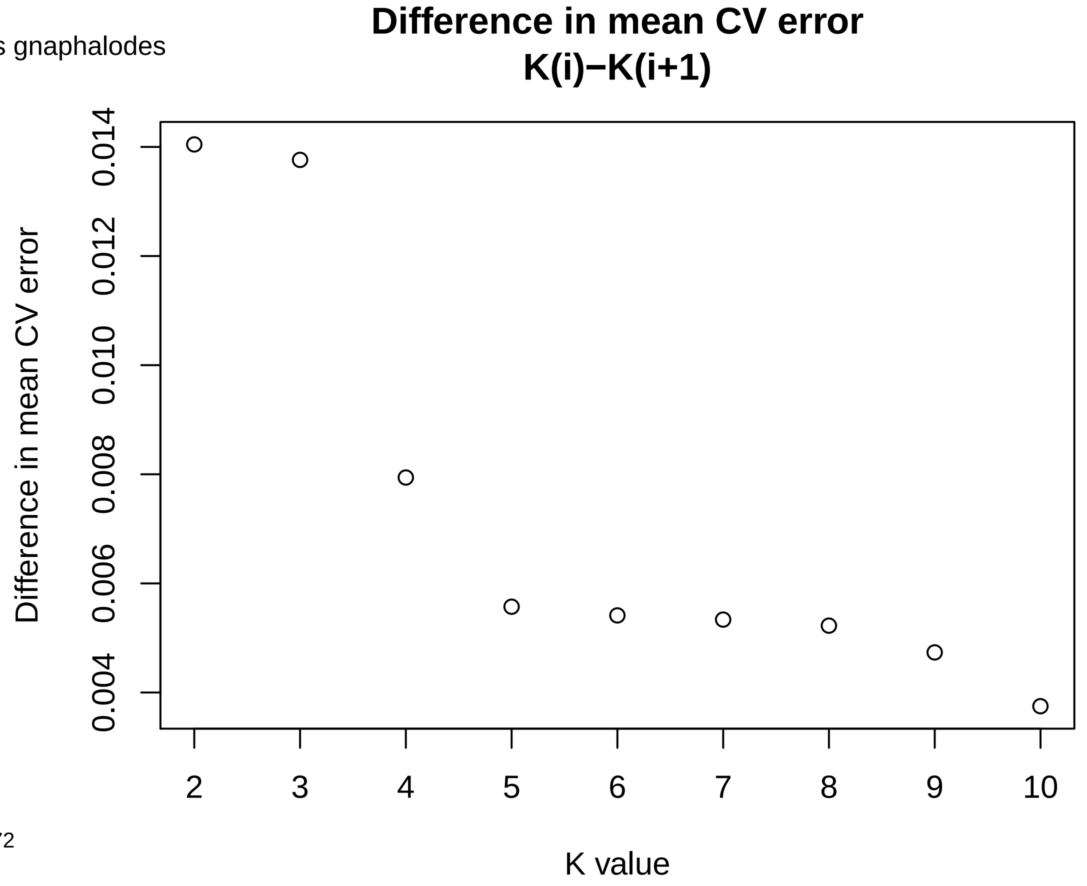

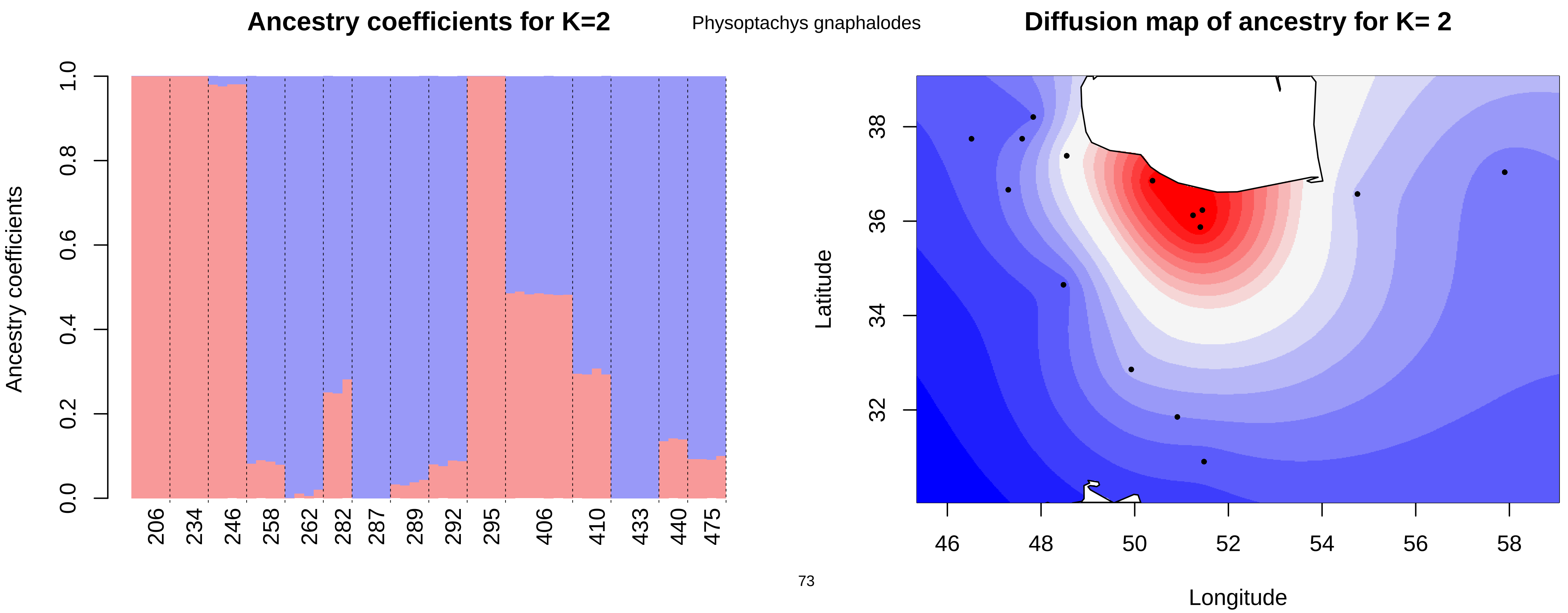
Ancestry coefficients for K=2
Physoptachys gnaphalodes
Diffusion map of ancestry for K= 2
Ancestry coefficients
0.0
0.2
0.4
0.6
0.8
1.0
206
234
246
258
262
282
287
289
292
295
406
410
433
440
475
Latitude
32
34
36
38
46
48
50
52
54
56
58
Longitude

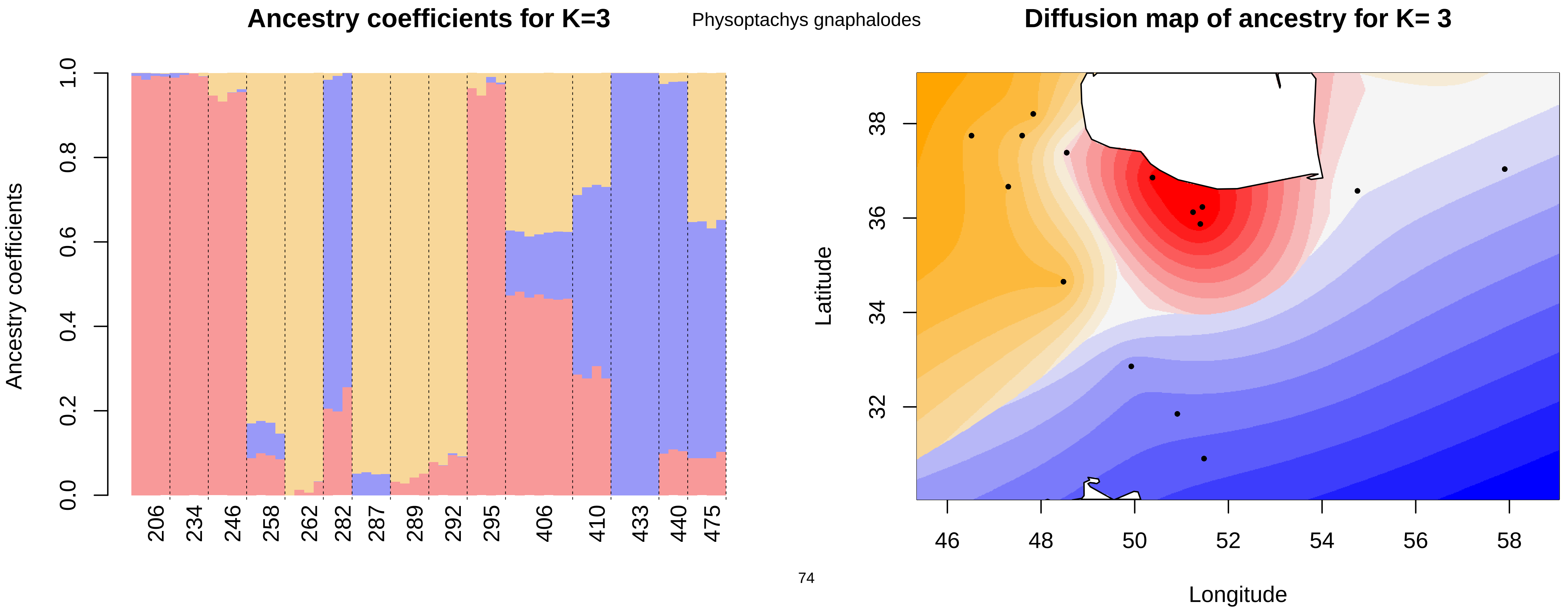
Ancestry coefficients for K=3
Physoptachys gnaphalodes
Diffusion map of ancestry for K= 3
Ancestry coefficients
0.0
0.2
0.4
0.6
0.8
1.0
206
234
246
258
262
282
287
289
292
295
406
410
433
440
475
Latitude
32
34
36
38
Longitude
46
48
50
52
54
56
58

Physoptachys gnaphalodes

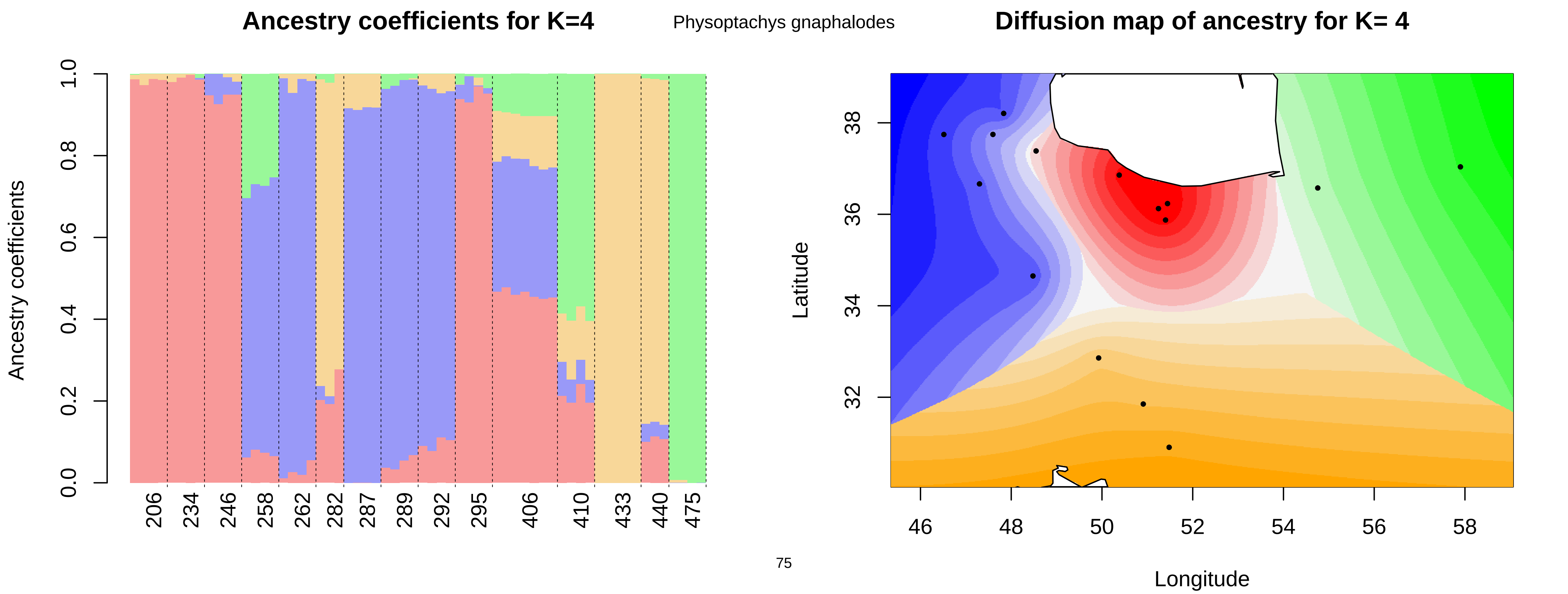

Physoptachys gnaphalodes

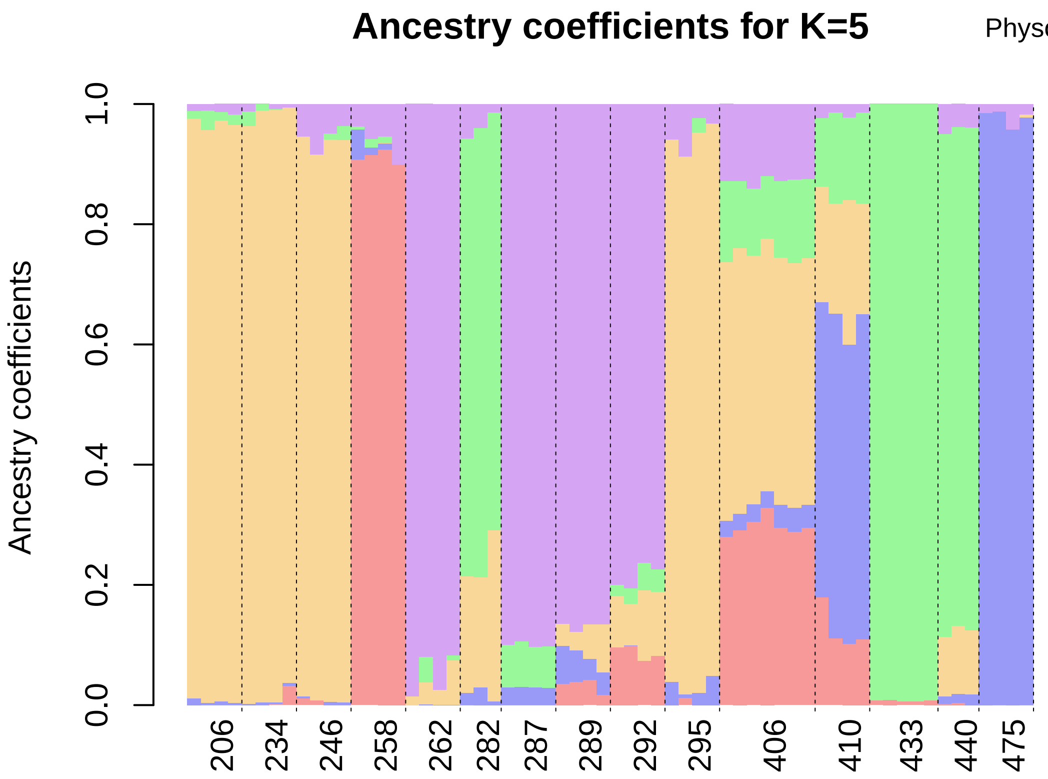

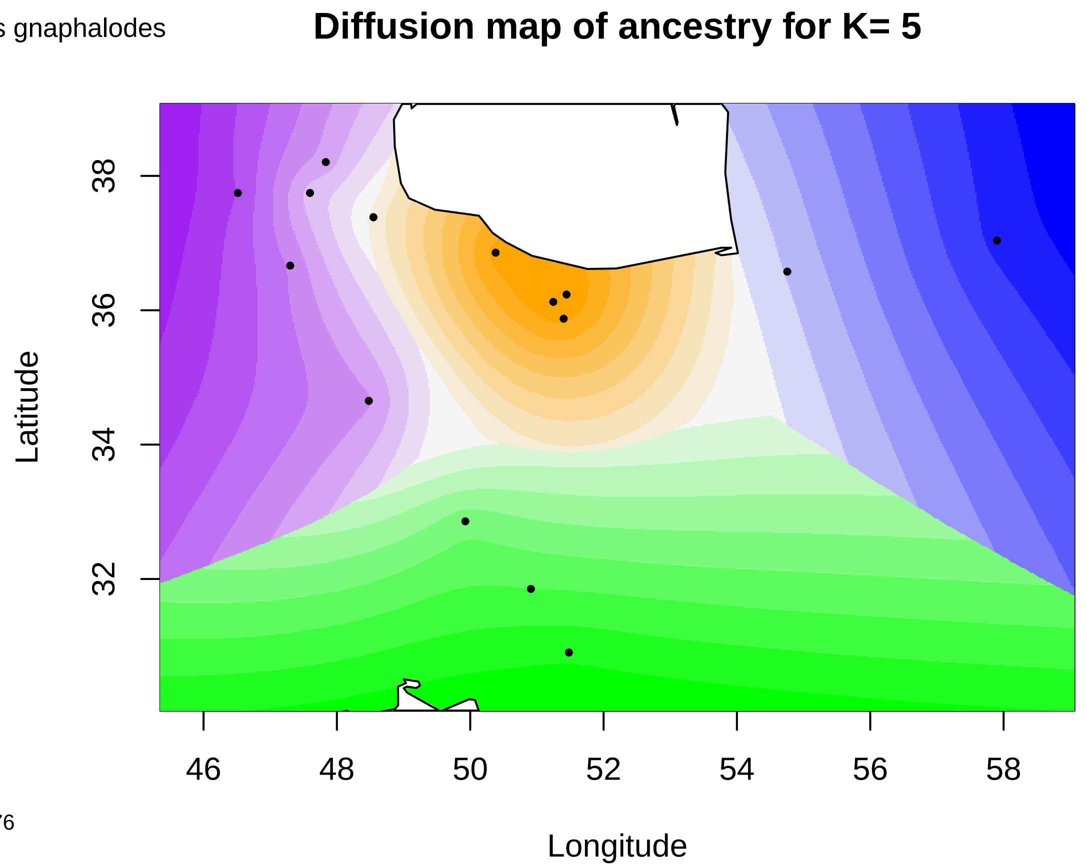

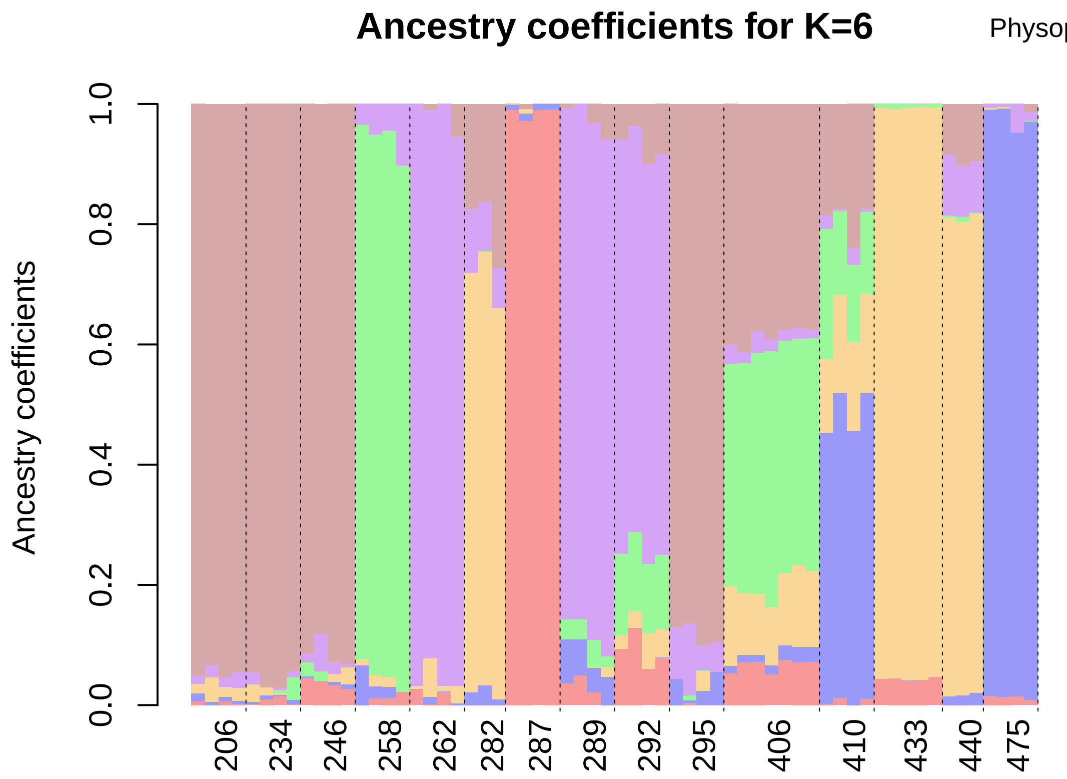

Physoptachys gnaphalodes

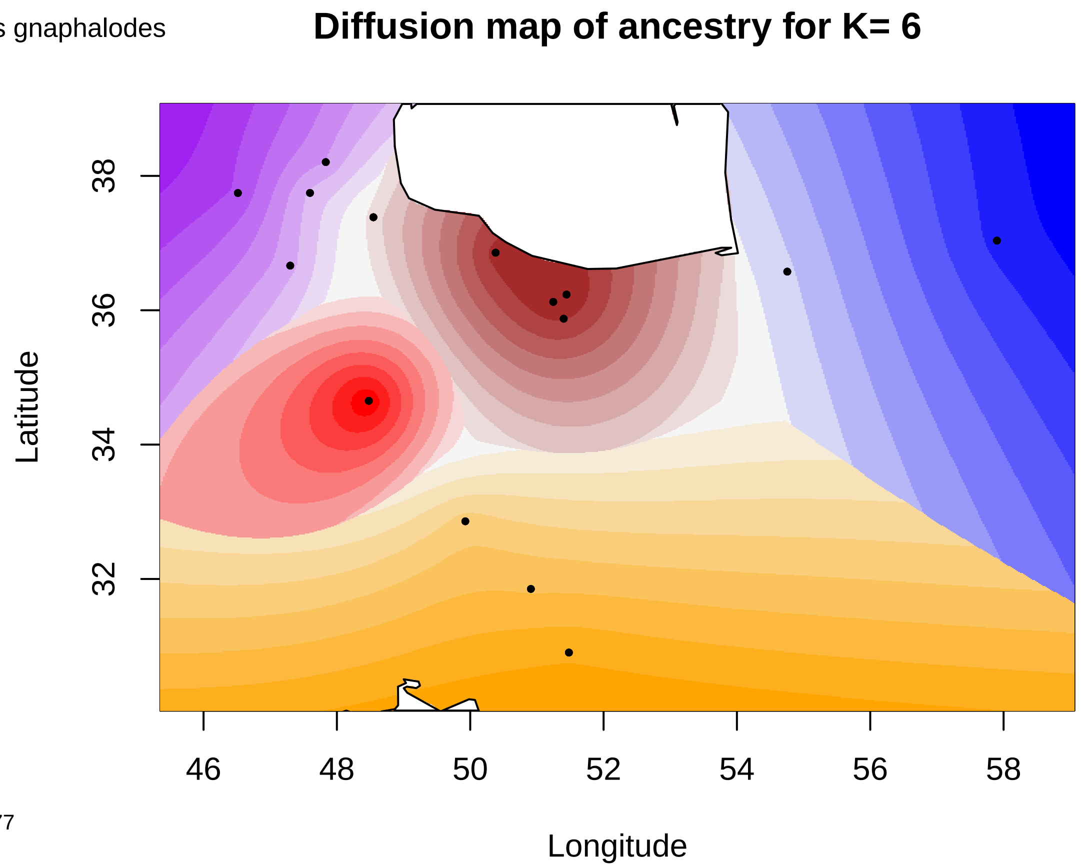

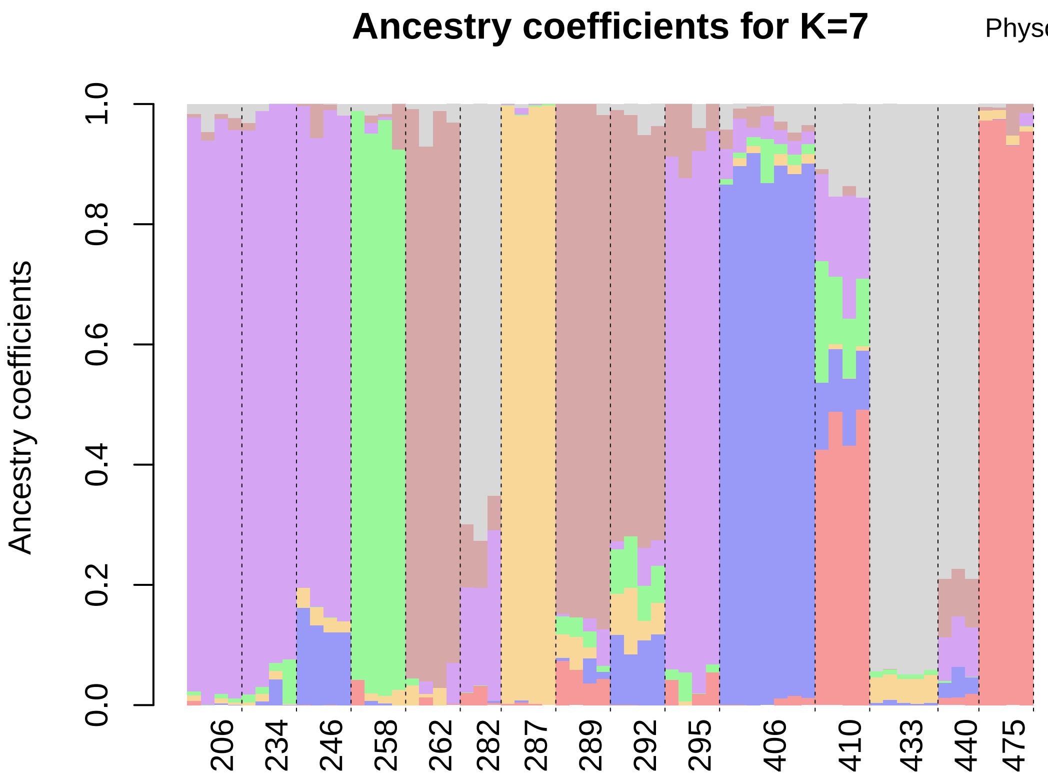

Physoptachys gnaphalodes

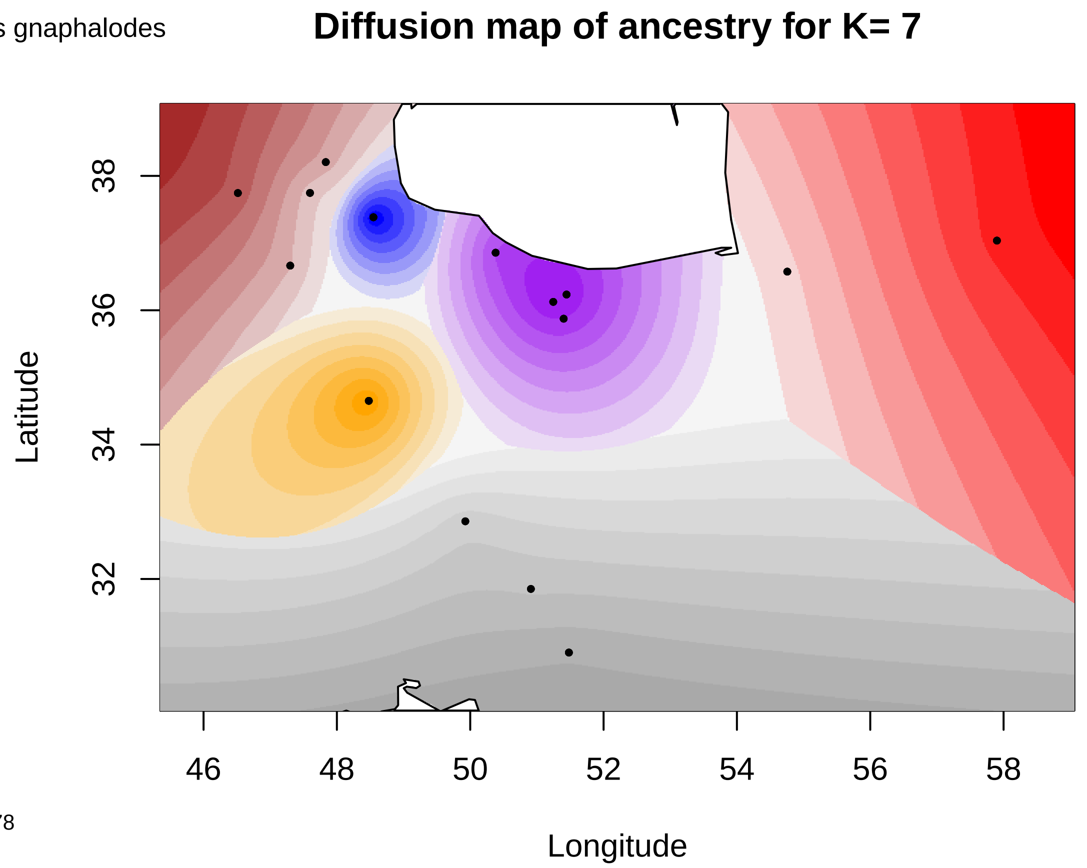

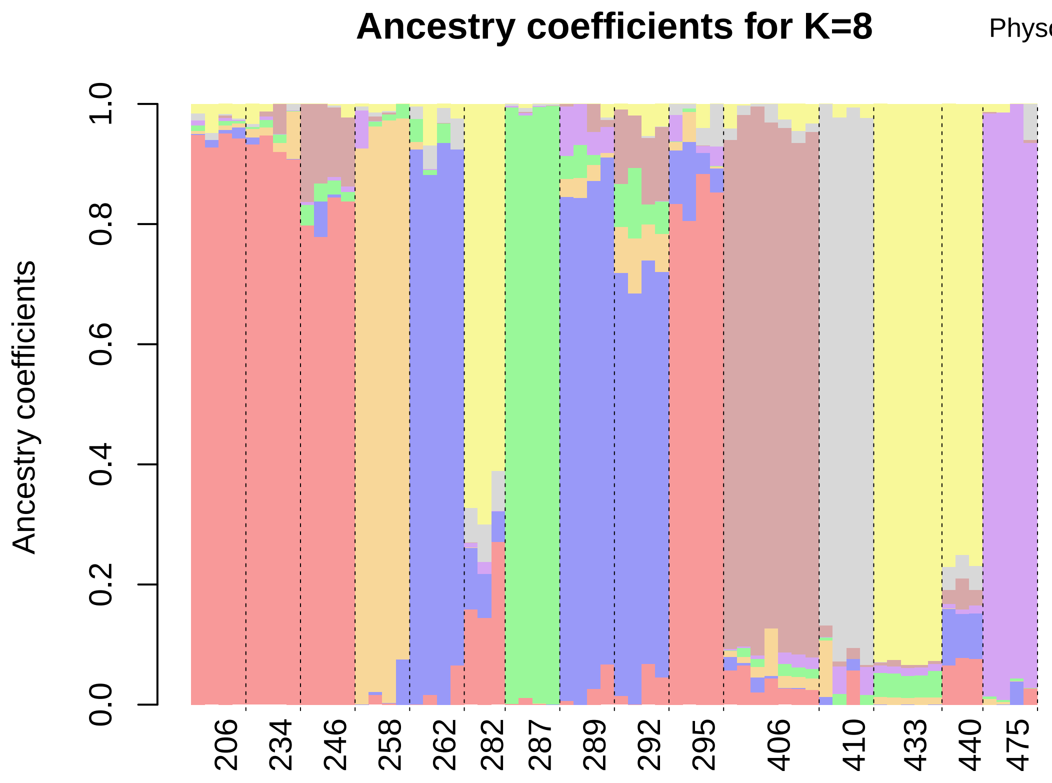

Physoptachys gnaphalodes

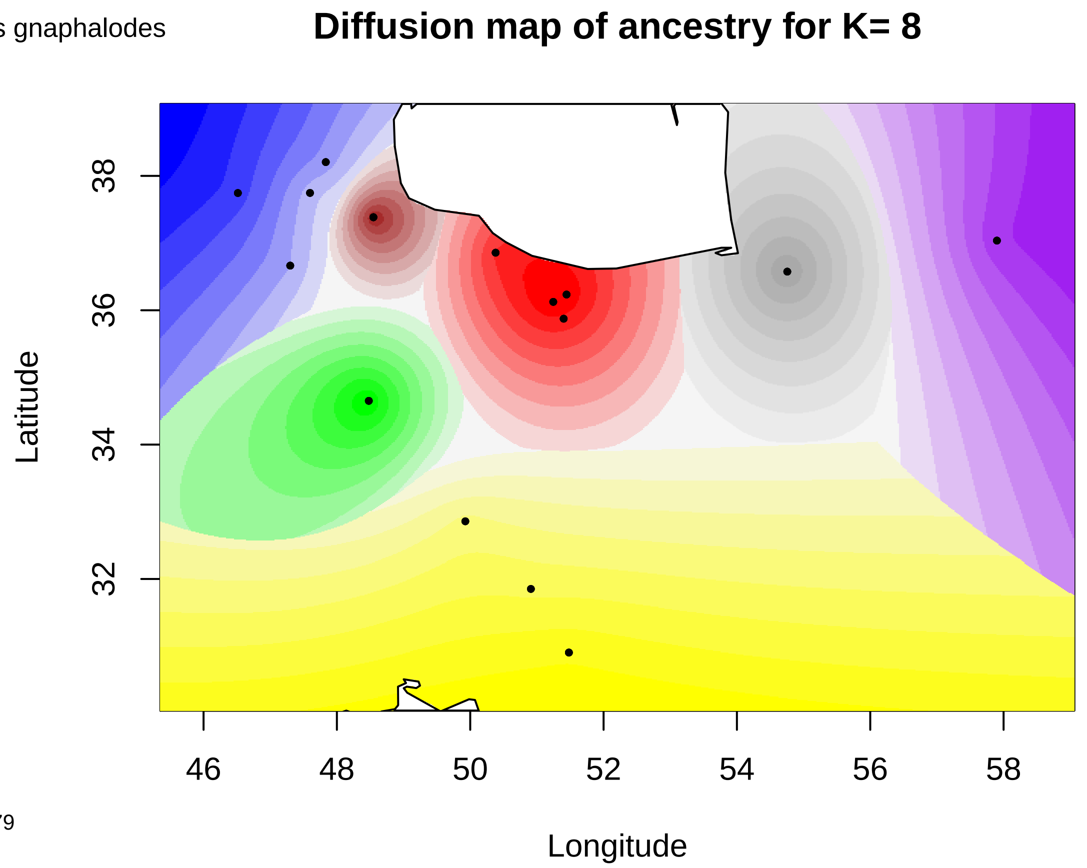

Physoptachys gnaphalodes

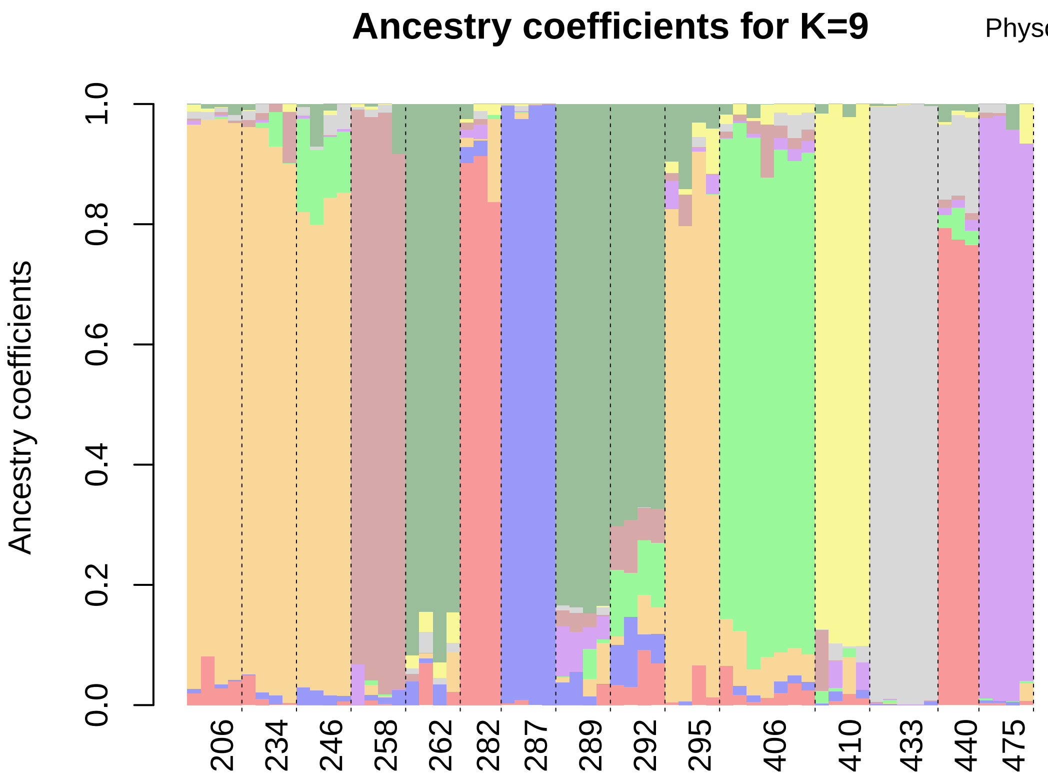

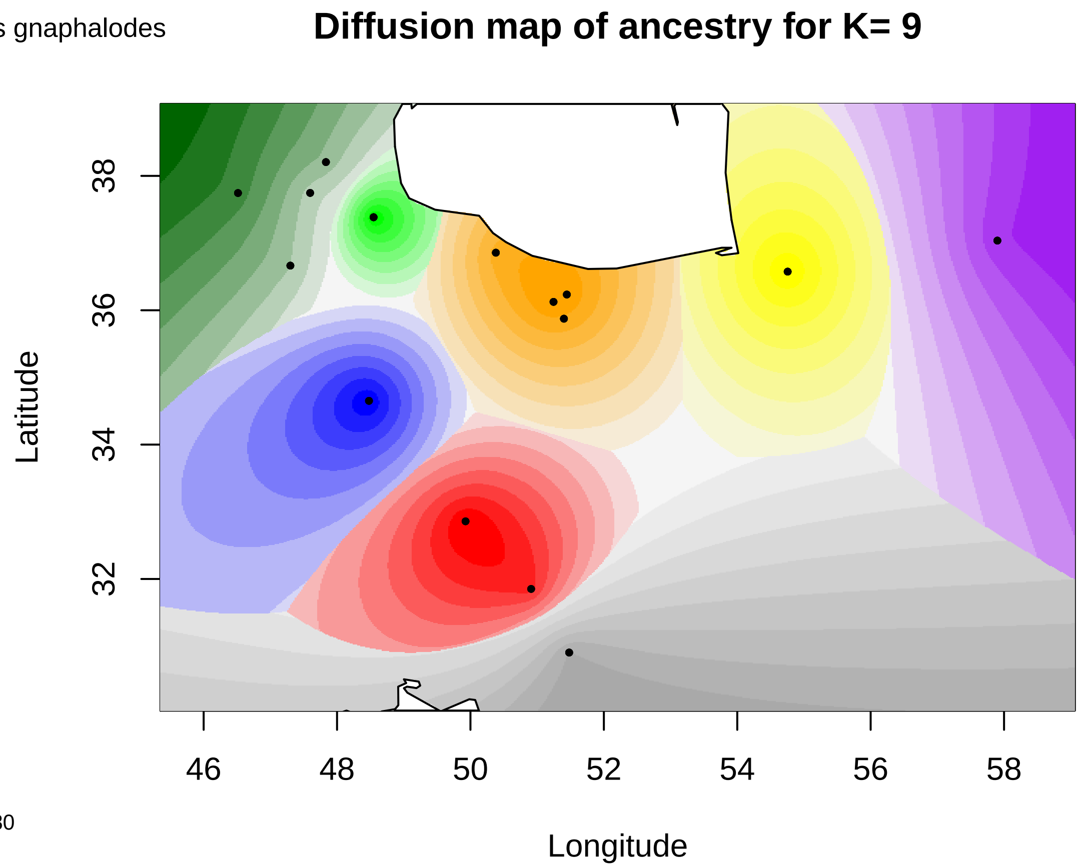

Physoptachys gnaphalodes

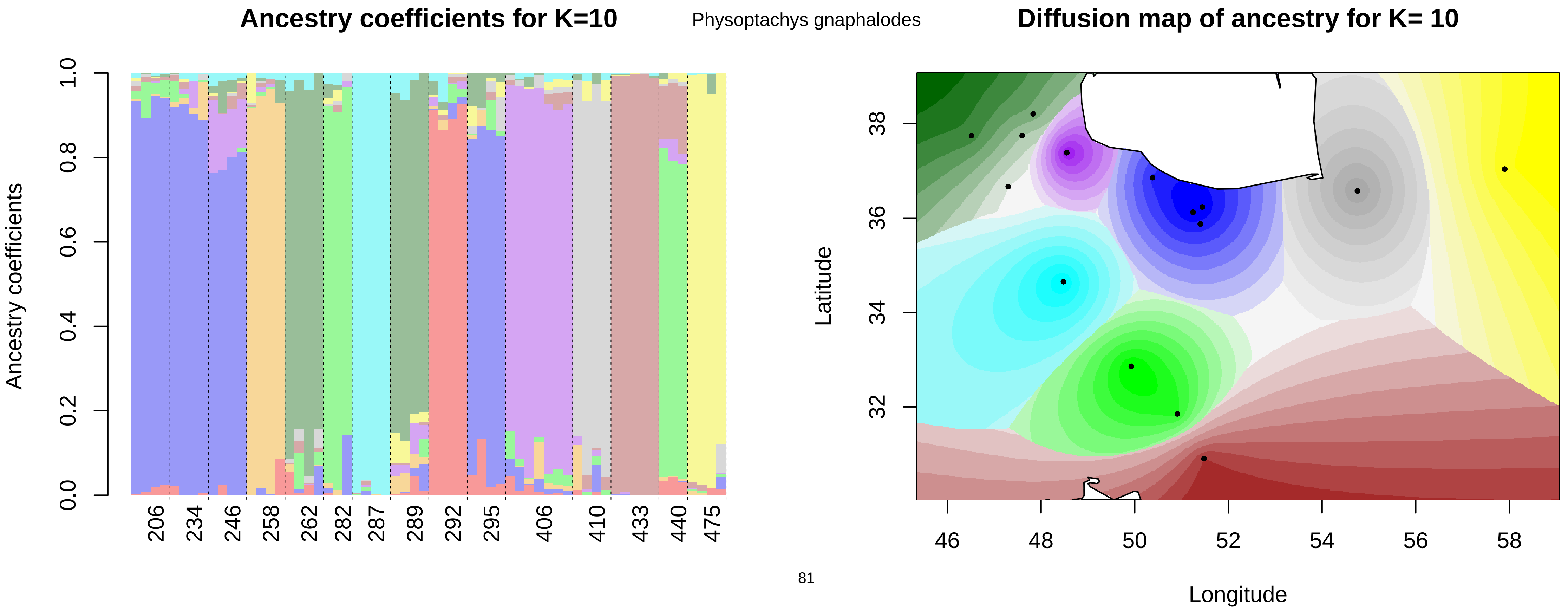

Tanacetum kotschyi

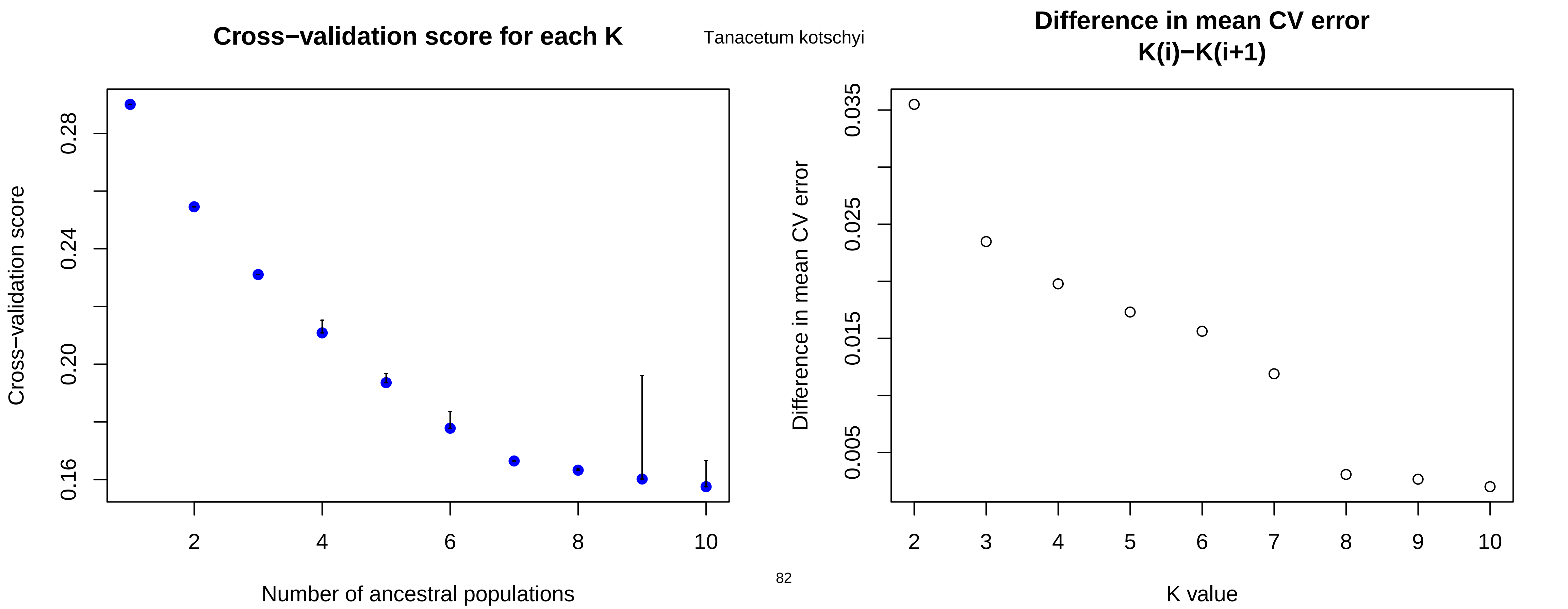

Tanacetum kotschyi

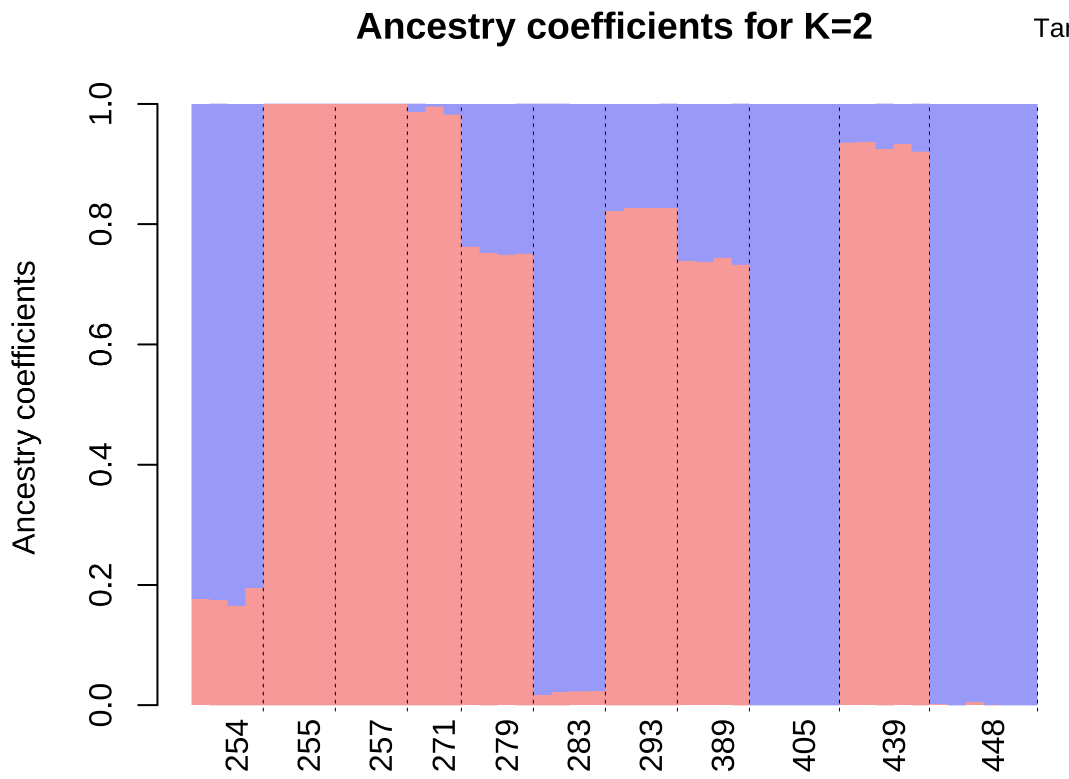

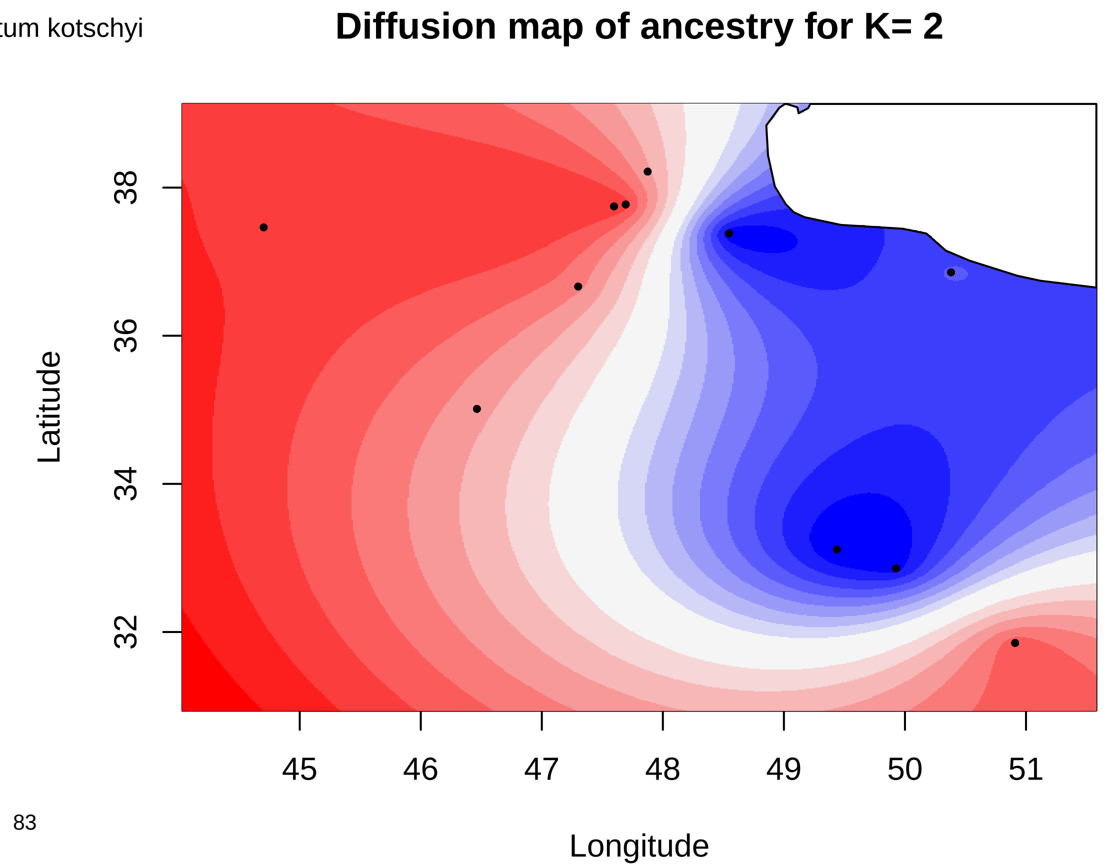

Tanacetum kotschyi

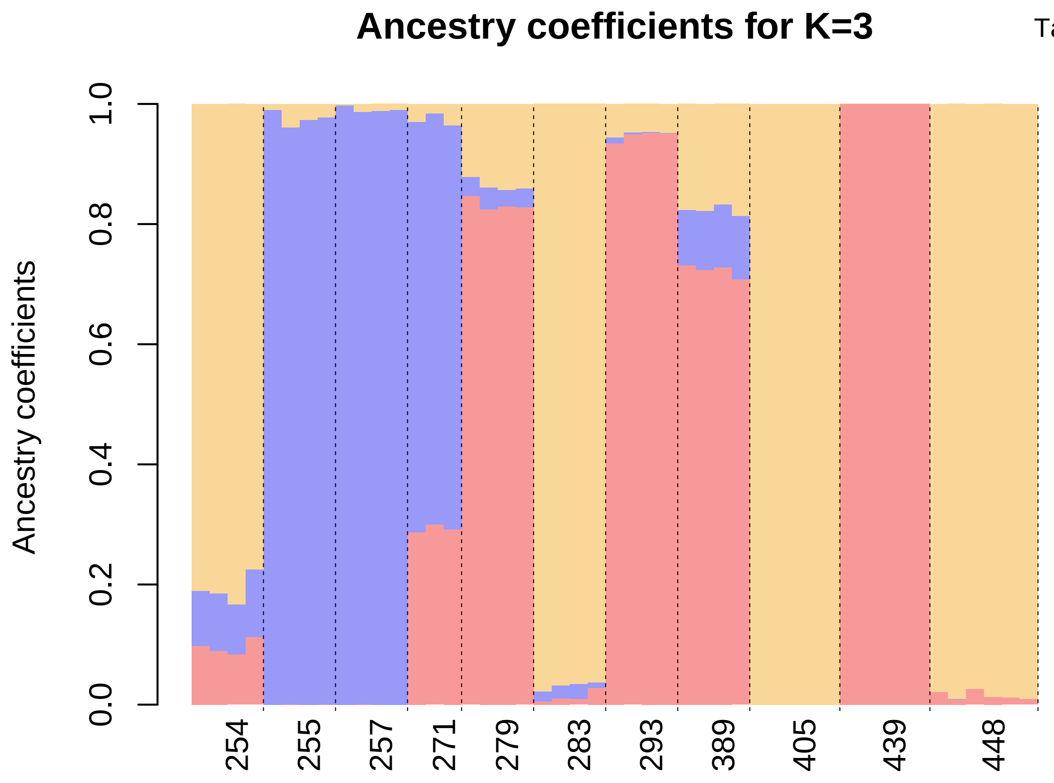

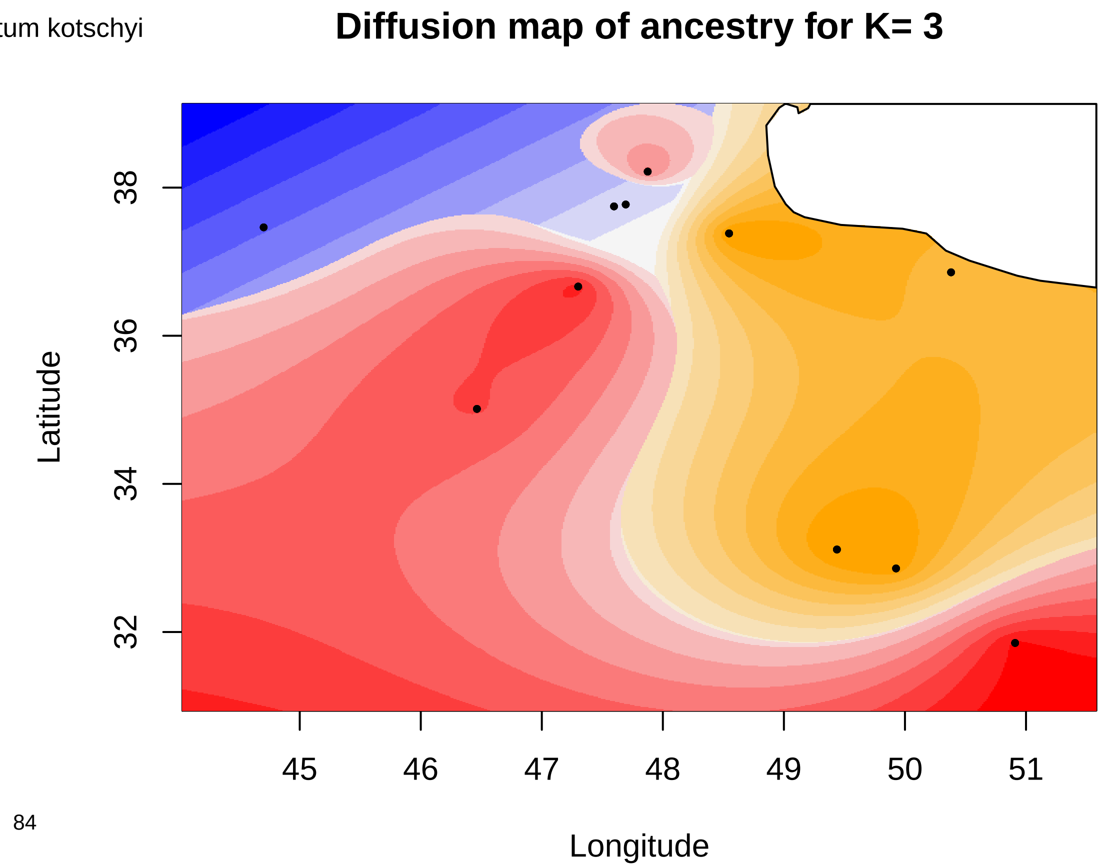

Tanacetum kotschyi

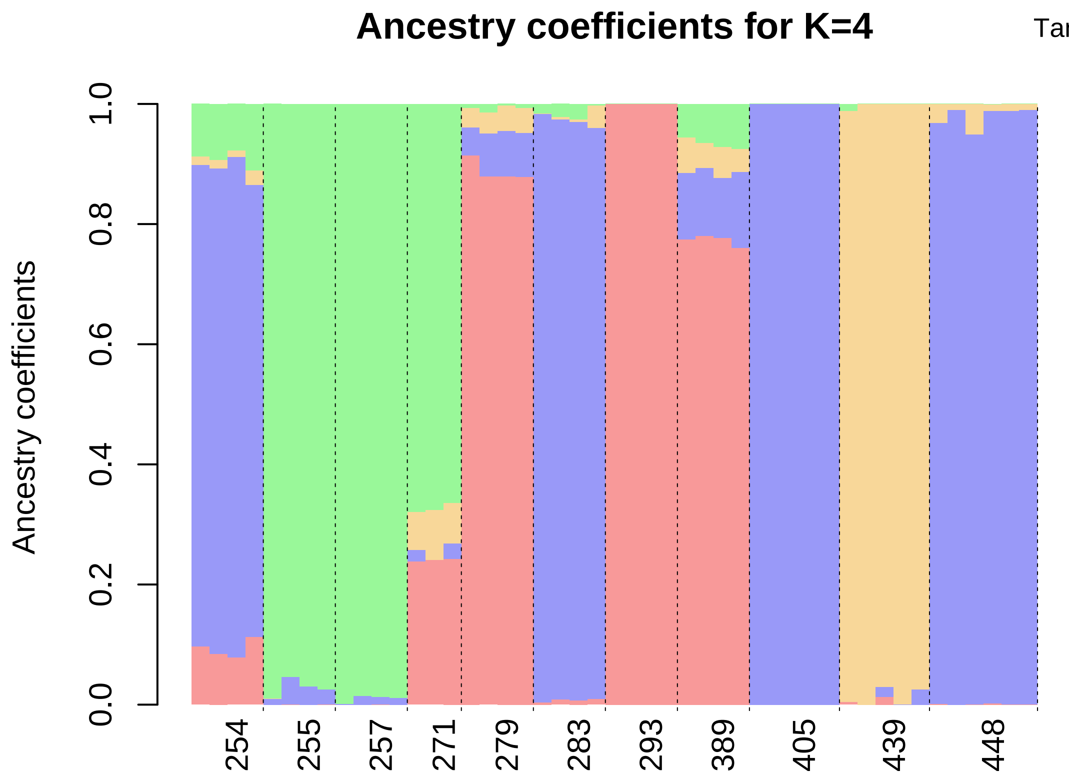

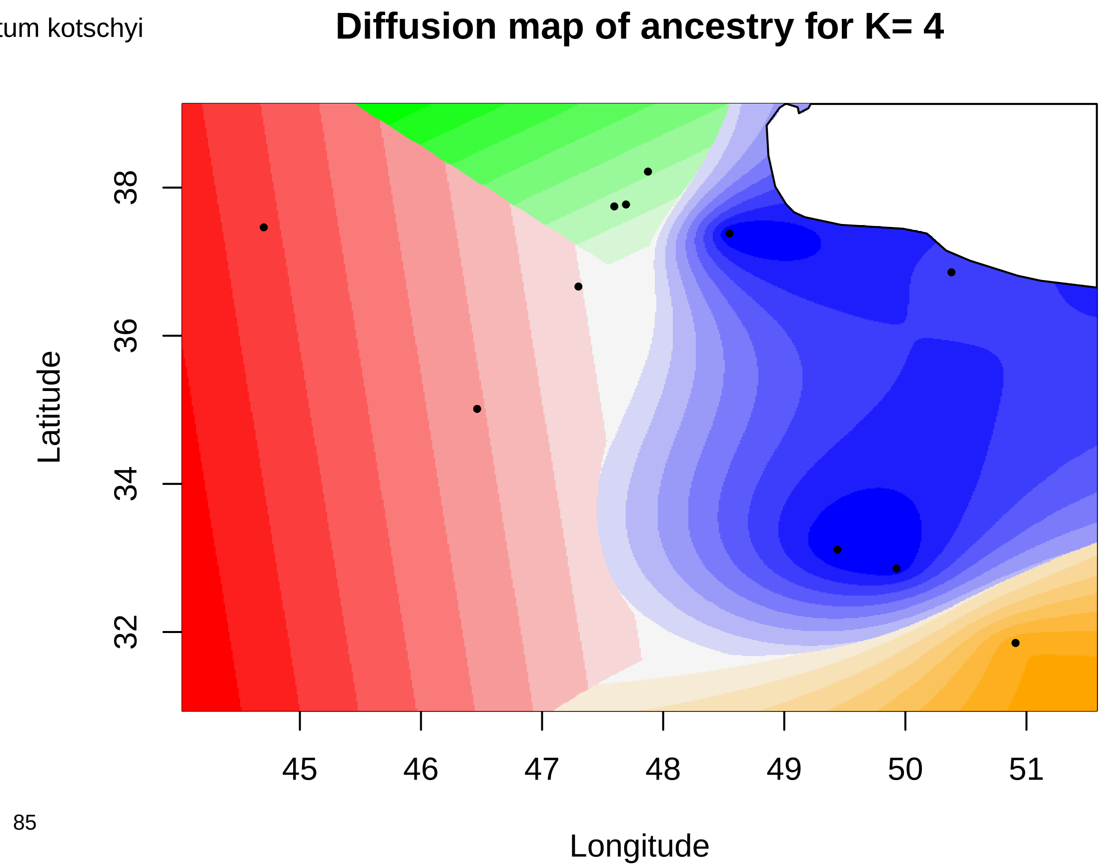

Tanacetum kotschyi

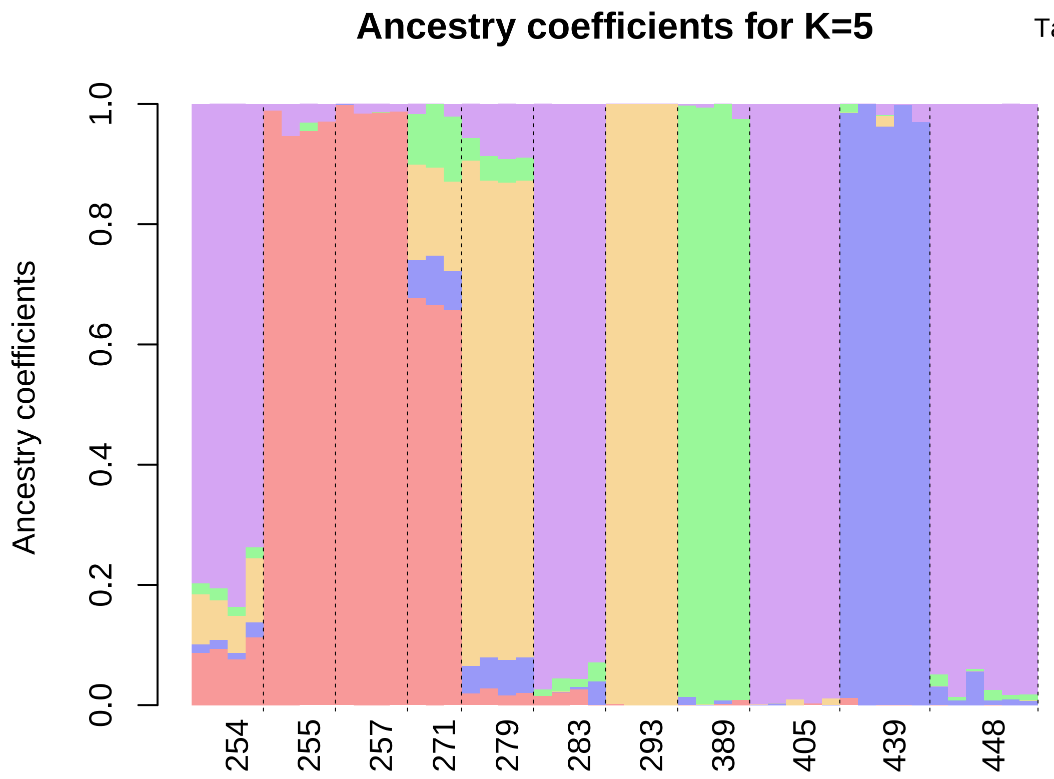

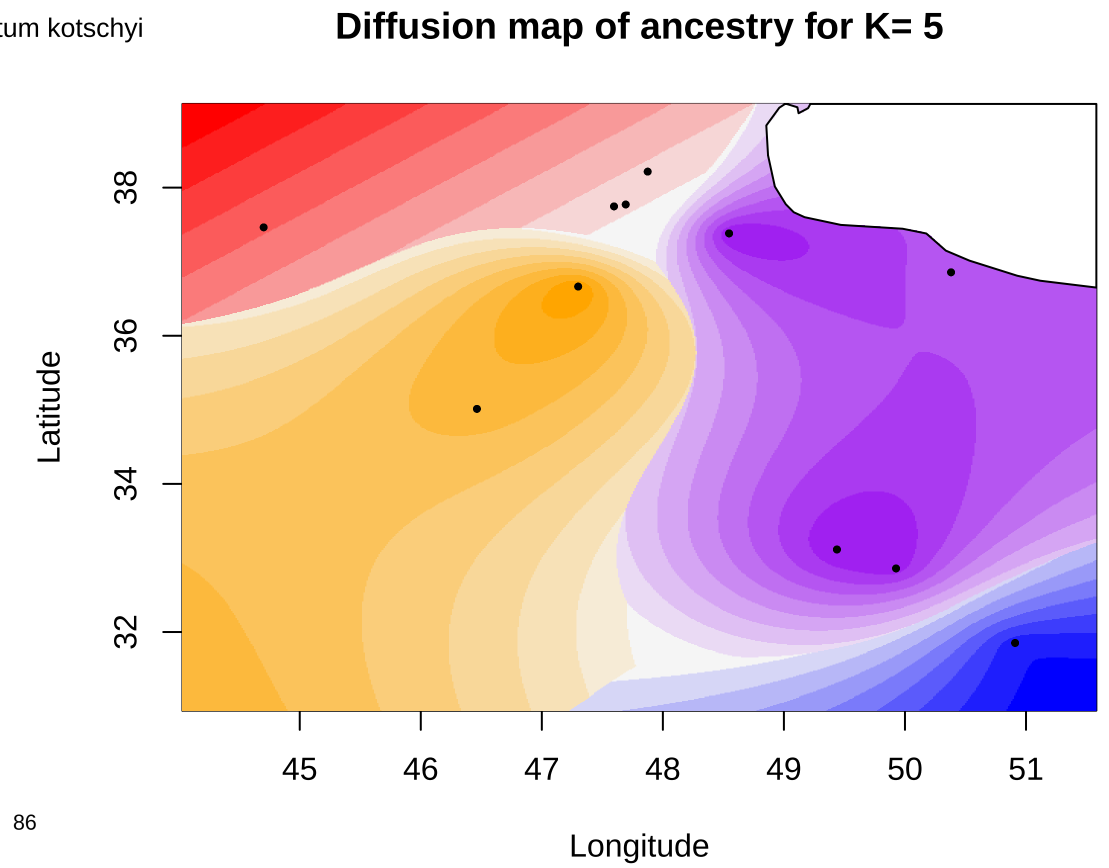

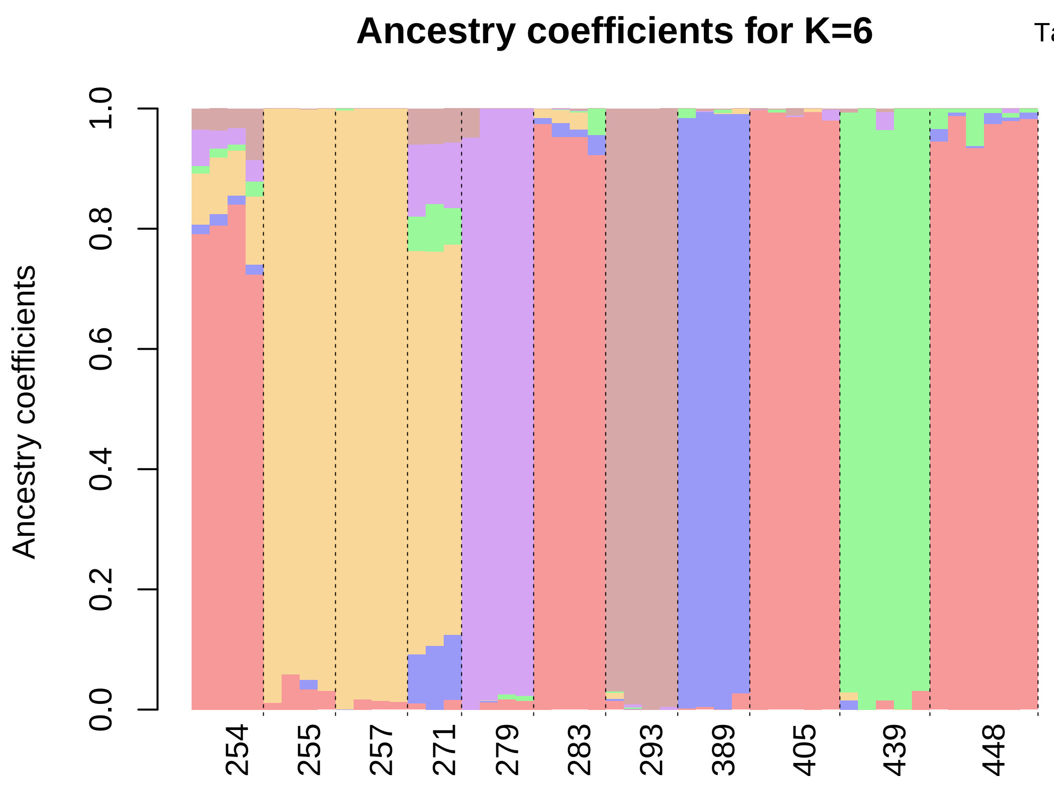

Tanacetum kotschyi

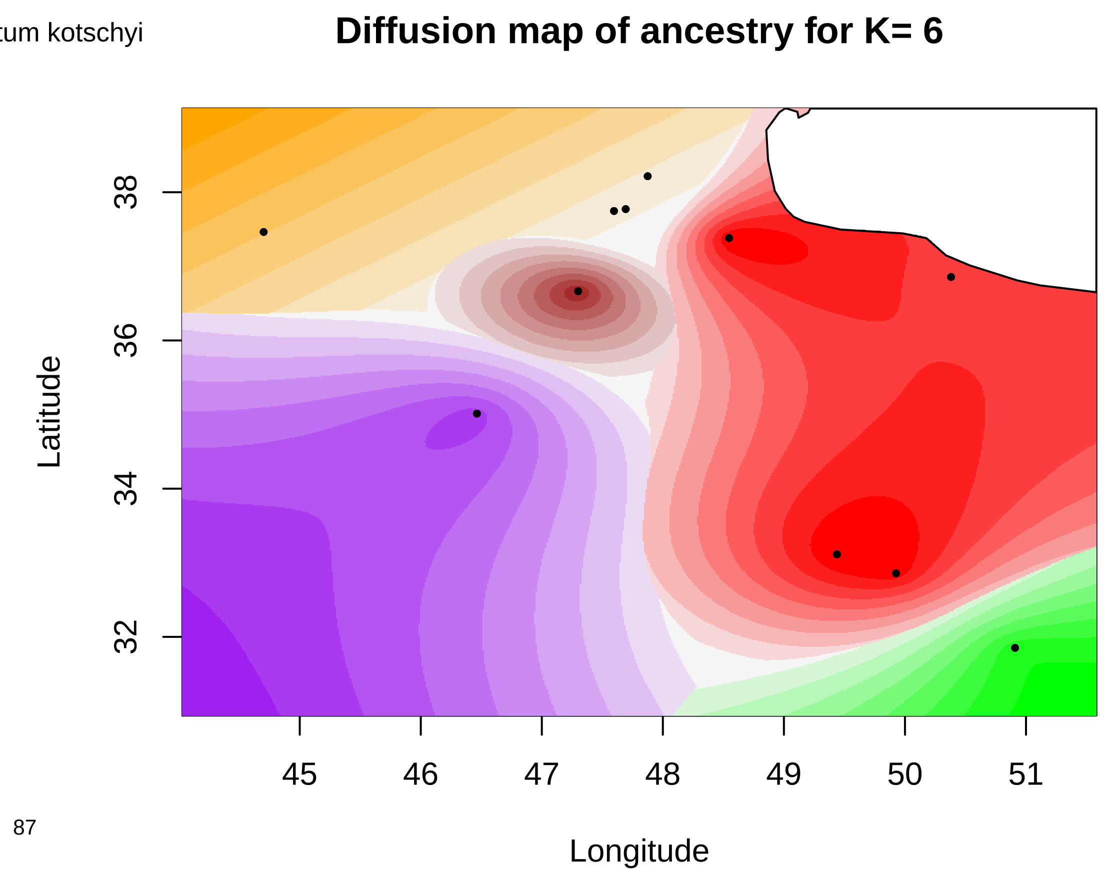

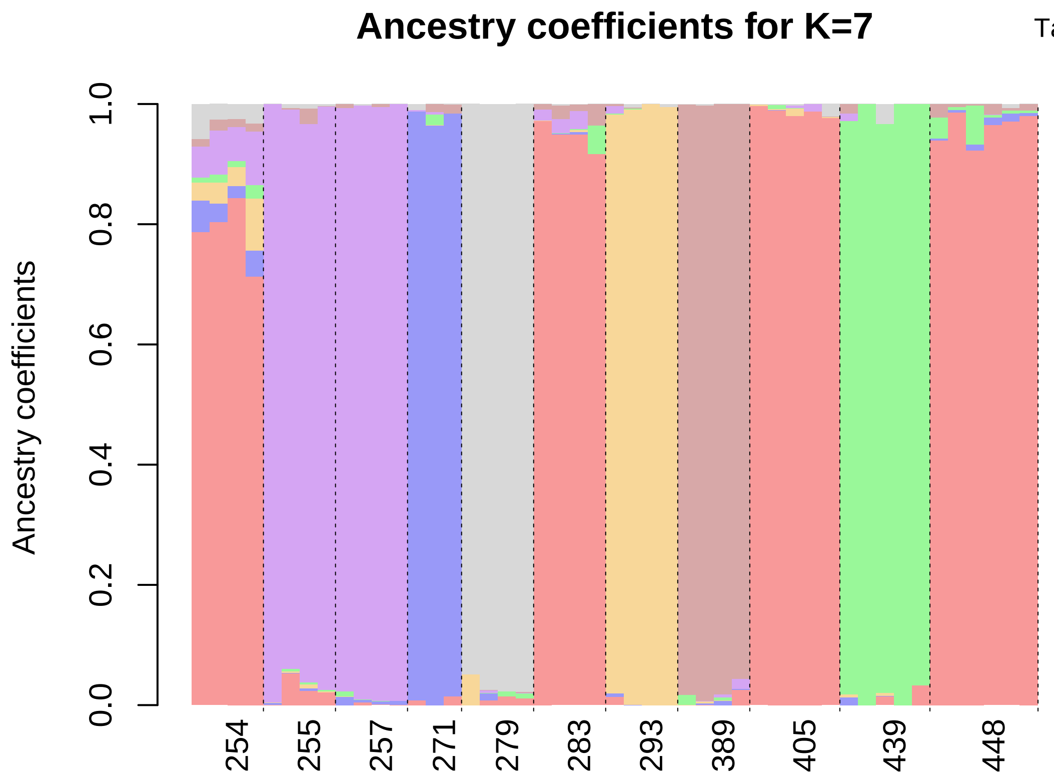

Tanacetum kotschyi

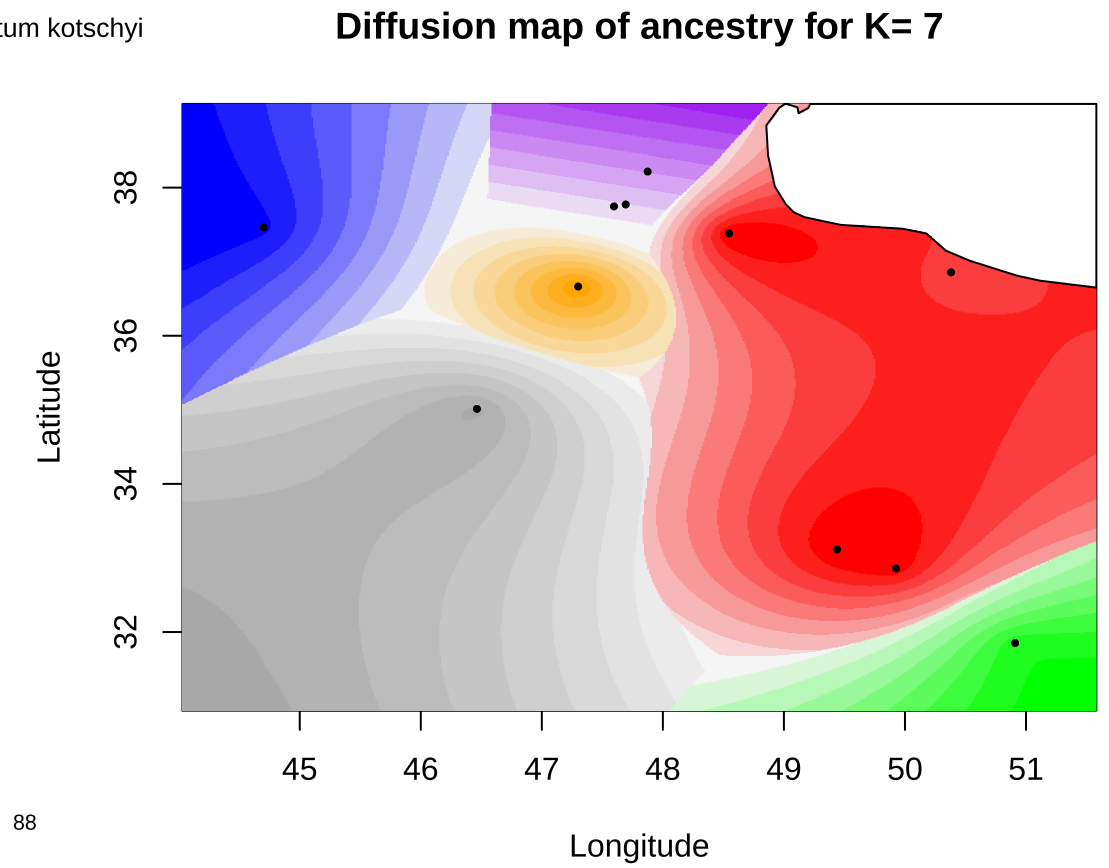

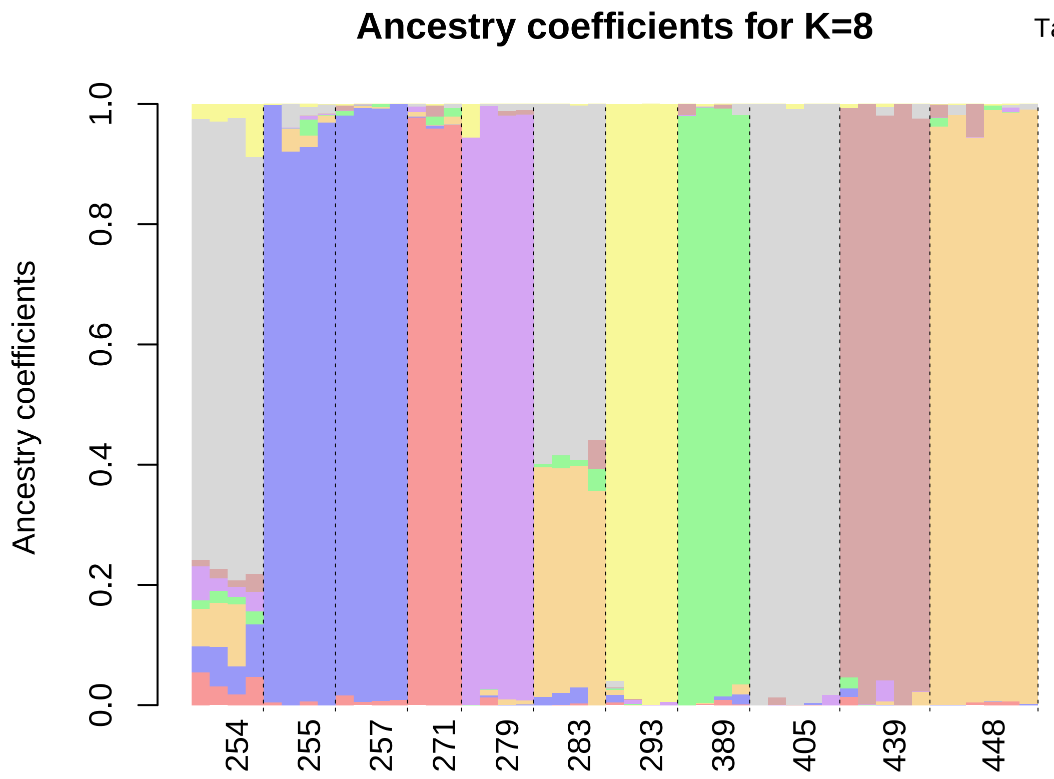

Tanacetum kotschyi

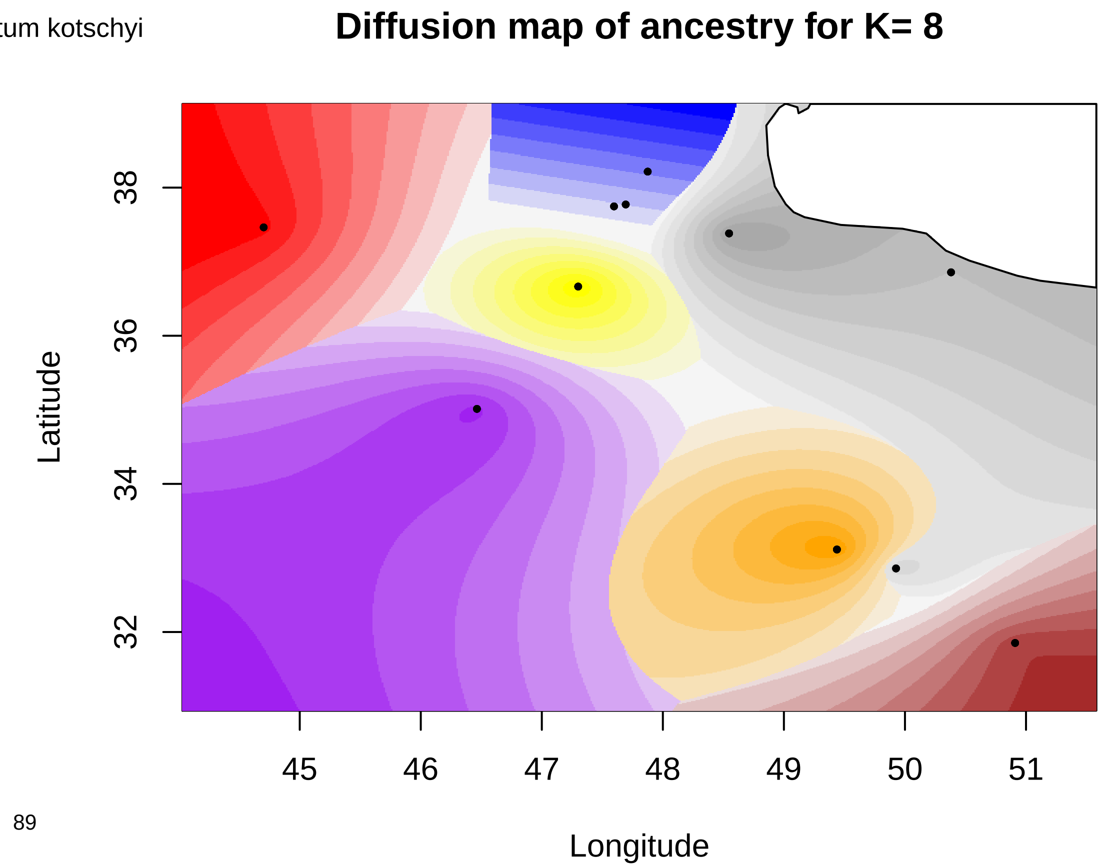

Tanacetum kotschyi

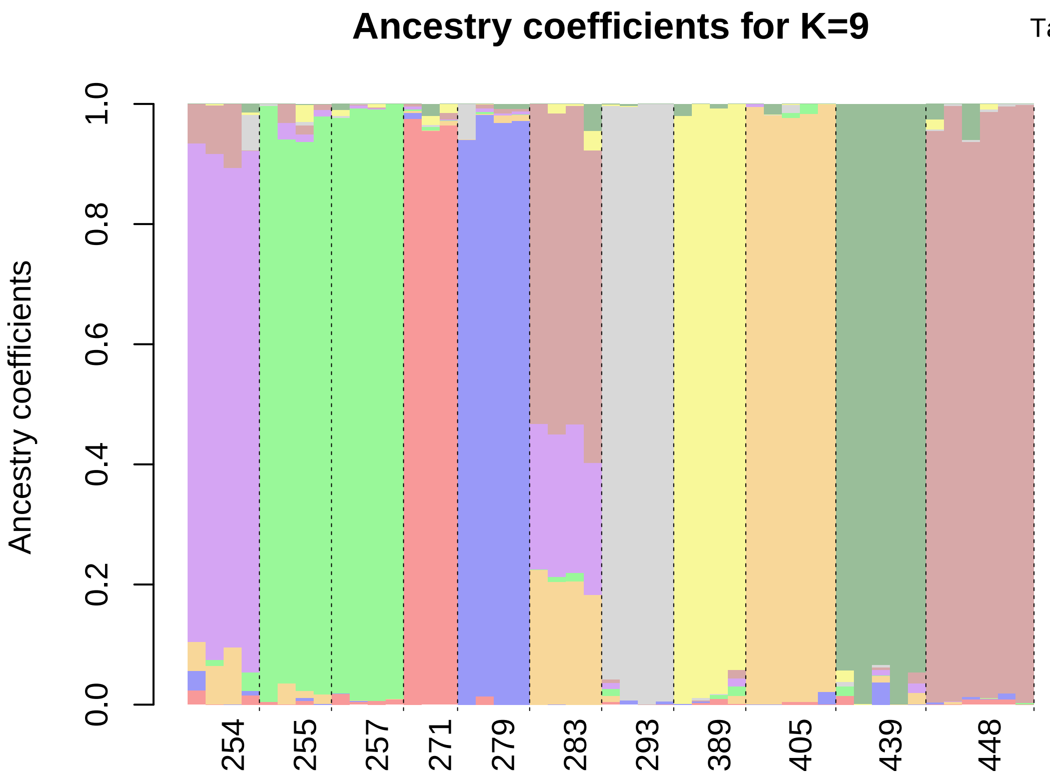

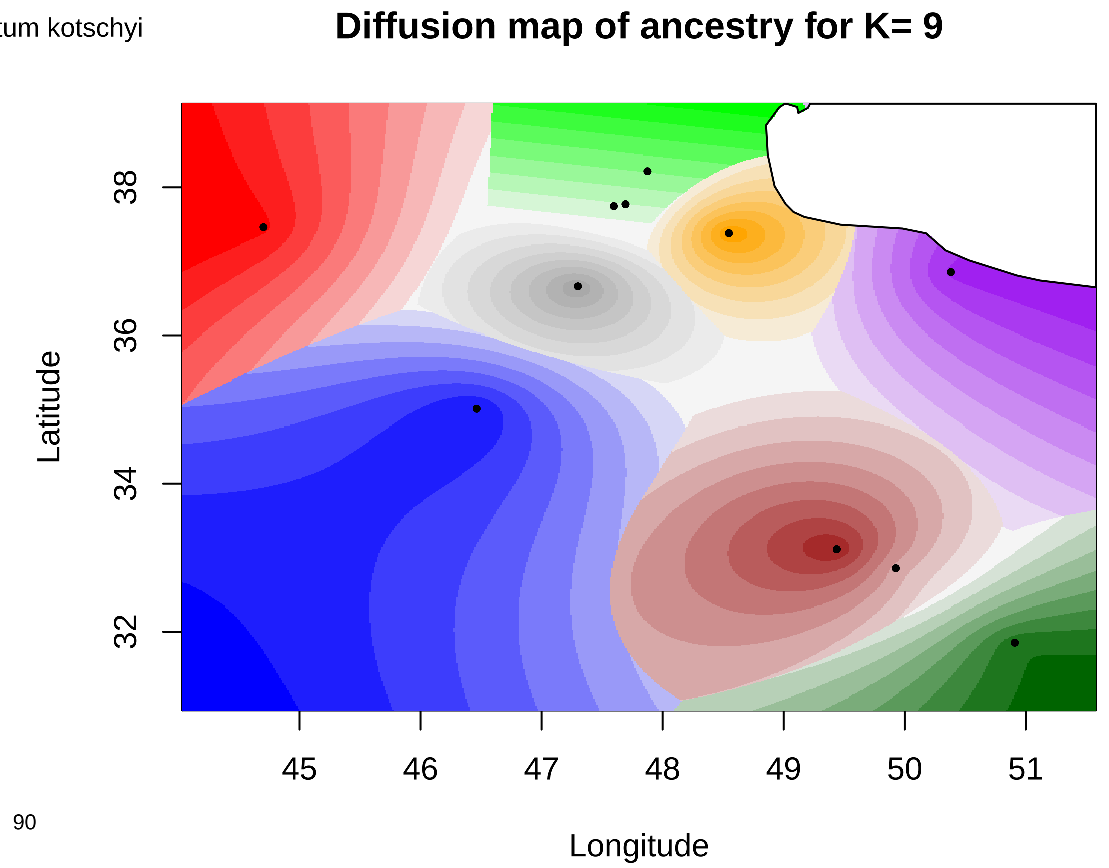

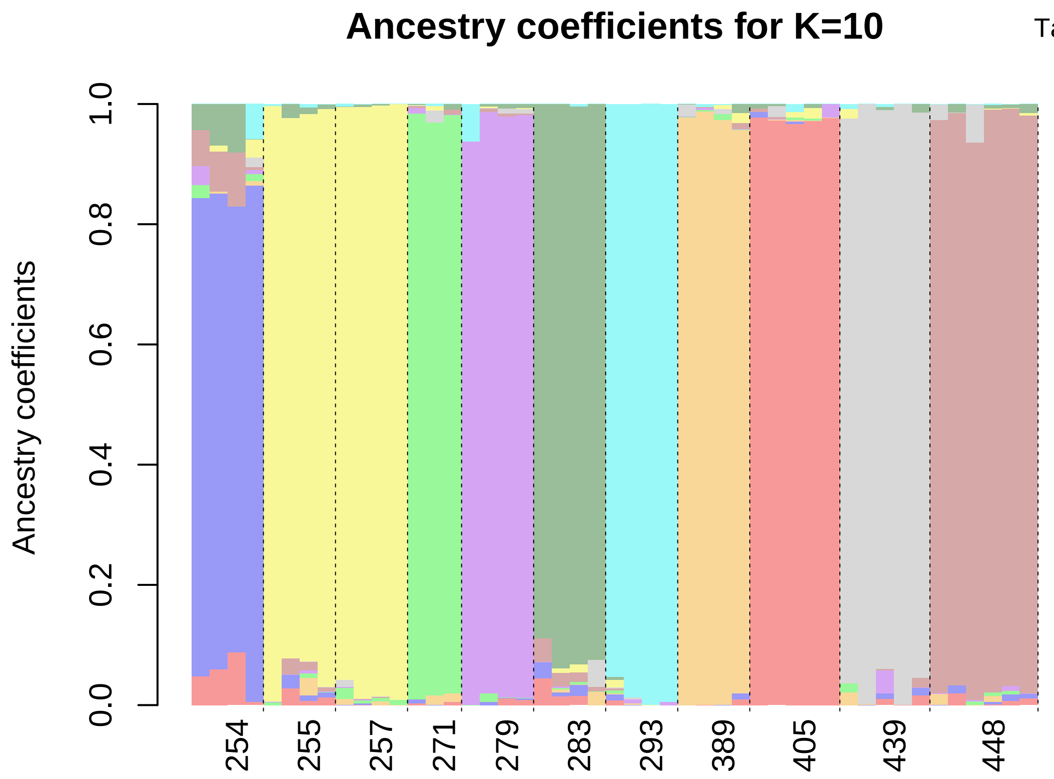

Tanacetum kotschyi

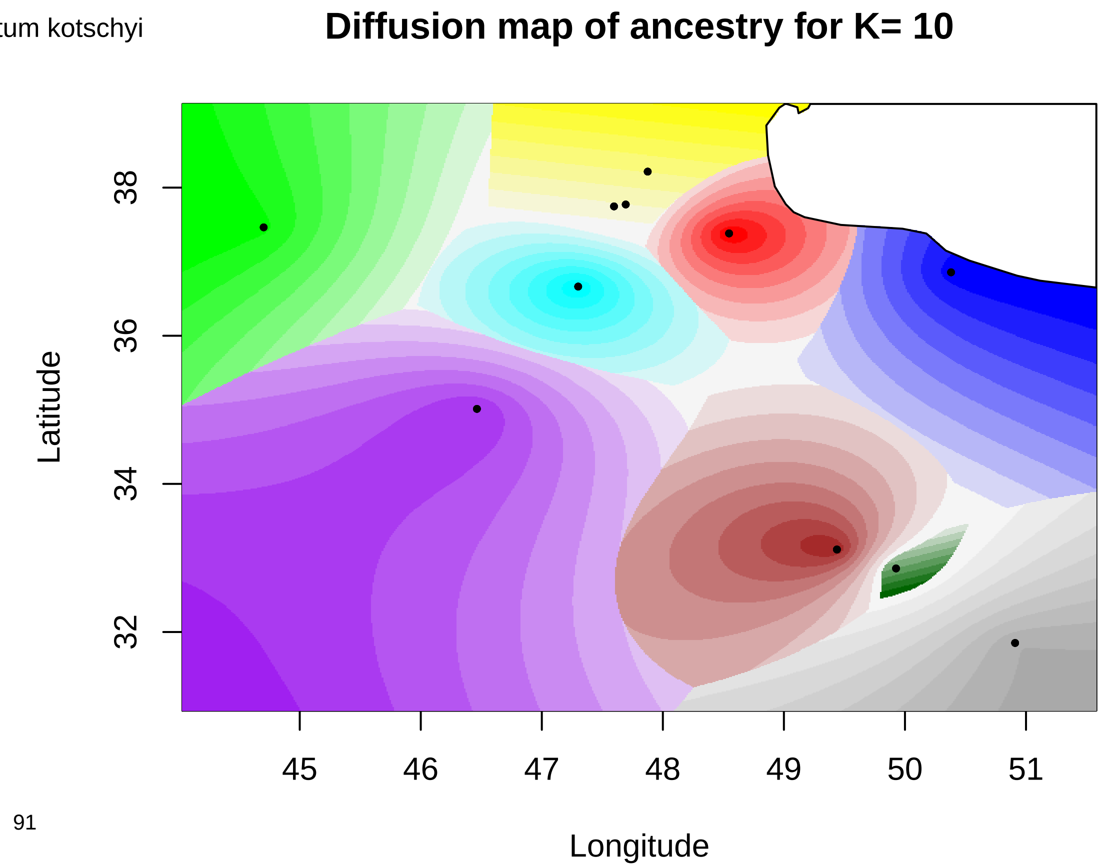

Supplement: Supplementary file 3 — Figure S1: Results of TESS3 analyses. For each species, the following information is shown: plot of the cross‐validation score across different K values (1–10); plot of the difference in mean cross validation error across different K values (2–10); histogram of ancestry coefficients for different K values (2–10); diffusion map of the genetic groups for different K values (2–10). Figure S2: Neighbour‐nets based on HKY distances (see main text for details). Colors of population numbers correspond to those used for genetic groups identified by TESS3 (see Figure 2; strongly admixed populations shown in grey). Species are arranged according to elevational zone (montane species, alpine species) and, within each zone, alphabetically. Figure S3: Principal component analysis (PCA). Colors of population numbers and of polygons delimiting genetic groups correspond to those used for genetic groups identified by TESS3 (see Figure 2; strongly admixed populations shown in grey). Species are arranged according to elevational zone (montane species, alpine species) and, within each zone, alphabetically. Figure S4: Phylogeographic barriers identified using Monmonier's maximum difference algorithm on population networks (left column: Delaunay triangulation; right column: Gabriel graph), where population distances are calculated from Nei distances (upper row) or Roger's distance (lower row; see main text for details). Arrows indicate the directionality of the path (from larger to smaller distances), different colors (blue, red) indicating distinct paths with the same starting point. Edges in orange (in Allium scabriscapum only) indicate a set of edges jointly corresponding to a single genetic boundary geographically placed at the lowlands between Alborz (north) and Zagros Mountains (south). Species are arranged according to elevational zone (montane species, alpine species) and, within each zone, alphabetically. Table S1: List of populations of all species, their collected locations, da [file MEC-35-e70355-s001.zip › Figure_S1.pdf]
