# Supplementary material for: Phylogeographic Patterns Are Strongly Associated With Biogeographic Patterns in the Irano‐Anatolian Global Biodiversity Hotspot
Source: Mol Ecol. 2026 Apr 29;35:e70355. doi: 10.1111/mec.70355 (PMC13126619; doi:10.1111/mec.70355)

Fig. S2. Neighbour-nets based on HKY distances (see main text for details). Colors of population numbers correspond to those used for genetic groups identified by TESS3 (see Figure 2; strongly admixed populations shown in grey). Species are arranged according to elevational zone (montane species, alpine species) and, within each zone, alphabetically.

Allium scabriscapum

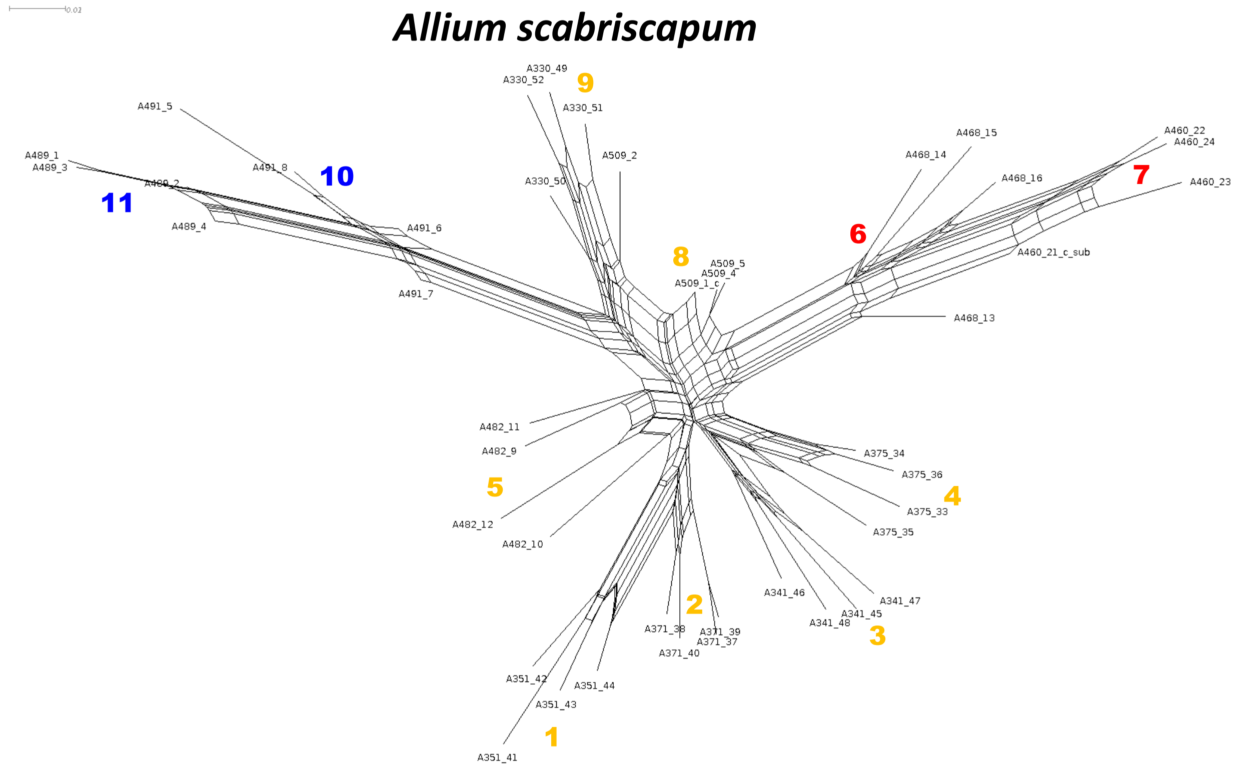

Helichrysum oligocephalum

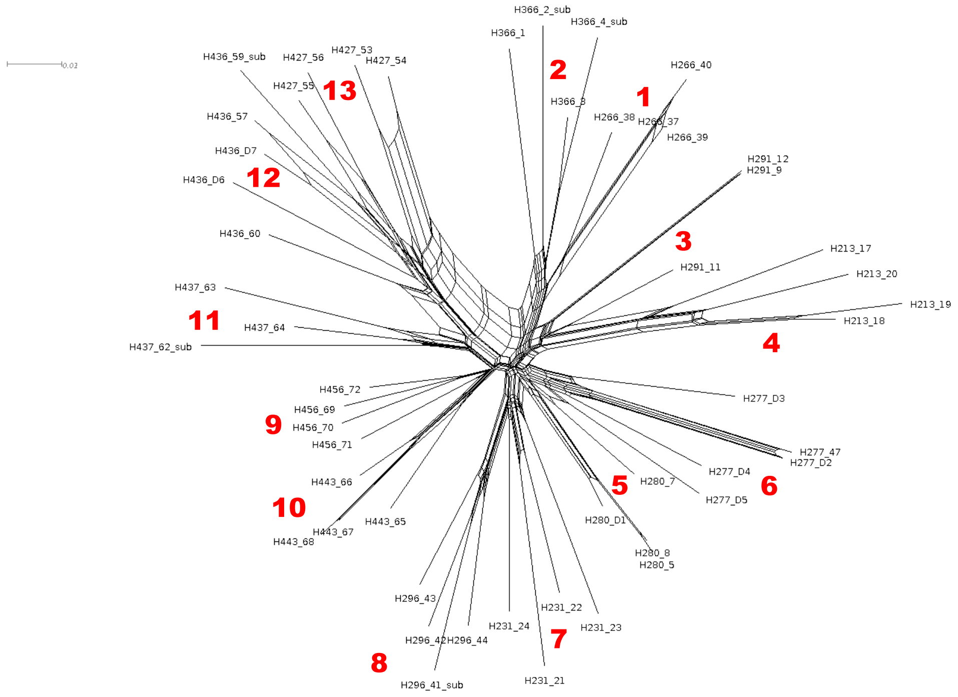

Onosma microcarpa

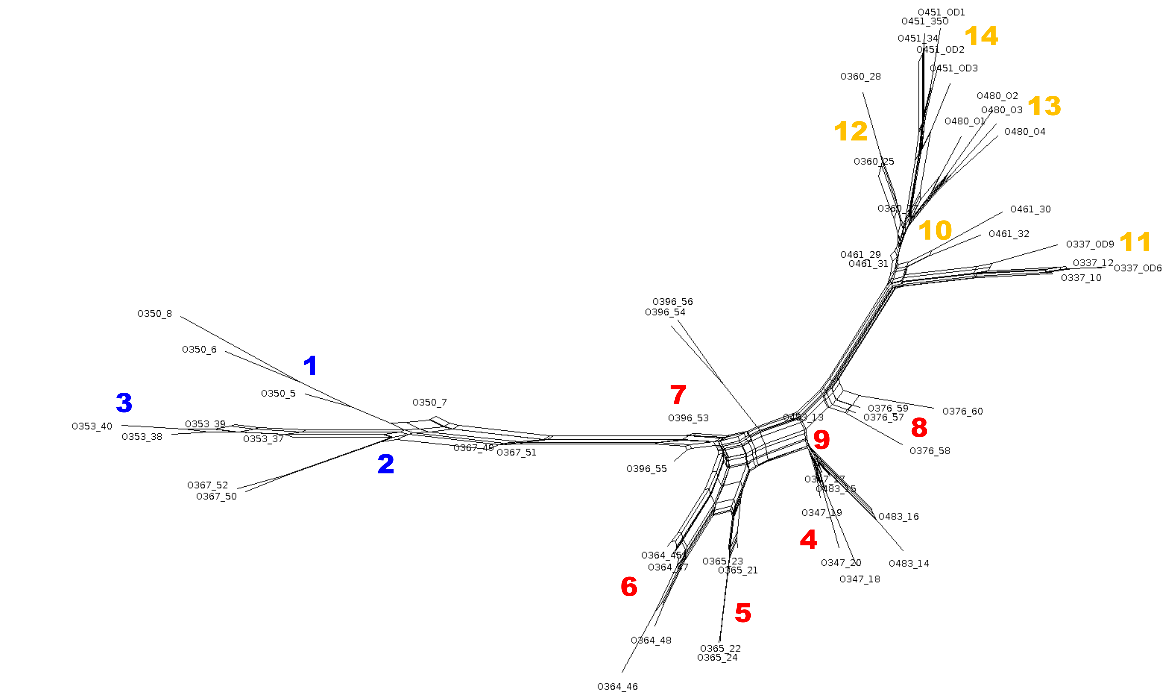

Phlomis olivieri

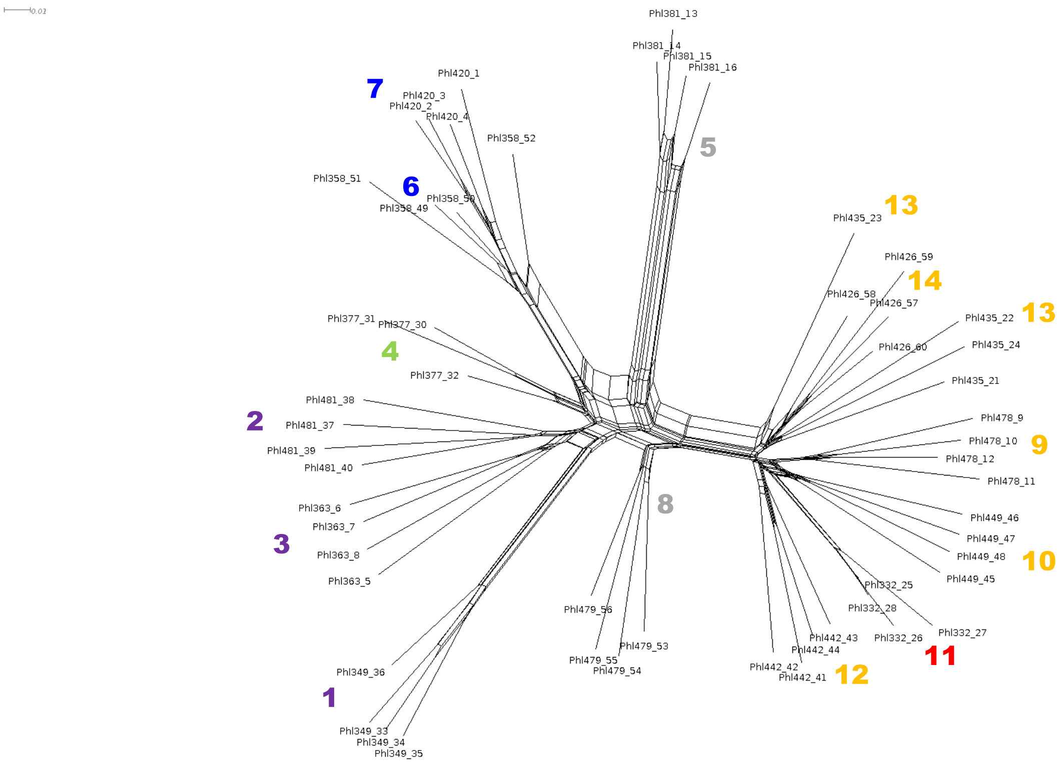

Crepis heterotricha

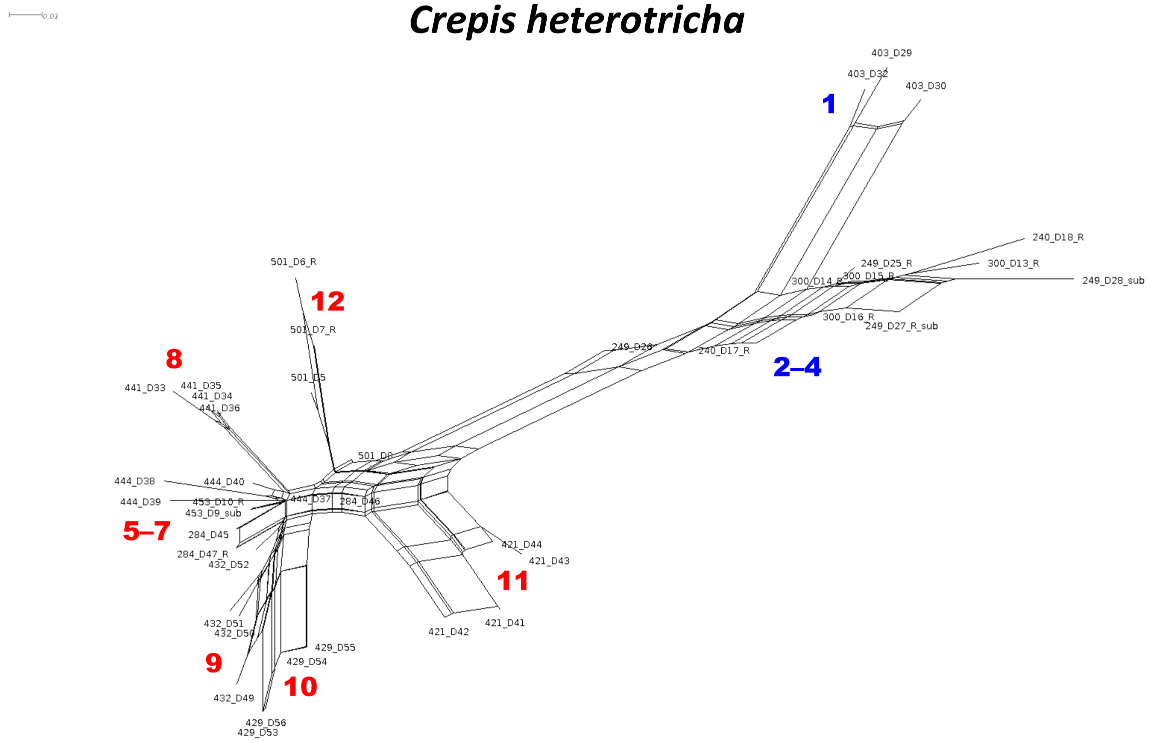

Didymophysa aucheri

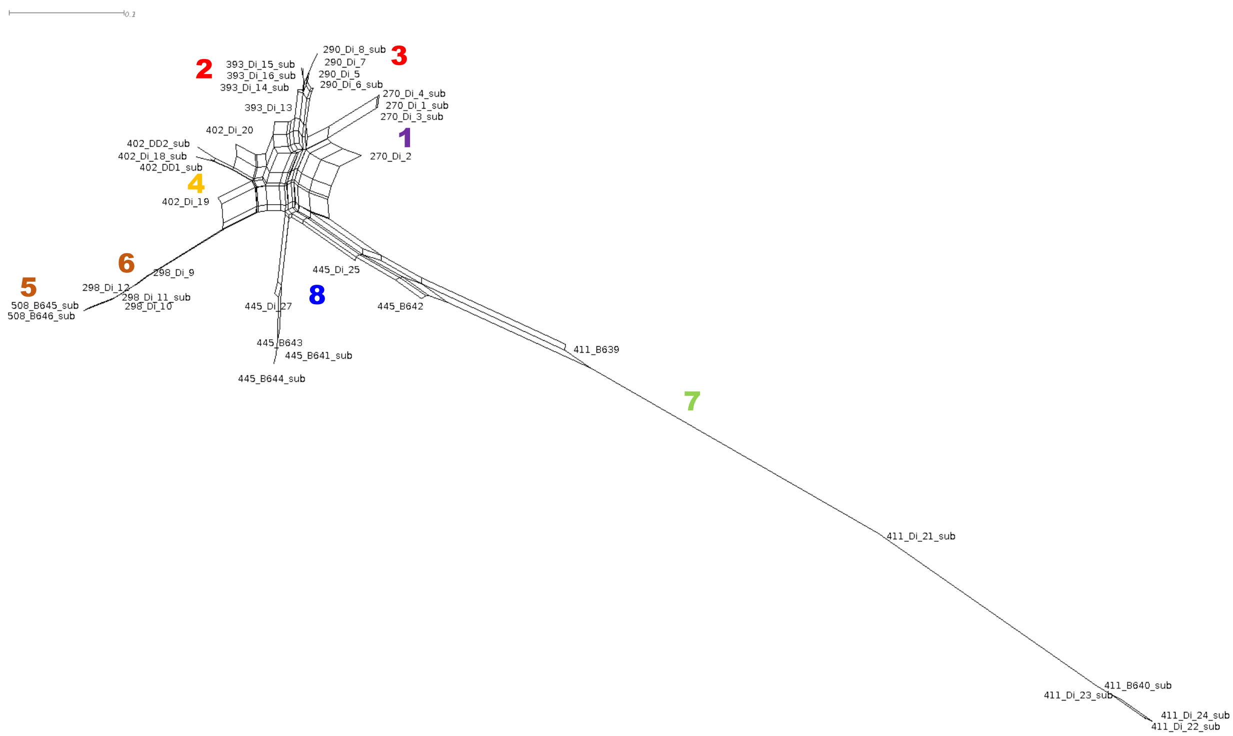

Dielsiocharis kotschyi

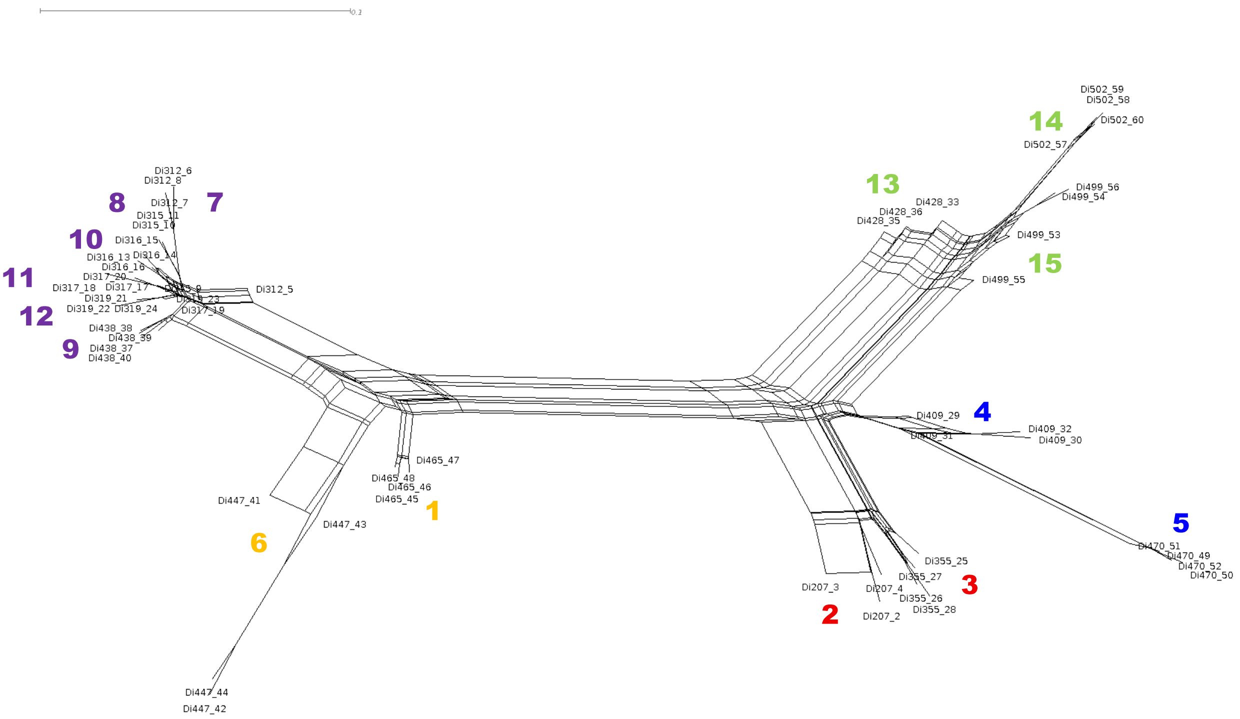

Physoptychis gnaphalodes

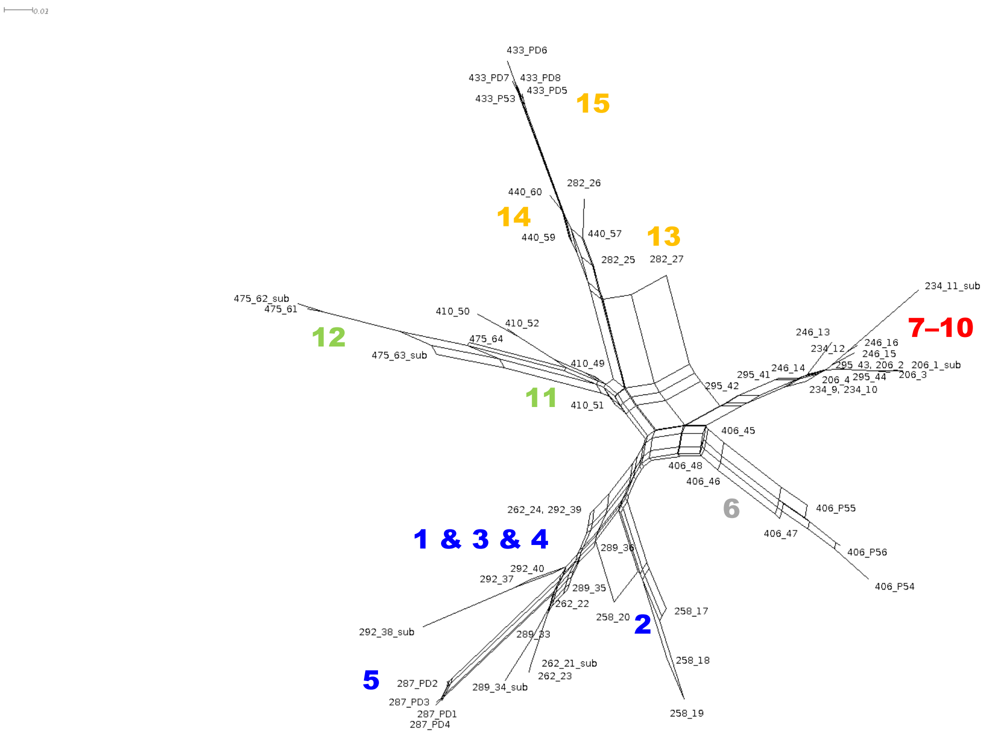

Tanacetum kotschyi

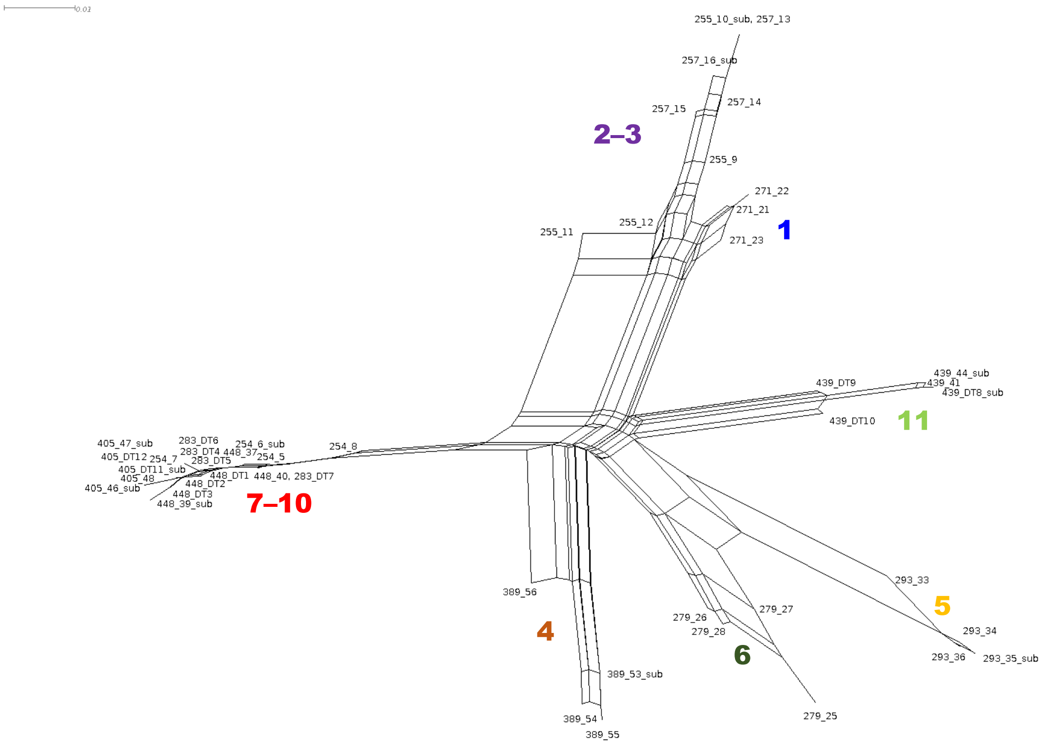

Supplement: Supplementary file 3 — Figure S1: Results of TESS3 analyses. For each species, the following information is shown: plot of the cross‐validation score across different K values (1–10); plot of the difference in mean cross validation error across different K values (2–10); histogram of ancestry coefficients for different K values (2–10); diffusion map of the genetic groups for different K values (2–10). Figure S2: Neighbour‐nets based on HKY distances (see main text for details). Colors of population numbers correspond to those used for genetic groups identified by TESS3 (see Figure 2; strongly admixed populations shown in grey). Species are arranged according to elevational zone (montane species, alpine species) and, within each zone, alphabetically. Figure S3: Principal component analysis (PCA). Colors of population numbers and of polygons delimiting genetic groups correspond to those used for genetic groups identified by TESS3 (see Figure 2; strongly admixed populations shown in grey). Species are arranged according to elevational zone (montane species, alpine species) and, within each zone, alphabetically. Figure S4: Phylogeographic barriers identified using Monmonier's maximum difference algorithm on population networks (left column: Delaunay triangulation; right column: Gabriel graph), where population distances are calculated from Nei distances (upper row) or Roger's distance (lower row; see main text for details). Arrows indicate the directionality of the path (from larger to smaller distances), different colors (blue, red) indicating distinct paths with the same starting point. Edges in orange (in Allium scabriscapum only) indicate a set of edges jointly corresponding to a single genetic boundary geographically placed at the lowlands between Alborz (north) and Zagros Mountains (south). Species are arranged according to elevational zone (montane species, alpine species) and, within each zone, alphabetically. Table S1: List of populations of all species, their collected locations, da [file MEC-35-e70355-s001.zip › Figure_S2.pdf]
